# Exploring the Denitrification Proteome of *Paracoccus denitrificans* PD1222

**DOI:** 10.3389/fmicb.2018.01137

**Published:** 2018-05-29

**Authors:** Alfonso Olaya-Abril, Jesús Hidalgo-Carrillo, Víctor M. Luque-Almagro, Carlos Fuentes-Almagro, Francisco J. Urbano, Conrado Moreno-Vivián, David J. Richardson, María D. Roldán

**Affiliations:** ^1^Departamento de Bioquímica y Biología Molecular, Universidad de Córdoba, Córdoba, Spain; ^2^Departamento de Química Orgánica, Universidad de Córdoba, Córdoba, Spain; ^3^Servicio Central de Apoyo a la Investigación, Unidad de Proteómica, Córdoba, Spain; ^4^School of Biological Sciences, University of East Anglia, Norwich, United Kingdom

**Keywords:** acetyl-CoA, denitrification, liquid chromatography-mass spectrometry, nitrate reductase, nitrous oxide, nitrous oxide reductase, *Paracoccus*, proteomics

## Abstract

Denitrification is a respiratory process that produces nitrous oxide as an intermediate, which may escape to the atmosphere before its reduction to dinitrogen through the nitrous oxide reductase NosZ. In this work, the denitrification process carried out by *Paracoccus denitrificans* PD1222 has been explored through a quantitative proteomic analysis. Under anaerobic conditions, with nitrate as sole nitrogen source, the synthesis of all the enzymes involved in denitrification, the respiratory nitrate, nitrite, nitric oxide, and nitrous oxide reductases, was increased. However, the periplasmic and assimilatory nitrate reductases decreased. Synthesis of transporters for alcohols, D-methionine, sulfate and copper, most of the enzymes involved in the tricarboxylic acid cycle, and proteins involved in other metabolic processes like lysine catabolism, fatty acids degradation and acetyl-CoA synthesis, was increased during denitrification in *P. denitrificans* PD1222. As consequence, an enhanced production of the central metabolite acetyl-CoA was observed. After establishing the key features of the denitrification proteome, its changes by the influence of a competitive electron acceptor, oxygen, or competitive nitrogen source, ammonium, were evaluated.

## Introduction

Denitrification is the respiratory reduction of the water soluble nitrogen oxyanions, nitrate (NO_3_^-^) and nitrite (NO_2_^-^), to the gaseous products nitric oxide (NO), nitrous oxide (N_2_O) and molecular nitrogen (N_2_) under oxygen-limited conditions through four reactions whereby 10 electrons are consumed in converting two nitrate ions to dinitrogen molecule (Zumft, 1997; Richardson, 2000; Philippot et al., 2007; van Spanning et al., 2007; Bakken et al., 2012; Hu et al., 2015). The complete process involves the enzymes nitrate reductase (Nar), nitrite reductase (Nir), nitric oxide reductase (Nor) and nitrous oxide reductase (Nos). The functioning of these enzymes is conditioned by both the need to regulate the process avoiding accumulation of toxic intermediates, mainly nitric oxide, and to maximize energy conservation when oxygen is fluctuating (Bergaust et al., 2008, 2010; Sullivan et al., 2013). The reduction of nitrite to nitric oxide by the nitrite reductase defines the denitrification process since it represents the conversion of a non-gaseous water-soluble nitrogen oxyanion to a nitrogen oxide gas.

Biological denitrification is a major biological process for producing the potent greenhouse gas N_2_O and it accounts globally for about 60% of total N_2_O emissions to the atmosphere. The denitrification pathway has been extensively studied, and several key factors have been described for controlling nitrous oxide/molecular nitrogen ratio in soils, such as oxygen levels, pH, temperature, water content and nitrate or carbon substrate availability (Bergaust et al., 2010). Copper concentration in the media has been also described to be essential to achieve an adequate synthesis of the nitrous oxide reductase (NosZ) of *P. denitrificans* PD1222 because this enzyme contains a Cu_z_ active center, which is a multi-copper-sulfide [Cu_4_S] cluster (Richardson et al., 2009; Sullivan et al., 2013). The highest denitrification activity is observed under oxygen-limitation or anaerobic conditions, although aerobic denitrification has also been described in some bacteria isolated from soils and sediments (Patureau et al., 2000; Hu et al., 2015). Field experiments with bacterial denitrifiers have revealed that low pH increases nitrous oxide emissions (Richardson et al., 2009; Shcherbak et al., 2014; Hu et al., 2015). It has been demonstrated that nitrous oxide reduction is severely reduced under acidic pH, suggesting an effect of pH on nitrous oxide reductase at transcriptional or posttranscriptional level (Wrage et al., 2001; Bakken et al., 2012; Hu et al., 2015).

The α-proteobacteria *Paracoccus denitrificans* PD1222 (Baker et al., 1998) is a model soil microorganism able to perform the complete denitrification pathway, in which nitrate reduction is molybdenum-dependent (membrane-bound and periplasmic nitrate reductases), nitrite and nitric oxide reduction are iron-dependent (cytochrome *cd*_1_-type nitrite reductase or heme *c/b* nitric oxide reductase) and nitrous oxide reduction is copper-dependent (Cu_z_-nitrous oxide reductase). All these reductases can be coupled to the core electron transport pathway at the level of the ubiquinol pool in the membrane and the periplasmic heme-containing cytochrome *c*_550_ or Cu-containing pseudoazurin pool (Richardson, 2000; Felgate et al., 2012; Giannopoulos et al., 2017). Denitrification is negatively controlled in response to oxygen by FnrP and positively regulated in response to nitric oxide by Nnr. Recently, it has been suggested that FnrP can also be negatively regulated by nitric oxide (Crack et al., 2016). In addition, NarR, NirI, and NosR specifically regulate expression of the *nar*, *nir*, and *nos* genes, respectively (Saunders et al., 1999; van Spanning et al., 1999; Wood et al., 2001; Bergaust et al., 2012; Spiro, 2012). A recent genomic analysis of steady-state chemostat cultures revealed that the transition from aerobic to anaerobic growth supported by denitrification resulted in the up- or down-regulation (≥2-fold) of just 4% of the genome, with 157 upregulated genes putatively involved in the synthesis or activity of the *P. denitrificans* respirome, including the terminal reductases (Giannopoulos et al., 2017). This represents a very efficient adaptation from aerobic to anaerobic metabolism.

To date, much of the experimental work addressing the regulation of denitrification has been focused on transcriptional studies. However, to understand regulation in more detail it is important to get a perspective on protein synthesis, and therefore differences in the proteome, rather than just in the transcriptome of the denitrification. This is important because a twofold change in gene expression (the threshold used for significance in transcriptomics), does not necessarily lead to an equivalent change in levels of the relevant proteins, and indeed transcriptional analyses supply no information at all on protein levels. Thus, for a full (holistic) picture of the denitrification process, a global overview of all the denitrification proteins, most of them complex metalloproteins, is required. Second-generation proteomics uses gel-free approaches, based on liquid chromatography-mass spectrometry/mass spectrometry (LC-MS/MS) analysis for the identification of thousands of species (generally peptides, after sample treatment with a protease) in a liquid sample. The wide array of combinations derived from these approaches makes it possible to overcome the limitations attributed to gel-based proteomics regarding membrane proteins, as well as a more accurate quantification through both labeling and non-labeling strategies (Speers and Wu, 2007). In this work, a proteomic study of *P. denitrificans* PD1222 has been carried out under nitrate-respiring denitrifying conditions and used as reference point from which to consider the impact of the additional nitrogen source ammonium, and the additional electron acceptor oxygen.

## Results and Discussion

### Defining the Growth Physiology of *P. denitrificans* Prior to Proteomic Analyses

Prior to undertaking the proteomic study, the experimental growth conditions for culturing *P. denitrificans* under: (i) anaerobic denitrifying conditions with nitrate as electron acceptor and sole N-source (Den-N); (ii) anaerobic denitrifying conditions with nitrate as electron acceptor and with both ammonium and nitrate as potential N-sources (Den-NA); and (iii) aerobically with both oxygen and nitrate as potential electron acceptors and nitrate as a sole N-source (Aer-N) were established. N-oxyanion concentrations and N-gas emissions were determined in bacterial cultures in early exponential phase (A_600_ ∼0.3) and stationary phase (A_600_ ∼0.9) of the growth curves (**Table 1**). In Den-N cultures (30 mM nitrate, 30 mM succinate) at early growth phase (A_600_ ∼0.3), about 11 mM nitrate remained in the media, whereas at stationary growth phase (A_600_ ∼0.9) nitrate was completely exhausted and mainly transformed to N_2_ (**Table 1**). In the Den-NA cultures at early logarithmic phase only a small amount of nitrate was converted into dinitrogen. However, at the stationary growth phase (A_600_ ∼0.9) ammonium was completely exhausted and almost all nitrate was denitrified to N_2_ (**Table 1**). Nitrite accumulated was ∼6-fold higher (∼600 μM) under Den-N conditions than in Den-NA conditions (**Table 1**). This high level of extracellular nitrite accumulation was also observed under Aer-N conditions, suggesting a link between nitrite extrusion and the process of assimilating nitrogen from nitrate under both aerobic and anaerobic growth conditions. The gaseous intermediates nitric oxide and nitrous oxide were not detected (<1 μM) under either Den-N or DenNA growth conditions. This suggests that denitrification was proceeding all the way through to dinitrogen gas as the end-product. In Aer-N cultures, neither dinitrogen nor nitric oxide/nitrous oxide intermediates were detected at significant levels, indicating suppression of denitrification. This is consistent with the cultures being highly aerated, with all nitrate used therefore assignable to nitrogen assimilation (**Table 1**).

**Table 1:** Concentration of nitrate, nitrite, ammonium, and gasses of denitrification in *P. denitrificans* PD1222.

Sample^∗^	A_600_	Protein (μg ml^-1^)	NO_2_^-^ accumlation (μM)	NO_3_^-^ consumed (mM)	NH_4_^+^ consumed (mM)	N_2_ produced (mM)
Den-N	0.30 ± 0.01	32.4 ± 2.8	35.6 ± 4.2	19.2 ± 0.4	–	7.3 ± 0.2
Den-N	0.91 ± 0.02	219.8 ± 4.3	641.6 ± 2.9	30 ± 0	–	14.2 ± 1.0
Den-NA	0.30 ± 0.01	28.6 ± 1.2	124.7 ± 4.1	2.7 ± 1.9	4.7 ± 0.8	1.4 ± 0.4
Den-NA	0.93 ± 0.02	226.9 ± 0.3	109.5 ± 11.3	26.6 ± 0.1	10 ± 0	16.7 ± 0.3
Aer-N	0.30 ± 0.01	30.5 ± 1.0	616.0 ± 22.4	1.2 ± 0.3	–	0 ± 0
Aer-N	0.91 ± 0.01	222.6 ± 0.9	646.04 ± 1.6	10 ± 0	–	0 ± 0


**^∗^Den-N, cells grown in anaerobiosis with 30 mM nitrate; Den-NA, cells grown in anaerobiosis with 30 mM nitrate plus 10 mM ammonium; Aer-N, cells grown in aerobiosis with 10 mM nitrate. Samples were taken at early (A_600_ ∼0.3) or late (A_600_ ∼0.9) exponential growth phase. Data are means ± DE of 2 independent biological samples, and from each of these, 2 methodological replicates (*n* = 4). N-ions and denitrification gasses were determined as indicated in experimental procedures. NO and N_2_O were not detected.**

### The Proteome of *P. denitrificans* PD1222 During Anaerobic Denitrification With Nitrate as Sole Nitrogen Source

**FIGURE 1:**
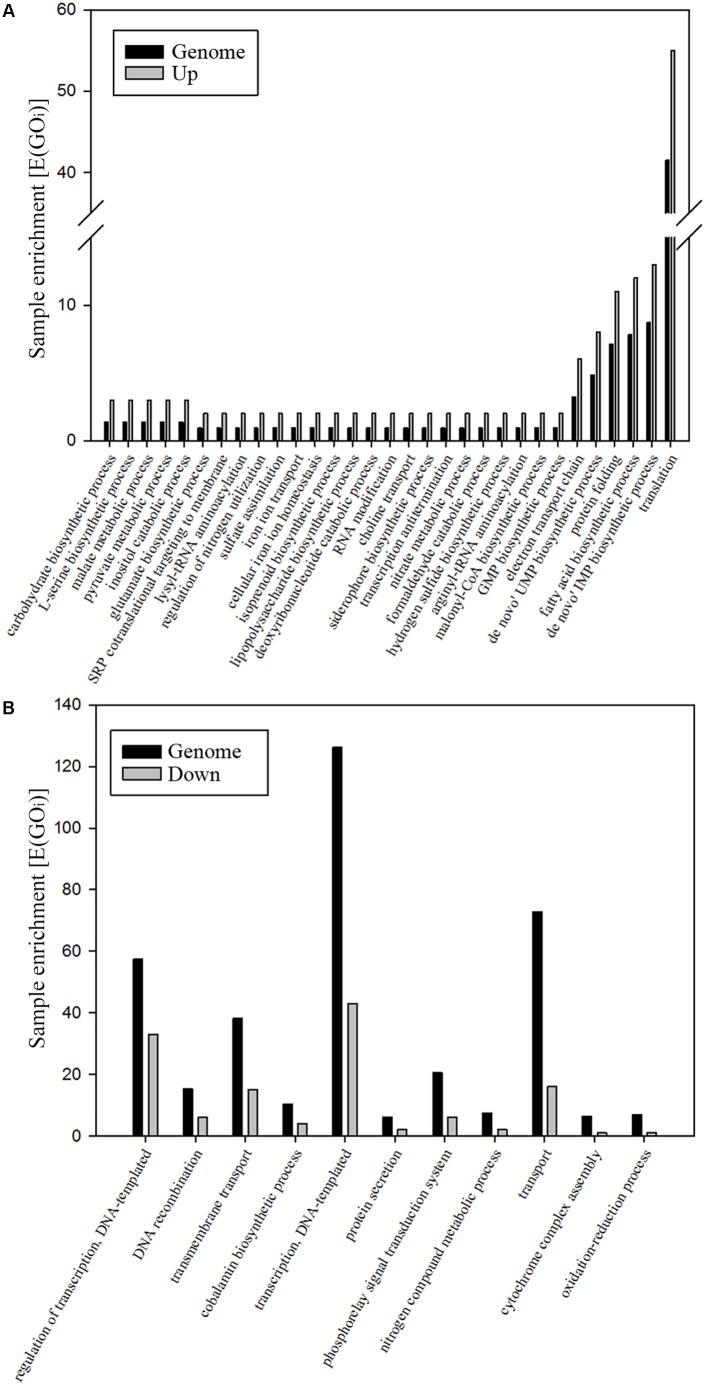
Analysis of the denitrification proteome of *P. denitrificans* PD1222. Significative changes in GO groups. Only those changes with a *p-*value < 0.05 are shown. The whole genome of *P. denitrificans* PD1222 was used as reference (black bars) and over-represented **(A)** and down-represented **(B)** GO groups are shown (gray bars). Hyper-geometric distribution [E(GOi)] of GO (ComparativeGO), biological function, third level of proteins identified under denitrifying conditions. The parameter E(GOi) is calculated by using the formula: [E(GOi)] = sample size/genome size × GOi.

**FIGURE 2:**
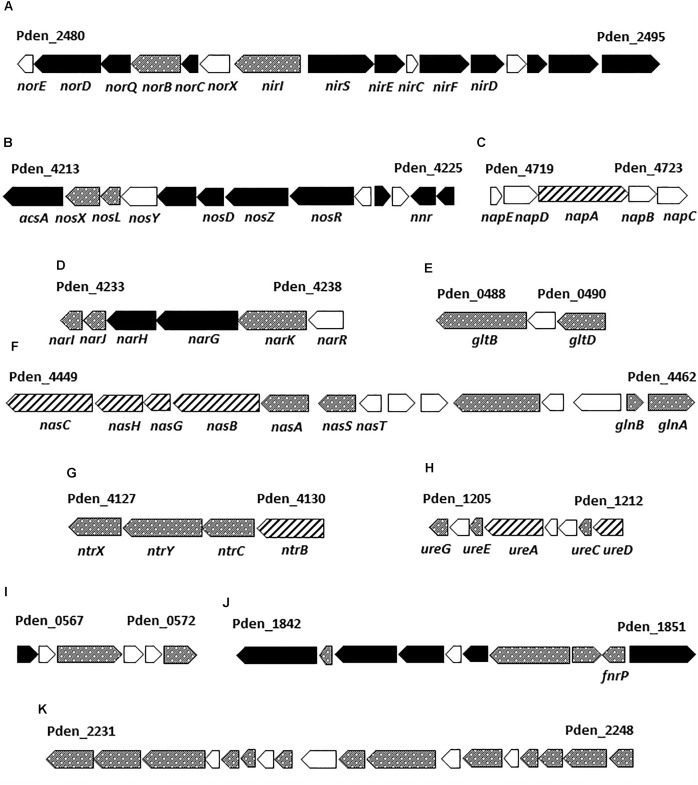
*Paracoccus denitrificans* PD1222 genes encoding several proteins detected by LC-MS/MS under denitrifying conditions. The reference codes correspond to the first and the last gene of each gene cluster according with UniProt (UP000000361). Gene-filling: black, genes encoding proteins found in abundance under anaerobic denitrifying conditions when compared to aerobic cultures; dotted, genes that code for proteins of unchanged levels in the presence or absence of oxygen; dashed, genes encoding proteins displaying low abundance under anaerobic conditions. Gene cluster descriptions are provided in Supplementary Figure S2.

To gain insight into the metabolism of *P. denitrificans* when using nitrate as both electron acceptor and nitrogen source, the strain PD1222 was grown under anaerobic conditions with 30 mM succinate as carbon source and 30 mM nitrate as N-source, in the absence of ammonium (Den-N cultures). When cultures reached an absorbance at 600 nm of approximately 0.3, cells were harvested and processed to perform a LC-MS/MS analysis of the global proteome in response to denitrifying conditions. Two independent cultures were set up, and from each one two independent samples were prepared from each culture for a full proteome analysis. A total of 1293 proteins were reproducibly identified from ∼5100 putative structural genes present in the whole genome of the PD1222 strain (Supplementary Table S1), giving a ∼25% representation of the genome in the detectable proteome. Breaking down the proteome into gene ontology (GO) groups can give some insight into functional categories of proteins that are either enriched (highly represented) or suppressed (poorly represented) under denitrifying and nitrate assimilating conditions (**Figure 1A**). Highly represented GO groups included ‘carbohydrate biosynthesis,’ ‘malate and pyruvate metabolism,’ ‘regulation of nitrogen utilization,’ ‘nitrate metabolic process,’ ‘electron transport chain,’ ‘protein folding,’ ‘RNA modification,’ ‘transcription anti-termination and translation.’ Poorly represented GO groups (**Figure 1B**) included ‘cobalamin biosynthesis,’ ‘nitrogen compound metabolism,’ ‘cytochrome complex assembly,’ ‘phosphorelay signal transduction,’ ‘DNA recombination’ and ‘regulation of transcription and translation.’ Some of these proteins, encoded by genes located in several notable gene clusters, merit specific mention (**Figure 2**).

Firstly, most of the structural proteins required for denitrification were detected, including the respiratory nitrate reductase system NarKGHJI (A1B9V7-A1B9V3), the periplasmic nitrate reductase NapA (A1BB88), the *cd*_1_-type nitrite reductase NirS (Q51700), the nitric oxide reductase NorBC (A1B4X6 and A1B4X7), the nitrous oxide reductase NosZ (A1B9T9), and the Cu-protein pseudoazurin (Q71RW5). Regarding regulatory proteins, the general transcriptional regulators of denitrification involved in the *P. denitrificans* NO-response Nnr (A1B353), the O_2_-responsive FnrP (A1B9U4) and the system-specific regulatory proteins of the nitrite, nitric oxide and nitrous oxide reductases NirI (Q51699) and NosR (A1B9U0) were also detected (Supplementary Table S1).

Secondly, several proteins involved in assimilatory nitrate reduction were identified, including the structural NasABGHC proteins (A1BAH3-A1BAH0, A1BAG9) and the regulatory protein NasS (A1BAH4) that acts as a nitrate sensor (Gates et al., 2011; Luque-Almagro et al., 2013), along with proteins functionally associated with the nitrogen starvation response, such as the global regulatory proteins NtrBC (A1B9K0, A1B9J9), NtrYX (A1B9J8, A1B9J7) and PII-GlnB (A1BAI1), glutamine synthetase GlnA (A1BAI2), glutamate synthase large and small subunits (A1AZA6 and A1AZA8), urease structural and accessory proteins (A1B1B6, A1B1B8, A1B1B9, and A1B1C2), and the transporters of ammonium and some amino acids (A1B3N0, A1B061, A1B064, and A1B066) (Supplementary Table S1). Under denitrifying conditions in the presence of nitrate as the sole nitrogen source, the main fate of nitrate is to serve as end-electron acceptor, but some also needs to be assimilated *via* ammonium. NasH (A1BAUD) is a putative nitrite extrusion protein that may play a role in nitrite homeostasis during nitrate assimilation and, therefore could contribute to the high levels of extracellular nitrite observed in Den-N cultures (**Table 1**).

Thirdly, regarding central metabolic pathways, many enzymes of the Krebs and glyoxylate cycles could be detected in this proteomic study during denitrification, including malate dehydrogenase (A1B0K8), fumarate hydratase (A1B3A9), succinate and isocitrate dehydrogenases (A1AZI5, A1B6A1), aconitase (A1BAT6) and malate synthase (A1B9C3). Also, different components of the respiratory electron transport chain like proteins belonging to complex I-NADH-quinone oxidoreductase (A1B481, A1B486, A1B489, A1B491, A1B495, A1B496), complex II-succinate dehydrogenase (A1AZJ0, A1AZI7) and the cytochrome *cbb*_3_-type cytochrome *c* oxidase (A1B348 and A1B350) were detected under denitrifying conditions (Supplementary Table S1).

Fourthly, several membrane-bound transporters were highly represented under anaerobic denitrifying conditions, including those specific for carbon compounds (e.g., the C4-dicarboxylate transporter A1BBD2 and the ribose/galactose/methylgalactoside import ATP-binding protein A1B2N6) and, also for D-methionine (A1BA28). Microorganisms able to synthesize *de novo* methionine may require vitamin B_12_ as cofactor. Thus, a B_12_-dependent methionine synthase catalyzes the conversion of 5-methyltetrahydrofolate and L-homocysteine to tetrahydrofolate and L-methionine. In *P. denitrificans* PD1222 it has been recently demonstrated that cytotoxic N_2_O emissions modulate expression of genes controlled by vitamin B_12_ riboswitches, because N_2_O binds to and inactivates vitamin B_12_. Cytotoxicity of N_2_O was relieved by the addition of vitamin B_12_ to the media (Sullivan et al., 2013). Therefore, the import of methionine by the A1BA28 transporter could be a mechanism to bypass the cobalamin dependence of *de novo* biosynthetic process. Copper-limitation has a negative effect on the expression of the *nos* genes, leading to accumulation of N_2_O and inhibition of vitamin B_12_ dependent-enzymes (Sullivan et al., 2013). The copper-binding periplasmic (A1B97T8) and the membrane (A1B97T7) components of an ABC transporter involved in NosZ maturation (Saunders et al., 2000) were found highly induced under denitrifying conditions in the strain PD1222. Although copper is a trace element essential for all aerobic organisms, it could exert toxicity to the cell when its homeostasis is not maintained. In this sense, a P-type copper extrusion system (A1B375) was highly induced under anaerobic growth in *P. denitrificans* PD1222, suggesting that copper needs to be transported inside the cell to favor its incorporation to metalloproteins, including NosZ, and therefore this metal extrusion system is induced under denitrifying conditions to allow copper homeostasis in the cell. It is notable that the Pden_1842 gene coding for this metal extrusion component is located near the genes encoding the copper-containing *cbb*_3_-type cytochrome oxidase (Pden_1845-Pden_1848) and the Pden-1850 gene that codes for the transcription regulator FnrP involved in the aerobic-anaerobic transition (**Figure 2**).

### Changes in the Denitrification Proteome of *P. denitrificans* PD1222 in Response to Ammonium as Additional N-source

**FIGURE 3:**
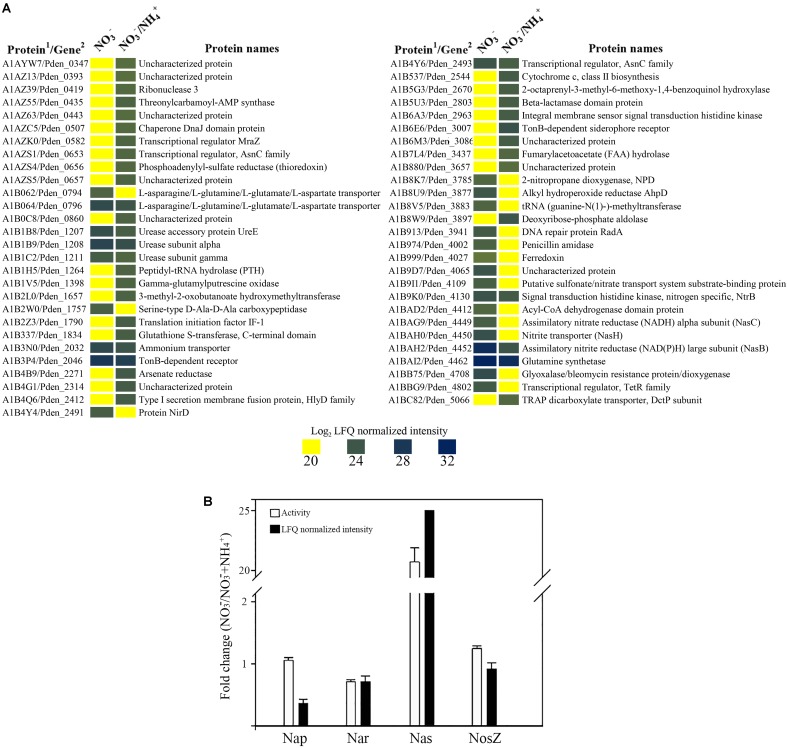
Effect of ammonium on the denitrification proteome of *P. denitrificans* PD1222. Heatmap of over-represented (blue) or down-represented (yellow) proteins (log_2_ normalized expression) of *P. denitrificans* PD1222 in the presence or absence of ammonium during denitrification. Proteomic data from cells grown with 30 mM nitrate were compared to data from cells grown with 30 mM nitrate plus 10 mM ammonium. (^1^) Protein code according to Uniprot database under the accession number UP000000361. (2) Gene code according to Genebank under the accession number CP000489.1 **(A)**. Fold change of Nar, Nap, Nas, and NosZ proteins. White bars represent enzymic activity and black bars show normalized peptide intensities. Fold changes were shown using the enzymic activity or peptide intensity ratio (NO_3_^-^/NO_3_^-^ + NH_4_^+^) **(B)**, error bars correspond to data from at least 3 independent replicates.

*Paracoccus denitrificans* was grown under anaerobic conditions with 30 mM succinate as carbon source and 30 mM nitrate as nitrogen source and electron acceptor, plus 10 mM ammonium as additional nitrogen source (Den-NA), and the global proteome was analyzed by LC-MS/MS as described above. Two independent cultures were set up and from each one two independent samples were prepared (Supplementary Figure S2). Variability within samples was assessed by heat maps and volcano plots (Supplementary Figure S1A). Then, a differential proteomic study was performed by comparison of data from cells grown with nitrate as sole nitrogen and electron acceptor (Den-N) with data from cells grown with nitrate plus ammonium (Den-NA). One thousand three hundred and eighty four proteins were detected from the Den-NA cultures, representing 27% of the total potential proteome (Supplementary Table S2). Of these, only 55 proteins (4% of the detectable proteome) significantly changed their intensities (26 proteins with increased expression and 29 with decreased expression) compared to in the absence of ammonium (**Figure 3A**). This reflects a very efficient adaptation to utilization of ammonium, rather than nitrate, as the primary nitrogen source.

Among the 29 proteins with decreased intensities under Den-NA conditions were peptides of the assimilatory nitrate (NasC, A1BAG9) and nitrite (NasB, A1BAH2) reductases, the nitrite transporter (NasH, A1BAH0) and the transcriptional regulators NtrB (A1B9K0) and NirD (A1B4Y4). NirD is involved in the biosynthesis of the heme *d*_1_ from precorrin 2. The heme *d*_1_ is required for the active site of NirS. Precorrin 2 is also an intermediate in siroheme biosynthesis, the active site cofactor for the large subunit of the assimilatory nitrite reductase NasB. It is likely therefore that NirS and NasB biosynthesis competes for precorrin 2. Levels of NirD may play an important role in the routing of precorrin 2 to siroheme or heme *d*_1_ biosynthesis (Bali et al., 2011), which may impact in the apparent suppression by ammonium of onset of denitrification in the early stages of the growth cultures as illustrated in **Table 1**.

NtrB is a sensor kinase that autophosphorylates a histidine residue under low nitrogen conditions and, in turn, transfers a phosphoryl group on a specific aspartate residue to the response regulator NtrC. Phosphorylated NtrC acts as σ^54^-dependent transcriptional activator of different genes related with nitrogen metabolism (Sanders et al., 1992; Luque-Almagro et al., 2011). In *P. denitrificans* it has been recently demonstrated that the NtrBC system controls, in response to ammonium, the glutamine synthetase *glnA* gene, the ammonium transporter *amtB* gene, the urease *ure* genes and the nitrate/nitrite assimilation *nas* genes, among others (Luque-Almagro et al., 2017). Although the regulation exerted by ammonium through the NtrBC system occurs at the post-transcriptional level, *P. denitrificans* PD1222 NtrB was found at a lower peptide intensity in the presence of ammonium than in its absence (**Figure 3A**), and this could also contribute to reduce the expression of genes with a predicted NtrC-binding site. A putative nitrate/sulfonate transporter (A1B9I1) was also decreased in representation, which may suggest a role for this protein in nitrate or nitrite uptake (**Figure 3A**).

The 26 proteins with increased intensities included a transcriptional regulator belonging to the AsnC family (A1AZS1) encoded by the Pden_0653 gene that clusters together with a putative heme-containing nitrite/sulfite reductase gene (Pden_0655) and a thioredoxin (A1AZS4) encoded by Pden_0656. A protein involved in cytochrome *c* class II biosynthesis, which is encoded by the Pden_2544 gene that clusters together with cobyrinate *a,c*-diamide synthase (Pden_2541) and precorrin-6A synthesis (Pden_2542) genes also displayed increased peptide intensity.

When the ratios of normalized peptide intensities of the three nitrate reductases (respiratory Nar, periplasmic Nap and assimilatory Nas), and the nitrous oxide reductase NosZ were calculated for the Den-N versus Den-NA cultures, it was clear that the synthesis of assimilatory nitrate reductase was negatively affected by the presence of ammonium in the media (Den-NA cultures), with an increase of ∼25-fold observed in lysates from Den-N cultures compared to Den-NA conditions. This data was also supported by determination of the assimilatory nitrate reductase activity in cytoplasmic fractions using NADH as electron donor in the enzymic assay (**Figure 3B**).

Gas emissions were measured during denitrification (**Table 1**). In the absence of ammonium, almost all nitrate reduced was converted into dinitrogen without accumulation of intermediate gasses, and only a low amount of nitrate was used for assimilative purposes. However, when ammonium was present in the media, the production of N_2_ was very low at early exponential growth phase, and only when ammonium was consumed was denitrification maximal (**Table 1**). This finding may have an ecophysiological impact on microbial communities of nitrifiers *versus* denitrifiers and in denitrification itself that could be performed with both nitrate and ammonium coexisting in agricultural soils when they are used as crop fertilizers.

### Effect of Oxygen on the Denitrification Proteome of *P. denitrificans* PD1222

**FIGURE 4:**
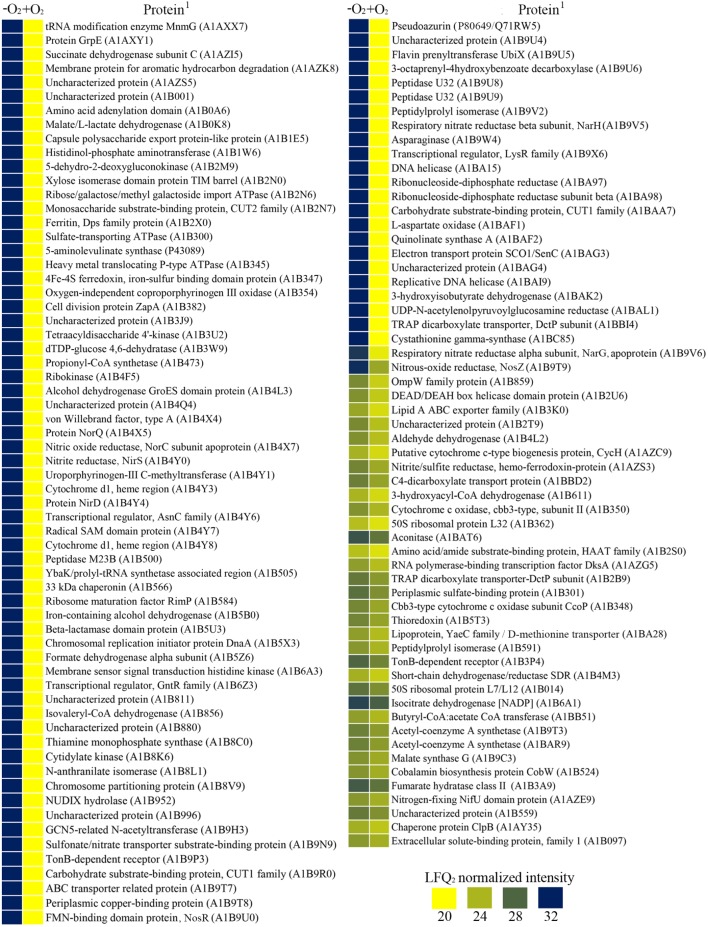
Exclusive and over-represented proteins of *P. denitrificans* PD1222 under anaerobic-denitrifying conditions with nitrate as the sole nitrogen source. Data were obtained from a comparative analysis from cells cultured with or without oxygen in the presence of nitrate as the sole nitrogen source. Results are shown in a scale color from dark blue to yellow as log_2_ normalized expression. (^1^) Protein code according to Uniprot database under the accession number UP000000361.

**FIGURE 5:**
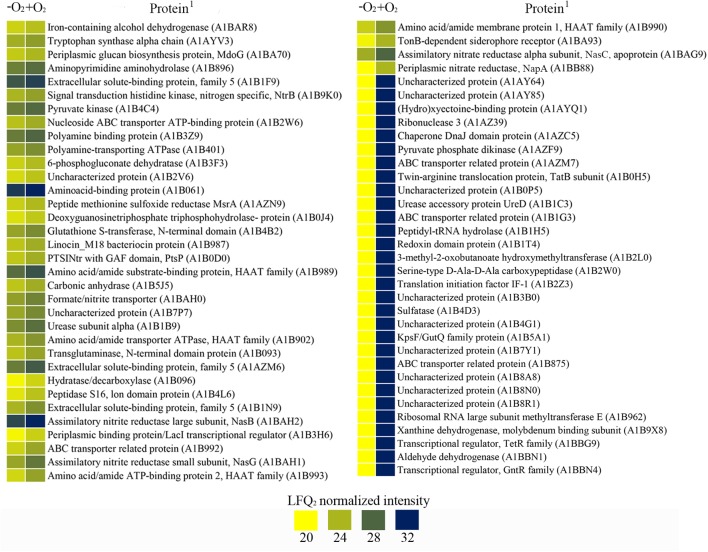
Exclusive and over-represented proteins of *P. denitrificans* PD1222 under aerobiosis when nitrate was used as the sole nitrogen source. Data were obtained from a comparative analysis from cells grown with or without oxygen. Results are shown in a scale color from dark blue to yellow as log_2_ normalized expression. (^1^) Protein code according to Uniprot database under the accession number UP000000361.

A proteomic study of *P. denitrificans* cells grown in aerobic conditions with nitrate as sole N-source (Aer-N) was also performed (Supplementary Table S3). Variability within samples was shown by heatmap and volcano plots (Supplementary Figure S1B). Hundred and ninety one proteins were identified with significant changed levels in the comparative proteomic analysis carried out from cells cultured with nitrate as the nitrogen source and electron acceptor in the absence (Den-N) or presence (Aer-N) of oxygen (**Figures 4**, **5**). Of these 87 proteins were only found under anaerobic conditions (as defined here by fold change > 552 in anaerobiosis compared to aerobiosis), and 36 proteins were up-represented in anaerobiosis (**Figure 4**). In addition, 38 proteins were down-represented under anaerobiosis and 30 proteins were exclusive to aerobic conditions as defined by a fold change > 13 in aerobiosis compared to anaerobiosis (**Figure 5**).

Peptides exclusively detected in anaerobic cultures (**Figure 4**) corresponded to the respiratory nitrate reductase NarGH (A1B9V6, A1B9V5), the *cd*_1_-type nitrite reductase NirS (A1B4Y0), the nitric oxide reductase subunit NorC (A1BAX7), and the proteins NorQ (A1B4X5), NirD (A1B4Y4) and pseudoazurin (Q71RW5). Other peptides only found in anaerobiosis were the periplasmic components of several alcohol transporters (A1B9R0, A1BAA7, A1B2N6, and A1B2N7), the cytoplasmic ATPase component of a sulfate transporter (A1B300), a copper ABC transporter component (A1B9T7), a heavy metal (copper) translocating P-type ATPase component (A1B345) and the enzymes malate dehydrogenase (A1B0K8) and succinate dehydrogenase (A1AZI5). Highly represented in anaerobiosis, but also detected at lower intensities under aerobic conditions (**Figure 4**), were the nitrous oxide reductase NosZ (A1B9T9), and a D-methionine transporter (A1BA28). High levels of synthesis under denitrifying conditions was also observed for several enzymes of the tricarboxylic acid (TCA) and glyoxylate cycles, such as aconitase (A1BAT6), isocitrate dehydrogenase (A1B6A1), fumarate hydratase class II (A1B3A9) and malate synthase (A1B9C3), and several enzymes that produce the key metabolite acetyl-CoA, including the acetyl-CoA synthetase (A1B9T3 and A1BAR9), the 3-hydroxyalcyl-CoA dehydrogenase (A1B611) and the butyryl-CoA:acetate CoA transferase (A1BB51).

A regulatory protein involved in denitrification up-regulated under nitrate-anaerobic conditions was NosR (A1B9U0), whereas the levels of the regulators FnrP (A1B9U4) and NirI (Q51699) remained unchanged independently of presence/absence of oxygen. On the other hand, proteins down-regulated under Den-N conditions, with higher levels during aerobiosis than in anaerobiosis, were the assimilatory nitrate reductase NasC (A1BAG9), the assimilatory nitrite reductase small (NasG) and large (NasB) subunits (A1BAH1, A1BAH2), the periplasmic nitrate reductase NapA (A1BB88), and the urease (A1B1B9) and the urease transporter (A1B989, A1B990, A1B992, A1B993), among others (**Figure 5**).

The high affinity *cbb*_3_ terminal oxidase was negatively affected by the presence of oxygen as revealed by the decreased level of two of its cytoplasmic components quantified in the proteomic analysis (**Figure 4**). However, none of the cytochrome *aa*_3_ oxidase components, which were expected to be expressed under aerobic conditions, could be detected in this study probably due to their large transmembrane regions.

**FIGURE 6:**
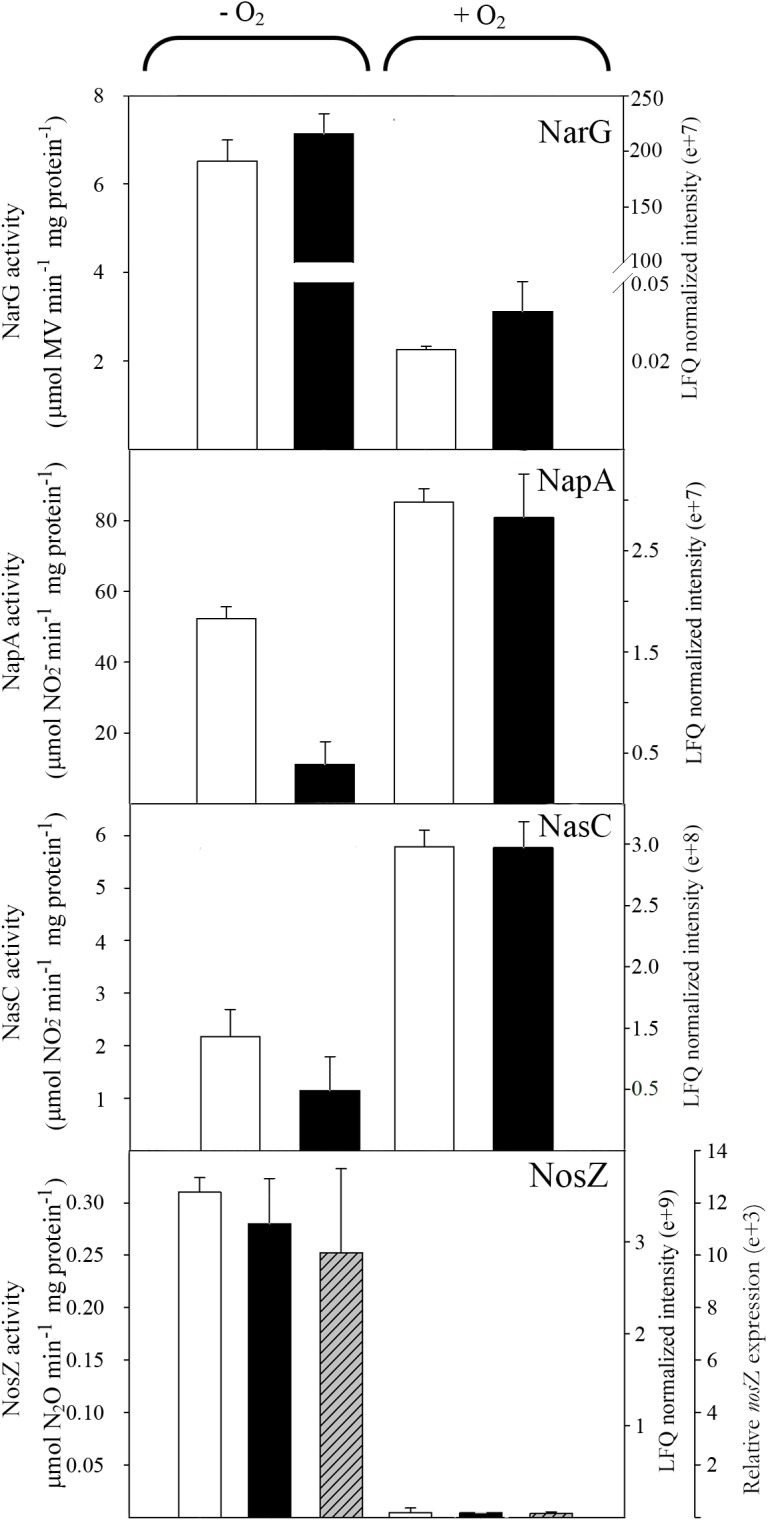
Effect of oxygen on nitrate and nitrous oxide reductases in *P. denitrificans* PD1222. Cells were cultured under anaerobic or aerobic conditions with nitrate as the sole nitrogen source. Normalized peptide abundance (black bars) and enzymatic activity (white bars) of membrane-bound respiratory (Nar), periplasmic (Nap) and assimilatory (Nas) nitrate reductases and nitrous oxide reductase (NosZ) are shown in cells grown with nitrate under anaerobic or aerobic conditions. The relative *nosZ* gene expression (dashed bars) has been also determined (*n* = 3, error bars correspond to data from 3 independent replicates).

In contrast to ammonium, oxygen showed a strong effect on the representation of reductases and other proteins involved in denitrification (**Figure 6**). Thus, the respiratory nitrate reductase Nar decreased in the presence of oxygen to half of the activity and protein intensity found in anaerobiosis. On the contrary, the assimilatory and periplasmic nitrate reductases, Nas and Nap, increased significantly under aerobic conditions (**Figure 6**). These results indicate that under anaerobic conditions the nitrate fate is mainly the denitrification pathway, whereas in aerobiosis nitrate assimilation is the major nitrate reduction pathway, although the periplasmic nitrate reductase may function as a redox balancing enzyme contributing to the production of extracellular nitrite through aerobic nitrate reduction (Richardson et al., 2001). The levels of gene expression, protein intensity and activity of the nitrous oxide reductase NosZ were much lower with oxygen than in anoxic conditions (**Figure 6**). Expression of *P. denitrificans* nitrous oxide reductase has been demonstrated to be regulated by oxygen and nitric oxide through FnrP and Nnr transcription regulators (Bergaust et al., 2012).

**FIGURE 7:**
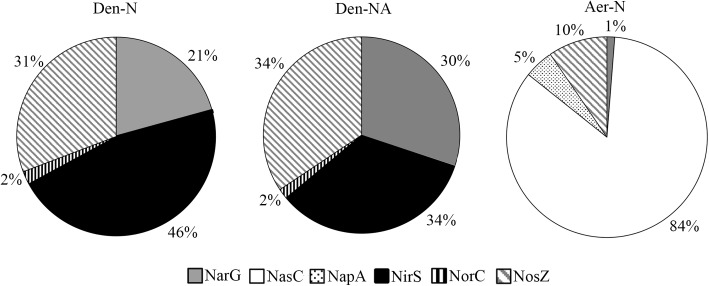
Effect of ammonium and oxygen on the normalized protein intensity of the denitrification enzymes. Data are shown as percentages in each growth condition. The sum of the peptide intensities of the represented proteins was considered the 100% and this value was used as reference to calculate specific % for each protein. Den-N, cells cultured with nitrate in anaerobiosis; Den-NA, cells cultured with nitrate plus ammonium in anaerobiosis; Aer-N, cells cultured with nitrate in aerobiosis.

Focusing on the denitrification and nitrate assimilation proteins, analysis of the peptide quantification (normalized peptide intensities) revealed that in Den-N conditions the cytochrome *cd*_1_-type nitrite reductase NirS was the most abundant enzyme, followed by the nitrous oxide reductase NosZ. The high abundance of NirS could be explained considering that nitrite is the most toxic soluble oxyanion described, or it may reflect the need to increase the amount of this enzyme compared to other denitrification enzymes to compensate for its reported catalytic inefficiency (Richter et al., 2002). Regarding the three nitrate reductases, the membrane-bound respiratory nitrate reductase (NarG) was the most abundant, whereas periplasmic (NapA) and assimilatory (NasC) nitrate reductases were detected at much lower levels (**Figure 7**). It was notable that NorC peptides were only detected at low levels, which may indicate some recalcitrance of this specific protein to LC-MS/MS analysis. Ammonium did not have a drastic effect on the peptide intensities of the main oxide-reductases involved in denitrification under anaerobic conditions (**Figure 7**). However, at early stages of growth it has been demonstrated that when ammonium is used as alternative N-source in the presence of nitrate (Den-NA), it exerts a negative effect on the denitrification process (**Table 1**). Normalized peptide intensities from cells grown aerobically with nitrate (Aer-N) revealed that the most abundant reductase was the assimilatory nitrate reductase Nas, whereas other reductases that are considered essential for denitrification decreased drastically (**Figures 6**, **7**). Accordingly, gasses involved in denitrification were not detected in cell cultures grown aerobically with nitrate, demonstrating that most of the nitrate is incorporated to organic nitrogen *via* the assimilatory pathway (**Table 1**). This result was also supported by the determination of the assimilatory nitrate reductase activity assayed in cytoplasmic fractions with NADH as electron donor (**Figure 6**). Also, the normalized intensities obtained in the proteomic study for the nitrous oxide reductase were the highest during denitrification, and this result was validated by determining NosZ enzymatic activity and also correlated with *nosZ* gene expression by qRT-PCR (**Figure 6**). This will contribute to maintaining low N_2_O emissions during denitrification under the experimental conditions tested here.

To complement the proteomic analyses, several intracellular metabolites were examined (**Table 2**). Concentrations of acetyl-CoA and oxaloacetate were higher in anaerobiosis (Den-N and Den-NA) than in aerobiosis (Aer-N), whereas intracellular ammonium concentration was slightly higher under nitrate-aerobic (Aer-N) than in nitrate-respiring anaerobic conditions (Den-N). Accumulation of acetyl-CoA and oxaloacetate under anaerobic conditions could be related to the increased intensities found in anaerobiosis, when compared to aerobiosis, of several enzymes like the acetyl-CoA synthetase (A1B9T3, A1BAR9), the 3-hydroxyalcyl-CoA dehydrogenase (A1B611) and the butyryl-CoA:acetate CoA transferase (A1BB51). Levels of oxaloacetate were also higher in anaerobiosis than in aerobiosis because peptide intensities of TCA enzymes like aconitase (A1BAT6), isocitrate dehydrogenase (A1B6A1) and fumarate hydratase (A1B3A9) were increased under anaerobic conditions (**Figure 4**).

**Table 2:** Intracellular metabolite determinations in *P. denitrificans* PD1222.

Sample^∗^	Acetyl-CoA (pM)	Oxaloacetate (μM)	Ammonium (mM)
Den-N	3.72 ± 0.43	27.33 ± 1.15	2.66 ± 0.34
Den-NA	3.57 ± 0.31	20.90 ± 0.93	11.37 ± 1.20
Aer-N	0.51 ± 0.02	9.64 ± 0.20	3.54 ± .052


**^∗^Den-N, cells grown in anaerobiosis with 30 mM nitrate; Den-NA, cells grown in anaerobiosis with 30 mM nitrate plus 10 mM ammonium; Aer-N, cells grown in aerobiosis with 10 mM nitrate. Samples were taken at early exponential (A_600_ ∼0.3) growth phase. Data are means ± DE of 2 independent biological replicates, and from each of these, 2 methodological samples (*n* = 4). Acetyl-CoA was determined by fluorescence, oxaloacetate was measured enzymatically with malate dehydrogenase and ammonium was determined spectrophotometrically as indicated in experimental procedures.**

## Conclusion

**FIGURE 8:**
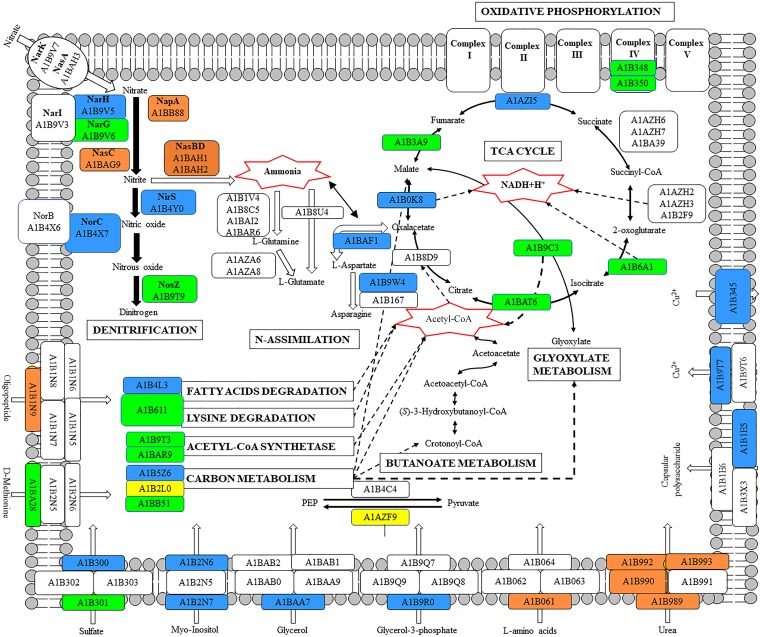
Global scheme of *P. denitrificans* PD1222 whole proteome under denitrifying conditions. Color code: blue, exclusive of denitrifying conditions; green, over-represented in denitrification; orange, down-represented in denitrification; yellow, exclusive of aerobiosis; white, not detected or without change when comparing different growth conditions.

This is the first study in which a global overview of the denitrification process has been described at a comprehensive quantitative proteome level, by using the soil denitrifier *P. denitrificans* PD1222 as a model organism. The proteomic approach by LC-MS/MS not only allowed identification and quantification of large number of proteins involved in denitrification, but also provided a holistic view of the effect of oxygen (an alternative electron acceptor) and ammonium (an alternative nitrogen source) on the denitrification process (**Figure 8**). Insights into the inter-relation of carbon and nitrogen metabolism were also forthcoming. In this sense, the low energetic yield obtained from the anaerobic reduction of nitrate to dinitrogen compared to the energy produced in aerobic respiration is compensated by the induction of enzymes that synthesize acetyl-CoA or belonging to the central metabolic carbon pathways like the TCA and the glyoxylate cycles.

## Experimental Procedures

### Bacterial Strain, Media and Growth Conditions

*Paracoccus denitrificans* PD1222 was routinely cultured under aerobic or anaerobic conditions at 30°C in a defined mineral salt medium (Harms et al., 1985). Under aerobic conditions, potassium nitrate (10 mM) was used as nitrogen source, and sodium succinate (30 mM) was the sole carbon source in media adjusted to pH 7.2. Aerobic cultures (25 ml) were shaken at 225 rpm. Under anaerobic conditions, 30 mM potassium nitrate was used as nitrogen source and electron acceptor in the presence or absence of 10 mM ammonium chloride as additional nitrogen source, and 30 mM succinate was used as carbon source in media at pH 7.2. In all cases, an aerobic overnight culture, prepared from a frozen stock in mineral salt medium supplemented with 10 mM ammonium chloride, was centrifuged and used as inoculum. Cell growth was followed by measuring the absorbance of cultures at 600 nm (A_600_) and cells were harvested at A_600_ ∼0.3 or A_600_ ∼0.9 as specified in the experiment. Spectinomycin was used as antibiotic at 25 μg ml^-1^ final concentration.

### Nitrate and Nitrous Oxide Reductase Assays

To determine periplasmic nitrate reductase (Nap), respiratory nitrate reductase (Nar) and assimilatory nitrate reductase (Nas) activities, *P. denitrificans* cells (35 ml culture) were harvested by centrifugation at 6,000 rpm at 4°C for 15 min and washed twice with 10 mM Tris-HCl buffer at pH 7.5. Cells were resuspended in 5 ml STE buffer (10 mM Tris-HCl pH 8.3, 3 mM EDTA and 171 g l^-1^ sucrose) and incubated with 2 mg lysozyme at 30°C for 20 min. After centrifugation at 10,000 rpm and 4°C for 15 min, the supernatant corresponding to the periplasmic fraction was stored on ice until used to determine Nap activity. The pellet with the spheroplasts was resuspended in 1 ml 100 mM Tris-HCl (pH 8) supplemented with DNase. Spheroplasts were sonicated (3 cycles of 20 s on/off pulsing) and unbroken spheroplasts were removed by centrifugation at 6,000 rpm at 4°C for 15 min. The membrane and cytoplasmic fractions were collected by ultracentrifugation at 45,000 rpm at 4°C for 45 min. Supernatant corresponding to cytoplasmic fraction was used to determine Nas activity and the membrane fraction was homogenized in 50 mM Tris-HCl (pH 8.0) to measure Nar activity.

Periplasmic nitrate reductase assay was performed with subcellular periplasmic fraction in 500 μl total volume of reaction mixture containing 50 μl sodium dithionite (prepared in 46 mM Tris-HCl, pH 7.5), 80 μM methyl viologen as non-physiological electron donor, 5 mM Tris-HCl (pH 7.5), 10 mM KNO_3_ and 200 μl of periplasmic extract. After 15 min of incubation at 30°C, reactions were stopped by vigorous shaking and 500 μl distilled water was added. Finally, nitrite production was measured as previously described (Snell and Snell, 1949). As negative controls, reaction mixtures were prepared as indicated above, but an initial vigorous shaking was applied to reoxidize methyl-viologen.

Respiratory nitrate reductase (Nar) was measured following the microtiter protocol previously described (Ridley et al., 2006a,b). In a 200 μl final volume, 170 μl of a mixture with 80 μM methyl viologen, 10 mM KNO_3_ and 5 mM Tris-HCl (pH 7.5) were incubated with 10 μl membrane fractions and 20 μl of a solution containing 46 mM sodium dithionite prepared in 0.5 M Tris-HCl, pH 7.5. Each biological replicate was measured in quadrupled, as well as two controls with an initial vigorous shaking. Other controls lacking either membrane fractions or KNO_3_ were also used. Absorbance at 600 nm was recorded at 1-min intervals over a 10-min periods incubating at 30°C throughout to measure the disappearance of electrons from methyl viologen. A best-fit slope of each curve was used to calculate the activity.

Assimilatory nitrate reductase (Nas) was assayed in cytoplasmic fractions by using NADH as electron donor as previously described (Gates et al., 2011). The assay was performed in 1 ml total volume containing 200 μl cytoplasmic fraction, 1 mM NADH, 100 mM Tris-HCl (pH 8.0), 10 mM KNO_3_ and distilled water. After 10 min incubation at 30°C, nitrite production was measured as described above. Negative controls were carried out in absence of the electron donor NADH.

N_2_O-reducing NosZ activity was measured *in vivo* by using 35 ml cell cultures that were washed twice in 10 mM Tris-HCl (pH 7.5) and resuspended in 3 ml 50 mM Tris-HCl (pH 7.5). Then were sealed in a 10-ml serum vial under helium atmosphere. The reaction was started after 10 min of adaptation at 30°C by adding 500 μl N_2_O. Samples from the headspace (100 μl) were analyzed by gas chromatography at 10 min intervals (Frunzke and Zumft, 1984) with a column SUPELCO CarboxenTM 1010. The concentration of NO, N_2_O and N_2_ was estimated by using a calibration plot previously elaborated.

Enzymic activities were assayed in three separated independent cultures. Protein concentration was estimated either in subcellular fractions (Bradford, 1976) or in whole cells by a modified method of the Lowry procedure (Shakir et al., 1994).

### *In Vivo* Gas Emissions and Extracellular Nitrate and Nitrite Determinations

*Paracoccus denitrificans* PD1222 was cultured (30 ml total volume) in sealed tubes with 15 ml of anaerobic atmosphere. When cells reached an absorbance at 600 nm (A_600_) of about 0.3 (early exponential growth phase) or about 0.9 (stationary growth phase), 1 ml from the headspace was analyzed by GC as described above. NO and N_2_O gasses were not detected (<1 μM). The concentration of nitrate was measured in the extracellular media (supernatants) by using a previously described method with sulfamic and perchloric acids (Cawse, 1967). Nitrite and ammonium in the extracellular media were measured by using the Snell and Snell (1949) and the Solorzano (1969) methods, respectively.

### Intracellular Metabolite Determinations

Total extracts were deproteinized by using 10% trichloroacetic acid. After 5 min of incubation on ice, samples were centrifuged at 14,000 rpm and supernatants were recovered for further analysis. Intracellular ammonium was determined by using a method previously described (Solorzano, 1969). Acetyl-CoA was measured from deproteinized total extracts by using the Acetyl-Coenzyme A Assay Kit (Sigma) according with the instructions of the manufacturer. Oxaloacetate measurement was enzymatically assayed with malate dehydrogenase in a spectrophotometer following the disappearance of NADH at 340 nm. The concentration of analytes was estimated by using calibration plots previously elaborated with stock solutions of the analytes detected.

### Quantification of *nosZ* Gene Expression

*Paracoccus denitrificans* PD1222 cells were harvested at the early exponential growth (A_600_ ∼0.3) and then washed in TEG buffer containing 25 mM Tris-HCl (pH 8.0) with 1% glucose and 10 mM EDTA. RNA isolations were performed following the Qiagen RNA extraction kit (RNeasy midi kit). DNase incubation was carried out in the column with RNase-free DNase set (Qiagen) and an additional post-column treatment was required with DNase I (Ambion). The concentration and purity of the RNA samples were measured by using a ND1000 spectrophotometer (Nanodrop Technologies). Synthesis of total cDNA was achieved in 20 μl final volume containing 500 ng RNA, 0.7 mM dNTPs, 200 U SuperScript II Reverse Transcriptase (Invitrogen), and 3.75 mM random hexamers (Applied Biosystems). Samples were initially heated at 65°C for 5 min and then incubated at 42°C for 50 min, followed by incubation at 70°C for 15 min. The cDNA was purified using Favorprep Gel/PCR purification kit (Favorgen) and the concentration was measured using a Nanodrop. The iQ5 Multicolor Real-Time PCR Detection System (Bio-Rad) was used in a 25 μl reaction (final volume), containing 2 μl of diluted cDNA (12.5, 2.5, and 0.5 ng), 0.2 μM of each primer nosZQ1: 5′-TCTTCTGCAATGGCGAGGACGAGAC-3′ and nosZQ2: 5′-CGAGCACCTGCCAGGCGACC-3′ and 12.5 μl of iQ SYBR Green Supermix (Bio-Rad). Target cDNAs and reference samples were amplified three times in separate PCR reactions. Samples were initially denatured by heating at 95°C for 3 min, followed by 40 cycles of amplification (95°C, 30 s; test annealing temperature, 60°C, 30 s; elongation and signal acquisition, 72°C, 30 s). For relative quantification of the fluorescence values, a calibration curve was made using dilution series from 80 to 0.008 ng of *P. denitrificans* PD1222 genomic DNA sample. Data were normalized by using the *dnaN* gene as housekeeping with dnaN-1F′: 5′-CATGTCGTGGGTCAGCATAC-3′ and dnaN-1R′: 5′-CTCGCGACCATGCATATAGA-3′ primers.

### Proteomic Analysis

*Paracoccus denitrificans* cells were grown both under anaerobic or aerobic conditions with different nitrogen sources as described above. When cultures reached A_600_ ∼0.3, cells were harvested by centrifugation at 12,000 rpm for 15 min and kept at -80°C until use. Samples for LC-MS/MS proteomic analysis were prepared by resuspension of frozen cells in 50 mM Tris-HCl buffer (pH 8.0) containing 4% CHAPS and 8 M urea, then cells were broken by cavitation with ultrasound (3 pulses of 90 W, for 5 min each pulse). Proteins were cleaned with the 2-D Clean-UP Kit (GE Healthcare, Little Chalfont, United Kingdom) and after precipitation were resuspended in a solution containing 6 M urea. Protein concentration was estimated as described previously (Bradford, 1976) and sample concentration ranged from 2 to 4 μg μl^-1^. Samples were digested with trypsin overnight at 37°C without agitation. All analyses were performed at the Research Support Central Service (SCAI), University of Cordoba, with a Dionex Ultimate 3000 nano UHPLC system (Thermo Fisher Scientific, San Jose, CA, United States) connected to a mass spectrometer Orbitrap Fusion (Thermo Fisher Scientific, San Jose, CA, United States) equipped with nanoelectrospray ionization interface. The separation column was Acclaim Pepmap C18, 500 mm × 0.075 mm, 2 μm pore size (Thermo Fisher Scientific, San Jose, CA, United States). For trapping of the digest, it was used a 5 mm × 0.3 mm precolumn Acclaim Pepmap C18 (Thermo Fisher Scientific, San Jose, CA, United States). The samples, containing 0.2 μg μl^-1^, were trapped at 10 μl min^-1^ flow rate, for 5 min, with 2% acetonitrile/0.05% trifluoroacetic acid. After the trapping column was switched on-line with the separation column and the gradient was started. Peptides were eluted with a 60-min gradient 5–40% acetonitrile/0.1% formic acid solution at a flow rate of 300 nl min^-1^. Survey scans of peptide precursors from 400 to 1500 m/z were performed at 120 K resolution (at 200 m/z) with a 5 × 10^5^ ion count target. Tandem MS was performed by isolation at 1.6 Da with the quadrupole, CID fragmentation with normalized collision energy of 35, and rapid scan MS analysis in the ion trap. The AGC ion count target was set to 2 × 10^3^ and the max injection time was 75 ms. Only those precursors with charge state 2–5 were sampled for MS2. The dynamic exclusion duration was set to 15 s with 10 ppm tolerance around the selected precursor and its isotopes. Monoisotopic precursor selection was turned on. The instrument was run in top speed mode with 3 s cycles, meaning the instrument would continuously perform MS2 events until the list of non-excluded precursors diminishes to zero or 3 s, whichever is shorter. Charge state deconvolution and deisotoping were not performed. MS2 spectra were searched using MaxQuant software v. 1.5.7.4 (Cox and Mann, 2008). MS2 spectra were searched with Andromeda engines, respectively, against a database of *P. denitrificans* PD1222 (deposited in the Uniprot database under the accession number UP000000361). Peptides generated from a tryptic digestion were searched by using the following parameters: up to one missed cleavages, carbamidomethylation of cysteines as fixed modifications, and oxidation of methionine as variable modifications. Precursor mass tolerance was 10 ppm and product ions were searched at 0.6 Da tolerances. Peptides were validated by filtering according with 1% FDR *q-*value. A target-decoy search strategy was applied, which integrates multiple peptide parameters such as length, charge, number of modifications and the identification score into a single quality that acts as the statistical evidence on the quality of each single peptide spectrum match. Peptide identifications were carried out by using MaxQuant software, in a MaxLFQ label-free quantification method (Cox and Mann, 2008; Cox et al., 2014). In the MaxLFQ label-free quantification method a retention time alignment and identification transfer protocol (“match-between-runs” feature in MaxQuant) was applied. Proteins identified from only one peptide were not considered in this analysis.

Identified peptides were grouped into proteins according with the law of parsimony and filtered to 1% FDR. Differentially expressed proteins analysis was carried out with the freely available software Perseus (version 1.5.6.0)^1^. Peak intensities across the whole set of quantitative data for all the peptides in the samples were imported from the LFQ intensities of proteins from the MaxQuant analysis and normalized according with the median. LFQ normalized intensity values were transformed to logarithmic scale with base two. Proteins identified in only one replicate were discarded. Proteins identified in at least three replicates per condition were used after replacing missing values (imputated) with the value of the lowest intensity. The protein quantification and calculation of statistical significance were carried out using two-way Student-*t* test and error correction (*p-*value < 0.05) with the Benjamini–Hochberg method. For further visualization, and to obtain *p-*value, a heat-map and a Principle Component Analysis (PCA) were performed. Relative protein quantification after LC-MS/MS procedure was performed by using the MaxQuant software (Cox and Mann, 2008). Results were then filtered, and a *p*-value ≤ 0.05 and a fold change ≥ 2 were considered. The mass spectrometry data have been deposited to the ProteomeXchange Consortium^2^ via the PRIDE partner repository (Vizcaino et al., 2013) with the dataset identifier PDX006821.

BLAST2GO bioinformatics platform (Conesa et al., 2005) was used to assign GO terms to proteins identified by MS/MS. BLASTP search was conducted against the Bacteria protein database at Uniprot site and the recovery of up to 20 hits (*e*-value < 10^-5^) was allowed. Interpro search, PSORTb subcellular prediction search, and subsequent mapping and annotation (*e*-value hit filter < 10^-6^) steps were conducted using default parameters. Protein distribution within GO terms was retrieved from GO level 3. GO analysis were performed using the web application ComparativeGO (Fruzangohar et al., 2013). Integration of final proteomic data were performed by using the tool KEGG Mapper. Interaction maps were assembled by using STRING and functionally grouped network with ClueGO from Cytoscape, respectively.

## Author Contributions

Proteomic analysis was performed by AO-A and CF-A. qRT-PCR analysis was performed by VL-A. Gases determination and enzymic activities were carried out by JH-C, AO-A, and FU. Conceptualization, administration, and funding of the project were supplied by MR, CM-V, and DR. The manuscript was written by MR.

## Conflict of Interest Statement

The authors declare that the research was conducted in the absence of any commercial or financial relationships that could be construed as a potential conflict of interest.

## Footnotes

1 http://www.coxdocs.org/doku.php?id=perseus:start

2 http://proteomecentral.proteomexchange.org

## References

[B1] Baker S. C. Ferguson S. J. Ludwig B. Page M. D. Richter O. M. H. van Spanning R. J. M. (1998). Molecular genetics of the genus *Paracoccus*: metabolically versatile bacteria with bioenergetic flexibility. *Microbiol. Mol. Biol. Rev.* 62 1046–1078. 9841665 10.1128/mmbr.62.4.1046-1078.1998 PMC98939

[B2] Bakken L. Bergaust L. Liu B. Frostegård Å. (2012). Regulation of denitrification at the cellular level: a clue to the understanding of N_2_O emissions from soils. *Philos. Trans. R. Soc. Lond. B Biol. Sci.* 367 1226–1234. 10.1098/rstb.2011.0321 22451108 PMC3306626

[B3] Bali S. Lawrence A. D. Lobo S. A. Saraiva L. M. Golding B. T. Palmer D. J. (2011). Molecular hijacking of siroheme for the synthesis of heme and d1 heme. *Proc. Natl. Acad. Sci. U.S.A.* 108 18260–18265. 10.1073/pnas.1108228108 21969545 PMC3215036

[B4] Bergaust L. Mao Y. Bakken L. Frostegård A. (2010). Denitrification response patterns during the transition to anoxic respiration and posttranscriptional effects of suboptimal pH on nitrogen oxide reductase in *Paracoccus denitrificans*. *Appl. Environ. Microbiol.* 76 6387–6396. 10.1128/AEM.00608-10 20709842 PMC2950438

[B5] Bergaust L. Shapleigh J. Frostegård Å Bakken L. (2008). Transcription and activities of NOx reductases in *Agrobacterium tumefaciens*: the influence of nitrate, nitrite and oxygen availability. *Environ. Microbiol.* 10 3070–3081. 10.1111/j.1462-2920.2007.01557.x 18312398

[B6] Bergaust L. van Spanning R. Frostegård Å. Bakken L. (2012). Expression of nitrous oxide reductase in *Paracoccus denitrificans* is regulated by oxygen and nitric oxide through FnrP and NNR. *Microbiology* 158 826–834. 10.1099/mic.0.054148-0 22174385 PMC3541799

[B7] Bradford M. M. (1976). A rapid and sensitive method for the quantification of microgram quantities of protein utilizing the principle of protein dye binding. *Anal. Biochem.* 72 248–256. 10.1016/0003-2697(76)90527-3 942051

[B8] Cawse P. A. (1967). The determination of nitrate in soil solutions by ultraviolet spectrophotometry. *Analyst* 92 311–315. 10.1016/j.saa.2013.01.049 23466321

[B9] Conesa A. Götz S. García-Gómez J. M. Terol J. Talón M. Robles M. (2005). Blast2GO: a universal tool for annotation, visualization and analysis in functional genomics research. *Bioinformatics* 21 3674–3676. 10.1093/bioinformatics/bti610 16081474

[B10] Cox J. Hein M. Y. Luber C. A. Paron I. Nagaraj N. Mann M. (2014). Accurate proteome-wide label-free quantification by delayed normalization and maximal peptide ratio extraction, termed MaxLFQ. *Mol. Cell. Proteomics* 13 2513–2526. 10.1074/mcp.M113.031591 24942700 PMC4159666

[B11] Cox J. Mann M. (2008). MaxQuant enables high peptide identification rates, individualized ppb-range mass accuracies and proteome-wide protein quantification. *Nat. Biotechnol.* 26 1367–1372. 10.1038/nbt.1511 19029910

[B12] Crack J. C. Hutchings M. I. Thomson A. J. Le Brun N. E. (2016). Biochemical properties of *Paracoccus denitrificans* FnrP: reactions with molecular oxygen and nitric oxide. *J. Biol. Inorg. Chem.* 21 71–82. 10.1007/s00775-015-1326-7 26790880 PMC4771820

[B13] Felgate H. Giannopoulos G. Sullivan M. J. Gates A. J. Clarke T. A. Baggs E. (2012). The impact of copper, nitrate and carbon status on the emission of nitrous oxide by two species of bacteria with biochemically distinct denitrification pathways. *Environ. Microbiol.* 14 1788–1800. 10.1111/j.1462-2920.2012.02789.x 22642644

[B14] Frunzke K. Zumft W. G. (1984). Rapid, single sample analysis of H_2_, O_2_, N_2_, NO, CO, N_2_O and CO_2_ by isothermal gas chromatography: applications to the study of bacterial denitrification. *J. Chromatogr.* 299 477–483. 10.1016/S0021-9673(01)97868-9

[B15] Fruzangohar M. Ebrahimie E. Ogunniyi A. D. Mahdi L. K. Paton J. C. Adelson D. L. (2013). Comparative GO: a web application for comparative gene ontology and gene ontology-based gene selection in bacteria. *PLoS One* 8:e58759. 10.1371/journal.pone.0058759 23536820 PMC3594149

[B16] Gates A. J. Luque-Almagro V. M. Goddard A. D. Ferguson S. J. Roldán M. D. Richardson D. J. (2011). A composite biochemical system for bacterial nitrate and nitrite assimilation as exemplified by *Paracoccus denitrificans*. *Biochem. J.* 435 743–753. 10.1042/BJ20101920 21348864

[B17] Giannopoulos G. Sullivan M. J. Hartop K. R. Rowley G. Gates A. J. Watmough N. J. (2017). Tuning the modular *Paracoccus denitrificans* respirome to adapt from aerobic respiration to anaerobic denitrification. *Environ. Microbiol.* 19 4953–4964. 10.1111/1462-2920.13974 29076595

[B18] Harms N. de Vries G. E. Maurer K. Veltkamp E. Stouthamer A. H. (1985). Isolation and characterization of *Paracoccus denitrificans* mutants with defects in the metabolism of one-carbon compounds. *J. Bacteriol.* 164 1064–1070. 3905763 10.1128/jb.164.3.1064-1070.1985 PMC219298

[B19] Hu W. Chen D. He J.-Z. (2015). Microbial regulation of terrestrial nitrous oxide formation: understanding the biological pathways for prediction of emission rates. *FEMS Microbiol. Rev.* 39 729–749. 10.1093/femsre/fuv021 25934121

[B20] Luque-Almagro V. M. Gates A. J. Moreno-Vivián C. Ferguson S. J. Richardson D. J. Roldán M. D. (2011). Bacterial nitrate assimilation; gene distribution and regulation. *Biochem. Soc. Trans.* 39 1838–1843. 10.1042/BST20110688 22103536

[B21] Luque-Almagro V. M. Lyall V. J. Ferguson S. J. Roldán M. D. Richardson D. J. Gates A. J. (2013). Nitrogen oxyanion-dependent dissociation of a two-component complex that regulates bacterial nitrate assimilation. *J. Biol. Chem.* 288 29692–29702. 10.1074/jbc.M113.459032 24005668 PMC3795266

[B22] Luque-Almagro V. M. Manso I. Sullivan M. J. Rowley G. Ferguson S. J. Moreno-Vivián C. (2017). Transcriptional and translational adaptation to aerobic nitrate anabolism in the denitrifier *Paracoccus denitrificans*. *Biochem. J.* 474 1769–1787. 10.1042/BCJ20170115 28385879 PMC5424462

[B23] Patureau D. Zumstein E. Delgenes J. P. Moletta R. (2000). Aerobic denitrifiers isolated from diverse natural and managed ecosystems. *Microb. Ecol.* 39 145–152. 10.1007/s002480000009 10833227

[B24] Philippot L. Hallin S. Schloter M. (2007). Ecology of denitrifying prokaryotes in agricultural soil. *Adv. Agron.* 96 249–305. 10.1016/S0065-2113(07)96003-4 14711656

[B25] Richardson D. J. (2000). Bacterial respiration: a flexible process for a changing environment. *Microbiology* 146 551–571. 10.1099/00221287-146-3-551 10746759

[B26] Richardson D. J. Berks B. C. Russell D. A. Spiro S. Taylor C. J. (2001). Functional, biochemical and genetic diversity of prokaryotic nitrate reductases. *Cell. Mol. Life Sci.* 58 165–178. 10.1007/PL00000845 11289299 PMC11146511

[B27] Richardson D. J. Felgate H. Watmough N. Thomson A. Baggs E. (2009). Mitigating release of the potent greenhouse gas N_2_O from the nitrogen cycle – could enzymic regulation hold the key? *Trends Biotechnol.* 27 388–397. 10.1016/j.tibtech.2009.03.009 19497629

[B28] Richter C. D. Allen J. W. A. Higham C. W. Koppenhöfer A. Watmough N. J. Ferguson S. J. (2002). Cytochrome cd1, reductive activation and kinetic analysis of a multifunctional respiratory enzyme. *J. Biol. Chem.* 277 3093–3100. 10.1074/jbc.M108944200 11709555

[B29] Ridley H. Watts C. A. Richardson D. J. Butler C. S. (2006a). Development of a viologen-based microtiter plate assay for the analysis of oxyanion activity: application to the membrane-bound selenate reductase from *Enterobacter cloacae* SLD1a-1. *Anal. Biochem.* 358 289–294. 17027906 10.1016/j.ab.2006.08.028

[B30] Ridley H. Watts C. A. Richardson D. J. Butler C. S. (2006b). Resolution of distinct membrane-bound enzymes from *Enterobacter cloacae* SLD1a-1 that are responsible for selective reduction of nitrate and selenate oxyanions. *Appl. Envirom. Microbiol.* 72 5173–5180. 16885262 10.1128/AEM.00568-06 PMC1538730

[B31] Sanders D. A. Gillece-Castro B. L. Burlingame A. L. Koshland D. E. Jr. (1992). Phosphorylation site of NtrC, a protein phosphatase whose covalent intermediate activates transcription. *J. Bacteriol.* 174 5117–5122. 10.1128/jb.174.15.5117-5122.1992 1321122 PMC206329

[B32] Saunders N. F. W. Hornberg J. J. Reijnders W. N. Westerhoff H. V. de Vries S. van Spanning R. J. (2000). The NosX and NirX proteins of *Paracoccus denitrificans* are functional homologues: their role in maturation of nitrous oxide reductase. *J. Bacteriol.* 182 5211–5217. 10.1128/JB.182.18.5211-5217.2000 10960107 PMC94671

[B33] Saunders N. F. W. Houben E. N. Koefoed S. de Weert S. Reijnders W. N. Westerhoff H. V. (1999). Transcription regulation of the nir gene cluster encoding nitrite reductase of *Paracoccus denitrificans* involves NNR and NirI, a novel type of membrane protein. *Mol. Microbiol.* 34 24–36. 10.1046/j.1365-2958.1999.01563.x 10540283

[B34] Shakir F. K. Audilet D. Drake A. J. Shakir K. M. (1994). A rapid protein determination by modification of the Lowry procedure. *Anal. Biochem.* 216 232–233. 10.1006/abio.1994.1031 8135358

[B35] Shcherbak I. Millara N. Robertson G. P. (2014). Global metaanalysis of the nonlinear response of soil nitrous oxide (N_2_O) emissions to fertilizer nitrogen. *Proc. Nat. Acad. Sci. U.S.A.* 111 9199–9204. 10.1073/pnas.1322434111 24927583 PMC4078848

[B36] Snell F. D. Snell C. T. (1949). *Colorimetric Methods of Analysis.* New York, NY: Van Nostrand, 802–807.

[B37] Solorzano L. (1969). Determination of ammonia in natural waters by the phenol hypochlorite method. *Limnol. Oceanogr.* 14 799–801.

[B38] Speers A. E. Wu C. C. (2007). Proteomics of integral membrane proteins-theory and application. *Chem. Rev.* 107 3687–3714. 10.1021/cr068286z 17683161

[B39] Spiro S. (2012). Nitrous oxide production and consumption: regulation of gene expression by gas-sensitive transcription factors. *Philos. Trans. R. Soc. Lond. B. Biol. Sci.* 367 1213–1225. 10.1098/rstb.2011.0309 22451107 PMC3306620

[B40] Sullivan M. J. Gates A. J. Appia-Ayme C. Rowley G. Richardson D. J. (2013). Copper control of bacterial nitrous oxide emission and its impact on vitamin B12-dependent metabolism. *Proc. Nat. Acad. Sci. U.S.A.* 110 19926–19931. 10.1073/pnas.1314529110 24248380 PMC3856849

[B41] van Spanning R. J. Houben E. Reijnders W. N. Spiro S. Westerhoff H. V. Saunders N. (1999). Nitric oxide is a signal for NNR-mediated transcription activation in *Paracoccus denitrificans*. *J. Bacteriol.* 181 4129–4132. 10383987 10.1128/jb.181.13.4129-4132.1999 PMC93909

[B42] van Spanning R. J. Richardson D. J. Ferguson S. J. (2007). “Introduction to the biochemistry and molecular biology of denitrification,” in *The Biology of the Nitrogen Cycle*, eds Bothe H. Ferguson S. J. Newton W. E. (Amsterdam: Elselvier), 3–21.

[B43] Vizcaino J. A. Cote R. G. Csordas A. Dianes J. A. Fabregat A. Foster J. M. (2013). The proteomics identifications (PRIDE) database and associated tools: status in 2013. *Nucleic Acids Res.* 41(Database issue), D1063–D1069. 10.1093/nar/gks1262 23203882 PMC3531176

[B44] Wood N. Alizadeh T. Bennett S. Pearce J. Ferguson S. J. Richardson D. J. (2001). Maximal expression of membrane-bound nitrate reductase in *Paracoccus* is induced by nitrate via a third FNR-like regulator named NarR. *J. Bacteriol.* 183 3606–3613. 10.1128/JB.183.12.3606-3613.2001 11371524 PMC95237

[B45] Wrage N. Velthoft G. L. van Beisichem M. L. Oenema O. (2001). Role of nitrifier denitrification in the production of nitrous oxide. *Soil Biol. Biochem.* 33 1723–1732. 10.1016/S0038-0717(01)00096-7

[B46] Zumft W. G. (1997). Cell biology and molecular basis of denitrification. *Microbiol. Mol. Biol. Rev.* 61 533–616. 9409151 10.1128/mmbr.61.4.533-616.1997 PMC232623

