# Supplementary material for: Exploring the Denitrification Proteome of Paracoccus denitrificans PD1222
Source: Front Microbiol. 2018 May 29;9:1137. doi: 10.3389/fmicb.2018.01137 (PMC5987163; doi:10.3389/fmicb.2018.01137)
Supplement: Supplementary file 1 [file Table_1.PDF]

Table S1. Proteins identified under anaerobic conditions on *P. denitrificans* PD1222

Protein[1]/Gene[2]	R1	R2	R3	R4	Protein names	Location[3]	GO[4]
A1B017/Pden_0749	65	71	71	67	DNA-directed RNA polymerase subunit beta	Cytoplasmic	6,1,8,4,7
A1B9V6/Pden_4236	63	78	79	75	Respiratory nitrate reductase alpha subunit (NarI)	Membrane	6,4,7,12,14
A1B877/Pden_3654	61	58	67	62	60 kDa chaperonin (GroEL protein, Cpn60)	Cytoplasmic	25
A1B015/Pden_0747	58	65	90	79	DNA-directed RNA polymerase subunit beta	Cytoplasmic	6,1,8,4,7
A1B002/Pden_0734	53	55	59	57	Elongation factor Tu (EF-Tu)	Cytoplasmic	0
A1B4Z0/Pden_2497	50	49	58	57	Catalase-peroxidase (CP) (Peroxidase/catalase)	Cytoplasmic	11,4,12,14,2
A1B023/Pden_0755	50	56	61	64	Elongation factor G (EF-G)	Cytoplasmic	0
A1B2C5/Pden_1569	49	52	55	55	Extracellular solute-binding protein, family 5	Periplasmic	12,5,13
A1B1N9/Pden_1328	48	52	56	55	Extracellular solute-binding protein, family 5	Periplasmic	12,5,13
A1AZA6/Pden_0488	47	50	55	56	Glutamate synthase (NADPH) large subunit	Unknown	8,1,4,7,12,14
A1B8K8/Pden_3786	44	48	42	43	30S ribosomal protein S1	Cytoplasmic	6,1,8,4,7
A1AZH7/Pden_0559	44	46	51	44	Succinate--CoA ligase	Cytoplasmic	8,4,7,12,14
A1BAH2/Pden_4452	43	43	39	40	Assimilatory nitrite reductase large subunit	Cytoplasmic	6,4,7,12,14
A1B5P9/Pden_2759	43	49	51	46	Polyribonucleotide nucleotidyltransferase (Polynucleotide phosphorylase) (PNPase)	Cytoplasmic	6,8,4,7,2
A1B587/Pden_2594	42	40	45	40	Translation initiation factor IF-2	Unknown	0
A1AZH3/Pden_0555	42	47	48	48	2-oxoglutarate dehydrogenase E1 component	Cytoplasmic	8,4,7,12,14
Q51700/Pden_2487	41	41	46	45	Nitrite reductase (cytochrome cd_1)	Periplasmic	0
A1B8N8/Pden_3816	41	43	48	49	ATP synthase subunit alpha	Cytoplasmic	7,4,12,14,5,13
A1BAT6/Pden_4567	39	45	48	48	Aconitase	Cytoplasmic	8,4,7,12,14
A1AZG9/Pden_0551	37	35	32	32	Dihydrolipoyl dehydrogenase	Cytoplasmic	12,7,14,9,2,10
A1AZL6/Pden_0598	36	41	46	49	Alanine--tRNA ligase (Alanyl-tRNA synthetase)	Cytoplasmic	8,6,4,7,12,14
A1B6A1/Pden_2961	34	35	36	39	Isocitrate dehydrogenase [NADP]	Cytoplasmic	8,4,7,12,14
A1B9T9/Pden_4219	34	38	46	46	Nitrous-oxide reductase (N_2O reductase, NosZ)	Periplasmic	0
A1B4E9/Pden_2302	32	33	40	32	Chaperone protein DnaK (HSP70)	Cytoplasmic	25
A1AZI7/Pden_0569	32	36	38	37	Succinate dehydrogenase flavoprotein subunit	Membrane	8,4,7,12,14
A1BAI2/Pden_4462	32	38	41	42	Glutamine synthetase	Cytoplasmic	8,1,4,7,12,14
A1B082/Pden_0814	31	32	35	33	D-3-phosphoglycerate dehydrogenase	Cytoplasmic	8,1,4,7,12,14
A1B971/Pden_3999	31	30	39	37	Protein assembly factor (BamA)	OuterMembrane	3,12,5
A1B1I6/Pden_1275	31	36	37	34	Transketolase	Cytoplasmic	0
A1BA21/Pden_4301	30	32	31	37	Aconitate hydratase (Aconitase)	Cytoplasmic	0
A1B9V5/Pden_4235	30	34	38	38	Respiratory nitrate reductase beta subunit (NarH)	Membrane	6,4,7,12,14
A1B9L4/Pden_4144	30	40	42	38	Pyruvate carboxylase	Cytoplasmic	8,1,4,7,12,14
A1B8P0/Pden_3818	29	29	36	32	ATP synthase subunit beta	Cytoplasmic	7,4,12,14,5,13
A1BAI5/Pden_4465	29	33	30	35	Glyceraldehyde-3-phosphate dehydrogenase	Cytoplasmic	8,7,14
A1B380/Pden_1879	29	34	37	38	Carbamoyl-phosphate synthase large chain	Unknown	8,1,4,7,12,14
A1B2M4/Pden_1671	28	25	28	28	Extracellular solute-binding protein, family 5	Periplasmic	12,5,13
A1B3M4/Pden_2026	28	28	31	27	Acetyl-CoA acetyltransferase	Cytoplasmic	0
A1AZM9/Pden_0611	27	23	28	27	Dihydrolipoyl dehydrogenase	Cytoplasmic	12,7,14,9,2,10
A1B0F4/Pden_0887	27	23	31	28	Trigger factor (TF) (PPIase)	Cytoplasmic	25,12,5,23
A1B5A3/Pden_2610	27	28	27	28	TonB-dependent receptor	OuterMembrane	5
A1B489/Pden_2241	27	28	28	31	NADH-quinone oxidoreductase	Cytoplasmic	4,12,14
A1B4H8/Pden_2331	27	31	27	33	Valine--tRNA ligase (Valyl-tRNA synthetase)	Cytoplasmic	8,6,4,7,12,14
A1B8E8/Pden_3725	27	31	31	31	Elongation factor Ts (EF-Ts)	Cytoplasmic	0

A1BA23/Pden_4303	27	31	31	32	30S ribosomal protein S4	Cytoplasmic	6,1,8,4,7
A1AY66/Pden_0093	26	27	26	29	Polysaccharide biosynthesis protein CapD	Cytoplasmic	0
A1B3C0/Pden_1920	25	24	26	22	Fructose-bisphosphate aldolase	Cytoplasmic	0
A1B3P4/Pden_2046	25	28	26	23	TonB-dependent receptor	OuterMembrane	5
A1AZL0/Pden_0592	25	26	26	27	Inosine-5'-monophosphate dehydrogenase	Cytoplasmic	8,1,4,7,12,14
A1B5H5/Pden_2682	24	19	22	22	Adenosylhomocysteinase (S-adenosyl-L-homocysteine hydrolase) (AdoHcyase)	Cytoplasmic	4,12,14
A1B5R5/Pden_2775	24	22	23	23	Aspartate-semialdehyde dehydrogenase	Cytoplasmic	8,1,4,7,12,14
A1AZP8/Pden_0630	24	24	22	25	Aromatic amino acid aminotransferase	Cytoplasmic	8,1,4,7,12,14
A1B1N2/Pden_1321	24	22	28	22	Lysine--tRNA ligase (Lysyl-tRNA synthetase)	Cytoplasmic	8,6,4,7,12,14
A1AZN7/Pden_0619	24	23	26	23	Glutamine--fructose-6-phosphate aminotransferase	Cytoplasmic	8,1,4,7,12,14
A1AZH2/Pden_0554	24	29	28	25	Dihydrolipoyllysine-residue succinyltransferase component of 2-oxoglutarate dehydrogenase	Cytoplasmic	4,8,7,12,14,2
A1AXX5/Pden_0002	24	27	29	27	Transcription termination factor Rho (ATP-dependent helicase Rho)	Cytoplasmic	1,6,8,4,7,9
A1B5Z2/Pden_2852	24	30	34	32	Phosphoenolpyruvate carboxykinase (ATP) (PCK) (PEP carboxykinase) (PEPCK)	Cytoplasmic	1,8,7,14
A1B592/Pden_2599	24	38	34	41	Protein translocase subunit (SecA)	Cytoplasmic	12,5,13,23
A1B012/Pden_0744	23	21	24	26	50S ribosomal protein L1	Cytoplasmic	1,6,8,4,7,9
A1B0H0/Pden_0903	23	25	28	25	Probable cytosol aminopeptidase	Cytoplasmic	0
A1B0I7/Pden_0920	23	27	28	25	Serine hydroxymethyltransferase	Cytoplasmic	8,1,4,7,12,14
A1B6A5/Pden_2965	23	22	31	30	Ribonuclease E (RNase E)	Cytoplasmic	6,8,4,7,2
A1B2W3/Pden_1760	22	24	26	23	Ketol-acid reductoisomerase (NADP(+))	Cytoplasmic	8,1,4,7,12,14
A1B5Q3/Pden_2763	22	23	24	27	Outer membrane porin	OuterMembrane	0
A1B3B8/Pden_1918	21	21	22	21	Phosphoglycerate kinase	Cytoplasmic	4,8,7,12,14,2
A1B028/Pden_0760	21	20	24	21	50S ribosomal protein L4	Cytoplasmic	6,1,8,4,7
A1B3K6/Pden_2008	21	22	26	19	ABC transporter related protein	Cytoplasmic	0
A1B590/Pden_2597	21	26	24	19	Arginine biosynthesis protein (ArgJ)	Cytoplasmic	8,1,4,7,12,14
A1B431/Pden_2183	21	23	26	21	Aspartate--tRNA(Asp/Asn) ligase	Cytoplasmic	8,6,4,7,12,14
A1B9J9/Pden_4129	21	24	24	23	Two component, sigma54 specific, transcriptional regulator, Fis family	Cytoplasmic	1,6,8,4,7,9
A1B4B0/Pden_2262	21	22	28	23	Phenylalanine--tRNA ligase beta subunit	Cytoplasmic	8,6,4,7,12,14
A1B472/Pden_2224	21	23	26	26	Malate dehydrogenase	Cytoplasmic	4,7,12,14
A1B9D2/Pden_4060	21	23	28	26	Enolase	Cytoplasmic	4,8,7,12,14,2
A1AZH9/Pden_0561	20	20	20	20	Malate dehydrogenase	Cytoplasmic	8,4,7,12,14
A1B8E9/Pden_3726	20	18	22	21	30S ribosomal protein S2	Cytoplasmic	6,1,8,4,7
A1B2B5/Pden_1559	20	20	21	21	Polysaccharide export protein	Unknown	0
A1B585/Pden_2592	20	19	20	24	Transcription termination/antitermination protein (NusA)	Cytoplasmic	1,6,8,4,7,9
A1B513/Pden_2520	20	25	20	23	3-isopropylmalate dehydratase large subunit	Cytoplasmic	8,1,4,7,12,14
A1B1L0/Pden_1299	20	24	23	22	Proline--tRNA ligase (Prolyl-tRNA synthetase)	Cytoplasmic	8,6,4,7,12,14
A1B3A9/Pden_1908	20	23	24	23	Fumarate hydratase class II (Fumarase C)	Cytoplasmic	8,4,7,12,14
A1B1U1/Pden_1384	20	28	20	26	Ribonucleoside-diphosphate reductase class II	Cytoplasmic	6,1,8,4,7
A1AZB7/Pden_0499	20	27	28	26	Argininosuccinate synthase	Cytoplasmic	8,1,4,7,12,14
A1B835/Pden_3612	20	31	28	26	Isoleucine--tRNA ligase	Cytoplasmic	8,6,4,7,12,14
A1B0F6/Pden_0889	19	20	18	15	50S ribosomal protein L9	Cytoplasmic	6,1,8,4,7
A1B616/Pden_2876	19	15	20	20	ATP synthase subunit b	Unknown	7,4,12,14,5,13
A1B543/Pden_2550	19	19	20	17	Electron transfer flavoprotein beta-subunit	Cytoplasmic	0
A1AZV8/Pden_0690	19	14	25	20	ATP-dependent zinc metalloprotease (FtsH)	Membrane	8,7,2
A1B0B6/Pden_0848	19	18	22	22	Malate dehydrogenase	Cytoplasmic	4,7,12,14

A1BAB6/Pden_4396	19	20	22	21	Adenylyl-sulfate kinase	Cytoplasmic	4
A1B894/Pden_3671	19	19	21	23	Protease FtsH subunit HflC	Cytoplasmic	0
A1B8W3/Pden_3891	19	21	22	22	Transketolase, central region	Cytoplasmic	0
A1AZH6/Pden_0558	19	20	23	22	Succinate--CoA ligase [ADP-forming] subunit alpha	Cytoplasmic	0
A1B0M6/Pden_0959	19	21	21	24	Uncharacterized protein	Unknown	0
A1B9C0/Pden_4048	19	20	21	26	NAD(P) transhydrogenase subunit alpha	Membrane	5,13
A1B5Q5/Pden_2765	19	23	25	21	Leucine--tRNA ligase (Leucyl-tRNA synthetase)	Cytoplasmic	8,6,4,7,12,14
A1B8W2/Pden_3890	19	23	24	24	Acetyltransferase component of pyruvate dehydrogenase complex	Cytoplasmic	4,7,12,14
A1AZF8/Pden_0540	18	14	12	14	Glycine--tRNA ligase beta subunit (Glycyl-tRNA synthetase beta subunit) (GlyRS)	Cytoplasmic	8,6,4,7,12,14
A1AZS3/Pden_0655	18	15	13	16	Nitrite/sulfite reductase, hemoprotein beta-component, ferrodoxin domain protein	Cytoplasmic	0
A1B352/Pden_1849	18	15	16	14	UspA domain protein	Cytoplasmic	11
A1B8B9/Pden_3696	18	17	16	15	GMP synthase (glutamine-hydrolyzing)	Cytoplasmic	8,1,4,7,12,14
A1AY14/Pden_0041	18	17	17	17	Glycine oxidase	Cytoplasmic	0
A1B2W9/Pden_1766	18	19	16	17	Nucleoside-binding protein	Unknown	0
A1B081/Pden_0813	18	21	19	13	Phosphoserine aminotransferase apoenzyme	Cytoplasmic	8,1,4,7,12,14
A1B0F1/Pden_0884	18	14	20	19	Glutamate--tRNA ligase 1 (Glutamyl-tRNA synthetase 1) (GluRS 1)	Cytoplasmic	8,6,4,7,12,14
A1B644/Pden_2904	18	19	19	18	3-oxoacyl-[acyl-carrier-protein] synthase 2	Cytoplasmic	8,1,4,7,12,14
A1B027/Pden_0759	18	16	20	20	50S ribosomal protein L3	Cytoplasmic	6,1,8,4,7
A1AZM6/Pden_0608	18	19	20	19	Extracellular solute-binding protein, family 5	Periplasmic	0
A1B030/Pden_0762	18	22	20	18	50S ribosomal protein L2	Cytoplasmic	6,1,8,4,7
A1AY63/Pden_0090	18	21	21	20	Uncharacterized protein	Cytoplasmic	1
A1B904/Pden_3932	18	20	20	22	Amino acid/amide ABC transporter substrate-binding protein, HAAT family	Periplasmic	0
A1B373/Pden_1870	18	21	21	21	Arginine--tRNA ligase (Arginyl-tRNA synthetase)	Cytoplasmic	8,6,4,7,12,14
A1B2B1/Pden_1555	18	22	22	20	3,4-dihydroxy-2-butanone 4-phosphate synthase (DHBP synthase)	Cytoplasmic	1,6,4,7,12,14
A1B8D9/Pden_3716	18	22	18	25	Citrate synthase	Cytoplasmic	8,4,7,12,14
A1B684/Pden_2944	18	21	25	25	Phosphomethylpyrimidine synthase	Cytoplasmic	1,6,4,7,12,14
A1AZM1/Pden_0603	17	16	13	18	Adenylosuccinate synthetase (AMPSase) (AdSS) (IMP--aspartate ligase)	Cytoplasmic	8,1,4,7,12,14
A1B5P5/Pden_2755	17	16	17	15	Aldehyde dehydrogenase	Cytoplasmic	0
A1B417/Pden_2169	17	18	19	15	Acetolactate synthase	Cytoplasmic	8,1,4,7,12,14
A1B3D2/Pden_1933	17	20	15	18	O-acetylhomoserine sulfhydrolase	Cytoplasmic	0
A1B9A2/Pden_4030	17	15	21	18	2-isopropylmalate synthase	Cytoplasmic	8,1,4,7,12,14
A1BB40/Pden_4673	17	14	19	21	Band 7 protein	Membrane	0
A1B906/Pden_3934	17	17	23	21	Phospho-2-dehydro-3-deoxyheptonate aldolase	Unknown	8,1,4,7,12,14
Q51664/Pden_2482	17	21	21	20	Protein NorQ	Cytoplasmic	0
A1B8N9/Pden_3817	17	22	21	21	ATP synthase gamma chain	Membrane	7,4,12,14,5,13
A1B1F9/Pden_1248	17	22	23	22	Extracellular solute-binding protein, family 5	Periplasmic	12,5,13
A1B3J4/Pden_1996	17	20	25	24	Uncharacterized protein	Unknown	0
A1BAK7/Pden_4487	17	18	27	25	Cell division protein FtsZ	Cytoplasmic	3,12,30
A1B5L7/Pden_2727	16	15	14	11	Type I secretion protein, TolC family	OuterMembrane	0
A1B559/Pden_2566	16	15	12	14	Uncharacterized protein	Unknown	0
A1B989/Pden_4017	16	12	16	15	Amino acid/amide ABC transporter substrate-binding protein, HAAT family	Unknown	0
A1B049/Pden_0781	16	12	18	15	Adenylate kinase (AK)	Cytoplasmic	8,1,4,7,12,14
A1B8V6/Pden_3884	16	13	14	18	50S ribosomal protein L19	Cytoplasmic	6,1,8,4,7

A1B4Y3/Pden_2490	16	16	15	18	Cytochrome d1, heme region	Unknown	0
A1B3C9/Pden_1929	16	18	16	19	Aspartyl/glutamyl-tRNA(Asn/Gln) amidotransferase subunit B	Cytoplasmic	6,1,8,4,7
A1AY62/Pden_0089	16	19	18	18	Uncharacterized protein	Cytoplasmic	0
A1B936/Pden_3964	16	19	18	18	Iron-regulated ABC transporter component (SufB)	Cytoplasmic	1,3
A1B331/Pden_1828	16	18	22	19	Helicase domain protein	Cytoplasmic	0
A1B5K8/Pden_2718	16	21	21	20	Bifunctional purine biosynthesis protein (PurH)	Cytoplasmic	8,1,4,7,12,14
A1AZS7/Pden_0659	16	21	22	21	DNA topoisomerase 1 (DNA topoisomerase I)	Cytoplasmic	6,8,3,4,7
A1BAG7/Pden_4447	15	12	12	12	Extracellular solute-binding protein, family 1	Periplasmic	0
A1B495/Pden_2247	15	14	13	12	NADH-quinone oxidoreductase subunit D	Cytoplasmic	5
A1B9C3/Pden_4051	15	11	16	12	Malate synthase G	Cytoplasmic	8,4,7,12,14
A1B3M5/Pden_2027	15	17	15	15	3-oxoacyl-[acyl-carrier-protein] reductase	Cytoplasmic	1,7
A1B1I2/Pden_1271	15	17	17	15	Acetyl-CoA carboxylase carboxyltransferase subunit alpha	Cytoplasmic	0
A1B997/Pden_4025	15	14	20	15	GTP-binding protein TypA	Membrane	0
A1B8E0/Pden_3717	15	15	16	19	Glutamate--tRNA ligase 2 (Glutamyl-tRNA synthetase 2) (GluRS 2)	Cytoplasmic	8,6,4,7,12,14
A1B691/Pden_2951	15	17	18	16	Toxic anion resistance family protein	Cytoplasmic	0
A1B4J2/Pden_2346	15	17	16	18	Glycine dehydrogenase (decarboxylating)	Cytoplasmic	4,8,7,12,14,2
A1B052/Pden_0784	15	16	17	18	DNA-directed RNA polymerase subunit alpha	Cytoplasmic	6,1,8,4,7
A1AZV4/Pden_0686	15	16	16	19	Protein TolB	Periplasmic	5,13,23
A1BAJ9/Pden_4479	15	17	17	18	Methylmalonate-semialdehyde dehydrogenase (Acylating)	Cytoplasmic	0
A1B301/Pden_1798	15	16	16	20	Sulfate ABC transporter, periplasmic sulfate-binding protein	Periplasmic	0
A1B033/Pden_0765	15	17	19	17	30S ribosomal protein S3	Cytoplasmic	6,1,8,4,7,
A1B061/Pden_0793	15	22	18	20	L-aspartate-binding protein / L-glutamate-binding protein / L-glutamine-binding protein / L-asparagine-binding protein	Unknown	0
A1BA35/Pden_4315	14	11	14	11	L-threonine synthase	Cytoplasmic	0
A1B1V2/Pden_1395	14	14	12	14	Aminotransferase, class V	Cytoplasmic	0
A1B8T3/Pden_3861	14	15	16	13	Pyridoxal-5'-phosphate-dependent enzyme, beta subunit	Cytoplasmic	8,1,4,7,12,14
A1B695/Pden_2955	14	16	16	13	Carboxynorspermidine dehydrogenase	Cytoplasmic	0
D1MWL3/Pden_0734	14	15	16	14	Elongation factor Tu (Fragment)	Unknown	0
A1B956/Pden_3984	14	17	10	18	Aminotransferase	Cytoplasmic	0
A1B9A4/Pden_4032	14	17	16	13	Rod shape-determining protein MreB	Cytoplasmic	3,12,31,17
A1B044/Pden_0776	14	15	15	16	30S ribosomal protein S5	Cytoplasmic	6,1,8,4,7
A1B039/Pden_0771	14	14	16	16	50S ribosomal protein L5	Cytoplasmic	6,1,8,4,7
A1B939/Pden_3967	14	14	15	17	SufBD protein	Cytoplasmic	1,3
A1AZ60/Pden_0440	14	15	17	15	3-isopropylmalate dehydrogenase (3-IPM-DH)	Cytoplasmic	8,1,4,7,12,14
A1B4A5/Pden_2257	14	15	17	15	Succinate semialdehyde dehydrogenase	Cytoplasmic	4,8,7,12,14,2
A1B3C5/Pden_1925	14	14	16	17	Histidine--tRNA ligase (Histidyl-tRNA synthetase) (HisRS)	Cytoplasmic	8,6,4,7,12,14
A1B1B9/Pden_1208	14	15	14	18	Urease subunit alpha (Urea amidohydrolase subunit alpha)	Cytoplasmic	4,6,7,12,14,2
A1B6A9/Pden_2969	14	14	18	16	50S ribosomal protein L13	Cytoplasmic	6,1,8,4,7
A1B0M2/Pden_0955	14	18	16	15	Threonine--tRNA ligase (Threonyl-tRNA synthetase) (ThrRS)	Cytoplasmic	8,6,4,7,12,14
A1B8C3/Pden_3700	14	16	15	18	Carboxypeptidase Taq, Metallo peptidase, MEROPS family M32	Cytoplasmic	0
A1B437/Pden_2189	14	18	18	15	Cobaltochelatase (CobS)	Cytoplasmic	0
A1B8D4/Pden_3711	14	19	21	18	CTP synthase	Cytoplasmic	8,1,4,7,12,14
A1B503/Pden_2510	13	12	11	11	Lytic murein transglycosylase	Membrane	0

A1B6B0/Pden_2970	13	10	13	11	30S ribosomal protein S9	Cytoplasmic	6,1,8,4,7
A1B524/Pden_2531	13	12	13	12	Cobalamin biosynthesis protein (CobW)	Cytoplasmic	1,6,4,7,12,14
A1B7Y3/Pden_3557	13	15	12	12	DegT/DnrJ/EryC1/StrS aminotransferase	Cytoplasmic	0
A1B9U8/Pden_4228	13	12	14	13	Peptidase U32	Cytoplasmic	0
A1B476/Pden_2228	13	12	12	15	Beta-lactamase domain protein	Cytoplasmic	0
A1BAI3/Pden_4463	13	11	13	15	Adenylosuccinate lyase (ASL) (Adenylosuccinase)	Cytoplasmic	8,1,4,7,12,14
A1AZ45/Pden_0425	13	14	13	13	Electron-transferring-flavoprotein dehydrogenase	Unknown	0
A1B9K4/Pden_4134	13	12	15	15	Uncharacterized protein	OuterMembrane	0
A1BA18/Pden_4298	13	12	14	16	O-succinylhomoserine sulfhydrylase	Cytoplasmic	8,1,4,7,12,14
A1B595/Pden_2602	13	16	14	13	Branched chain amino acid aminotransferase	Cytoplasmic	8,6,4,7,12,14
A1B022/Pden_0754	13	15	14	15	30S ribosomal protein S7	Cytoplasmic	6,1,8,4,7
A1AZN6/Pden_0618	13	14	13	17	Bifunctional protein GlmU	Cytoplasmic	9,14,31,17,10,19
A1B491/Pden_2243	13	17	14	15	NADH dehydrogenase subunit F	Cytoplasmic	0
A1B3J3/Pden_1995	13	13	17	16	HemY domain protein	Unknown	0
A1AYV4/Pden_0334	13	13	20	14	Ribosome-binding ATPase YchF	Unknown	0
A1B501/Pden_2508	13	16	17	16	Carboxyl-terminal protease	Membrane	0
A1AZS6/Pden_0658	13	16	13	20	Ferredoxin--NADP(+) reductase	Cytoplasmic	0
A1B4C4/Pden_2276	13	15	20	18	Pyruvate kinase	Cytoplasmic	0
A1B9E4/Pden_4072	13	21	18	22	RNA polymerase sigma factor RpoD (σ^{70})	Cytoplasmic	6,1,8,4,7
A1B5X4/Pden_2834	13	22	24	26	DNA polymerase III subunit beta	Cytoplasmic	6,1,8,4,7
A1B2K6/Pden_1653	12	8	8	9	Lytic murein transglycosylase	Membrane	0
A1B494/Pden_2246	12	9	8	10	NADH dehydrogenase subunit E	Unknown	0
A1B597/Pden_2604	12	11	13	11	SSU ribosomal protein S30P/sigma 54 modulation protein	Cytoplasmic	8
A1B617/Pden_2877	12	12	12	12	ATP synthase subunit b (ATP synthase F(0) sector subunit b)	Unknown	7,4,12,14,5,13
A1B2A7/Pden_1551	12	11	13	12	Uncharacterized protein	OuterMembrane	0
Q51676/Pden_1851	12	9	13	14	Oxygen-independent coproporphyrinogen III oxidase (CPO)	Cytoplasmic	6,1,4,7
A1B2B9/Pden_1563	12	14	12	11	TRAP dicarboxylate transporter-DctP subunit	Periplasmic	5
A1B4R9/Pden_2426	12	13	13	11	Pyrrolo-quinoline quinone	OuterMembrane	0
A1BAG4/Pden_4444	12	13	13	12	Uncharacterized protein	Unknown	0
A1B5P6/Pden_2756	12	12	14	12	1-Cys peroxiredoxin	Cytoplasmic	0
A1B3Y1/Pden_2133	12	9	14	15	Putative peptidyl-prolyl cis-trans isomerase	Membrane	0
A1B047/Pden_0779	12	12	11	16	50S ribosomal protein L15	Cytoplasmic	6,1,8,4,7
A1BA64/Pden_4344	12	12	13	15	Sulfate ABC transporter, periplasmic sulfate-binding protein	Periplasmic	0
A1B1M5/Pden_1314	12	15	15	11	Periplasmic glucan biosynthesis protein (MdoG)	Periplasmic	1,8,7,14
A1B2U6/Pden_1743	12	12	14	15	DEAD/DEAH box helicase domain protein	Cytoplasmic	0
A1B938/Pden_3966	12	13	13	16	FeS assembly ATPase SufC	Cytoplasmic	5
A1B8N4/Pden_3812	12	16	13	15	ATP-dependent Clp protease, ATP-binding subunit clpA	Cytoplasmic	0
A1AZA8/Pden_0490	12	13	16	15	Glutamate synthase (NADPH) small subunit	Cytoplasmic	8,1,4,7,12,14
A1B3D4/Pden_1935	12	12	17	15	Methionine synthase (B12-dependent)	Cytoplasmic	6,4,7
A1B4Z2/Pden_2499	12	14	15	16	Probable transaldolase	Cytoplasmic	8,6,4,7,12,14
A1B377/Pden_1876	12	11	20	15	Fructose-1,6-bisphosphatase	Cytoplasmic	8,1,4,7,12,14
A1B893/Pden_3670	12	13	17	17	Protease FtsH subunit (HflK)	Cytoplasmic	0
A1B5R6/Pden_2776	12	14	14	19	Uncharacterized protein	Unknown	0
A1B943/Pden_3971	12	14	15	19	Phosphoribosylformylglycinamidine synthase subunit (PurL)	Cytoplasmic	8,1,4,7,12,14

A1B9T3/Pden_4213	12	17	18	14	Acetyl-coenzyme A synthetase	Cytoplasmic	1,4,7,12,14
A1BAR9/Pden_4550	12	17	18	14	Acetyl-coenzyme A synthetase	Cytoplasmic	1,4,7,12,14
A1B0C9/Pden_0862	12	19	13	17	Aspartokinase	Cytoplasmic	8,1,4,7,12,14
A1B832/Pden_3609	12	21	15	18	Lon protease (ATP-dependent protease La)	Cytoplasmic	11,8,7,13
A1B6A4/Pden_2964	12	19	20	18	Two component, sigma54 specific, transcriptional regulator, Fis family	Cytoplasmic	1,6,8,4,7,9
A1B673/Pden_2933	12	21	17	20	Dihydroxy-acid dehydratase (DAD)	Cytoplasmic	8,1,4,7,12,14
A1B8T7/Pden_3865	12	19	20	21	DNA gyrase subunit A	Cytoplasmic	1,6,8,3,4,7
A1B1J4/Pden_1283	11	9	7	7	3-dehydroquinate synthase	Cytoplasmic	8,1,4,7,12,14
A1B591/Pden_2598	11	8	6	9	Peptidylprolyl isomerase	Membrane	0
A1AXY0/Pden_0007	11	10	10	8	ParB-like partition protein	Cytoplasmic	0
A1B4E4/Pden_2297	11	10	10	9	UDP-glucose pyrophosphorylase	Cytoplasmic	1
A1AY12/Pden_0039	11	8	11	10	Thiazole synthase	Cytoplasmic	6,1,4,7
A1B4L2/Pden_2366	11	10	10	10	Aldehyde dehydrogenase (NAD(+))	Cytoplasmic	0
A1B4X4/Pden_2481	11	10	10	10	von Willebrand factor, type A	Unknown	0
A1B4F2/Pden_2305	11	10	11	11	Ubiquinol-cytochrome c reductase iron-sulfur subunit	Membrane	0
A1AZD6/Pden_0518	11	11	11	11	Superoxide dismutase	Periplasmic	0
A1B496/Pden_2248	11	14	9	12	NADH-quinone oxidoreductase subunit C	Cytoplasmic	5
A1B544/Pden_2551	11	10	13	12	Electron transfer flavoprotein, alpha subunit	Unknown	0
A1B882/Pden_3659	11	10	13	12	Inorganic diphosphatase	Cytoplasmic	0
A1B474/Pden_2226	11	12	14	10	3-phosphoshikimate 1-carboxyvinyltransferase (5-enolpyruvylshikimate-3-phosphate synthase)	Cytoplasmic	8,1,4,7,12,14
A1B0K1/Pden_0934	11	13	12	11	Flavin-dependent thymidylate synthase	Cytoplasmic	8,1,4,7,12,14
A1B335/Pden_1832	11	13	12	11	Oligoendopeptidase, pepF/M3 family	Cytoplasmic	0
A1B8P5/Pden_3823	11	13	12	11	Ribose-phosphate pyrophosphokinase (RPPK)	Cytoplasmic	8,1,4,7,12,14
A1B0G6/Pden_0899	11	10	14	12	Chaperone (SurA)	Unknown	0
A1B050/Pden_0782	11	10	12	14	30S ribosomal protein S13	Cytoplasmic	6,1,8,4,7
A1B2U9/Pden_1746	11	12	13	12	Nucleoside diphosphate kinase (NDK) (NDP kinase) (Nucleoside-2-P kinase)	Extracellular	8,1,4,7,12,14
A1B9C5/Pden_4053	11	14	11	13	Alanyl aminopeptidase, Metallo peptidase, MEROPS family M01	Unknown	0
A1B8U4/Pden_3872	11	15	12	12	Glutamate dehydrogenase	Unknown	8,6,4,7,12,14
A1BB79/Pden_4712	11	16	13	11	Site-determining protein	Cytoplasmic	12
A1B2K3/Pden_1650	11	13	15	13	3-oxoacyl-[acyl-carrier-protein] synthase I	Cytoplasmic	0
A1B1M8/Pden_1317	11	11	16	14	Formate--tetrahydrofolate ligase	Cytoplasmic	8,1,4,7,12,14
A1B0C5/Pden_0857	11	14	15	13	Enoyl-[acyl-carrier-protein] reductase [NADH]	Membrane	8,1,4,7,12,14
A1B4A6/Pden_2258	11	12	16	16	Uncharacterized protein	Unknown	0
A1B053/Pden_0785	11	14	18	19	50S ribosomal protein L17	Cytoplasmic	6,1,8,4,7
A1B8W4/Pden_3892	11	17	14	20	Pyruvate dehydrogenase E1 component subunit alpha	Cytoplasmic	1,4,7,12,14
A1B5H1/Pden_2678	11	18	21	19	Uncharacterized protein	Unknown	0
A1AZI4/Pden_0566	10	10	8	9	L-erythro-3-methylmalyl-CoA dehydratase	Cytoplasmic	0
A1B3N9/Pden_2041	10	8	12	8	Acetylornithine aminotransferase (ACOAT)	Cytoplasmic	8,1,4,7,12,14
A1AZ46/Pden_0426	10	9	9	10	Transcription elongation factor GreA (Transcript cleavage factor GreA)	Cytoplasmic	1,6,8,4,7,9
A1BA25/Pden_4305	10	10	10	9	Triosephosphate isomerase (TIM) (TPI) (Triose-phosphate isomerase)	Cytoplasmic	4,8,7,12,14,2
A1AXZ0/Pden_0017	10	10	10	11	ATP-dependent Clp protease proteolytic subunit (Endopeptidase Clp)	Cytoplasmic	0
A1B896/Pden_3673	10	12	10	10	Aminopyrimidine aminohydrolase	Unknown	1,6,4,7,12,14
A1AZN4/Pden_0616	10	11	11	10	Bacterioferritin	Cytoplasmic	12,5,13,10

A1B615/Pden_2875	10	11	11	10	OmpA/MotB domain protein	OuterMembrane	0
A1B032/Pden_0764	10	10	11	11	50S ribosomal protein L22	Cytoplasmic	6,1,8,4,7
A1B4Y8/Pden_2495	10	10	11	11	Cytochrome d1, heme region	Periplasmic	0
A1B9U4/Pden_4224	10	10	11	11	Regulatory protein Nnr	Cytoplasmic	0
A1B3T6/Pden_2088	10	14	10	9	Endonuclease	Unknown	0
A1B8Z8/Pden_3926	10	12	11	10	Phosphoribosylformylglycinamidine cyclo-ligase	Cytoplasmic	8,1,4,7,12,14
A1BAM3/Pden_4503	10	11	11	11	Gamma-glutamyl phosphate reductase (GPR)	Cytoplasmic	8,1,4,7,12,14
A1AY09/Pden_0036	10	11	13	10	NlpA lipoprotein	Unknown	1,6,4,7,12,14
A1B1T9/Pden_1382	10	11	9	14	Protease Do	Periplasmic	0
A1B3Z9/Pden_2151	10	9	11	14	Polyamine ABC trasnporter, periplasmic polyamine-binding protein	Periplasmic	0
A1B041/Pden_0773	10	11	11	13	30S ribosomal protein S8	Cytoplasmic	6,1,8,4,7
A1B4Z9/Pden_2506	10	11	13	12	2,3-bisphosphoglycerate-independent phosphoglycerate mutase	Cytoplasmic	4,8,7,12,14,2
A1AZJ0/Pden_0572	10	11	12	13	Succinate dehydrogenase iron-sulfur subunit	Membrane	8,4,7,12,14,
A1B374/Pden_1873	10	14	14	9	S-adenosylmethionine synthase	Cytoplasmic	8,1,4,7,12,14
A1B9E9/Pden_4077	10	12	14	13	50S ribosomal protein L25 (General stress protein CTC)	Cytoplasmic	6,1,8,4,7
A1B622/Pden_2882	10	11	15	14	5'-Nucleotidase domain protein	Periplasmic	4,8,7,12,14,2
A1B326/Pden_1823	10	13	14	15	Peptidase M20	Cytoplasmic	0
A1B4S2/Pden_2429	10	16	13	16	Serine--tRNA ligase (Seryl-tRNA synthetase)	Cytoplasmic	8,1,4,7,12,14
A1B4L0/Pden_2364	10	14	16	16	Alpha-2-macroglobulin domain protein	Unknown	0
A1B8X0/Pden_3898	9	8	8	5	Aldehyde dehydrogenase (NAD(+))	Cytoplasmic	0
A1B486/Pden_2238	9	6	8	8	NADH-quinone oxidoreductase subunit I	Cytoplasmic	0
A1B4W5/Pden_2472	9	6	7	9	Carbohydrate ABC transporter substrate-binding protein, CUT1 family	Periplasmic	0
A1B091/Pden_0823	9	8	6	9	Uncharacterized protein	Cytoplasmic	0
A1B2K9/Pden_1656	9	10	7	7	N-acetyl-gamma-glutamyl-phosphate reductase	Cytoplasmic	8,1,4,7,12,14
A1B3M0/Pden_2022	9	9	7	8	Argininosuccinate lyase (ASAL,Arginosuccinase)	Cytoplasmic	8,1,4,7,12,14
A1B011/Pden_0743	9	7	8	9	50S ribosomal protein L11	Cytoplasmic	6,1,8,4,7
A1B328/Pden_1825	9	7	8	9	Transcriptional regulator, CarD family	Cytoplasmic	0
A1B4L8/Pden_2372	9	10	8	7	Exodeoxyribonuclease III Xth	Cytoplasmic	4,8,7,12,14,13
A1B400/Pden_2152	9	7	11	7	Extracellular solute-binding protein, family 1	Periplasmic	0
A1AZK4/Pden_0586	9	9	8	8	UDP-N-acetylmuramoyl-L-alanyl-D-glutamate--2,6-diaminopimelate ligase	Cytoplasmic	12,7,14,9,10,19
A1B3F7/Pden_1959	9	8	6	11	Histidinol dehydrogenase (HDH)	Cytoplasmic	8,1,4,7,12,14
A1B401/Pden_2153	9	9	9	8	Polyamine-transporting ATPase	Membrane	0
A1B3K0/Pden_2002	9	12	7	8	Lipid A ABC exporter family, fused ATPase and inner membrane subunits	Membrane	0
A1B3D0/Pden_1931	9	6	12	9	Uncharacterized protein	Unknown	0
A1AZE0/Pden_0522	9	9	6	12	ErfK/YbiS/YcfS/YnhG family protein	Unknown	0
A1B092/Pden_0824	9	10	9	9	Uncharacterized protein	Unknown	0
A1B945/Pden_3973	9	10	9	9	Glutamate racemase	Cytoplasmic	12,7,14,9,10,19
A1B2C3/Pden_1567	9	12	9	8	Nicotinate-nucleotide--dimethylbenzimidazole phosphoribosyltransferase	Cytoplasmic	8,1,4,7,12,14
A1B9H5/Pden_4103	9	12	8	9	50S ribosomal protein L21	Unknown	6,1,8,4,7
A1BC43/Pden_5027	9	10	9	10	CDP-glycerol:poly(Glycerophosphate) glycerophosphotransferase	Membrane	0
A1B5A6/Pden_2613	9	12	9	9	Formyltetrahydrofolate deformylase (Formyl-FH(4) hydrolase)	Cytoplasmic	8,1,4,7,12,14
A1AZ54/Pden_0434	9	12	8	10	Acyl-CoA dehydrogenase domain protein	Cytoplasmic	0
A1B507/Pden_2514	9	11	9	10	2,3,4,5-tetrahydropyridine-2,6-dicarboxylate N-succinyltransferase	Cytoplasmic	8,1,4,7,12,14

A1B7X8/Pden_3552	9	11	9	10	ATP-dependent carboxylate-amine ligase domain protein, ATP-grasp	Cytoplasmic	0
A1B5L3/Pden_2723	9	9	11	11	Selenide, water dikinase (Selenium donor protein) (Selenophosphate synthase)	Cytoplasmic	8,1,4,7,12,14
A1B8M8/Pden_3806	9	10	9	12	Single-stranded DNA-binding protein (SSB)	Cytoplasmic	4,8,7,12,14,13
A1B934/Pden_3962	9	9	13	10	Phosphopantothenoylcysteine decarboxylase	Cytoplasmic	4,8,7,12,14,2
A1B3F9/Pden_1961	9	10	9	13	UDP-N-acetylglucosamine 1-carboxyvinyltransferase	Cytoplasmic	12,7,14,9,10,19
A1B539/Pden_2546	9	13	11	9	DNA topoisomerase 4 subunit A (Topoisomerase IV subunit A)	Cytoplasmic	8,6,3,4,7,12
A1B051/Pden_0783	9	12	10	11	30S ribosomal protein S11	Cytoplasmic	6,1,8,4,7
A1B026/Pden_0758	9	13	8	12	30S ribosomal protein S10	Cytoplasmic	6,1,8,4,7
A1B5S4/Pden_2784	9	10	11	12	Protein-export protein SecB	Cytoplasmic	25,3,5,23
A1BA86/Pden_4366	9	13	12	9	DEAD/DEAH box helicase domain protein	Cytoplasmic	0
A1B7Y2/Pden_3556	9	11	13	10	N-acetylneuraminate synthase	Cytoplasmic	1,8,7,14
A1B1H7/Pden_1266	9	11	12	11	ATP-dependent Clp protease ATP-binding subunit ClpX	Cytoplasmic	25
A1B010/Pden_0742	9	13	9	12	Transcription termination/antitermination protein NusG	Cytoplasmic	1,6,8,4,7,9
A1AZY4/Pden_0716	9	11	11	12	Amino acid ABC transporter substrate-binding protein, PAAT family	Periplasmic	0
A1B0F8/Pden_0891	9	10	11	14	30S ribosomal protein S6	Cytoplasmic	6,1,8,4,7
A1B042/Pden_0774	9	13	12	11	50S ribosomal protein L6	Cytoplasmic	6,1,8,4,7
A1AZX2/Pden_0704	9	12	13	11	4-hydroxy-tetrahydrodipicolinate synthase	Cytoplasmic	8,1,4,7,12,14
A1AY35/Pden_0062	9	8	12	16	Chaperone protein ClpB	Cytoplasmic	11,8,7,27
A1B3P3/Pden_2045	9	11	13	13	Methionine--tRNA ligase (Methionyl-tRNA synthetase) (MetRS)	Cytoplasmic	8,6,4,7,12,14
A1BBB2/Pden_4745	9	11	13	13	Sel1 domain protein repeat-containing protein	Unknown	0
A1B075/Pden_0807	9	12	11	14	ErfK/YbiS/YcfS/YnhG family protein	Unknown	0
A1B356/Pden_1853	9	10	15	13	DNA polymerase I	Cytoplasmic	6,1,8,4,7
A1AZJ4/Pden_0576	9	10	14	15	Glutamyl-tRNA(Gln) amidotransferase subunit A (Glu-ADT subunit A)	Cytoplasmic	6,1,8,4,7
A1B8A1/Pden_3678	9	14	11	17	Homoserine dehydrogenase	Cytoplasmic	8,6,4,7,12,14
A1B9K0/Pden_4130	8	8	7	5	Signal transduction histidine kinase, nitrogen specific, NtrB	Membrane	0
A1AZC9/Pden_0511	8	9	5	6	Putative cytochrome c-type biogenesis protein, CycH	Unknown	0
A1B1B4/Pden_1203	8	8	6	6	Uncharacterized protein	Unknown	0
A1B4Q1/Pden_2407	8	8	6	6	Mur ligase, middle domain protein	Membrane	12,7,14,9,10,19
A1B5L9/Pden_2729	8	7	7	6	Peptidase M23B	OuterMembrane	0
A1B4N7/Pden_2393	8	7	7	7	6-phosphogluconate dehydrogenase, decarboxylating	Unknown	8,6,4,7,12,14
A1B5L6/Pden_2726	8	7	7	9	Protein-L-isoaspartate(D-aspartate) O-methyltransferase	Cytoplasmic	0
A1B097/Pden_0829	8	9	9	7	Extracellular solute-binding protein, family 1	Unknown	0
A1B1F2/Pden_1241	8	9	8	8	UDP-3-O-acylglucosamine N-acyltransferase	Cytoplasmic	8,1,4,7,12,14
A1B4M3/Pden_2377	8	8	9	8	Short-chain dehydrogenase/reductase SDR	Cytoplasmic	0
A1B5Y5/Pden_2845	8	9	7	9	Glutamate--cysteine ligase	Cytoplasmic	6,1,4,7
A1AZP9/Pden_0631	8	8	7	10	3-mercaptopyruvate sulfurtransferase	Cytoplasmic	0
A1BA42/Pden_4322	8	8	6	11	Uncharacterized protein	Cytoplasmic	0
A1B310/Pden_1807	8	10	9	7	Extracellular solute-binding protein, family 5	Unknown	12,5,13
A1AZT2/Pden_0664	8	9	9	8	TRAP transporter solute receptor, TAXI family	Unknown	0
A1B2X3/Pden_1770	8	7	9	10	Malonyl CoA-acyl carrier protein transacylase	Cytoplasmic	0
A1B8B5/Pden_3692	8	12	9	6	PhoH family protein	Cytoplasmic	0
A1B9U0/Pden_4220	8	10	9	8	FMN-binding domain protein (NosR)	Membrane	9

A1B9U9/Pden_4229	8	10	8	9	Peptidase U32	Unknown	0
A1B064/Pden_0796	8	8	10	9	L-aspartate ABC transporter ATP-binding protein / L-glutamine ABC transporter ATP-binding protein / L-glutamate ABC transporter ATP-binding protein / L-asparagine ABC transporter ATP-binding protein	Membrane	0
A1B318/Pden_1815	8	9	8	10	Peptide chain release factor 2 (RF-2)	Cytoplasmic	0
A1B5H9/Pden_2686	8	6	9	12	Uncharacterized protein	Periplasmic	0
A1B0K5/Pden_0938	8	9	10	9	Aminotransferase	Cytoplasmic	1
A1B9K5/Pden_4135	8	9	9	10	Uncharacterized protein	Unknown	0
A1B515/Pden_2522	8	7	11	10	3-isopropylmalate dehydratase small subunit (Alpha-IPM isomerase)	Cytoplasmic	8,1,4,7,12,14
A1B037/Pden_0769	8	8	8	12	50S ribosomal protein L14	Cytoplasmic	6,1,8,4,7
A1B348/Pden_1845	8	10	10	9	*Cbb$_3$*-type cytochrome c oxidase subunit CcoP	Cytoplasmic	7,4,12,14,5,13
A1BAV5/Pden_4586	8	10	9	10	10 kDa chaperonin (GroES protein, Cpn10)	Cytoplasmic	25
A1B021/Pden_0753	8	8	11	10	30S ribosomal protein S12	Cytoplasmic	6,1,8,4,7
A1B0G0/Pden_0893	8	6	12	11	Cytochrome-*c* peroxidase	Unknown	0
A1B2Y3/Pden_1780	8	8	12	10	Efflux transporter, RND family, MFP subunit	Membrane	12,5,13
A1B4S0/Pden_2427	8	9	9	12	GTPase Der (GTP-binding protein EngA)	Membrane	20
A1B014/Pden_0746	8	8	12	11	50S ribosomal protein L7/L12	Unknown	6,1,8,4,7
A1B066/Pden_0798	8	11	10	11	Branched chain amino acid: 2-keto-4-methylthiobutyrate aminotransferase	Cytoplasmic	0
A1B4B8/Pden_2270	8	10	10	12	Phenylalanine--tRNA ligase alpha subunit	Cytoplasmic	8,6,4,7,12,14
A1BAB5/Pden_4395	8	11	13	9	Sulfate adenylyltransferase subunit 2	Cytoplasmic	1,4,12,14
A1B601/Pden_2861	8	13	10	11	Phosphoribosylaminoimidazole-succinocarboxamide synthase	Cytoplasmic	8,1,4,7,12,14
A1B339/Pden_1836	8	11	10	13	DNA topoisomerase 4 subunit B (Topoisomerase IV subunit B)	Cytoplasmic	8,6,3,4,7,12
A1BB82/Pden_4715	8	9	11	14	TRAP dicarboxylate transporter, DctP subunit	Periplasmic	5
A1BCB1/Pden_5095	8	12	10	13	Amino acid/amide ABC transporter substrate-binding protein, HAAT family	Periplasmic	0
A1B9U6/Pden_4226	8	11	11	13	3-octaprenyl-4hydroxybenzoate decarboxylase	Cytoplasmic	0
A1B8V3/Pden_3881	8	10	12	13	30S ribosomal protein S16	Cytoplasmic	6,1,8,4,7
A1B8N7/Pden_3815	8	10	11	14	ATP synthase subunit delta (ATP synthase F(1) sector subunit delta)	Cytoplasmic	7,4,12,14,5,13
A1B3E0/Pden_1941	8	8	13	14	Glyoxylate reductase	Cytoplasmic	0
A1B456/Pden_2208	8	13	12	12	Thioredoxin reductase	Cytoplasmic	4,12,14,21
A1B8V0/Pden_3878	8	10	13	15	Signal recognition particle protein (Fifty-four homolog)	Membrane	12,3,5,13,23,22
A1BAE3/Pden_4423	8	13	13	13	Phosphoglucomutase/phosphomannomutase alpha/beta/alpha domain I	Cytoplasmic	8,7
A1B0M4/Pden_0957	8	13	13	15	Polyhydroxyalkanoate depolymerase, intracellular	Cytoplasmic	0
A1AZ36/Pden_0416	8	18	18	18	DNA gyrase subunit B	Cytoplasmic	1,6,8,3,4,7
A1B5F6/Pden_2663	7	3	3	6	Acetyl-CoA acetyltransferase	Cytoplasmic	0
A1BB50/Pden_4683	7	7	6	4	3-oxoacid CoA-transferase, A subunit	Cytoplasmic	0
A1B2N1/Pden_1678	7	5	8	4	Inositol 2-dehydrogenase	Unknown	4,8,7,12,14,2
A1AYS8/Pden_0308	7	6	6	5	Uncharacterized protein UPF0065	Periplasmic	0
A1B4G8/Pden_2321	7	5	7	5	Orn/DAP/Arg decarboxylase 2	Cytoplasmic	6,1,4,7
A1AYM5/Pden_0255	7	3	6	8	Uncharacterized protein	Cytoplasmic	0
A1B608/Pden_2868	7	5	7	6	Acyl-CoA dehydrogenase domain protein	Cytoplasmic	0
A1BAK5/Pden_4485	7	6	6	7	Protein assembly factor (BamD)	Unknown	3,12,5
A1AZ44/Pden_0424	7	7	7	6	Tetratricopeptide TPR_2 repeat protein	Unknown	0
P54810/Pden_2026	7	7	7	6	Acetyl-CoA acetyltransferase (Acetoacetyl-CoA thiolase)	Unknown	0

A1B029/Pden_0761	7	7	6	7	50S ribosomal protein L23	Cytoplasmic	6,1,8,4,7
A1B5G6/Pden_2673	7	7	6	7	DNA translocase FtsK	Membrane	12
A1BAJ4/Pden_4474	7	7	5	8	Uncharacterized protein	Cytoplasmic	0
A1B545/Pden_2552	7	7	4	9	3-hydroxyacyl-CoA dehydrogenase	Cytoplasmic	8,4,7,12,14
A1BCE1/Pden_5125	7	6	5	9	Putative monooxygenase protein	Cytoplasmic	0
A1B3X4/Pden_2126	7	8	9	5	Capsule polysaccharide export protein	Membrane	0
A1BC54/Pden_5038	7	8	9	5	Capsule polysaccharide export protein	Membrane	0
A1B3M9/Pden_2031	7	8	8	6	Trans-hexaprenyltranstransferase	Cytoplasmic	8,1,4,7,12,14
A1B2K5/Pden_1652	7	7	9	6	Hydrolase or acyltransferase	Cytoplasmic	0
A1B4E7/Pden_2300	7	6	8	8	3'(2'),5'-bisphosphate nucleotidase	Cytoplasmic	8,4,7,12,14
A1B364/Pden_1861	7	5	7	10	3-oxoacyl-[acyl-carrier-protein] synthase 3	Cytoplasmic	8,1,4,7,12,14
A1B391/Pden_1890	7	8	8	7	Ribonuclease D (RNase D)	Cytoplasmic	6,8,4,7
A1B5Q0/Pden_2760	7	9	6	8	30S ribosomal protein S15	Cytoplasmic	6,1,8,4,7
A1B6E6/Pden_3007	7	7	8	8	TonB-dependent siderophore receptor	OuterMembrane	5,13
A1B2B0/Pden_1554	7	8	6	9	6,7-dimethyl-8-ribityllumazine synthase	Cytoplasmic	1,6,4,7,12,14
A1BA52/Pden_4332	7	7	7	9	Chorismate synthase (CS)	Cytoplasmic	8,1,4,7,12,14
A1AZ51/Pden_0431	7	6	7	10	Uncharacterized protein	Unknown	0
A1B3U3/Pden_2095	7	9	7	8	DSBA oxidoreductase	Unknown	0
A1B929/Pden_3957	7	9	7	8	Amino acid/amide ABC transporter substrate-binding protein, HAAT family	Unknown	0
A1B8L3/Pden_3791	7	7	7	10	Tryptophan synthase beta chain	Cytoplasmic	0
A1B034/Pden_0766	7	8	9	8	50S ribosomal protein L16	Cytoplasmic	6,1,8,4,7
A1B8A4/Pden_3681	7	10	9	7	Methylmalonyl-CoA mutase	Cytoplasmic	0
A1B036/Pden_0768	7	8	8	10	30S ribosomal protein S17	Cytoplasmic	6,1,8,4,7
A1B5S2/Pden_2782	7	8	8	10	Histone family protein nucleoid-structuring protein H-NS	Unknown	9
A1B4I6/Pden_2339	7	10	8	9	Acetyl-coenzyme A carboxylase carboxyl transferase subunit alpha (ACCase subunit alpha)	Cytoplasmic	8,1,4,7,12,14
A1B3B7/Pden_1917	7	8	10	9	Peptidyl-prolyl cis-trans isomerase (PPIase)	Periplasmic	25
A1AZM8/Pden_0610	7	9	10	9	Amidohydrolase	Cytoplasmic	0
A1B1N5/Pden_1324	7	7	12	9	Oligopeptide/dipeptide ABC transporter, ATPase subunit	Membrane	5,13
A1BAG9/Pden_4449	7	7	11	10	Assimilatory nitrate reductase (NADH) alpha subunit apoprotein	Cytoplasmic	0
A1AZL5/Pden_0597	7	10	8	11	Protein RecA (Recombinase A)	Cytoplasmic	7,4,12,14,13,29
A1AZK7/Pden_0589	7	11	10	9	UDP-N-acetylmuramoylalanine--D-glutamate ligase	Unknown	12,7,14,9,10,19
A1B2X2/Pden_1769	7	10	9	11	3-oxoacyl-[acyl-carrier-protein] reductase	Unknown	8,1,4,7,12,14
A1B892/Pden_3669	7	10	13	8	NADPH-glutathione reductase	Cytoplasmic	12,9,10
A1B1X3/Pden_1416	7	8	12	12	Protein translocase subunit SecD	Membrane	12,5,13,23
A1AZB0/Pden_0492	7	8	10	14	NAD-dependent epimerase/dehydratase	Cytoplasmic	0
A1B5Z6/Pden_2856	7	14	11	8	Formate dehydrogenase alpha subunit	Cytoplasmic	4,7,12,14
A1B9A1/Pden_4029	7	13	11	9	Phosphoribosylamine--glycine ligase (GARS)	Cytoplasmic	8,1,4,7,12,14
A1BCE3/Pden_5127	6	3	3	3	Transcriptional regulator, Fis family	Unknown	0
A1B439/Pden_2191	6	4	2	4	Cobaltochelatase CobT subunit	Cytoplasmic	1,6,4,7,12,14
A1B4H1/Pden_2324	6	4	2	4	FAD-dependent pyridine nucleotide-disulfide oxidoreductase	Cytoplasmic	12,9,10
A1B9F5/Pden_4083	6	3	3	4	DNA ligase (Polydeoxyribonucleotide synthase [NAD(+)])	Cytoplasmic	4,8,7,12,14,13
A1B887/Pden_3664	6	5	3	3	L-threonine aldolase	Cytoplasmic	4,8,7,12,14,2
A1B508/Pden_2515	6	3	3	5	Uncharacterized protein	Cytoplasmic	0

A1B990/Pden_4018	6	3	4	5	Amino acid/amide ABC transporter membrane protein 1, HAAT family	Membrane	0
A1B610/Pden_2870	6	5	5	3	Acetyl-CoA acetyltransferase	Cytoplasmic	0
A1BAM2/Pden_4502	6	4	5	4	ABC polyamine transporter, periplasmic substrate-binding protein	Periplasmic	5,13
A1B9D8/Pden_4066	6	6	3	5	ABC transporter related protein	Membrane	0
A1B2T8/Pden_1735	6	4	4	6	Dyp-type peroxidase family	Periplasmic	12,5,13,10
A1B5W2/Pden_2822	6	5	7	4	Response regulator receiver protein	Cytoplasmic	1,6,8,4,7,9
A1B4A0/Pden_2252	6	6	5	5	UvrABC system protein A (UvrA protein) (Excinuclease ABC subunit A)	Cytoplasmic	7,4,12,14,13,29
A1BBI4/Pden_4817	6	6	5	5	TRAP dicarboxylate transporter, DctP subunit	Periplasmic	5
A1B8C8/Pden_3705	6	6	6	5	Amino acid ABC transporter substrate-binding protein, PAAT family	Periplasmic	0
A1BAE9/Pden_4429	6	9	4	5	Alpha-1,4 glucan phosphorylase	Cytoplasmic	8,7
A1B3A6/Pden_1905	6	6	7	5	Probable transcriptional regulatory protein	Cytoplasmic	1,6,8,4,7,9
A1B1T7/Pden_1380	6	8	4	6	Uncharacterized protein	Unknown	0
A1B363/Pden_1860	6	5	7	6	Phosphate acyltransferase	Cytoplasmic	8,1,4,7,12,14
A1B435/Pden_2187	6	5	7	6	D-lactate dehydrogenase (Cytochrome)	Cytoplasmic	0
A1B5S1/Pden_2781	6	5	6	7	Two component transcriptional regulator, Fis family	Cytoplasmic	9
A1B0I9/Pden_0922	6	6	4	8	Peptide chain release factor 3 (RF-3)	Cytoplasmic	9
A1B311/Pden_1808	6	5	5	8	Cytochrome c, class I	Membrane	0
A1B965/Pden_3993	6	8	5	6	Uridylate kinase (UK)	Cytoplasmic	8,1,4,7,12,14
A1B366/Pden_1863	6	7	6	6	Integration host factor subunit alpha (IHF-alpha)	Cytoplasmic	1,6,8,4,7,9
A1B3Z1/Pden_2143	6	7	6	6	Serine-type D-Ala-D-Ala carboxypeptidase	Membrane	0
A1B8R2/Pden_3840	6	6	7	6	Uncharacterized protein	Cytoplasmic	0
A1BAL4/Pden_4494	6	6	7	6	UDP-N-acetylmuramate--L-alanine ligase	Cytoplasmic	12,7,14,9,10,19
A1B0E4/Pden_0877	6	7	5	7	Membrane protein insertase YidC (Foldase YidC)	Membrane	5,23
A1B452/Pden_2204	6	5	7	7	Uncharacterized protein	Unknown	0
A1BA12/Pden_4292	6	6	5	8	50S ribosomal protein L33	Cytoplasmic	6,1,8,4,7
A1B1L7/Pden_1306	6	6	8	6	Cobyrinic acid a,c-diamide synthase	Cytoplasmic	0
A1B3C7/Pden_1927	6	7	6	7	Uncharacterized protein	Unknown	0
A1B1X4/Pden_1417	6	7	5	8	Protein translocase subunit yajC	Membrane	0
A1B383/Pden_1882	6	7	5	8	Uncharacterized protein	Cytoplasmic	0
A1B1H6/Pden_1265	6	5	7	8	ATP-dependent Clp protease proteolytic subunit (Endopeptidase Clp)	Cytoplasmic	0
A1B6A2/Pden_2962	6	6	5	9	Phosphoribosylformylglycinamidine synthase subunit PurQ	Cytoplasmic	8,1,4,7,12,14
A1BAD7/Pden_4417	6	8	7	6	Elongation factor 4 (EF-4) (Ribosomal back-translocase LepA)	Membrane	9
P54414/Pden_0017	6	7	7	7	ATP-dependent Clp protease proteolytic subunit (Endopeptidase Clp)	Unknown	0
A1AY08/Pden_0035	6	7	7	7	Aminopyrimidine aminohydrolase	Unknown	6,4,7,12,14
A1B387/Pden_1886	6	7	6	8	1-(5-phosphoribosyl)-5-[(5-phosphoribosylamino)methylideneamino] imidazole-4-carboxamide isomerase	Cytoplasmic	8,1,4,7,12,14
A1B0L8/Pden_0951	6	6	6	9	Ribonucleoside-diphosphate reductase, adenosylcobalamin-dependent	Cytoplasmic	6,1,8,4,7
A1B7X9/Pden_3553	6	9	7	6	NAD-dependent epimerase/dehydratase	Unknown	0
A1B2T9/Pden_1736	6	7	9	6	Uncharacterized protein	Periplasmic	0
A1B611/Pden_2871	6	7	9	6	3-hydroxyacyl-CoA dehydrogenase	Cytoplasmic	4,8,7,12,14,2
A1B932/Pden_3960	6	6	7	9	Glutathione synthetase	Cytoplasmic	0
A1B852/Pden_3629	6	9	7	7	Uroporphyrinogen decarboxylase	Cytoplasmic	6,1,4,7

A1AY15/Pden_0042	6	8	7	8	Hydroxymethylpyrimidine kinase / phosphomethylpyrimidine kinase	Unknown	1,6,4,7,12,14
A1B853/Pden_3630	6	9	8	7	Porphobilinogen deaminase (PBG)	Cytoplasmic	6,1,8,4,7
A1B418/Pden_2170	6	10	6	8	Acetolactate synthase, small subunit	Cytoplasmic	8,1,4,7,12,14
Q9WX79/Pden_0957	6	8	7	9	Intracellular poly(3-hydroxyalkanoate) depolymerase	Unknown	0
A1B944/Pden_3972	6	8	7	9	Transcriptional regulator, LysR family	Cytoplasmic	6,1,8,4,7
A1B9J7/Pden_4127	6	8	7	9	Two component, sigma54 specific, transcriptional regulator, Fis family	Cytoplasmic	1,6,8,4,7,9
A1B038/Pden_0770	6	6	9	9	50S ribosomal protein L24	Cytoplasmic	6,1,8,4,7
A1B5T3/Pden_2793	6	8	8	9	Thioredoxin	Cytoplasmic	7,12,14,9,10
A1AZ25/Pden_0405	6	6	9	10	Histone family protein DNA-binding protein	Cytoplasmic	0
A1B8A7/Pden_3684	6	8	12	6	Biotin carboxyl carrier protein / biotin carboxylase	Cytoplasmic	0
A1B5S6/Pden_2786	6	8	10	8	Translocase, subunit Tim44	Unknown	0
P80649/Pden_4222	6	8	9	9	Pseudoazurin	Periplasmic	0
A1BAA2/Pden_4382	6	9	7	10	TonB-dependent receptor	OuterMembrane	5
A1B872/Pden_3649	6	8	8	10	DNA polymerase III, subunits gamma and tau	Cytoplasmic	6,1,8,4,7
A1B911/Pden_3939	6	11	9	8	Amidophosphoribosyltransferase (ATase)	Cytoplasmic	8,1,4,7,12,14
A1B067/Pden_0799	6	9	11	8	Citrate (Pro-3S)-lyase	Cytoplasmic	0
A1B449/Pden_2201	6	10	10	9	N5-carboxyaminoimidazole ribonucleotide synthase (N5-CAIR synthase)	Membrane	8,1,4,7,12,14
A1B322/Pden_1819	6	7	10	13	DSBA oxidoreductase	Unknown	0
A1B043/Pden_0775	6	8	10	13	50S ribosomal protein L18	Cytoplasmic	6,1,8,4,7
A1BA97/Pden_4377	6	15	8	12	Ribonucleoside-diphosphate reductase	Cytoplasmic	6,1,8,4,7
A1B9V7/Pden_4237	5	2	2	1	Nitrite transporter	Membrane	0
A1B2M8/Pden_1675	5	3	3	2	3D-(3,5/4)-trihydroxycyclohexane-1,2-dione hydrolase	Cytoplasmic	4,8,7,12,14,2
A1B1K1/Pden_1290	5	3	4	2	Orn/DAP/Arg decarboxylase 2	Cytoplasmic	0
A1B371/Pden_1868	5	3	2	4	Beta-N-acetylhexosaminidase	Cytoplasmic	8,7
A1B9S2/Pden_4202	5	2	3	4	Hemin-degrading family protein	Cytoplasmic	5,13
A1B1H8/Pden_1267	5	4	3	3	NADH:ubiquinone oxidoreductase 17.2 kD subunit	Unknown	0
A1BAI7/Pden_4467	5	5	4	2	Dihydroorotase	Cytoplasmic	8,1,4,7,12,14
A1B8D7/Pden_3714	5	5	3	3	Short-chain dehydrogenase/reductase SDR	Unknown	0
A1BC52/Pden_5036	5	4	3	4	ParB-like partition protein	Cytoplasmic	0
A1BA61/Pden_4341	5	4	2	5	Sulfate-transporting ATPase	Membrane	0
A1B1W2/Pden_1405	5	4	6	2	Uncharacterized protein	Membrane	0
A1BC44/Pden_5028	5	5	3	4	Uncharacterized protein	Cytoplasmic	0
A1B300/Pden_1797	5	3	5	4	Sulfate-transporting ATPase	Membrane	0
A1BA41/Pden_4321	5	3	5	4	Cytochrome c oxidase subunit 2	Membrane	4,12,14
A1B5I0/Pden_2687	5	3	4	5	Uncharacterized protein	Cytoplasmic	0
A1BC39/Pden_5023	5	3	4	5	NAD-dependent epimerase/dehydratase	Cytoplasmic	8,7
A1B9C8/Pden_4056	5	2	5	5	GTPase Obg (GTP-binding protein Obg)	Cytoplasmic	20
A1AZG6/Pden_0548	5	5	4	4	ATPase associated with various cellular activities, AAA_5	Cytoplasmic	0
A1B703/Pden_3216	5	3	5	5	Tartronate semialdehyde reductase	Cytoplasmic	0
A1AZN9/Pden_0621	5	6	4	4	Peptide methionine sulfoxide reductase MsrA	Unknown	11,8,4,7
A1B4H2/Pden_2325	5	5	5	4	Thiol peroxidase (Atypical 2-Cys peroxiredoxin)	Unknown	0
A1AZI1/Pden_0563	5	4	6	4	Citryl-CoA lyase	Cytoplasmic	0
A1B0F7/Pden_0890	5	5	4	5	30S ribosomal protein S18	Cytoplasmic	6,1,8,4,7
A1B0M5/Pden_0958	5	2	4	8	Poly(R)-hydroxyalkanoic acid synthase, class I	Cytoplasmic	1,7

A1B1V7/Pden_1400	5	6	5	4	(P)ppGpp synthetase I, SpoT/RelA	Cytoplasmic	8,6,4,7,12,14
A1B4F0/Pden_2303	5	5	6	4	Chaperone protein DnaJ	Cytoplasmic	1,11,8,4,7,27
A1B3Y7/Pden_2139	5	5	4	6	Indole-3-glycerol phosphate synthase (IGPS)	Cytoplasmic	8,1,4,7,12,14
A1B565/Pden_2572	5	4	5	6	Ribulose-phosphate 3-epimerase	Cytoplasmic	8,6,4,7,12,14
A1B5I4/Pden_2691	5	4	5	6	Uncharacterized protein	Unknown	0
A1B890/Pden_3667	5	4	5	6	Bifunctional enzyme IspD/IspF	Cytoplasmic	8,1,4,7,12,14
A1B1L9/Pden_1308	5	3	4	8	UvrABC system protein B (Protein UvrB) (Excinuclease ABC subunit B)	Cytoplasmic	7,4,12,14,13,29
A1B2E4/Pden_1588	5	3	4	8	UvrABC system protein B (Protein UvrB) (Excinuclease ABC subunit B)	Cytoplasmic	7,4,12,14,13,29
A1AZR6/Pden_0648	5	7	5	4	Aminotransferase, class I and II	Cytoplasmic	1
A1AZJ9/Pden_0581	5	5	6	5	Iron-sulfur cluster carrier protein	Cytoplasmic	0
A1B8B2/Pden_3689	5	5	6	5	Alanine racemase	Cytoplasmic	8,1,4,7,12,14
A1AYU3/Pden_0323	5	4	7	5	Biotin carboxyl carrier protein / biotin carboxylase	Cytoplasmic	0
A1B5Y0/Pden_2840	5	6	4	6	Acyl-CoA dehydrogenase	Cytoplasmic	0
A1AXY9/Pden_0016	5	5	5	6	S-(hydroxymethyl)glutathione dehydrogenase	Cytoplasmic	7,14
A1B3K2/Pden_2004	5	5	5	6	Peptidoglycan-binding LysM	Unknown	0
A1B4C1/Pden_2273	5	5	5	6	50S ribosomal protein L20	Cytoplasmic	1,6,8,3,4,7
A1B0L2/Pden_0945	5	4	6	6	Substrate-binding region of ABC-type glycine betaine transport system	Periplasmic	0
A1B3I7/Pden_1989	5	7	6	4	AFG1-family ATPase	Cytoplasmic	0
A1B576/Pden_2583	5	6	7	4	Ferrochelatase (Heme synthase) (Protoheme ferro-lyase)	Cytoplasmic	1,6,4,7,12,14
A1B4B1/Pden_2263	5	6	6	5	Uncharacterized protein	Unknown	0
A1B1T8/Pden_1381	5	7	4	6	Invasion associated locus B family protein	Periplasmic	0
A1B8X3/Pden_3901	5	7	4	6	ATPase associated with various cellular activities, AAA_3	Cytoplasmic	0
A1AY33/Pden_0060	5	6	5	6	Orotidine 5'-phosphate decarboxylase	Cytoplasmic	8,1,4,7,12,14
A1B9R0/Pden_4190	5	5	6	6	Carbohydrate ABC transporter substrate-binding protein, CUT1 family	Periplasmic	0
A1AZK1/Pden_0583	5	4	7	6	Ribosomal RNA small subunit methyltransferase H	Cytoplasmic	6,8,4,7,24
A1B5X6/Pden_2836	5	4	7	6	Adenine deaminase (Adenase) (Adenine aminase)	Cytoplasmic	4,8,7,12,14,2
A1B3B3/Pden_1913	5	7	3	7	Tyrosine--tRNA ligase (Tyrosyl-tRNA synthetase) (TyrRS)	Cytoplasmic	8,6,4,7,12,14
A1B0C3/Pden_0855	5	5	5	7	Xanthine phosphoribosyltransferase	Cytoplasmic	8,1,4,7,12,14
A1AZB5/Pden_0497	5	5	4	8	L-threonine dehydratase (Threonine deaminase)	Cytoplasmic	8,1,4,7,12,14
A1B3P1/Pden_2043	5	6	7	5	GcrA cell cycle regulator	Unknown	0
A1B5H7/Pden_2684	5	7	5	6	Uncharacterized protein	Unknown	0
A1B2N0/Pden_1677	5	6	6	6	Xylose isomerase domain protein TIM barrel	Cytoplasmic	0
A1B4X7/Pden_2484	5	6	6	6	Nitric oxide reductase, NorC subunit apoprotein	Membrane	0
A1B446/Pden_2198	5	5	6	7	Pyrroline-5-carboxylate reductase	Cytoplasmic	8,1,4,7,12,14
A1AZV5/Pden_0687	5	4	7	7	OmpA domain protein	OuterMembrane	0
A1B5Q4/Pden_2764	5	7	6	6	Uncharacterized protein	Unknown	0
A1AY30/Pden_0057	5	6	7	6	SPFH domain, Band 7 family protein	Cytoplasmic	0
A1B319/Pden_1816	5	5	8	6	Penicillin-binding protein, 1A family	Membrane	0
A1B4J4/Pden_2348	5	5	8	6	Aminomethyltransferase	Cytoplasmic	4,8,7,12,14,2
A1AZX5/Pden_0707	5	7	5	7	Inositol monophosphatase	Cytoplasmic	4,8,7,12,14,2
A1B2N7/Pden_1684	5	6	6	7	Monosaccharide ABC transporter substrate-binding protein, CUT2 family	Periplasmic	0
A1B4Z7/Pden_2504	5	5	7	7	Uncharacterized protein	Unknown	0
A1B413/Pden_2165	5	5	6	8	Carbamoyl-phosphate synthase small chain	Cytoplasmic	8,1,4,7,12,14

A1B0J9/Pden_0932	5	7	7	6	3-hydroxybutyrate dehydrogenase	Cytoplasmic	0
A1B643/Pden_2903	5	7	7	6	Aminodeoxychorismate lyase	Unknown	0
A1B5H3/Pden_2680	5	6	8	6	Glucokinase	Cytoplasmic	4,8,7,12,14,2
Q9Z463/Pden_4586	5	7	6	7	10 kDa chaperonin (GroES protein, Cpn10)	Unknown	0
A1B4M0/Pden_2374	5	6	7	7	Two component transcriptional regulator, winged helix family	Cytoplasmic	1,6,8,4,7,9
A1AZG3/Pden_0545	5	7	5	8	Phosphoglucosamine mutase	Cytoplasmic	8,7
A1B4J5/Pden_2349	5	6	6	8	Short-chain dehydrogenase/reductase SDR	Cytoplasmic	0
A1B859/Pden_3636	5	6	6	8	OmpW family protein	OuterMembrane	0
A1BAP1/Pden_4521	5	5	7	8	Uncharacterized protein	Unknown	0
A1B5K5/Pden_2715	5	4	8	8	Peptidase M16 domain protein	Unknown	0
A1B1E4/Pden_1233	5	6	10	6	2-dehydro-3-deoxyphosphooctonate aldolase	Cytoplasmic	8,1,4,7,12,14
A1B0D0/Pden_0863	5	8	6	8	PTSINtr with GAF domain, PtsP	Cytoplasmic	5,13
A1B013/Pden_0745	5	6	8	8	50S ribosomal protein L10	Cytoplasmic	1,6,8,4,7,20
A1B467/Pden_2219	5	7	8	8	Uracil phosphoribosyltransferase (UMP pyrophosphorylase) (UPRTase)	Cytoplasmic	8,1,4,7,12,14
Q51701/Pden_2488	5	7	8	8	Uroporphyrinogen-III C-methyltransferase	Cytoplasmic	1,6,4,7,12,14
A1BAD6/Pden_4416	5	8	8	9	Succinate semialdehyde dehydrogenase	Cytoplasmic	0
A1AY11/Pden_0038	5	8	8	10	Thiamine-phosphate diphosphorylase	Cytoplasmic	1,6,4,7,12,14
A1B4B2/Pden_2264	5	8	8	11	Glutathione S-transferase, N-terminal domain	Cytoplasmic	0
A1B2K4/Pden_1651	5	11	11	7	Enoyl-[acyl-carrier-protein] reductase [NADH]	Membrane	8,1,4,7,12,14
A1B925/Pden_3953	5	8	12	9	Tryptophan--tRNA ligase (Tryptophanyl-tRNA synthetase) (TrpRS)	Cytoplasmic	8,6,4,7,12,14
A1AXZ2/Pden_0019	5	10	9	10	S-formylglutathione hydrolase (FGH)	Unknown	4,7,12,14,2
A1B4S6/Pden_2433	5	11	11	12	50S ribosomal protein L28	Cytoplasmic	6,1,8,4,7
A1BAH3/Pden_4453	4	2	0	0	Major facilitator superfamily MFS_1	Membrane	12,5,13,
A1B0B7/Pden_0849	4	0	0	2	Putative outer membrane protein	OuterMembrane	0
A1B4G2/Pden_2315	4	0	1	2	Mammalian cell entry related domain protein	Unknown	0
A1B0L0/Pden_0943	4	2	1	1	Bifunctional protein PutA	Cytoplasmic	4,8,7,12,14,2
A1B385/Pden_1884	4	3	1	1	Imidazole glycerol phosphate synthase subunit HisH (IGP synthase glutamine amidotransferase subunit) (IGP synthase subunit HisH) (ImGP synthase subunit HisH) (IGPS subunit HisH)	Cytoplasmic	8,1,4,7,12,14
A1B9J8/Pden_4128	4	4	2	0	PAS/PAC sensor signal transduction histidine kinase	Membrane	9
A1B0J4/Pden_0927	4	3	2	1	Deoxyguanosinetriphosphate triphosphohydrolase-like protein	Cytoplasmic	8,6,4,7,12,14
A1B3L4/Pden_2016	4	1	2	3	Uncharacterized protein	Membrane	0
A1B9W4/Pden_4244	4	3	2	2	Asparaginase	Unknown	8,6,4,7,12,14
A1BA50/Pden_4330	4	3	4	1	Phosphate ABC transporter substrate-binding protein, PhoT family	Membrane	0
A1BC85/Pden_5069	4	3	3	2	Cystathionine gamma-synthase	Cytoplasmic	0
A1AXY1/Pden_0008	4	2	4	2	Protein GrpE (HSP-70 cofactor)	Cytoplasmic	25
A1B423/Pden_2175	4	4	3	2	Nitroreductase	Cytoplasmic	0
A1B358/Pden_1855	4	3	4	2	ABC transporter related protein	Membrane	0
A1BBQ4/Pden_4888	4	2	5	2	Substrate-binding region of ABC-type glycine betaine transport system	Membrane	0
A1B1J1/Pden_1280	4	3	3	3	Uncharacterized protein	Unknown	0
A1B3V4/Pden_2106	4	3	3	3	ErfK/YbiS/YcfS/YnhG family protein	Unknown	0
A1B473/Pden_2225	4	3	3	3	Propionyl-CoA synthetase	Cytoplasmic	0
A1B5B3/Pden_2620	4	3	3	3	Kynureninase (L-kynurenine hydrolase)	Cytoplasmic	4,8,7,12,14,2
A1BA28/Pden_4308	4	3	3	3	Lipoprotein, YaeC family	Membrane	0

A1B3W7/Pden_2119	4	2	3	4	Glucose-1-phosphate thymidylyltransferase	Cytoplasmic	8,1,4,7,12,14
A1B1U6/Pden_1389	4	2	2	5	Alcohol dehydrogenase, zinc-binding domain	Cytoplasmic	0
A1B1T3/Pden_1376	4	5	2	3	Lipoyl synthase (Lip-syn) (LS)	Cytoplasmic	8,4,7
A1B312/Pden_1809	4	5	2	3	Prephenate dehydratase	Cytoplasmic	8,1,4,7,12,14
A1B1L5/Pden_1304	4	4	3	3	Dipeptidase AC, Metallo peptidase, MEROPS family M19	Cytoplasmic	0
A1B2T2/Pden_1729	4	3	4	3	Alanine dehydrogenase	Cytoplasmic	4,8,7,12,14,2
A1B9L0/Pden_4140	4	2	5	3	Periplasmic solute binding protein	Periplasmic	5,13
A1B652/Pden_2912	4	4	4	3	Replication protein, putative	Cytoplasmic	0
A1B995/Pden_4023	4	4	4	3	Phosphoglycolate phosphatase	Cytoplasmic	1,4,7,12,14
A1B9V2/Pden_4232	4	5	2	4	Peptidylprolyl isomerase	Cytoplasmic	0
A1B683/Pden_2943	4	4	3	4	RND efflux system, outer membrane lipoprotein, NodT family	OuterMembrane	0
A1B876/Pden_3653	4	4	3	4	Cold-shock DNA-binding protein family	Cytoplasmic	9
A1B9K3/Pden_4133	4	4	3	4	FHA domain containing protein	Unknown	0
A1B9Z3/Pden_4273	4	5	4	3	Aminotransferase	Cytoplasmic	0
A1B1X2/Pden_1415	4	4	5	3	Protein-export membrane protein (SecF)	Membrane	12,5,13,23
A1B5G5/Pden_2672	4	4	5	3	Aminotransferase	Cytoplasmic	1
A1B3D7/Pden_1938	4	4	4	4	Cytochrome c oxidase subunit 1	Membrane	4,12,14
A1B478/Pden_2230	4	4	4	4	Biotin--acetyl-CoA-carboxylase ligase	Cytoplasmic	8,4,7
A1B5A2/Pden_2609	4	4	4	4	3'-5' exonuclease	Cytoplasmic	0
A1B8K6/Pden_3784	4	4	4	4	Cytidylate kinase (CK) (Cytidine monophosphate kinase) (CMP kinase)	Cytoplasmic	8,6,4,7,12,14
A1B1E5/Pden_1234	4	4	3	5	Capsule polysaccharide export protein-like protein	Unknown	0
A1BAC4/Pden_4404	4	4	3	5	Uncharacterized protein UPF0065	Unknown	0
A1B0H4/Pden_0907	4	2	5	5	Sec-independent protein translocase (TatA)	Membrane	12,5,13,23
A1B9J3/Pden_4123	4	2	5	5	GTPase HflX (GTP-binding protein HflX)	Cytoplasmic	0
A1B384/Pden_1883	4	6	4	3	Imidazoleglycerol-phosphate dehydratase (IGPD)	Cytoplasmic	8,1,4,7,12,14
A1B0H7/Pden_0910	4	6	3	4	Uncharacterized protein	Cytoplasmic	0
A1B464/Pden_2216	4	5	4	4	RNA polymerase sigma factor RpoH (RNA polymerase sigma-32 factor)	Cytoplasmic	7,4,12,31,27,16
A1B4Y7/Pden_2494	4	5	4	4	Radical SAM domain protein	Cytoplasmic	0
A1B553/Pden_2560	4	5	4	4	Enoyl-CoA hydratase/isomerase	Cytoplasmic	0
A1B3E3/Pden_1944	4	4	5	4	Peptidyl-dipeptidase Dcp, Metallo peptidase, MEROPS family M03A	Cytoplasmic	0
A1BBD2/Pden_4765	4	4	5	4	C4-dicarboxylate transport protein	Membrane	5,13
A1B2W6/Pden_1763	4	5	3	5	Nucleoside ABC transporter ATP-binding	Membrane	0
A1B4I0/Pden_2333	4	5	3	5	Uncharacterized protein	Cytoplasmic	0
A1B7P7/Pden_3470	4	4	4	5	Uncharacterized protein	Unknown	0
A1BA70/Pden_4350	4	3	5	5	Periplasmic glucan biosynthesis protein (MdoG)	Periplasmic	1,8,7,14
A1B4D2/Pden_2285	4	3	4	6	Substrate-binding region of ABC-type glycine betaine transport system	Periplasmic	5,13
A1B987/Pden_4015	4	3	4	6	Linocin_M18 bacteriocin protein	Unknown	11,29,28
A1BAH8/Pden_4458	4	2	5	6	RNA binding S1 domain protein	Cytoplasmic	6,8,4,7
A1AZK8/Pden_0590	4	3	3	7	Membrane protein involved in aromatic hydrocarbon degradation	OuterMembrane	0
A1B0B4/Pden_0846	4	6	5	3	Lipopolysaccharide biosynthesis	Membrane	8,1,4,7,12,14
A1B3V1/Pden_2103	4	6	5	3	Ureidoglycolate lyase	Cytoplasmic	4,8,7,12,14,2
A1B1G6/Pden_1255	4	6	4	4	Short-chain dehydrogenase/reductase SDR	Cytoplasmic	0
A1AXY3/Pden_0010	4	5	5	4	Ribonuclease PH (RNase PH) (tRNA nucleotidyltransferase)	Cytoplasmic	6,8,4,7

A1B2N6/Pden_1683	4	5	4	5	Putative ribose/galactose/methyl galactoside import ATP-binding protein	Membrane	0
A1B5K6/Pden_2716	4	5	4	5	Uncharacterized protein	Unknown	0
A1B134/Pden_1120	4	4	5	5	Methylenetetrahydromethanopterin reductase	Cytoplasmic	0
A1B9B0/Pden_4038	4	4	5	5	Mannose-6-phosphate isomerase, type 2	Cytoplasmic	1,8,7,14
A1AZD3/Pden_0515	4	5	3	6	Sarcosine oxidase, alpha subunit family	Cytoplasmic	8,6,4,7,12,14
A1BAH0/Pden_4450	4	4	4	6	Formate/nitrite transporter	Membrane	0
A1BB25/Pden_4658	4	3	5	6	Extracellular solute-binding protein, family 1	Unknown	0
A1B4L7/Pden_2371	4	7	5	3	Thioredoxin domain	Cytoplasmic	7,12,14,9,10
A1AZP4/Pden_0626	4	6	5	4	OmpA/MotB domain protein	Membrane	0
A1AZZ5/Pden_0727	4	6	5	4	Outer membrane transport energization protein (ExbB)	Membrane	0
A1B1F8/Pden_1247	4	6	5	4	Glutathionylspermidine synthase	Cytoplasmic	0
A1B9B9/Pden_4047	4	6	5	4	NAD(P) transhydrogenase subunit beta	Membrane	0
A1B5K4/Pden_2714	4	5	6	4	Peptidase M16 domain protein	Unknown	0
A1B0G1/Pden_0894	4	6	4	5	Peptide chain release factor 1 (RF-1)	Cytoplasmic	0
A1B444/Pden_2196	4	6	3	6	D-isomer specific 2-hydroxyacid dehydrogenase, NAD-binding protein	Cytoplasmic	0
A1B4F4/Pden_2307	4	4	5	6	Cytochrome c_1	Unknown	0
A1B9T8/Pden_4218	4	6	7	3	Periplasmic copper-binding protein (NosD)	Periplasmic	0
A1B8N5/Pden_3813	4	4	8	4	Hydroxyacylglutathione hydrolase (Glyoxalase II) (Glx II)	Cytoplasmic	4,7,12,14,2
A1B5J7/Pden_2707	4	6	5	5	Leucyl aminopeptidase	Cytoplasmic	0
A1B902/Pden_3930	4	5	6	5	Amino acid/amide ABC transporter ATP-binding protein 2, HAAT family	Unknown	0
A1BAA7/Pden_4387	4	5	6	5	Carbohydrate ABC transporter substrate-binding protein, CUT1 family	Periplasmic	0
A1BAG3/Pden_4443	4	5	5	6	Electron transport protein SCO1/SenC	Unknown	0
A1B573/Pden_2580	4	2	7	7	Allergen V5/Tpx-1 family protein	Unknown	0
A1B0A6/Pden_0838	4	7	6	4	Amino acid adenylation domain	Membrane	1
A1B3T8/Pden_2090	4	7	5	5	Uncharacterized protein	Cytoplasmic	0
A1B2K8/Pden_1655	4	4	7	6	Elongation factor P (EF-P)	Cytoplasmic	0
A1B3I5/Pden_1987	4	5	5	7	Acetyl-coenzyme A carboxylase carboxyl transferase subunit beta	Cytoplasmic	8,1,4,7,12,14
A1AZE6/Pden_0528	4	6	5	7	Nucleoside-binding protein	Unknown	0
A1AZE9/Pden_0531	4	6	5	7	Nitrogen-fixing NifU domain protein	Unknown	1,3
A1B1M0/Pden_1309	4	6	5	7	ETC complex I subunit conserved region	Unknown	4,12,14
A1B0F9/Pden_0892	4	4	7	7	YceI family protein	Unknown	0
A1AZ42/Pden_0422	4	6	4	8	Pyridoxine 5'-phosphate synthase (PNP synthase)	Cytoplasmic	1,6,4,7,12,14
A1B547/Pden_2554	4	6	7	6	Dihydroorotase (DHOase)	Cytoplasmic	8,1,4,7,12,14
A1B8P1/Pden_3819	4	6	7	6	ATP synthase epsilon chain	Cytoplasmic	7,4,12,14,5,13
A1BAL0/Pden_4490	4	8	4	7	D-alanine--D-alanine ligase	Cytoplasmic	12,7,14,9,10,19
A1B3H7/Pden_1979	4	9	5	6	Methionine aminopeptidase (MAP) (MetAP) (Peptidase M)	Cytoplasmic	8,4,7
A1AZY8/Pden_0720	4	5	9	6	NADH:flavin oxidoreductase/NADH oxidase	Cytoplasmic	0
A1B5T0/Pden_2790	4	7	6	7	ATP-dependent protease ATPase subunit HslU (Unfoldase HslU)	Cytoplasmic	26
A1AZG5/Pden_0547	4	6	6	8	RNA polymerase-binding transcription factor (DksA)	Cytoplasmic	9
A1B080/Pden_0812	4	5	7	8	Phosphoserine phosphatase	Cytoplasmic	8,1,4,7,12,14
A1B3N1/Pden_2033	4	5	7	8	Penicillin-binding protein, 1A family	Membrane	0
A1BAI8/Pden_4468	4	7	8	6	Orotate phosphoribosyltransferase	Cytoplasmic	8,1,4,7,12,14
A1AZK5/Pden_0587	4	6	9	6	UDP-N-acetylmuramoyl-tripeptide--D-alanyl-D-alanine ligase	Cytoplasmic	12,7,14,9,10,19

A1BAJ7/Pden_4477	4	7	7	7	Putative signal-transduction protein with CBS domains	Cytoplasmic	0
A1BB80/Pden_4713	4	7	6	8	Probable septum site-determining (MinC)	Cytoplasmic	3,12,9,31,17
A1B5T8/Pden_2798	4	5	8	8	4-hydroxy-tetrahydrodipicolinate reductase (HTPA reductase)	Cytoplasmic	8,1,4,7,12,14
A1B441/Pden_2193	4	4	8	9	Peptidase M24	Cytoplasmic	0
A1AY25/Pden_0052	4	7	7	8	Amino acid/amide ABC transporter substrate-binding protein, HAAT family	Unknown	0
A1B9P3/Pden_4173	4	6	9	8	TonB-dependent receptor	OuterMembrane	5
A1B3Y0/Pden_2132	4	9	5	9	Anthranilate synthase, component I	Cytoplasmic	8,1,4,7,12,14
A1B040/Pden_0772	4	8	6	9	30S ribosomal protein S14	Cytoplasmic	6,1,8,4,7
A1B9T7/Pden_4217	4	9	8	8	ABC transporter related protein	Cytoplasmic	0
A1B3I2/Pden_1984	4	7	7	11	Dihydroxy-acid dehydratase (DAD)	Cytoplasmic	8,1,4,7,12,14
A1B350/Pden_1847	4	7	9	10	Cytochrome *c* oxidase, *cbb*$_3$-type, subunit II	Cytoplasmic	4,12,14
A1BAI1/Pden_4461	4	7	8	13	Nitrogen regulatory protein P-II (GlnB)	Membrane	1,6,8,4,7,9
A1B031/Pden_0763	4	11	10	10	30S ribosomal protein S19	Cytoplasmic	6,1,8,4,7
A1B647/Pden_2907	3	0	0	1	Acetyl-CoA acetyltransferase	Cytoplasmic	0
A1AZN2/Pden_0614	3	1	1	1	S-adenosylmethionine:tRNA ribosyltransferase-isomerase	Cytoplasmic	8,1,4,7,12,14
A1B395/Pden_1894	3	1	1	1	Uncharacterized protein	Unknown	0
A1B557/Pden_2564	3	1	1	1	Amino acid adenylation domain	Cytoplasmic	1
A1B465/Pden_2217	3	2	1	1	Uncharacterized protein	Cytoplasmic	0
A1B4E3/Pden_2296	3	1	2	1	UDP-galactose 4-epimerase	Cytoplasmic	8,7,14
A1BBN3/Pden_4867	3	2	0	2	Dihydrodipicolinate synthase	Cytoplasmic	0
A1B3Z3/Pden_2145	3	0	2	2	DNA polymerase III, delta prime subunit	Cytoplasmic	0
A1B8V1/Pden_3879	3	0	2	2	GCN5-related N-acetyltransferase	Unknown	0
A1BAD2/Pden_4412	3	0	1	3	Acyl-CoA dehydrogenase domain protein	Cytoplasmic	0
A1BCB9/Pden_5103	3	2	2	1	Poly-beta-hydroxybutyrate polymerase domain protein	Cytoplasmic	1,7
A1B0C2/Pden_0854	3	1	3	1	MORN repeat-containing protein	Periplasmic	0
A1B3A0/Pden_1899	3	1	3	1	Substrate-binding region of ABC-type glycine betaine transport system	Unknown	5,13
A1B810/Pden_3586	3	1	3	1	Transglutaminase domain protein	Unknown	0
A1B2N2/Pden_1679	3	3	0	2	Xylose isomerase domain protein TIM barrel	Cytoplasmic	0
A1B868/Pden_3645	3	2	1	2	Uncharacterized protein	Unknown	0
A1B992/Pden_4020	3	2	1	2	ABC transporter related protein	Membrane	0
A1B347/Pden_1844	3	1	2	2	4Fe-4S ferredoxin, iron-sulfur binding domain	Membrane	0
A1B812/Pden_3588	3	1	2	2	Amidohydrolase	Cytoplasmic	0
A1AZC8/Pden_0510	3	1	1	3	Putative pre-16S rRNA nuclease	Cytoplasmic	6,8,4,7
A1B3J7/Pden_1999	3	4	2	0	Glycerol-3-phosphate dehydrogenase	Unknown	4,8,7,12,14,2
A1B8X7/Pden_3905	3	4	2	0	Uncharacterized protein	Membrane	0
A1B481/Pden_2233	3	3	2	1	NADH dehydrogenase subunit L	Membrane	4,12,14
A1BAY8/Pden_4619	3	3	2	1	TRAP dicarboxylate transporter-DctP subunit	Periplasmic	5
A1B8L1/Pden_3789	3	2	3	1	N-(5'-phosphoribosyl)anthranilate isomerase (PRAI)	Unknown	8,1,4,7,12,14
A1AZF7/Pden_0539	3	4	0	2	Signal transduction histidine kinase regulating citrate/malate metabolism	Unknown	0
A1B718/Pden_3231	3	3	1	2	Putative transcriptional regulator, IclR family	Cytoplasmic	1,6,8,4,7,9
A1B1K5/Pden_1294	3	2	2	2	Uncharacterized protein	Cytoplasmic	0
A1BB09/Pden_4642	3	2	2	2	Uncharacterized protein	Unknown	0
A1B5X9/Pden_2839	3	1	2	3	Uncharacterized protein	Unknown	0

A1B9I1/Pden_4109	3	3	3	1	Putative sulfonate/nitrate transport system substrate-binding protein	Cytoplasmic	0
A1BA15/Pden_4295	3	3	2	2	DNA helicase	Cytoplasmic	0
A1BAI9/Pden_4469	3	3	2	2	Replicative DNA helicase	Cytoplasmic	6,1,8,4,7
A1AZS5/Pden_0657	3	2	3	2	Uncharacterized protein	Unknown	0
A1AZV3/Pden_0685	3	2	3	2	Cell division and transport-associated protein TolA	OuterMembrane	12
A1B0M3/Pden_0956	3	2	3	2	Uncharacterized protein	Cytoplasmic	0
A1B0J6/Pden_0929	3	3	1	3	Exodeoxyribonuclease III	Cytoplasmic	4,8,7,12,14,13
A1B396/Pden_1895	3	1	3	3	Uncharacterized protein	Cytoplasmic	0
A1B9C4/Pden_4052	3	2	1	4	Mucin-associated surface protein	Unknown	0
A1BAF0/Pden_4430	3	2	1	4	Nicotinate-nucleotide pyrophosphorylase (Carboxylating)	Cytoplasmic	8,1,4,7,12,14
A1B093/Pden_0825	3	4	3	1	Transglutaminase, N-terminal domain protein	Cytoplasmic	0
A1B9X6/Pden_4256	3	4	2	2	Transcriptional regulator, LysR family	Cytoplasmic	6,1,8,4,7
A1BAR5/Pden_4546	3	4	2	2	TRAP dicarboxylate transporter-DctP subunit	Unknown	5
A1AZJ5/Pden_0577	3	3	3	2	Uncharacterized protein	Unknown	0
A1B0K8/Pden_0941	3	3	3	2	Malate/L-lactate dehydrogenase	Cytoplasmic	0
A1B1F7/Pden_1246	3	3	3	2	Uncharacterized protein	Unknown	0
Q51687/Pden_1409	3	3	3	2	Histidinol-phosphate aminotransferase	Cytoplasmic	8,1,4,7,12,14
A1B284/Pden_1528	3	3	3	2	Uncharacterized protein	Cytoplasmic	0
A1B9N9/Pden_4169	3	3	3	2	Putative sulfonate/nitrate transport system substrate-binding protein	Unknown	0
A1BAK2/Pden_4482	3	3	3	2	3-hydroxyisobutyrate dehydrogenase (HIBADH)	Cytoplasmic	4,8,7,12,14,2
A1B450/Pden_2202	3	3	2	3	N5-carboxyaminoimidazole ribonucleotide mutase	Unknown	8,1,4,7,12,14
A1B475/Pden_2227	3	3	2	3	tRNA (guanine-N(7)-)-methyltransferase	Cytoplasmic	0
A1B4Q5/Pden_2411	3	3	2	3	Outer membrane efflux protein	Cytoplasmic	0
A1BAF1/Pden_4431	3	2	2	4	L-aspartate oxidase	Cytoplasmic	0
A1B993/Pden_4021	3	1	3	4	Amino acid/amide ABC transporter ATP-binding protein 2, HAAT family	Membrane	0
A1B542/Pden_2549	3	2	1	5	ATP:cob(I)alamin adenosyltransferase	Cytoplasmic	0
A1B696/Pden_2956	3	1	2	5	Carboxynorspermidine/carboxyspermidine decarboxylase	Cytoplasmic	6,1,4,7
A1B856/Pden_3633	3	4	4	1	Isovaleryl-CoA dehydrogenase	Cytoplasmic	0
A1AZV1/Pden_0683	3	4	3	2	Cell division and transport-associated protein TolQ	Membrane	12
A1B443/Pden_2195	3	4	3	2	Hydroxypyruvate reductase	Cytoplasmic	0
A1B0C7/Pden_0859	3	3	4	2	Cold-shock DNA-binding protein family	Cytoplasmic	9
A1B3Y9/Pden_2141	3	3	4	2	Molybdopterin molybdochelatase	Cytoplasmic	6,1,4,7
A1B5L0/Pden_2720	3	3	4	2	Fmu (Sun) domain protein	Cytoplasmic	9
A1B5V3/Pden_2813	3	4	2	3	Shikimate dehydrogenase (NADP(+)) (SDH)	Cytoplasmic	8,1,4,7,12,14
A1B2V5/Pden_1752	3	2	4	3	Coproporphyrinogen oxidase	Cytoplasmic	6,1,4,7
A1BAK4/Pden_4484	3	3	2	4	DNA repair protein RecN (Recombination protein N)	Cytoplasmic	4,8,7,12,14,13
A1B1I3/Pden_1272	3	2	3	4	Biotin carboxyl carrier protein	Unknown	8,1,4,7,12,14
A1B5B6/Pden_2623	3	2	2	5	Uncharacterized protein	Unknown	0
A1B1R3/Pden_1355	3	4	4	2	Methionine synthase (B12-independent)	Cytoplasmic	8,1,4,7,12,14
A1B1U4/Pden_1387	3	4	4	2	Inositol monophosphatase	Cytoplasmic	8,4,7,12,14
A1BA98/Pden_4378	3	5	2	3	Ribonucleoside-diphosphate reductase subunit beta	Cytoplasmic	8,1,4,7,12,14
A1B3H8/Pden_1980	3	4	3	3	Molybdopterin binding domain	Cytoplasmic	0
A1B3Y4/Pden_2136	3	4	3	3	Anthranilate synthase, component II	Cytoplasmic	0

A1B8K9/Pden_3787	3	4	3	3	Integration host factor subunit beta (IHF-beta)	Cytoplasmic	1,6,8,4,7,9
A1B1H0/Pden_1259	3	3	4	3	Periplasmic solute binding protein	Periplasmic	18,5,13
A1B488/Pden_2240	3	3	3	4	Uncharacterized protein	Unknown	0
A1B9H4/Pden_4102	3	3	3	4	50S ribosomal protein L27	Cytoplasmic	6,1,8,4,7
A1BAJ6/Pden_4476	3	2	4	4	Phosphopantetheine adenylyltransferase	Cytoplasmic	8,1,4,7,12,14
A1B368/Pden_1865	3	2	2	6	2-deoxycytidine 5-triphosphate deaminase	Cytoplasmic	8,6,4,7,12,14
A1B333/Pden_1830	3	5	3	3	Sterol-binding domain protein	Unknown	0
A1BAE1/Pden_4421	3	5	3	3	Phosphonate metabolism protein PhnM	Cytoplasmic	4,7,2
A1B5P3/Pden_2753	3	4	4	3	5-carboxymethyl-2-hydroxymuconate delta-isomerase	Cytoplasmic	0
A1B4Q4/Pden_2410	3	5	2	4	Uncharacterized protein	Cytoplasmic	0
A1AY07/Pden_0034	3	4	3	4	ABC transporter related protein	Membrane	0
A1B3Z4/Pden_2146	3	4	3	4	Hydrolase, TatD family	Cytoplasmic	0
A1B699/Pden_2959	3	4	3	4	Uncharacterized protein	Unknown	0
A1BAR8/Pden_4549	3	4	3	4	Iron-containing alcohol dehydrogenase	Cytoplasmic	0
A1B5J5/Pden_2705	3	4	2	5	Carbonic anhydrase (Carbonate dehydratase)	Cytoplasmic	15
A1B511/Pden_2518	3	1	5	5	Iojap-like protein	Cytoplasmic	0
A1B841/Pden_3618	3	2	3	6	Glutathione S-transferase, C-terminal domain	Cytoplasmic	0
A1B891/Pden_3668	3	6	3	3	Ribose-5-phosphate isomerase A (Phosphoriboisomerase A) (PRI)	Cytoplasmic	4,7,12,14
A1B4Q0/Pden_2406	3	5	3	4	D-alanine--D-alanine ligase (D-Ala-D-Ala ligase) (D-alanylalanine synthetase)	Cytoplasmic	12,7,14,9,10,19
A1B9E3/Pden_4071	3	5	3	4	NADPH-dependent FMN reductase	Cytoplasmic	0
Q51703/Pden_2491	3	4	4	4	Protein NirD	Cytoplasmic	9
A1AZ20/Pden_0400	3	3	5	4	1-deoxy-D-xylulose-5-phosphate synthase	Cytoplasmic	8,1,4,7,12,14
A1B9E7/Pden_4075	3	3	5	4	L-lactate dehydrogenase (Cytochrome)	Cytoplasmic	0
A1B4R8/Pden_2425	3	2	6	4	Uncharacterized protein	Membrane	0
A1B426/Pden_2178	3	3	3	6	Methylmalonyl-CoA epimerase	Cytoplasmic	0
A1AZL7/Pden_0599	3	5	4	4	Uncharacterized protein	Unknown	0
A1B8P4/Pden_3822	3	5	4	4	DSBA oxidoreductase	Cytoplasmic	0
A1B2M9/Pden_1676	3	4	5	4	5-dehydro-2-deoxygluconokinase	Cytoplasmic	0
A1BA34/Pden_4314	3	3	6	4	Peptidase M16 domain protein	Cytoplasmic	0
A1B5M6/Pden_2736	3	5	3	5	Endoribonuclease L-PSP	Unknown	0
A1BAN3/Pden_4513	3	5	3	5	Short chain enoyl-CoA hydratase	Cytoplasmic	0
A1B0G3/Pden_0896	3	4	4	5	Uncharacterized protein	Unknown	0
A1B1B8/Pden_1207	3	4	4	5	Urease accessory protein (UreE)	Cytoplasmic	3,8,4,7,12,14
A1B1U9/Pden_1392	3	4	4	5	Glutaredoxin	Unknown	12,9,10
A1B8C5/Pden_3702	3	4	4	5	Glutamate--putrescine ligase	Cytoplasmic	8,1,4,7,12,14
A1B8Y2/Pden_3910	3	4	4	5	Endoribonuclease L-PSP	Cytoplasmic	0
A1B2A9/Pden_1553	3	2	5	6	N utilization substance protein B homolog	Unknown	1,6,8,4,7,9
A1B8L7/Pden_3795	3	3	3	7	Polysaccharide deacetylase	Cytoplasmic	6,1,8,4,7
A1B4E6/Pden_2299	3	6	5	3	3-deoxy-manno-octulosonate cytidylyltransferase	Cytoplasmic	0
A1B126/Pden_1112	3	5	5	4	Dihydropyrimidinase	Cytoplasmic	0
A1B8N1/Pden_3809	3	5	5	4	Delta-aminolevulinic acid dehydratase	Cytoplasmic	6,1,4,7
A1B8X9/Pden_3907	3	3	7	4	Acyl-[acyl-carrier-protein]--UDP-N-acetylglucosamine O-acyltransferase	Cytoplasmic	8,1,4,7,12,14
A1B3C1/Pden_1921	3	5	4	5	Signal recognition particle receptor FtsY	Cytoplasmic	12,3,5,13,23,22
A1B1V8/Pden_1401	3	4	5	5	DNA-directed RNA polymerase subunit omega (RNAP omega subunit)	Cytoplasmic	6,1,8,4,7

A1B133/Pden_1119	3	6	2	6	Reduced coenzyme F420:NADP oxidoreductase	Unknown	8,6,4,7,12,14
A1B602/Pden_2862	3	4	4	6	Uncharacterized protein	Cytoplasmic	0
A1B095/Pden_0827	3	3	5	6	DNA mismatch repair protein (MutS)	Cytoplasmic	4,8,7,12,14,13
A1B0C0/Pden_0852	3	9	3	3	Nicotinate phosphoribosyltransferase (NAPRTase)	Cytoplasmic	8,1,4,7,12,14
A1B0E7/Pden_0880	3	6	5	4	Acetylglutamate kinase	Cytoplasmic	8,1,4,7,12,14
A1AZX9/Pden_0711	3	4	6	5	Histidine ammonia-lyase (Histidase)	Cytoplasmic	4,8,7,12,14,2
A1B2K2/Pden_1649	3	5	4	6	3-hydroxydecanoyl-[acyl-carrier-protein] dehydratase	Cytoplasmic	8,1,4,7,12,14
A1AY60/Pden_0087	3	4	4	7	Acylneuraminate cytidylyltransferase	Cytoplasmic	0
A1B9D4/Pden_4062	3	4	4	7	2-isopropylmalate synthase	Cytoplasmic	8,1,4,7,12,14
A1B2X0/Pden_1767	3	3	5	7	Ferritin, Dps family protein	Cytoplasmic	11,12,10
A1B5M8/Pden_2738	3	6	8	2	Chromosome partition protein Smc	Cytoplasmic	8,1,3,4,7,12
A1B612/Pden_2872	3	8	5	3	PfkB domain protein	Cytoplasmic	0
A1BB51/Pden_4684	3	7	5	4	Butyryl-CoA:acetate CoA transferase	Cytoplasmic	0
A1B686/Pden_2946	3	6	5	5	PfkB domain protein	Unknown	0
A1AY10/Pden_0037	3	6	4	6	UBA/THIF-type NAD/FAD binding protein	Membrane	0
A1B3I8/Pden_1990	3	4	6	6	Uncharacterized protein	Unknown	0
A1B0M7/Pden_0960	3	4	5	7	Polyhydroxyalkonate synthesis repressor, PhaR	Cytoplasmic	0
A1B8U5/Pden_3873	3	5	6	6	Crotonyl-CoA reductase	Cytoplasmic	0
A1B0M1/Pden_0954	3	7	3	7	Cold-shock DNA-binding protein family	Cytoplasmic	1,6,8,4,7,9
A1B3B6/Pden_1916	3	5	7	6	Peptidyl-prolyl cis-trans isomerase (PPIase)	Unknown	25
A1AYV3/Pden_0333	3	6	5	7	Tryptophan synthase alpha chain	Cytoplasmic	0
A1B8B1/Pden_3688	3	5	6	7	Propionyl-CoA carboxylase carboxyltransferase subunit	Cytoplasmic	0
A1B2B8/Pden_1562	3	4	7	7	Transcriptional repressor (NrdR)	Cytoplasmic	1,6,8,4,7,9
A1B935/Pden_3963	3	5	6	8	Cysteine desulfurase	Unknown	0
A1B2V1/Pden_1748	3	6	7	7	MltA-interacting MipA family protein	Unknown	0
A1B903/Pden_3931	3	10	6	6	Amino acid/amide ABC transporter ATP-binding protein 1, HAAT family	Membrane	0
A1B6A0/Pden_2960	2	0	0	0	Metal dependent phosphohydrolase	Cytoplasmic	0
A1B9S8/Pden_4208	2	0	0	0	Acetate kinase (Acetokinase)	Cytoplasmic	1,4,7,12,14
A1BA62/Pden_4342	2	0	0	0	Sulfate ABC transporter, inner membrane subunit CysW	Membrane	0
A1BC46/Pden_5030	2	0	0	0	Uncharacterized protein	Cytoplasmic	0
A1AZE1/Pden_0523	2	1	0	0	tRNA 2-selenouridine synthase	Unknown	6,8,4,7
A1B3U9/Pden_2101	2	1	0	0	Methyltransferase	Cytoplasmic	6,8,4,7,24
A1B9E2/Pden_4070	2	0	1	0	Uncharacterized protein	Cytoplasmic	0
A1BAW3/Pden_4594	2	0	1	0	Thiamine pyrophosphate enzyme domain protein TPP-binding protein	Cytoplasmic	0
A1B1F4/Pden_1243	2	0	0	1	3-oxoacyl-[acyl-carrier-protein] synthase II	Membrane	0
A1B3X0/Pden_2122	2	0	0	1	dTDP-4-dehydrorhamnose 3,5-epimerase	Cytoplasmic	0
A1BAH4/Pden_4454	2	2	0	0	Putative nitrate transport protein	Membrane	0
A1B1V5/Pden_1398	2	1	1	0	Gamma-glutamylputrescine oxidase	Cytoplasmic	0
A1B2T7/Pden_1734	2	1	1	0	Uncharacterized protein	Periplasmic	0
A1B479/Pden_2231	2	1	1	0	NADH-quinone oxidoreductase subunit N	Membrane	4,12,14
A1BB73/Pden_4706	2	0	2	0	Beta-lactamase domain protein	Cytoplasmic	0
A1AYL3/Pden_0243	2	1	0	1	Succinate semialdehyde dehydrogenase	Cytoplasmic	0
A1B1W0/Pden_1403	2	1	0	1	Uncharacterized protein	Cytoplasmic	0
A1B970/Pden_3998	2	1	0	1	Zinc metalloprotease	Membrane	0

A1B8T2/Pden_3860	2	0	1	1	NUDIX hydrolase	Cytoplasmic	0
A1B9B4/Pden_4042	2	0	1	1	Uncharacterized protein	Cytoplasmic	0
A1BAE7/Pden_4427	2	0	1	1	Glucose-1-phosphate adenylyltransferase	Cytoplasmic	8,1,4,7,12,14
A1BBI9/Pden_4822	2	0	1	1	Short-chain dehydrogenase/reductase SDR	Cytoplasmic	0
A1B045/Pden_0777	2	0	0	2	50S ribosomal protein L30	Unknown	6,1,8,4,7
A1B2Z5/Pden_1792	2	1	2	0	Ribonuclease	Cytoplasmic	0
A1B9E5/Pden_4073	2	1	2	0	DNA primase	Cytoplasmic	0
A1B1H5/Pden_1264	2	2	0	1	Peptidyl-tRNA hydrolase (PTH)	Cytoplasmic	6,1,8,4,7
A1B3H9/Pden_1981	2	2	0	1	GCN5-related N-acetyltransferase	Periplasmic	0
A1B8L4/Pden_3792	2	2	0	1	DSBA oxidoreductase	Unknown	0
A1B1U3/Pden_1386	2	1	1	1	Magnesium transporter (MgtE)	Membrane	5,13
A1B2B3/Pden_1557	2	1	1	1	Riboflavin synthase, alpha subunit	Cytoplasmic	1,6,4,7,12,14
A1B2X5/Pden_1772	2	1	1	1	Beta-lactamase domain protein	Unknown	0
A1B320/Pden_1817	2	1	1	1	N-acetylmuramoyl-L-alanine amidase	Unknown	6,7,2
A1B3C4/Pden_1924	2	1	1	1	ATP phosphoribosyltransferase regulatory subunit	Cytoplasmic	0
A1B8U3/Pden_3871	2	1	1	1	Uncharacterized protein	Cytoplasmic	0
A1B9A0/Pden_4028	2	1	1	1	Exodeoxyribonuclease 7 large subunit	Cytoplasmic	6,8,4,7,2
A1BBJ1/Pden_4824	2	1	1	1	Phosphoglucomutase/phosphomannomutase alpha/beta/alpha domain I	Cytoplasmic	8,7
A1B1A8/Pden_1197	2	0	2	1	NADH:flavin oxidoreductase/NADH oxidase	Cytoplasmic	0
A1B2N3/Pden_1680	2	0	2	1	Oxidoreductase domain protein	Unknown	0
A1B2V8/Pden_1755	2	0	2	1	16S rRNA m(2)G 1207 methyltransferase	Cytoplasmic	0
A1B0L9/Pden_0952	2	0	1	2	DSBA oxidoreductase	Cytoplasmic	0
A1B8C1/Pden_3698	2	0	1	2	tRNA (cytidine/uridine-2'-O-)-methyltransferase (TrmJ)	Cytoplasmic	6,8,4,7
A1B1M6/Pden_1315	2	3	1	0	Glycosyl transferase, family 2	Membrane	0
A1BAW1/Pden_4592	2	3	1	0	Alkyl hydroperoxide reductase (AhpD)	Unknown	0
A1BAE8/Pden_4428	2	2	2	0	1,4-alpha-glucan branching enzyme (GlgB)	Cytoplasmic	8,1,4,7,12,14
A1B1E8/Pden_1237	2	3	0	1	Uncharacterized protein	Unknown	0
A1AZN5/Pden_0617	2	2	1	1	HAD-superfamily hydrolase, subfamily IA	Cytoplasmic	0
A1AZZ1/Pden_0723	2	2	1	1	Uncharacterized protein	Membrane	0
A1B0E9/Pden_0882	2	2	1	1	Phosphofructokinase	Cytoplasmic	8,7
A1B4X6/Pden_2483	2	2	1	1	Nitric oxide reductase, NorB subunit apoprotein	Membrane	4,12,14
A1B653/Pden_2913	2	2	1	1	Cobyrinic acid a,c-diamide synthase	Cytoplasmic	0
A1AZ24/Pden_0404	2	1	2	1	AMP nucleosidase	Cytoplasmic	8,1,4,7,12,14
A1B3D6/Pden_1937	2	1	2	1	Cytochrome c, class I	Periplasmic	0
A1B5Y9/Pden_2849	2	1	2	1	PTS system fructose subfamily IIA component	Unknown	5,13
A1B8W6/Pden_3894	2	1	2	1	Acetylornithine deacetylase or succinyl-diaminopimelate desuccinylase	Cytoplasmic	0
A1AYV0/Pden_0330	2	2	0	2	NADPH-dependent FMN reductase	Cytoplasmic	0
A1B062/Pden_0794	2	2	0	2	L-asparagine ABC transporter membrane protein / L-glutamine ABC transporter membrane protein / L-glutamate ABC transporter membrane protein / L-aspartate ABC transporter membrane protein	Membrane	0
A1AXY2/Pden_0009	2	1	1	2	Heat-inducible transcription repressor (HrcA)	Membrane	1,6,8,4,7,9
A1AZ64/Pden_0444	2	1	1	2	Uncharacterized protein	Cytoplasmic	0
A1B0X5/Pden_1060	2	1	1	2	Glyceraldehyde-3-phosphate dehydrogenase, type I	Cytoplasmic	0
A1B7P9/Pden_3472	2	1	1	2	Uncharacterized protein	Cytoplasmic	0

A1BB12/Pden_4645	2	1	1	2	Transcriptional regulator, TetR family	Cytoplasmic	1,6,8,4,7,9
A1BBC6/Pden_4759	2	1	1	2	Dihydrolipoamide acetyltransferase component of pyruvate dehydrogenase complex	Cytoplasmic	0
A1BC45/Pden_5029	2	1	1	2	Uncharacterized protein	Cytoplasmic	0
A1B001/Pden_0733	2	0	2	2	Uncharacterized protein	Unknown	0
A1B624/Pden_2884	2	0	2	2	Dihydroorotate dehydrogenase (quinone)	Membrane	8,1,4,7,12,14
A1BB46/Pden_4679	2	0	2	2	6,7-dimethyl-8-ribityllumazine synthase	Cytoplasmic	1,6,4,7,12,14
A1B5W0/Pden_2820	2	1	0	3	RNA polymerase sigma factor	Cytoplasmic	6,1,8,4,7
A1AZC3/Pden_0505	2	3	1	1	Mannose-binding protein / fructose-binding protein / ribose-binding protein	Periplasmic	0
A1B3Z2/Pden_2144	2	2	2	1	Thymidylate kinase (dTMP kinase)	Cytoplasmic	8,1,4,7,12,14
A1B598/Pden_2605	2	2	2	1	ABC transporter related protein	Cytoplasmic	12,5,13
A1B9V4/Pden_4234	2	2	2	1	Respiratory nitrate reductase chaperone (NarJ)	Cytoplasmic	3
A1B5P2/Pden_2752	2	2	1	2	Transcriptional regulator, GntR family	Cytoplasmic	6,1,8,4,7
A1B8C9/Pden_3706	2	2	1	2	Amino acid ABC transporter ATP-binding protein, PAAT family	Membrane	0
A1BAM0/Pden_4500	2	2	1	2	Uncharacterized protein	Cytoplasmic	0
A1AZ38/Pden_0418	2	1	2	2	GTPase Era	Membrane	20
A1AZX7/Pden_0709	2	1	2	2	Formiminoglutamate deiminase	Cytoplasmic	0
A1B0E5/Pden_0878	2	1	2	2	MOSC domain containing protein	Cytoplasmic	0
A1B3Z8/Pden_2150	2	1	2	2	Binding-protein-dependent transport systems inner membrane component	Membrane	5
A1B493/Pden_2245	2	1	2	2	Uncharacterized protein	Membrane	0
A1B574/Pden_2581	2	1	2	2	ErfK/YbiS/YcfS/YnhG family protein	Unknown	0
A1B690/Pden_2950	2	1	2	2	Uncharacterized protein	Unknown	0
A1B9B3/Pden_4041	2	1	2	2	Arsenate reductase related protein	Unknown	0
A1B9H0/Pden_4098	2	1	2	2	tRNA-specific 2-thiouridylase (MnmA)	Cytoplasmic	6,8,4,7
A1B9H2/Pden_4100	2	1	2	2	Uncharacterized protein	Unknown	0
A1B337/Pden_1834	2	1	1	3	Glutathione S-transferase, C-terminal domain	Cytoplasmic	0
A1B8X5/Pden_3903	2	0	2	3	Conserved hypothetical membrane protein	Membrane	0
A1B862/Pden_3639	2	3	2	1	3-methylcrotonoyl-CoA carboxylase, alpha subunit	Cytoplasmic	0
A1B1L3/Pden_1302	2	3	1	2	Polyphosphate kinase	Membrane	1,4,7,12,14
A1B2F0/Pden_1594	2	3	1	2	Polyphosphate kinase	Membrane	0
A1B9T1/Pden_4211	2	3	1	2	SSS sodium solute transporter superfamily	Membrane	12,5,13
A1AZI5/Pden_0567	2	2	2	2	Succinate dehydrogenase subunit C	Membrane	8,4,7,12,14
A1AZZ3/Pden_0725	2	2	2	2	TonB family protein	Unknown	5
A1B1C2/Pden_1211	2	2	2	2	Urease subunit gamma (Urea amidohydrolase subunit gamma)	Cytoplasmic	4,6,7,12,14,2
A1B3N0/Pden_2032	2	2	2	2	Ammonium transporter	Membrane	0
A1B3U2/Pden_2094	2	2	2	2	Tetraacyldisaccharide 4'-kinase (Lipid A 4'-kinase)	Cytoplasmic	8,1,4,7,12,14
A1B586/Pden_2593	2	2	2	2	Uncharacterized protein	Unknown	0
A1B5B0/Pden_2617	2	2	2	2	Iron-containing alcohol dehydrogenase	Cytoplasmic	0
A1B6A3/Pden_2963	2	2	2	2	Integral membrane sensor signal transduction histidine kinase	Membrane	0
A1B811/Pden_3587	2	2	2	2	Uncharacterized protein	Membrane	0
A1B880/Pden_3657	2	2	2	2	Uncharacterized protein	Unknown	0
A1B8X8/Pden_3906	2	2	2	2	3-hydroxyacyl-[acyl-carrier-protein] dehydratase (FabZ)	Cytoplasmic	8,1,4,7,12,14
A1BA93/Pden_4373	2	2	2	2	TonB-dependent siderophore receptor	OuterMembrane	5,13
A1BAA0/Pden_4380	2	2	2	2	Putative transcriptional regulator, GntR family	Cytoplasmic	1

A1BAR7/Pden_4548	2	2	2	2	Aldehyde dehydrogenase	Cytoplasmic	0
A1B470/Pden_2222	2	1	3	2	Thymidine phosphorylase	Unknown	8,6,4,7,12,14
A1B4W6/Pden_2473	2	1	3	2	Uncharacterized protein	Unknown	8,7
A1B537/Pden_2544	2	1	3	2	Cytochrome c, class II	Periplasmic	4,12,14
A1B5J9/Pden_2709	2	1	3	2	Saccharopine dehydrogenase (NAD+, L-lysine-forming)	Cytoplasmic	8,1,4,7,12,14
A1BA00/Pden_4280	2	3	0	3	Transcriptional regulator, IclR family	Cytoplasmic	1,6,8,4,7,9
A1BAC1/Pden_4401	2	3	0	3	Uncharacterized protein UPF0065	Unknown	0
A1AZJ2/Pden_0574	2	2	1	3	UPF0173 metal-dependent hydrolase Pden_0574	Cytoplasmic	0
P43089/Pden_1822	2	2	1	3	5-aminolevulinate synthase	Cytoplasmic	6,1,4,7
A1B3H6/Pden_1978	2	2	1	3	Periplasmic binding protein/LacI transcriptional regulator	Cytoplasmic	6,1,8,4,7
A1B353/Pden_1850	2	1	2	3	Putative transcriptional regulator, Crp/Fnr family (FnrP)	Membrane	6,1,8,4,7
A1B5K1/Pden_2711	2	1	2	3	Uncharacterized protein	Cytoplasmic	0
A1BAN0/Pden_4510	2	1	2	3	Probable protein kinase (UbiB)	Membrane	1,4,7,12,14
A1B3P5/Pden_2047	2	3	3	1	Arginase	Unknown	8,6,4,7,12,14
A1B4F5/Pden_2308	2	3	3	1	Ribokinase (RK)	Cytoplasmic	8,7,14
A1BA29/Pden_4309	2	3	3	1	Acetyl-CoA hydrolase	Cytoplasmic	4,7
A1BB88/Pden_4721	2	3	3	1	Periplasmic nitrate reductase	Periplasmic	8,1,4,7,12,14
A1AZZ7/Pden_0729	2	4	1	2	Aminotransferase, class V	Cytoplasmic	0
A1B0A2/Pden_0834	2	3	2	2	Glycosyl transferase, WecB/TagA/CpsF family	Cytoplasmic	1
A1B0I5/Pden_0918	2	3	2	2	Alpha/beta hydrolase fold protein	Cytoplasmic	0
A1B566/Pden_2573	2	3	2	2	33 kDa chaperonin	Cytoplasmic	25
A1B8V4/Pden_3882	2	3	2	2	Ribosome maturation factor (RimM)	Cytoplasmic	6,8,4,7,20
A1B909/Pden_3937	2	3	2	2	Transferase hexapeptide protein	Cytoplasmic	0
A1B502/Pden_2509	2	2	3	2	RNA pyrophosphohydrolase ((Di)nucleoside polyphosphate hydrolase)	Cytoplasmic	0
A1BB75/Pden_4708	2	2	3	2	Glyoxalase/bleomycin resistance protein/dioxygenase	Cytoplasmic	0
A1AZL2/Pden_0594	2	2	2	3	Fmu (Sun) domain protein	Cytoplasmic	0
A1B357/Pden_1854	2	2	2	3	Uncharacterized protein	Unknown	0
A1B3F3/Pden_1955	2	2	2	3	6-phosphogluconate dehydratase	Cytoplasmic	4,8,7,12,14,2
A1B5U1/Pden_2801	2	2	2	3	tRNA pseudouridine synthase B	Cytoplasmic	6,8,4,7
A1B5X8/Pden_2838	2	2	2	3	Uncharacterized protein	Unknown	0
A1B711/Pden_3224	2	2	2	3	Acyl-CoA dehydrogenase domain protein	Cytoplasmic	0
A1B8V9/Pden_3887	2	2	2	3	Chromosome partitioning protein	Cytoplasmic	0
A1B5G4/Pden_2671	2	4	3	1	Amidase	Cytoplasmic	0
A1B5Z7/Pden_2857	2	4	3	1	Formate dehydrogenase beta subunit	Cytoplasmic	0
A1B0B3/Pden_0845	2	3	3	2	ATPases involved in chromosome partitioning-like protein	Unknown	0
A1B459/Pden_2211	2	3	3	2	Laccase domain protein	Unknown	0
A1AY61/Pden_0088	2	4	1	3	Flagellin modification protein (FlmD)	Cytoplasmic	0
A1AZY3/Pden_0715	2	4	1	3	Urocanate hydratase (Urocanase) (Imidazolonepropionate hydrolase)	Cytoplasmic	4,8,7,12,14,2
A1B500/Pden_2507	2	3	2	3	Peptidase M23B	Unknown	0
A1AXX9/Pden_0006	2	2	3	3	Cobyrinic acid a,c-diamide synthase	Membrane	0
A1B8B7/Pden_3694	2	2	3	3	CBS domain containing protein	Membrane	0
A1B8C0/Pden_3697	2	2	3	3	Thiamine monophosphate synthase	Cytoplasmic	1,6,4,7,12,14
A1BAM9/Pden_4509	2	2	3	3	Uncharacterized protein	Cytoplasmic	0
A1AZ21/Pden_0401	2	1	4	3	SH3, type 3 domain protein	Membrane	0

A1B9H3/Pden_4101	2	2	2	4	GCN5-related N-acetyltransferase	Unknown	0
A1BC41/Pden_5025	2	2	2	4	Glycerol-3-phosphate cytidylyltransferase	Cytoplasmic	1,4,7,12,14
A1AZ15/Pden_0395	2	4	3	2	Diaminopimelate epimerase (DAP epimerase)	Cytoplasmic	8,1,4,7,12,14
A1B883/Pden_3660	2	3	4	2	HAD-superfamily subfamily IIA hydrolase like protein	Cytoplasmic	0
A1B560/Pden_2567	2	4	2	3	Periplasmic binding protein	Unknown	0
A1AZF5/Pden_0537	2	3	3	3	Peptidoglycan-binding domain 1 protein	Cytoplasmic	0
A1B060/Pden_0792	2	3	3	3	ATP12 ATPase	Unknown	3
A1B4F3/Pden_2306	2	3	3	3	Cytochrome *b*	Membrane	4,12,14,
A1B9S9/Pden_4209	2	3	3	3	Phosphate butyryltransferase	Cytoplasmic	0
A1B378/Pden_1877	2	2	4	3	Exonuclease (RecJ)	Cytoplasmic	4,8,7,12,14,13
A1B5U3/Pden_2803	2	2	4	3	Beta-lactamase domain protein	Unknown	0
A1B8B8/Pden_3695	2	2	4	3	GCN5-related N-acetyltransferase	Cytoplasmic	0
A1B582/Pden_2589	2	3	2	4	Ubiquinone biosynthesis *O*-methyltransferase	Cytoplasmic	1,4,7,12,14
A1B0P2/Pden_0975	2	2	3	4	Putative phage repressor	Cytoplasmic	0
A1B4L3/Pden_2367	2	2	3	4	Alcohol dehydrogenase GroES domain protein	Cytoplasmic	0
A1B698/Pden_2958	2	2	3	4	Transcriptional regulator, BadM/Rrf2 family	Unknown	0
A1BAL9/Pden_4499	2	4	4	2	Homoserine O-succinyltransferase (Homoserine O-transsuccinylase) (HTS)	Cytoplasmic	8,1,4,7,12,14
A1BAF2/Pden_4432	2	5	2	3	Quinolinate synthase A	Cytoplasmic	8,1,4,7,12,14
A1B1V3/Pden_1396	2	4	3	3	Glutamine amidotransferase class-I	Cytoplasmic	8,6,4,7,12,14
A1B433/Pden_2185	2	4	3	3	Glycoside hydrolase, family 25	Unknown	6,8,4,7,2
A1BCF0/Pden_5134	2	4	3	3	Uncharacterized protein	Unknown	0
A1B3Y5/Pden_2137	2	3	4	3	Anthranilate phosphoribosyltransferase	Membrane	8,1,4,7,12,14
A1B5N3/Pden_2743	2	3	4	3	Elongation factor P (EF-P)	Cytoplasmic	0
A1B952/Pden_3980	2	3	4	3	NUDIX hydrolase	Unknown	0
A1B9D5/Pden_4063	2	4	2	4	Cysteine--tRNA ligase (Cysteinyl-tRNA synthetase) (CysRS)	Cytoplasmic	8,6,4,7,12,14
A1B9U5/Pden_4225	2	3	3	4	Flavin prenyltransferase UbiX	Membrane	1,4
A1BAN4/Pden_4514	2	3	3	4	30S ribosomal protein S20	Cytoplasmic	6,1,8,4,7
A1AYU5/Pden_0325	2	2	4	4	Uncharacterized protein	Unknown	0
A1B584/Pden_2591	2	2	4	4	Ribosome maturation factor (RimP)	Cytoplasmic	20
A1AZS0/Pden_0652	2	4	4	3	Aminotransferase	Cytoplasmic	1
A1B5G2/Pden_2669	2	4	4	3	Outer-membrane lipoprotein carrier protein	Unknown	5,23
A1B5I5/Pden_2692	2	4	4	3	Mammalian cell entry related domain protein	Unknown	0
A1B345/Pden_1842	2	3	5	3	Heavy metal translocating P-type ATPase	Membrane	5,13
A1BAL1/Pden_4491	2	4	3	4	UDP-N-acetylenolpyruvoylglucosamine reductase	Cytoplasmic	12,7,14,9,10,19
A1B1N1/Pden_1320	2	2	5	4	Uncharacterized protein	Cytoplasmic	0
A1B1N4/Pden_1323	2	2	5	4	Pyridoxamine 5'-phosphate oxidase-related, FMN-binding protein	Unknown	0
A1B2S2/Pden_1719	2	4	2	5	AMP-dependent synthetase and ligase	Cytoplasmic	0
A1B4Y6/Pden_2493	2	4	2	5	Transcriptional regulator, AsnC family	Unknown	9
A1B3W9/Pden_2121	2	6	3	3	dTDP-glucose 4,6-dehydratase	Cytoplasmic	6,8,4,7
A1B916/Pden_3944	2	5	3	4	Transcriptional regulator, GntR family	Cytoplasmic	6,1,8,4,7
A1B0E8/Pden_0881	2	3	5	4	Putative phosphohistidine phosphatase (SixA)	Cytoplasmic	0
A1B2S0/Pden_1717	2	3	4	5	Amino acid/amide ABC transporter substrate-binding protein, HAAT family	Unknown	0
A1B996/Pden_4024	2	3	4	5	Uncharacterized protein	Unknown	0
A1B3V3/Pden_2105	2	5	5	3	Transcriptional regulator, GntR family	Cytoplasmic	6,1,8,4,7

A1AZF6/Pden_0538	2	5	4	4	Glycine--tRNA ligase alpha subunit	Cytoplasmic	8,6,4,7,12,14
A1B8V7/Pden_3885	2	5	3	5	50S ribosomal protein L31	Cytoplasmic	6,1,8,4,7
A1B323/Pden_1820	2	4	4	5	4-hydroxy-3-methylbut-2-en-1-yl diphosphate synthase (flavodoxin)	Cytoplasmic	8,1,4,7,12,14
A1B3L8/Pden_2020	2	4	4	5	Diaminopimelate decarboxylase (DAP decarboxylase) (DAPDC)	Cytoplasmic	8,1,4,7,12,14
A1B946/Pden_3974	2	4	4	5	Cytochrome c-type biogenesis protein (CcmE)	Unknown	8,3,4,7
A1B2M0/Pden_1667	2	3	3	7	ABC transporter related protein	Membrane	5,13
A1B054/Pden_0786	2	7	4	3	Transcriptional regulator, LuxR family	Unknown	9
A1B4I5/Pden_2338	2	6	4	4	4-aminobutyrate aminotransferase apoenzyme	Cytoplasmic	8,6,4,7,12,14
A1B307/Pden_1804	2	7	2	5	ABC transporter related protein	Membrane	5,13
A1B5H2/Pden_2679	2	4	4	6	Surface antigen (D15)	OuterMembrane	0
A1B1L4/Pden_1303	2	5	5	5	Ppx/GppA phosphatase	Cytoplasmic	0
A1B656/Pden_2916	2	5	5	5	Biotin synthase 2	Cytoplasmic	1,6,4,7,12,14
A1B5A7/Pden_2614	2	4	4	7	Bifunctional protein FolD 1	Cytoplasmic	8,1,4,7,12,14
A1BBS1/Pden_4905	2	4	4	7	Bifunctional protein FolD 2	Cytoplasmic	8,1,4,7,12,14
A1BAL5/Pden_4495	2	5	4	7	UDP-N-acetylglucosamine--N-acetylmuramyl-(pentapeptide) pyrophosphoryl-undecaprenol N-acetylglucosamine transferase	Membrane	12,7,14,9,10,19
A1B966/Pden_3994	2	5	6	7	Ribosome-recycling factor (RRF) (Ribosome-releasing factor)	Cytoplasmic	3,12
A1B1F6/Pden_1245	2	5	8	6	2-keto-3-deoxy-phosphogluconate aldolase	Cytoplasmic	0
A1AY46/Pden_0073	2	6	6	7	Cobyrinic acid a,c-diamide synthase	Membrane	0
A1B0B2/Pden_0844	1	2	0	0	Glycosyl transferase, family 2	Cytoplasmic	0
A1B5Q2/Pden_2762	1	2	0	0	Alanine racemase domain protein	Cytoplasmic	0
A1B5R3/Pden_2773	1	2	0	0	Glycosyl transferase, group 1	Cytoplasmic	0
A1B648/Pden_2908	1	2	0	0	Short-chain dehydrogenase/reductase SDR	Unknown	0
A1AXY8/Pden_0015	1	0	0	2	Glutathione-dependent formaldehyde-activating enzyme	Cytoplasmic	4,7,12,14,2
A1B0J3/Pden_0926	1	0	0	2	Uncharacterized protein	Unknown	0
A1B3N2/Pden_2034	1	0	0	2	Toluene tolerance family protein	Unknown	0
A1B525/Pden_2532	1	0	0	2	Cobaltochelatase CobN subunit	Cytoplasmic	1
A1B8G9/Pden_3746	1	0	0	2	Putative transcriptional regulator, XRE family	Unknown	0
A1B999/Pden_4027	1	0	0	2	Ferredoxin	Cytoplasmic	0
A1B9F3/Pden_4081	1	0	0	2	NifU-related protein involved in Fe-S cluster formation	Unknown	0
A1BBN4/Pden_4868	1	0	0	2	Transcriptional regulator, GntR family	Cytoplasmic	6,1,8,4,7
A1AXX6/Pden_0003	1	2	1	0	tRNA modification GTPase MnmE	Cytoplasmic	6,8,4,7
A1AY45/Pden_0072	1	2	1	0	CHAD domain containing protein	Cytoplasmic	0
A1AZZ2/Pden_0724	1	2	1	0	Transcriptional regulator, LysR family	Cytoplasmic	6,1,8,4,7
A1BBG9/Pden_4802	1	2	1	0	Transcriptional regulator, TetR family	Cytoplasmic	1,6,8,4,7,9
A1AZZ8/Pden_0730	1	1	2	0	ABC transporter related protein	Membrane	0
A1B0I8/Pden_0921	1	1	2	0	NAD kinase (ATP-dependent NAD kinase)	Cytoplasmic	8,1,4,7,12,14
A1B157/Pden_1143	1	1	2	0	ABC transporter related protein	Membrane	0
A1B8K7/Pden_3785	1	1	2	0	2-nitropropane dioxygenase, NPD	Unknown	0
A1B1Z0/Pden_1433	1	2	0	1	Biotin synthase 1	Cytoplasmic	1,6,4,7,12,14
A1B3L3/Pden_2015	1	2	0	1	1-acyl-sn-glycerol-3-phosphate acyltransferase	Membrane	0
A1B8P8/Pden_3826	1	2	0	1	RNA methyltransferase, TrmH family, group 3	Cytoplasmic	6,8,4,7
A1B8S8/Pden_3856	1	2	0	1	Substrate-binding region of ABC-type glycine betaine transport system	Periplasmic	0
A1B9Y0/Pden_4260	1	2	0	1	Guanine deaminase	Cytoplasmic	4,8,7,12,14,2

A1BAM6/Pden_4506	1	2	0	1	Ornithine-acyl[acyl carrier protein] N-acyltransferase	Cytoplasmic	0
A1AXZ1/Pden_0018	1	1	1	1	GCN5-related N-acetyltransferase	Unknown	0
A1B4Q6/Pden_2412	1	0	2	1	Type I secretion membrane fusion protein, HlyD family	Membrane	12,5,13,23
A1B5A0/Pden_2607	1	1	0	2	Uncharacterized protein	Unknown	0
A1B5L1/Pden_2721	1	1	0	2	Efflux transporter, RND family, MFP subunit	Membrane	12,5,13
A1B842/Pden_3619	1	1	0	2	4-hydroxy-3-methylbut-2-enyl diphosphate reductase	Cytoplasmic	8,1,4,7,12,14
A1BC23/Pden_5007	1	1	0	2	Monooxygenase (NtaA/SnaA/SoxA)	Cytoplasmic	0
A1AYW7/Pden_0347	1	0	1	2	Uncharacterized protein	Unknown	0
A1B125/Pden_1111	1	0	1	2	NCS1 nucleoside transporter family	Membrane	12,5,13
A1B1R9/Pden_1362	1	0	1	2	Periplasmic binding protein	Unknown	0
A1B306/Pden_1803	1	0	1	2	Glycosyl transferase, group 1	Cytoplasmic	0
A1B329/Pden_1826	1	0	1	2	4Fe-4S ferredoxin, iron-sulfur binding domain	Cytoplasmic	0
A1B4B9/Pden_2271	1	0	1	2	Arsenate reductase	Cytoplasmic	0
A1B5M7/Pden_2737	1	0	1	2	Uncharacterized protein	Unknown	0
A1BBV6/Pden_4940	1	0	1	2	Transcriptional regulator, GntR family	Cytoplasmic	4,8,7,12,14,9
A1B1B5/Pden_1204	1	3	0	1	D-alanine--D-alanine ligase (D-Ala-D-Ala ligase) (D-alanylalanine synthetase)	Cytoplasmic	12,7,14,9,10,19
A1AZW2/Pden_0694	1	2	1	1	Protease HtpX homolog	Membrane	0
A1B1X7/Pden_1420	1	2	1	1	tRNA-dihydrouridine(20/20a) synthase	Cytoplasmic	0
A1B360/Pden_1857	1	2	1	1	Glycosyl transferase, family 14	Cytoplasmic	0
A1B480/Pden_2232	1	2	1	1	NADH dehydrogenase subunit M	Membrane	4,12,14
A1B4W2/Pden_2469	1	2	1	1	Carbohydrate ABC transporter ATP-binding protein, CUT1 family	Membrane	0
A1B5G3/Pden_2670	1	2	1	1	2-octaprenyl-3-methyl-6-methoxy-1,4-benzoquinol hydroxylase / 2-octaprenyl-6-methoxyphenol hydroxylase	Membrane	1,4,7,12,14
A1B5W3/Pden_2823	1	2	1	1	2-nitropropane dioxygenase, NPD	Unknown	0
A1B619/Pden_2879	1	2	1	1	ATP synthase subunit a (ATP synthase F0 sector subunit a) (F-ATPase subunit 6)	Membrane	7,4,12,14,5,13
A1B844/Pden_3621	1	2	1	1	Uncharacterized protein	Unknown	0
A1B913/Pden_3941	1	2	1	1	DNA repair protein (RadA)	Membrane	4,8,7,12,14,13
A1B951/Pden_3979	1	2	1	1	Enoyl-CoA hydratase	Cytoplasmic	0
A1B973/Pden_4001	1	2	1	1	Purine nucleosidase	Cytoplasmic	0
A1B9V3/Pden_4233	1	2	1	1	Respiratory nitrate reductase gamma subunit	Membrane	0
A1BAE6/Pden_4426	1	2	1	1	Glycogen synthase	Cytoplasmic	8,1,4,7,12,14
A1AYQ1/Pden_0281	1	1	2	1	Hydroxyectoine-binding protein /ectoine-binding protein	Periplasmic	0
A1AZ16/Pden_0396	1	1	2	1	MiaB-like tRNA modifying enzyme	Cytoplasmic	6,8,4,7
A1AZF0/Pden_0532	1	1	2	1	UspA domain protein	Cytoplasmic	11
A1B078/Pden_0810	1	1	2	1	Extracellular solute-binding protein, family 5	Periplasmic	12,5,13
A1B194/Pden_1183	1	1	2	1	Transcriptional regulator, GntR family	Cytoplasmic	6,1,8,4,7
A1B1G5/Pden_1254	1	1	2	1	Aldehyde dehydrogenase	Cytoplasmic	0
A1B324/Pden_1821	1	1	2	1	Uncharacterized protein	Cytoplasmic	0
A1B420/Pden_2172	1	1	2	1	Peptidase M48, Ste24p	Unknown	0
A1B483/Pden_2235	1	1	2	1	NADH dehydrogenase subunit J	Membrane	0
A1B8A9/Pden_3686	1	1	2	1	Uncharacterized protein	Unknown	0
A1B912/Pden_3940	1	1	2	1	Colicin V production protein	Membrane	1,4,12,14
A1B914/Pden_3942	1	1	2	1	Ubiquinone biosynthesis hydroxylase, UbiH/UbiF/VisC/COQ6 family	Cytoplasmic	1,4,7,12,14
A1BA47/Pden_4327	1	1	2	1	Phosphate import ATP-binding protein (PstB)	Membrane	0

A1BA26/Pden_4306	1	2	0	2	Methionine import ATP-binding protein (MetN)	Membrane	5,13
A1AZ39/Pden_0419	1	1	1	2	Ribonuclease 3 (RNase III)	Cytoplasmic	6,8,4,7,2
A1AZ52/Pden_0432	1	1	1	2	*aa₃* type cytochrome *c* oxidase subunit IV	Membrane	0
A1AZ56/Pden_0436	1	1	1	2	UPF0301 protein Pden_0436	Cytoplasmic	0
A1AZN0/Pden_0612	1	1	1	2	Uncharacterized protein	Unknown	0
A1AZP2/Pden_0624	1	1	1	2	Ribosomal RNA small subunit methyltransferase E	Cytoplasmic	6,8,4,7
A1AZW7/Pden_0699	1	1	1	2	Multisubunit potassium/proton antiporter, (PhaD)	Membrane	4,12,14
A1B0B9/Pden_0851	1	1	1	2	Nicotinamidase	Cytoplasmic	0
A1B3E9/Pden_1951	1	1	1	2	6-phosphogluconolactonase	Cytoplasmic	8,6,4,7,12,14
A1B428/Pden_2180	1	1	1	2	GCN5-related N-acetyltransferase	Unknown	0
A1B469/Pden_2221	1	1	1	2	Phosphopentomutase (Phosphodeoxyribomutase)	Cytoplasmic	4,8,7,12,14,2
A1B4B7/Pden_2269	1	1	1	2	Alcohol dehydrogenase, zinc-binding domain protein	Cytoplasmic	0
A1B596/Pden_2603	1	1	1	2	PTS IIA-like nitrogen-regulatory protein (PtsN)	Cytoplasmic	0
A1B5A1/Pden_2608	1	1	1	2	KpsF/GutQ family protein	Unknown	8,7
A1B5S5/Pden_2785	1	1	1	2	FxsA cytoplasmic membrane protein	Membrane	0
A1B8L0/Pden_3788	1	1	1	2	Uncharacterized protein	Membrane	0
A1B8R1/Pden_3839	1	1	1	2	Uncharacterized protein	Cytoplasmic	6,11,8,4,7
A1B950/Pden_3978	1	1	1	2	Cytochrome C biogenesis protein	Membrane	0
A1B969/Pden_3997	1	1	1	2	1-deoxy-D-xylulose 5-phosphate reductoisomerase	Cytoplasmic	8,1,4,7,12,14
A1BBC2/Pden_4755	1	1	1	2	Putative carbohydrate binding protein	Membrane	8,7
A1B1K3/Pden_1292	1	0	2	2	AMP-dependent synthetase and ligase	Cytoplasmic	0
A1B412/Pden_2164	1	0	2	2	GatB/Yqey domain protein	Cytoplasmic	0
A1B505/Pden_2512	1	0	2	2	YbaK/prolyl-tRNA synthetase associated region	Unknown	0
A1B548/Pden_2555	1	0	2	2	Aspartate carbamoyltransferase (Aspartate transcarbamylase) (ATCase)	Cytoplasmic	8,1,4,7,12,14
A1B898/Pden_3675	1	0	2	2	Uncharacterized protein	Cytoplasmic	0
A1AY64/Pden_0091	1	0	1	3	Uncharacterized protein	Cytoplasmic	0
A1B7Y1/Pden_3555	1	0	1	3	Uncharacterized protein	Cytoplasmic	0
A1AYH6/Pden_0206	1	2	3	0	Short-chain dehydrogenase/reductase SDR	Cytoplasmic	0
A1B908/Pden_3936	1	4	0	1	Guanylate kinase (GMP kinase)	Cytoplasmic	0
A1B063/Pden_0795	1	3	1	1	L-glutamate ABC transporter membrane protein / L-asparagine ABC transporter membrane protein / L-glutamine ABC transporter membrane protein / L-aspartate ABC transporter membrane protein	Membrane	0
A1B8C4/Pden_3701	1	3	1	1	tRNA-2-methylthio-N(6)-dimethylallyladenosine synthase	Cytoplasmic	6,8,4,7
A1B915/Pden_3943	1	3	1	1	Pyrimidine 5'-nucleotidase	Cytoplasmic	0
A1B9X8/Pden_4258	1	3	1	1	Xanthine dehydrogenase, molybdenum binding subunit apoprotein / Xanthine oxidase	Cytoplasmic	0
A1BBN1/Pden_4865	1	3	1	1	Aldehyde dehydrogenase	Cytoplasmic	0
A1B0A0/Pden_0832	1	2	2	1	ABC transporter related protein	Membrane	0
A1B351/Pden_1848	1	2	2	1	Cytochrome *c* oxidase, *cbb₃*-type, subunit I	Membrane	4,12,14
A1B3E8/Pden_1950	1	2	2	1	Glucose-6-phosphate isomerase (GPI)	Cytoplasmic	4,8,7,12,14,2
A1B5F2/Pden_2659	1	2	2	1	Murein endopeptidase, Metallo peptidase, MEROPS family M74	Periplasmic	0
A1B5M0/Pden_2730	1	2	2	1	Protein-L-isoaspartate *O*-methyltransferase	Cytoplasmic	8,4,7
A1B5Z0/Pden_2850	1	2	2	1	Nucleotide-binding protein	Cytoplasmic	0

A1B8Z9/Pden_3927	1	2	2	1	Phosphoribosylglycinamide formyltransferase (5'-phosphoribosylglycinamide transformylase) (GAR transformylase) (GART)	Cytoplasmic	8,1,4,7,12,14
A1BB77/Pden_4710	1	2	2	1	Glucose-methanol-choline oxidoreductase	Cytoplasmic	0
A1BBL2/Pden_4846	1	2	2	1	Periplasmic binding protein/LacI transcriptional regulator	Cytoplasmic	6,1,8,4,7
A1B0Y2/Pden_1067	1	1	3	1	OsmC family protein	Cytoplasmic	11
A1B9L2/Pden_4142	1	1	3	1	Pseudouridine synthase	Cytoplasmic	6,8,4,7
A1AZY9/Pden_0721	1	2	1	2	Patatin	Cytoplasmic	8,7,14
A1B0G5/Pden_0898	1	2	1	2	4-hydroxythreonine-4-phosphate dehydrogenase	Cytoplasmic	1,6,4,7,12,14
A1B2V6/Pden_1753	1	2	1	2	Uncharacterized protein	Unknown	0
A1B3F8/Pden_1960	1	2	1	2	Uncharacterized protein	Cytoplasmic	0
A1B5T4/Pden_2794	1	2	1	2	UvrD-like DNA helicase, C-terminal domain	Cytoplasmic	4,8,7,12,14,13
A1B9J6/Pden_4126	1	2	1	2	TrkA-N domain protein	Membrane	0
A1B9T5/Pden_4215	1	2	1	2	NosL family protein	Unknown	0
A1AZ63/Pden_0443	1	1	2	2	Uncharacterized protein	Cytoplasmic	0
A1AZP0/Pden_0622	1	1	2	2	Ribosomal protein L11 methyltransferase	Cytoplasmic	0
A1B096/Pden_0828	1	1	2	2	Hydratase/decarboxylase	Membrane	0
A1B375/Pden_1874	1	1	2	2	Heat shock protein DnaJ domain protein	Cytoplasmic	0
A1B4A3/Pden_2255	1	1	2	2	Uncharacterized protein	Periplasmic	0
A1B4D6/Pden_2289	1	1	2	2	Glutathione S-transferase, N-terminal domain	Cytoplasmic	0
A1B4F8/Pden_2311	1	1	2	2	Short-chain dehydrogenase/reductase SDR	Cytoplasmic	0
A1B605/Pden_2865	1	1	2	2	Uncharacterized protein	Cytoplasmic	0
A1B8V2/Pden_3880	1	1	2	2	Chorismate mutase	Cytoplasmic	4,7,12,14
A1B910/Pden_3938	1	1	2	2	PAS/PAC sensor signal transduction histidine kinase	Membrane	9
A1B8U7/Pden_3875	1	2	0	3	Methylmalonyl-CoA mutase	Cytoplasmic	0
A1AY85/Pden_0112	1	1	1	3	Uncharacterized protein	Cytoplasmic	0
A1B4N0/Pden_2384	1	1	1	3	Short-chain dehydrogenase/reductase SDR	Unknown	1,6,4,7,12,14
A1B5M5/Pden_2735	1	1	1	3	Glycerophosphoryl diester phosphodiesterase	Cytoplasmic	8,7,14
A1B9U7/Pden_4227	1	1	1	3	Uncharacterized protein	Membrane	0
A1BBZ8/Pden_4982	1	1	1	3	Short-chain dehydrogenase/reductase SDR	Unknown	0
A1AZK0/Pden_0582	1	1	0	4	Transcriptional regulator (MraZ)	Cytoplasmic	6,1,8,4,7
A1AZU8/Pden_0680	1	3	2	1	Transcriptional regulator, GntR family	Cytoplasmic	6,1,8,4,7
A1BBF5/Pden_4788	1	2	3	1	Uncharacterized protein	Unknown	0
A1AZS1/Pden_0653	1	3	1	2	Transcriptional regulator, AsnC family	Cytoplasmic	6,1,8,4,7
A1B9A9/Pden_4037	1	3	1	2	D-isomer specific 2-hydroxyacid dehydrogenase, NAD-binding protein	Cytoplasmic	14
A1B2T5/Pden_1732	1	2	2	2	Putative glutathione *S*-transferase	Cytoplasmic	0
A1B2W5/Pden_1762	1	2	2	2	DNA-directed DNA polymerase	Cytoplasmic	6,1,8,4,7
A1B8W9/Pden_3897	1	2	2	2	Deoxyribose-phosphate aldolase	Cytoplasmic	4,8,7,12,14,2
A1B0F0/Pden_0883	1	1	3	2	Uncharacterized protein	Cytoplasmic	0
A1B3Y8/Pden_2140	1	1	3	2	Cyclic pyranopterin monophosphate synthase accessory protein	Cytoplasmic	6,1,8,4,7
A1B9J4/Pden_4124	1	2	1	3	RNA-binding protein (Hfq)	Cytoplasmic	9
A1BC33/Pden_5017	1	2	1	3	Uncharacterized protein	Unknown	0
A1B4N1/Pden_2386	1	0	3	3	2,3-dihydroxybenzoate-AMP ligase	Cytoplasmic	1,6,4,7,12,14
A1B2Z3/Pden_1790	1	2	0	4	Translation initiation factor IF-1	Cytoplasmic	0
A1B4G1/Pden_2314	1	1	1	4	Uncharacterized protein	Unknown	0
A1B962/Pden_3990	1	1	1	4	Ribosomal RNA large subunit methyltransferase	Cytoplasmic	0

A1B346/Pden_1843	1	0	2	4	FixH family protein	Membrane	0
A1B697/Pden_2957	1	4	2	1	Oxidoreductase domain protein	Unknown	0
A1BAF3/Pden_4433	1	4	2	1	Transcriptional regulator, IclR family	Cytoplasmic	1,6,8,4,7,9
A1B4G6/Pden_2319	1	3	3	1	Methylenetetrahydrofolate reductase	Cytoplasmic	8,1,4,7,12,14
A1B0H8/Pden_0911	1	2	4	1	Penicillin amidase, Cysteine peptidase, MEROPS family C59	Unknown	0
A1AYE1/Pden_0169	1	3	2	2	Regulatory protein, IclR	Cytoplasmic	1,6,8,4,7,9
A1AYG9/Pden_0197	1	3	2	2	3-hydroxyacyl-CoA dehydrogenase	Unknown	4,8,7,12,14,2
A1AY16/Pden_0043	1	2	3	2	HAD-superfamily hydrolase, subfamily IA	Unknown	0
A1B058/Pden_0790	1	2	3	2	Pseudouridine synthase	Cytoplasmic	6,8,4,7
A1B2M5/Pden_1672	1	2	3	2	5-deoxyglucuronate isomerase	Cytoplasmic	4,8,7,12,14,2
A1B2N4/Pden_1681	1	2	3	2	Monosaccharide ABC transporter substrate-binding protein, CUT2 family	Periplasmic	0
A1B369/Pden_1866	1	2	3	2	Segregation and condensation protein B	Cytoplasmic	12
A1B372/Pden_1869	1	2	3	2	Sporulation domain protein	Unknown	0
A1B678/Pden_2938	1	2	3	2	Efflux transporter, RND family, MFP subunit	Membrane	12,5,13
A1B716/Pden_3229	1	2	3	2	Uncharacterized protein	Cytoplasmic	0
A1B7M2/Pden_3445	1	2	3	2	Efflux transporter, RND family, MFP subunit	Membrane	12,5,13
A1B930/Pden_3958	1	2	3	2	Ribosomal RNA small subunit methyltransferase I	Cytoplasmic	6,8,4,7,24
A1AYU0/Pden_0320	1	1	4	2	UPF0271 protein Pden_0320	Unknown	8,7
A1B9C7/Pden_4055	1	3	1	3	Glutamate 5-kinase (Gamma-glutamyl kinase)	Cytoplasmic	8,1,4,7,12,14
A1B0K2/Pden_0935	1	2	2	3	Glyoxalase/bleomycin resistance protein/dioxygenase	Cytoplasmic	0
A1B974/Pden_4002	1	2	2	3	Penicillin amidase	Unknown	1,4
A1B4X1/Pden_2478	1	2	1	4	Putative transcriptional regulator, Crp/Fnr family	Cytoplasmic	1,6,8,4,7,9
A1B2L0/Pden_1657	1	1	2	4	3-methyl-2-oxobutanoate hydroxymethyltransferase	Cytoplasmic	1,6,4,7,12,14
A1B0E6/Pden_0879	1	3	3	2	Probable GTP-binding protein (EngB)	Unknown	3,12
A1B843/Pden_3620	1	3	3	2	Adenine phosphoribosyltransferase (APRT)	Cytoplasmic	8,1,4,7,12,14
A1BA32/Pden_4312	1	3	3	2	FAD linked oxidase domain protein	Cytoplasmic	0
A1B1B6/Pden_1205	1	2	4	2	Urease accessory protein (UreG)	Cytoplasmic	6
A1B0C1/Pden_0853	1	3	2	3	NH(3)-dependent NAD(+) synthetase	Cytoplasmic	8,1,4,7,12,14
A1B4B5/Pden_2267	1	3	2	3	30S ribosomal protein (S21)	Unknown	6,1,8,4,7
A1B5X3/Pden_2833	1	3	2	3	Chromosomal replication initiator protein (DnaA)	Cytoplasmic	6,8,4,7,9
A1B6Z3/Pden_3206	1	3	2	3	Transcriptional regulator, GntR family	Cytoplasmic	6,1,8,4,7
A1B4Q7/Pden_2413	1	2	3	3	ABC transporter related protein	Membrane	0
A1B2X1/Pden_1768	1	2	2	4	Acyl carrier protein (ACP)	Cytoplasmic	0
A1B362/Pden_1859	1	2	2	4	50S ribosomal protein (L32)	Cytoplasmic	6,1,8,4,7
A1B6M1/Pden_3084	1	2	2	4	Lipoprotein, putative	Unknown	0
A1B942/Pden_3970	1	2	2	4	Cysteine desulfurase	Cytoplasmic	8,6,4,7,12,14
A1B928/Pden_3956	1	5	2	2	Bifunctional uridylyltransferase/uridylyl-removing enzyme (UTase/UR)	Cytoplasmic	6,9
A1AZZ9/Pden_0731	1	3	4	2	Glycine cleavage T protein (Aminomethyl transferase)	Cytoplasmic	0
A1B967/Pden_3995	1	5	1	3	Isoprenyl transferase	Cytoplasmic	0
A1B2M1/Pden_1668	1	3	3	3	ABC transporter related protein	Membrane	5,13
A1B4L6/Pden_2370	1	3	3	3	Peptidase S16, lon domain protein	Cytoplasmic	8,7,2
A1B457/Pden_2209	1	2	4	3	Transcriptional regulator, AsnC family	Cytoplasmic	6,1,8,4,7
A1BAN5/Pden_4515	1	3	2	4	3-demethylubiquinone-9 3-methyltransferase	Unknown	0

A1AZ19/Pden_0399	1	5	2	3	Farnesyl-diphosphate synthase	Cytoplasmic	8,1,4,7,12,14
A1B5Z3/Pden_2853	1	2	5	3	Ribonuclease R (RNase R)	Cytoplasmic	0
A1B688/Pden_2948	1	4	4	3	Antifreeze protein, type I	Cytoplasmic	0
A1B2A1/Pden_1545	1	4	3	4	Histone family protein nucleoid-structuring protein H-NS	Unknown	9
A1B6U3/Pden_3156	1	3	3	5	Histone family protein nucleoid-structuring protein H-NS	Unknown	9
A1B7C8/Pden_3349	1	3	3	5	Nucleoid protein H-NS	Unknown	9
A1B497/Pden_2249	1	4	4	4	NADH-quinone oxidoreductase subunit B	Membrane	5
A1B8N3/Pden_3811	1	6	3	4	Transcription-repair-coupling factor (TRCF)	Cytoplasmic	3,9
A1AZ40/Pden_0420	1	5	4	4	Signal peptidase I	Membrane	0
A1AXY4/Pden_0011	1	3	5	5	Non-canonical purine NTP pyrophosphatase	Cytoplasmic	4,8,7,12,14,2
A1B3N7/Pden_2039	1	5	4	5	Ornithine carbamoyltransferase (OTCase)	Cytoplasmic	8,1,4,7,12,14
A1B2S3/Pden_1720	0	2	0	0	Malonyl-CoA decarboxylase	Cytoplasmic	8,1,4,7,12,14
A1B3I1/Pden_1983	0	2	0	0	Uncharacterized protein	Cytoplasmic	0
A1B5L5/Pden_2725	0	2	0	0	Uncharacterized protein	Cytoplasmic	0
A1BB21/Pden_4654	0	2	0	0	HAD superfamily (Subfamily IIIB) phosphatase, TIGR01672	Unknown	0
A1B2K7/Pden_1654	0	0	2	0	Lysine--tRNA ligase	Cytoplasmic	8,6,4,7,12,14
A1B6Q2/Pden_3115	0	0	2	0	Uncharacterized protein	Membrane	0
A1B9T4/Pden_4214	0	0	2	0	FAD:protein FMN transferase	Unknown	8,4,7
A1BAN1/Pden_4511	0	0	2	0	Ubiquinone/menaquinone biosynthesis C-methyltransferase (UbiE)	Cytoplasmic	1,4,7,12,14
A1AZF9/Pden_0541	0	0	0	2	Pyruvate phosphate dikinase	Cytoplasmic	4,7,12,14
A1B8W8/Pden_3896	0	0	0	2	Uncharacterized protein	Membrane	0
A1BA36/Pden_4316	0	0	0	2	SURF1-like protein	Unknown	0
A1BB59/Pden_4692	0	0	0	2	Phosphatidylserine decarboxylase proenzyme	Cytoplasmic	8,1,4,7,12,14
A1BBA6/Pden_4739	0	0	0	2	Alkylphosphonate utilization protein (PhnA)	Unknown	0
A1B3T7/Pden_2089	0	3	0	0	Uncharacterized protein	Cytoplasmic	0
A1B1I0/Pden_1269	0	2	1	0	Uncharacterized protein	Unknown	0
A1BBT9/Pden_4923	0	2	1	0	Transcriptional regulator, IclR family	Cytoplasmic	1,6,8,4,7,9
A1AZQ7/Pden_0639	0	1	2	0	Uncharacterized protein	Cytoplasmic	0
A1BC60/Pden_5044	0	1	2	0	Aldo/keto reductase	Cytoplasmic	0
A1BC82/Pden_5066	0	1	2	0	TRAP dicarboxylate transporter, DctP subunit	Periplasmic	5
A1B316/Pden_1813	0	2	0	1	Pseudouridine-5'-phosphate glycosidase	Unknown	4,8,7,12,14,2
A1B3J8/Pden_2000	0	2	0	1	YCII-related protein	Unknown	0
A1B4B6/Pden_2268	0	2	0	1	RpsU-divergently transcribed protein	Cytoplasmic	1,4,7,12,14
A1B5E7/Pden_2654	0	2	0	1	Efflux transporter, RND family, MFP subunit	Unknown	12,5,13
A1B5T7/Pden_2797	0	2	0	1	Uncharacterized protein	Cytoplasmic	6,8,4,7
A1B3F6/Pden_1958	0	0	2	1	UPF0262 protein	Cytoplasmic	0
A1B6M0/Pden_3083	0	0	2	1	Putative outer membrane protein	OuterMembrane	0
A1BAK1/Pden_4481	0	0	2	1	Enoyl-CoA hydratase/isomerase	Cytoplasmic	0
A1AZS4/Pden_0656	0	1	0	2	Phosphoadenylyl-sulfate reductase (thioredoxin)	Cytoplasmic	1,4,12,14
A1B445/Pden_2197	0	1	0	2	Export-related chaperone CsaA	Cytoplasmic	0
A1B871/Pden_3648	0	1	0	2	Nucleoid-associated protein	Unknown	0
A1B9P1/Pden_4171	0	1	0	2	Uncharacterized protein	Unknown	0
A1BA24/Pden_4304	0	1	0	2	Iron-sulfur cluster assembly accessory protein	Cytoplasmic	1,3
A1AZD4/Pden_0516	0	0	1	2	Sarcosine oxidase, gamma subunit	Unknown	0
A1B484/Pden_2236	0	0	1	2	Carboxymuconolactone decarboxylase	Unknown	0

A1B571/Pden_2578	0	0	1	2	Uncharacterized protein	Unknown	0
A1B5H8/Pden_2685	0	0	1	2	Short chain enoyl-CoA hydratase / Enoyl-CoA hydratase	Membrane	0
A1B5Q6/Pden_2766	0	0	1	2	Secreted periplasmic protein	Unknown	3,12
A1B717/Pden_3230	0	0	1	2	AMP-dependent synthetase and ligase	Cytoplasmic	0
A1B8V5/Pden_3883	0	0	1	2	tRNA (guanine-N(1)-)-methyltransferase	Cytoplasmic	0
A1B8Z1/Pden_3919	0	0	1	2	D-3-hydroxyaspartate aldolase	Cytoplasmic	0
A1B998/Pden_4026	0	0	1	2	Uncharacterized protein	Cytoplasmic	0
A1BBI7/Pden_4820	0	0	1	2	AMP-dependent synthetase and ligase	Cytoplasmic	0
A1B0P5/Pden_0978	0	0	0	3	Uncharacterized protein	Unknown	0
A1B9H1/Pden_4099	0	0	0	3	Cysteine desulfuration protein (SufE)	Cytoplasmic	0
A1B4H9/Pden_2332	0	2	2	0	Cobalamin (Vitamin B12) biosynthesis (CbiX)	Cytoplasmic	1,6,4,7,12,14
A1B8R3/Pden_3841	0	2	2	0	Helicase domain protein	Cytoplasmic	0
A1AZF3/Pden_0535	0	3	0	1	Uncharacterized protein	Membrane	0
A1AZ17/Pden_0397	0	2	1	1	Histone deacetylase superfamily	Cytoplasmic	0
A1AZG1/Pden_0543	0	2	1	1	Dihydroneopterin aldolase	Cytoplasmic	8,6,4,7,12,14
A1AZR4/Pden_0646	0	2	1	1	Peptide deformylase (PDF) (Polypeptide deformylase)	Cytoplasmic	6,1,8,4,7
A1B0W8/Pden_1051	0	2	1	1	Aldehyde dehydrogenase (NAD(+))	Cytoplasmic	0
A1B1T4/Pden_1377	0	2	1	1	Redoxin domain protein	Unknown	0
A1B3K8/Pden_2010	0	2	1	1	Ribosomal protein S12 methylthiotransferase (RimO)	Cytoplasmic	6,8,4,7
A1B6M9/Pden_3092	0	2	1	1	Uncharacterized protein	Cytoplasmic	0
A1BA38/Pden_4318	0	2	1	1	Cytochrome *c* oxidase assembly protein (CtaG)	Unknown	3
A1BB57/Pden_4690	0	2	1	1	Phosphatidyl-*N*-methylethanolamine *N*-methyltransferase	Cytoplasmic	0
A1B3G2/Pden_1964	0	1	2	1	Uncharacterized protein	Cytoplasmic	0
A1BC28/Pden_5012	0	1	2	1	ABC transporter substrate-binding protein	Unknown	0
A1BCC4/Pden_5108	0	1	2	1	Cytochrome ba_3 quinol oxidase subunit 2	Membrane	4,12,14
A1B0C6/Pden_0858	0	0	3	1	Pyridoxine/pyridoxamine 5'-phosphate oxidase	Cytoplasmic	1,6,4,7,12,14
A1B2B7/Pden_1561	0	2	0	2	Diaminohydroxyphosphoribosylaminopyrimidine deaminase / 5-amino-6-(5-phosphoribosylamino)uracil reductase	Cytoplasmic	1,6,4,7,12,14
A1AZV6/Pden_0688	0	1	1	2	Uncharacterized protein	Unknown	0
A1B004/Pden_0736	0	1	1	2	Glyoxalase/bleomycin resistance protein/dioxygenase	Unknown	0
A1B121/Pden_1107	0	1	1	2	Alcohol dehydrogenase GroES domain protein	Cytoplasmic	0
A1B313/Pden_1810	0	1	1	2	Uncharacterized protein	Unknown	0
A1B3I3/Pden_1985	0	1	1	2	Putative pterin-4-alpha-carbinolamine dehydratase (PHS)	Cytoplasmic	6,1,4,7
A1B436/Pden_2188	0	1	1	2	Uncharacterized protein	Membrane	0
A1B875/Pden_3652	0	1	1	2	ABC transporter related protein	Cytoplasmic	0
A1B8D0/Pden_3707	0	1	1	2	Uncharacterized protein	Unknown	0
A1B8N0/Pden_3808	0	1	1	2	Uncharacterized protein	Unknown	0
A1B8U9/Pden_3877	0	1	1	2	Alkyl hydroperoxide reductase (AhpD)	Unknown	0
A1B953/Pden_3981	0	1	1	2	Uncharacterized protein	Membrane	0
A1B9T2/Pden_4212	0	1	1	2	Uncharacterized protein	Membrane	0
A1BA46/Pden_4326	0	1	1	2	Phosphate-specific transport system accessory protein (PhoU)	Cytoplasmic	12,9,5,13,10
A1B0C8/Pden_0860	0	0	1	3	Uncharacterized protein	Unknown	0
A1AZD0/Pden_0512	0	5	0	0	Sarcosine oxidase, beta subunit family	Cytoplasmic	8,6,4,7,12,14
A1B281/Pden_1525	0	3	1	1	Uncharacterized protein	Membrane	0

A1B8Y0/Pden_3908	0	3	1	1	Uncharacterized protein	Unknown	0
A1AZ47/Pden_0427	0	2	2	1	Uncharacterized protein	Membrane	0
A1B282/Pden_1526	0	2	2	1	Helicase domain protein	Cytoplasmic	0
A1B3I6/Pden_1988	0	2	2	1	FolC bifunctional protein	Cytoplasmic	0
A1B577/Pden_2584	0	2	2	1	SAM-dependent methyltransferase	Cytoplasmic	0
A1B3B0/Pden_1909	0	1	3	1	Uncharacterized protein	Cytoplasmic	0
A1BBI6/Pden_4819	0	1	3	1	Thiolase	Unknown	0
A1B1L6/Pden_1305	0	2	1	2	Antibiotic biosynthesis monooxygenase	Unknown	0
A1B4Z3/Pden_2500	0	2	1	2	Uncharacterized protein	Membrane	0
A1B7Y0/Pden_3554	0	2	1	2	Uncharacterized protein	Cytoplasmic	0
A1B9B5/Pden_4043	0	2	1	2	ErfK/YbiS/YcfS/YnhG family protein	Unknown	0
A1AZS2/Pden_0654	0	1	2	2	Uncharacterized protein	Unknown	0
A1B3E5/Pden_1946	0	1	1	3	Deoxyuridine 5'-triphosphate nucleotidohydrolase (dUTPase)	Cytoplasmic	8,6,4,7,12,14
A1B415/Pden_2167	0	3	2	1	Glutamate-ammonia-ligase adenylyltransferase	Cytoplasmic	0
A1B878/Pden_3655	0	3	1	2	Glycosyl transferase, group 1	Cytoplasmic	0
A1B392/Pden_1891	0	2	2	2	Queuine tRNA-ribosyltransferase	Cytoplasmic	8,1,4,7,12,14
A1B4C2/Pden_2274	0	2	2	2	50S ribosomal protein (L35)	Unknown	6,1,8,4,7
Q51699/Pden_2486	0	2	2	2	Protein (NirI)	Membrane	9
A1B7L4/Pden_3437	0	2	2	2	Fumarylacetoacetate (FAA) hydrolase	Cytoplasmic	0
A1B885/Pden_3662	0	2	2	2	Riboflavin biosynthesis protein	Cytoplasmic	8,1,4,7,12,14
A1BC00/Pden_4984	0	2	2	2	Transketolase, central region	Cytoplasmic	0
A1B600/Pden_2860	0	2	1	3	Phosphoribosylformylglycinamidine synthase subunit (PurS)	Unknown	8,1,4,7,12,14
A1B0L7/Pden_0950	0	0	2	4	Fervidolysin, Serine peptidase, MEROPS family S08A	Cytoplasmic	0
A1B359/Pden_1856	0	2	3	2	Histidine triad (HIT) protein	Cytoplasmic	0
A1B4S4/Pden_2431	0	2	2	3	LAO/AO transport system ATPase	Membrane	0
A1AXX7/Pden_0004	0	3	3	2	tRNA uridine 5-carboxymethylaminomethyl modification enzyme (MnmG)	Cytoplasmic	6,8,4,7
A1B9F0/Pden_4078	0	3	2	3	Methylenetetrahydrofolate--tRNA-(uracil-5-)-methyltransferase (TrmFO)	Cytoplasmic	0
A1BB78/Pden_4711	0	5	1	3	Cell division topological specificity factor	Cytoplasmic	12,9
A1B3K3/Pden_2005	0	2	4	3	AMP-dependent synthetase and ligase	Cytoplasmic	0
A1B3X6/Pden_2128	0	2	3	4	Uncharacterized protein	Unknown	0
A1B3Z0/Pden_2142	0	4	3	3	LexA repressor	Cytoplasmic	12,7,14,9,13,29
A1B3F0/Pden_1952	0	3	4	4	Glucose-6-phosphate 1-dehydrogenase	Cytoplasmic	8,6,4,7,12,14
A1B382/Pden_1881	0	4	2	5	Cell division protein (ZapA)	Unknown	3,12
A1AZQ1/Pden_0633	0	5	3	4	Thiamine pyrophosphate enzyme TPP binding domain protein	Cytoplasmic	0
A1B3J9/Pden_2001	0	4	2	6	Uncharacterized protein	Cytoplasmic	0
A1BAH1/Pden_4451	0	3	5	6	Assimilatory nitrite reductase (NAD(P)H) small subunit	Unknown	6,4,7,12,14
A1B6L9/Pden_3082	0	6	5	8	Autotransporter barrel domain	OuterMembrane	0

[1]Protein annotated from UniProt (UP000000361). [2]Genes annotated from GeneBank (T00440). [3]Subcellular location according with PSOTb v3.0.2. [4]GOi (Gene Ontology respect to biological process at the third level), numbers refer to 0: unknown, 1: biosynthetic process, 2: catabolic process, 3: cellular component organization, 4: cellular metabolic process, 5: establishment of localization, 6: nitrogen compound metabolic process, 7: organic substance metabolic process, 8: primary metabolic process, 9: regulation of biological process, 10: regulation of biological quality, 11: response to

stress, 12: single-organism cellular process, 13: single-organism localization, 14: single-organism metabolic process and 15: carbon utilization, 16: anatomical structure formation involved in morphogenesis, 17: anatomical structure morphogenesis, 18: cell adhesion, 19: cell wall organization or biogenesis, 20: cellular component biogenesis, 21: cellular detoxification, 22: cellular localization, 23: macromolecule localization, 24: methylation, 25: protein folding, 26: protein unfolding, 27: response to abiotic stimulus, 28: response to biotic stimulus, 29: response to external stimulus, 30: single organism reproductive process, 31: single-organism developmental process and 32: response to chemicals.
