# Supplementary material for: Exploring the Denitrification Proteome of Paracoccus denitrificans PD1222
Source: Front Microbiol. 2018 May 29;9:1137. doi: 10.3389/fmicb.2018.01137 (PMC5987163; doi:10.3389/fmicb.2018.01137)
Supplement: Supplementary file 2 [file Table_2.PDF]

Table S2. *P. denitrificans* PD1222 proteins identified from cells grown under anaerobic conditions with nitrate and ammonium.

Protein[1]/Gene[2]	R1	R2	R3	R4	Protein names	Subcellular location[3]	GO[4]
A1AXX5/Pden_0002	27	28	23	26	Transcription termination factor Rho	Cytoplasmic	1,6,8,4,7,9
A1AXX6/Pden_0003	3	2	1	1	tRNA modification GTPase (MnmE)	Cytoplasmic	6,8,4,7
A1AXX7/Pden_0004	1	2	4	1	tRNA uridine 5-carboxymethylaminomethyl modification enzyme (MnmG)	Cytoplasmic	6,8,4,7
A1AXX9/Pden_0006	3	5	3	5	Cobyrinic acid a,c-diamide synthase	Membrane	0
A1AXY0/Pden_0007	13	8	11	9	ParB-like partition protein	Cytoplasmic	0
A1AXY1/Pden_0008	6	6	4	6	Protein GrpE	Cytoplasmic	25
A1AXY2/Pden_0009	2	1	2	1	Heat-inducible transcription repressor (HrcA)	Membrane	1,6,8,4,7,9
A1AXY3/Pden_0010	6	6	7	7	Ribonuclease (PH)	Cytoplasmic	6,8,4,7
A1AXY4/Pden_0011	4	6	1	5	Non-canonical purine NTP pyrophosphatase	Cytoplasmic	4,8,7,12,14,2
A1AXY9/Pden_0016	7	6	5	7	S-glutathione dehydrogenase	Cytoplasmic	7,14
A1AXZ0/Pden_0017	11	10	10	10	ATP-dependent Clp protease proteolytic subunit	Cytoplasmic	0
A1AXZ2/Pden_0019	8	8	9	8	S-formylglutathione hydrolase	Unknown	4,7,12,14,2
A1AY07/Pden_0034	3	5	4	3	ABC transporter related protein	Membrane	0
A1AY08/Pden_0035	7	7	10	7	Aminopyrimidine aminohydrolase	Unknown	6,4,7,12,14
A1AY09/Pden_0036	13	11	10	12	NlpA lipoprotein	Unknown	1,6,4,7,12,14
A1AY10/Pden_0037	5	5	5	5	UBA/THIF-type NAD/FAD binding protein	Membrane	0
A1AY11/Pden_0038	6	9	9	7	Thiamine-phosphate diphosphorylase	Cytoplasmic	1,6,4,7,12,14
A1AY12/Pden_0039	10	14	13	14	Thiazole synthase	Cytoplasmic	6,1,4,7
A1AY14/Pden_0041	17	17	17	16	Glycine oxidase	Cytoplasmic	0
A1AY15/Pden_0042	9	8	7	10	Hydroxymethylpyrimidine kinase / phosphomethylpyrimidine kinase	Unknown	1,6,4,7,12,14
A1AY16/Pden_0043	1	2	4	2	HAD-superfamily hydrolase, subfamily IA, variant 3	Unknown	0
A1AY25/Pden_0052	7	7	8	7	Amino acid/amide ABC transporter substrate-binding protein, HAAT family	Unknown	0
A1AY30/Pden_0057	9	7	11	7	SPFH domain, Band 7 family protein	Cytoplasmic	0
A1AY32/Pden_0059	0	0	1	2	Glutathione S-transferase, N-terminal domain	Membrane	0
A1AY33/Pden_0060	5	6	6	8	Orotidine 5'-phosphate decarboxylase	Cytoplasmic	8,1,4,7,12,14
A1AY35/Pden_0062	12	12	11	13	Chaperone protein (ClpB)	Cytoplasmic	11,8,7,27
A1AY45/Pden_0072	2	1	1	0	CHAD domain containing protein	Cytoplasmic	0
A1AY46/Pden_0073	6	7	6	7	Cobyrinic acid a,c-diamide synthase	Membrane	0
A1AY60/Pden_0087	3	5	3	7	Acylneuraminate cytidylyltransferase	Cytoplasmic	0
A1AY61/Pden_0088	3	2	6	3	Flagellin modification protein (FlmD)	Cytoplasmic	0
A1AY62/Pden_0089	17	16	19	20	Uncharacterized protein	Cytoplasmic	0
A1AY63/Pden_0090	19	21	17	19	Uncharacterized protein	Cytoplasmic	1
A1AY64/Pden_0091	2	1	3	3	Uncharacterized protein	Cytoplasmic	0
A1AY66/Pden_0093	29	26	30	27	Polysaccharide biosynthesis protein (CapD)	Cytoplasmic	0
A1AY85/Pden_0112	0	0	2	1	Uncharacterized protein	Cytoplasmic	0
A1AYE1/Pden_0169	1	2	3	3	Regulatory protein (IclR)	Cytoplasmic	1,6,8,4,7,9
A1AYG0/Pden_0188	1	2	0	2	Regulatory protein (IclR)	Cytoplasmic	1,6,8,4,7,9
A1AYG9/Pden_0197	4	3	3	2	3-hydroxyacyl-CoA dehydrogenase	Unknown	4,8,7,12,14,2
A1AYH6/Pden_0206	2	3	1	2	Short-chain dehydrogenase/reductase (SDR)	Cytoplasmic	0
A1AYK1/Pden_0231	1	0	0	3	Transcriptional regulator, LysR family	Cytoplasmic	6,1,8,4,7
A1AYL3/Pden_0243	1	1	2	3	Succinate semialdehyde dehydrogenase	Cytoplasmic	0

A1AYM0/Pden_0250	1	3	0	1	Transcriptional regulator, RpiR family	Cytoplasmic	8,7
A1AYM5/Pden_0255	6	5	7	7	Uncharacterized protein	Cytoplasmic	0
A1AYS8/Pden_0308	4	5	5	5	Uncharacterized protein UPF0065	Periplasmic	0
A1AYU0/Pden_0320	0	2	0	1	UPF0271 protein	Unknown	8,7
A1AYU3/Pden_0323	2	4	5	2	Biotin carboxyl carrier protein / biotin carboxylase	Cytoplasmic	0
A1AYU5/Pden_0325	3	3	4	4	Uncharacterized protein	Unknown	0
A1AYU6/Pden_0326	0	0	2	0	Luciferase family protein	Unknown	0
A1AYV3/Pden_0333	7	9	7	10	Tryptophan synthase alpha chain	Cytoplasmic	0
A1AYV4/Pden_0334	17	13	12	18	Ribosome-binding ATPase (YchF)	Unknown	0
A1AYW7/Pden_0347	2	2	2	2	Uncharacterized protein	Unknown	0
A1AZ13/Pden_0393	1	2	3	3	Uncharacterized protein	Unknown	0
A1AZ15/Pden_0395	1	3	2	4	Diaminopimelate epimerase	Cytoplasmic	8,1,4,7,12,14
A1AZ19/Pden_0399	4	4	4	5	Farnesyl-diphosphate synthase	Cytoplasmic	8,1,4,7,12,14
A1AZ20/Pden_0400	2	4	3	4	1-deoxy-D-xylulose-5-phosphate synthase	Cytoplasmic	8,1,4,7,12,14
A1AZ21/Pden_0401	3	4	1	2	SH3, type 3 domain protein	Membrane	0
A1AZ24/Pden_0404	2	2	1	1	AMP nucleosidase	Cytoplasmic	8,1,4,7,12,14
A1AZ25/Pden_0405	8	9	8	9	Histone family protein DNA-binding protein	Cytoplasmic	0
A1AZ36/Pden_0416	21	22	24	21	DNA gyrase subunit B	Cytoplasmic	1,6,8,3,4,7
A1AZ38/Pden_0418	5	3	4	2	GTPase Era	Membrane	20
A1AZ39/Pden_0419	0	2	2	3	Ribonuclease 3	Cytoplasmic	6,8,4,7,2
A1AZ40/Pden_0420	4	4	4	4	Signal peptidase I	Membrane	0
A1AZ42/Pden_0422	3	4	5	6	Pyridoxine 5'-phosphate synthase	Cytoplasmic	1,6,4,7,12,14
A1AZ44/Pden_0424	4	6	5	9	Tetratricopeptide TPR_2 repeat protein	Unknown	0
A1AZ45/Pden_0425	15	16	14	12	Electron-transferring-flavoprotein dehydrogenase	Unknown	0
A1AZ46/Pden_0426	7	7	10	9	Transcription elongation factor GreA	Cytoplasmic	1,6,8,4,7,9
A1AZ47/Pden_0427	1	1	4	1	Uncharacterized protein	Membrane	0
A1AZ51/Pden_0431	6	9	10	10	Uncharacterized protein	Unknown	0
A1AZ52/Pden_0432	1	2	2	2	aa_3-type cytochrome *c* oxidase subunit IV	Membrane	0
A1AZ54/Pden_0434	10	7	10	11	Acyl-CoA dehydrogenase domain protein	Cytoplasmic	0
A1AZ55/Pden_0435	3	2	2	2	Threonylcarbamoyl-AMP synthase	Unknown	6,8,4,7
A1AZ56/Pden_0436	2	1	2	1	UPF0301 protein Pden_0436	Cytoplasmic	0
A1AZ60/Pden_0440	17	17	19	16	3-isopropylmalate dehydrogenase	Cytoplasmic	8,1,4,7,12,14
A1AZ63/Pden_0443	1	2	2	2	Uncharacterized protein	Cytoplasmic	0
A1AZ64/Pden_0444	2	2	2	3	Uncharacterized protein	Cytoplasmic	0
P50204/Pden_0474	13	15	17	17	Acetoacetyl-CoA reductase	Unknown	0
A1AZA6/Pden_0488	46	40	38	34	Glutamate synthase (large subunit)	Unknown	8,1,4,7,12,14
A1AZA8/Pden_0490	9	14	12	13	Glutamate synthase (small subunit)	Cytoplasmic	8,1,4,7,12,14
A1AZB0/Pden_0492	13	12	13	12	NAD-dependent epimerase/dehydratase	Cytoplasmic	0
A1AZB5/Pden_0497	6	7	7	6	L-threonine dehydratase	Cytoplasmic	8,1,4,7,12,14
A1AZB7/Pden_0499	27	25	26	26	Argininosuccinate synthase	Cytoplasmic	8,1,4,7,12,14
A1AZB9/Pden_0501	1	2	1	1	Molybdopterin adenylyltransferase	Cytoplasmic	6,1,8,4,7
A1AZC3/Pden_0505	2	2	2	2	Mannose-binding protein / fructose-binding protein / ribose-binding protein	Periplasmic	0
A1AZC5/Pden_0507	3	1	2	3	Chaperone DnaJ domain protein	Cytoplasmic	25
A1AZC9/Pden_0511	7	5	12	8	Putative cytochrome *c*-type biogenesis protein (CycH)	Unknown	0
A1AZD3/Pden_0515	5	2	6	5	Sarcosine oxidase, alpha subunit family	Cytoplasmic	8,6,4,7,12,14
A1AZD6/Pden_0518	11	11	13	11	Superoxide dismutase	Periplasmic	0

A1AZE0/Pden_0522	8	10	9	12	ErfK/YbiS/YcfS/YnhG family protein	Unknown	0
A1AZE6/Pden_0528	9	9	8	6	Nucleoside-binding protein	Unknown	0
A1AZE9/Pden_0531	5	5	6	6	Nitrogen-fixing NifU domain protein	Unknown	1,3
A1AZF5/Pden_0537	4	4	3	4	Peptidoglycan-binding domain 1 protein	Cytoplasmic	0
A1AZF6/Pden_0538	5	4	4	5	Glycine--tRNA ligase alpha subunit	Cytoplasmic	8,6,4,7,12,14
A1AZF7/Pden_0539	2	1	2	3	Signal transduction histidine kinase regulating citrate/malate metabolism	Unknown	0
A1AZF8/Pden_0540	18	13	14	14	Glycine--tRNA ligase beta subunit	Cytoplasmic	8,6,4,7,12,14
A1AZG1/Pden_0543	0	2	2	1	Dihydroneopterin aldolase	Cytoplasmic	8,6,4,7,12,14
A1AZG3/Pden_0545	6	5	5	7	Phosphoglucosamine mutase	Cytoplasmic	8,7
A1AZG5/Pden_0547	7	6	7	7	RNA polymerase-binding transcription factor (DksA)	Cytoplasmic	9
A1AZG6/Pden_0548	5	5	5	4	ATPase associated with various cellular activities, AAA_5	Cytoplasmic	0
A1AZG9/Pden_0551	34	38	35	32	Dihydrolipoyl dehydrogenase	Cytoplasmic	12,7,14,9,2,10
A1AZH1/Pden_0553	0	0	2	0	Uncharacterized protein	Unknown	0
A1AZH2/Pden_0554	31	28	28	28	Dihydrolipoyllysine-residue succinyltransferase component of 2-oxoglutarate dehydrogenase complex	Cytoplasmic	4,8,7,12,14,2
A1AZH3/Pden_0555	46	48	37	44	2-oxoglutarate dehydrogenase E1 component	Cytoplasmic	8,4,7,12,14
A1AZH6/Pden_0558	24	21	23	22	Succinate--CoA ligase [ADP-forming] subunit alpha	Cytoplasmic	0
A1AZH7/Pden_0559	42	40	40	40	Succinate--CoA ligase [ADP-forming] subunit beta	Cytoplasmic	8,4,7,12,14
A1AZH9/Pden_0561	21	21	22	21	Malate dehydrogenase	Cytoplasmic	8,4,7,12,14
A1AZI1/Pden_0563	6	8	7	6	Cityl-CoA lyase	Cytoplasmic	0
A1AZI4/Pden_0566	13	12	11	11	L-erythro-3-methylmalyl-CoA dehydratase	Cytoplasmic	0
A1AZI5/Pden_0567	2	2	1	2	Succinate dehydrogenase subunit C	Membrane	8,4,7,12,14
A1AZI7/Pden_0569	37	36	39	37	Succinate dehydrogenase flavoprotein subunit	Membrane	8,4,7,12,14
A1AZJ0/Pden_0572	11	11	10	13	Succinate dehydrogenase iron-sulfur subunit	Membrane	8,4,7,12,14
A1AZJ2/Pden_0574	4	4	3	4	UPF0173 metal-dependent hydrolase Pden_0574	Cytoplasmic	0
A1AZJ4/Pden_0576	12	12	12	9	Glutamyl-tRNA amidotransferase subunit A	Cytoplasmic	6,1,8,4,7
A1AZJ5/Pden_0577	2	4	2	3	Uncharacterized protein	Unknown	0
A1AZJ9/Pden_0581	6	2	8	6	Iron-sulfur cluster carrier protein	Cytoplasmic	0
A1AZK0/Pden_0582	2	2	2	2	Transcriptional regulator (MraZ)	Cytoplasmic	6,1,8,4,7
A1AZK1/Pden_0583	5	6	3	5	Ribosomal RNA small subunit methyltransferase H (C1402 methyltransferase, RsmH)	Cytoplasmic	6,8,4,7,24
A1AZK4/Pden_0586	10	8	9	9	UDP-N-acetylmuramoyl-L-alanyl-D-glutamate--2,6-diaminopimelate ligase	Cytoplasmic	12,7,14,9,10,19
A1AZK5/Pden_0587	7	7	7	9	UDP-N-acetylmuramoyl-tripeptide--D-alanyl-D-alanine ligase	Cytoplasmic	12,7,14,9,10,19
A1AZK6/Pden_0588	0	0	1	2	Phospho-N-acetylmuramoyl-pentapeptide-transferase	Membrane	12,7,14,9,10,19
A1AZK7/Pden_0589	13	12	13	7	UDP-N-acetylmuramoylalanine--D-glutamate ligase	Unknown	12,7,14,9,10,19
A1AZK8/Pden_0590	5	6	7	2	Membrane protein involved in aromatic hydrocarbon degradation	OuterMembrane	0
A1AZL0/Pden_0592	33	29	27	26	Inosine-5'-monophosphate dehydrogenase	Cytoplasmic	8,1,4,7,12,14
A1AZL2/Pden_0594	3	3	2	2	Fmu domain protein	Cytoplasmic	0
A1AZL5/Pden_0597	8	9	8	10	Protein RecA	Cytoplasmic	7,4,12,14,13,29
A1AZL6/Pden_0598	40	51	44	46	Alanine--tRNA ligase	Cytoplasmic	8,6,4,7,12,14
A1AZL7/Pden_0599	1	5	5	5	Uncharacterized protein	Unknown	0
A1AZM1/Pden_0603	21	22	18	18	Adenylosuccinate synthetase	Cytoplasmic	8,1,4,7,12,14
A1AZM6/Pden_0608	17	16	17	16	Extracellular solute-binding protein, family 5	Periplasmic	0
A1AZM8/Pden_0610	12	12	10	14	Amidohydrolase	Cytoplasmic	0
A1AZM9/Pden_0611	29	26	27	23	Dihydrolipoyl dehydrogenase	Cytoplasmic	12,7,14,9,2,10
A1AZN0/Pden_0612	1	1	2	1	Uncharacterized protein	Unknown	0

A1AZN2/Pden_0614	1	2	1	0	*S*-adenosylmethionine:tRNA ribosyltransferase-isomerase	Cytoplasmic	8,1,4,7,12,14
A1AZN4/Pden_0616	11	12	11	11	Bacterioferritin	Cytoplasmic	12,5,13,10
A1AZN5/Pden_0617	0	1	2	4	HAD-superfamily hydrolase, subfamily IA, variant 3	Cytoplasmic	0
A1AZN6/Pden_0618	16	16	15	14	Bifunctional protein (GlmU) [UDP-*N*-acetylglucosamine pyrophosphorylase; Glucosamine-1-phosphate *N*-acetyltransferase]	Cytoplasmic	9,14,31,17,10,19
A1AZN7/Pden_0619	24	24	23	28	Glutamine--fructose-6-phosphate aminotransferase [isomerizing]	Cytoplasmic	8,1,4,7,12,14
A1AZN9/Pden_0621	3	3	4	2	Peptide methionine sulfoxide reductase (MsrA), *S*-oxide reductase	Unknown	11,8,4,7
A1AZP0/Pden_0622	5	4	2	2	Ribosomal protein L11 methyltransferase	Cytoplasmic	0
A1AZP2/Pden_0624	1	2	1	1	Ribosomal RNA small subunit methyltransferase E	Cytoplasmic	6,8,4,7
A1AZP4/Pden_0626	8	7	7	4	OmpA/MotB domain protein	Membrane	0
A1AZP8/Pden_0630	26	22	22	24	Aromatic amino acid aminotransferase apoenzyme	Cytoplasmic	8,1,4,7,12,14
A1AZP9/Pden_0631	12	9	9	13	3-mercaptopyruvate sulfurtransferase	Cytoplasmic	0
A1AZQ1/Pden_0633	2	3	2	3	Thiamine pyrophosphate enzyme TPP binding domain protein	Cytoplasmic	0
A1AZQ7/Pden_0639	1	0	2	2	Uncharacterized protein	Cytoplasmic	0
A1AZR0/Pden_0642	1	2	0	1	Methionyl-tRNA formyltransferase	Cytoplasmic	0
A1AZR6/Pden_0648	4	8	6	9	Aminotransferase, class I and II	Cytoplasmic	1
A1AZS0/Pden_0652	3	3	3	3	Aminotransferase	Cytoplasmic	1
A1AZS1/Pden_0653	1	0	2	1	Transcriptional regulator, AsnC family	Cytoplasmic	6,1,8,4,7
A1AZS2/Pden_0654	2	2	2	1	Uncharacterized protein	Unknown	0
A1AZS3/Pden_0655	22	16	15	16	Nitrite/sulfite reductase, hemoprotein beta-component, ferrodoxin domain protein	Cytoplasmic	0
A1AZS4/Pden_0656	2	4	3	3	Phosphoadenylyl-sulfate reductase	Cytoplasmic	1,4,12,14
A1AZS5/Pden_0657	1	1	3	1	Uncharacterized protein	Unknown	0
A1AZS6/Pden_0658	23	21	17	19	Ferredoxin--NADP reductase	Cytoplasmic	0
A1AZS7/Pden_0659	24	23	23	26	DNA topoisomerase 1	Cytoplasmic	6,8,3,4,7
A1AZT2/Pden_0664	12	6	11	10	TRAP transporter solute receptor, TAXI family	Unknown	0
A1AZU8/Pden_0680	1	0	0	2	Transcriptional regulator, GntR family	Cytoplasmic	6,1,8,4,7
A1AZV1/Pden_0683	3	4	3	4	Cell division and transport-associated protein (TolQ)	Membrane	12
A1AZV3/Pden_0685	2	6	1	6	Cell division and transport-associated protein (TolA)	OuterMembrane	12
A1AZV4/Pden_0686	15	16	14	15	Protein TolB	Periplasmic	5,13,23
A1AZV5/Pden_0687	6	5	7	5	OmpA domain protein	OuterMembrane	0
A1AZV6/Pden_0688	2	2	2	2	Uncharacterized protein	Unknown	0
A1AZV8/Pden_0690	16	22	20	19	ATP-dependent zinc metalloprotease (FtsH)	Membrane	8,7,2
A1AZX2/Pden_0704	14	12	13	13	4-hydroxy-tetrahydrodipicolinate synthase	Cytoplasmic	8,1,4,7,12,14
A1AZX5/Pden_0707	8	8	7	9	Inositol monophosphatase	Cytoplasmic	4,8,7,12,14,2
A1AZX7/Pden_0709	2	2	1	1	Formiminoglutamate deiminase	Cytoplasmic	0
A1AZX9/Pden_0711	5	4	2	1	Histidine ammonia-lyase	Cytoplasmic	4,8,7,12,14,2
A1AZY3/Pden_0715	3	6	2	2	Urocanate hydratase	Cytoplasmic	4,8,7,12,14,2
A1AZY4/Pden_0716	11	10	11	11	Amino acid ABC transporter substrate-binding protein, PAAT family	Periplasmic	0
A1AZY8/Pden_0720	5	4	3	7	NADH:flavin oxidoreductase/NADH oxidase	Cytoplasmic	0
A1AZY9/Pden_0721	1	3	2	2	Patatin	Cytoplasmic	8,7,14
A1AZZ1/Pden_0723	1	2	2	2	Uncharacterized protein	Membrane	0
A1AZZ3/Pden_0725	3	2	2	1	TonB family protein	Unknown	5
A1AZZ5/Pden_0727	4	5	3	5	Outer membrane transport energization protein (ExbB)	Membrane	0
A1AZZ7/Pden_0729	3	3	4	2	Aminotransferase, class V	Cytoplasmic	0

A1AZZ8/Pden_0730	2	3	1	4	ABC transporter related protein	Membrane	0
A1AZZ9/Pden_0731	3	3	3	2	Glycine cleavage T protein	Cytoplasmic	0
A1B002/Pden_0734	54	56	53	54	Elongation factor Tu	Cytoplasmic	0
A1B010/Pden_0742	10	10	10	11	Transcription termination/antitermination protein (NusG)	Cytoplasmic	1,6,8,4,7,9
A1B011/Pden_0743	9	8	9	8	50S ribosomal protein L11	Cytoplasmic	6,1,8,4,7
A1B012/Pden_0744	22	21	20	21	50S ribosomal protein L1	Cytoplasmic	1,6,8,4,7,9
A1B013/Pden_0745	5	6	6	6	50S ribosomal protein L10	Cytoplasmic	1,6,8,4,7,20
A1B014/Pden_0746	13	12	12	13	50S ribosomal protein L7/L12	Unknown	6,1,8,4,7
A1B015/Pden_0747	79	73	72	78	DNA-directed RNA polymerase subunit beta	Cytoplasmic	6,1,8,4,7
A1B017/Pden_0749	63	66	63	68	DNA-directed RNA polymerase subunit beta	Cytoplasmic	6,1,8,4,7
A1B021/Pden_0753	8	8	9	9	30S ribosomal protein S12	Cytoplasmic	6,1,8,4,7
A1B022/Pden_0754	13	12	14	15	30S ribosomal protein S7	Cytoplasmic	6,1,8,4,7
A1B023/Pden_0755	60	62	59	59	Elongation factor G	Cytoplasmic	0
A1B026/Pden_0758	9	7	8	10	30S ribosomal protein S10	Cytoplasmic	6,1,8,4,7
A1B027/Pden_0759	20	17	19	16	50S ribosomal protein L3	Cytoplasmic	6,1,8,4,7
A1B028/Pden_0760	21	21	19	20	50S ribosomal protein L4	Cytoplasmic	6,1,8,4,7
A1B029/Pden_0761	6	8	7	8	50S ribosomal protein L23	Cytoplasmic	6,1,8,4,7
A1B030/Pden_0762	23	21	21	19	50S ribosomal protein L2	Cytoplasmic	6,1,8,4,7
A1B031/Pden_0763	10	10	10	9	30S ribosomal protein S19	Cytoplasmic	6,1,8,4,7
A1B032/Pden_0764	9	11	11	9	50S ribosomal protein L22	Cytoplasmic	6,1,8,4,7
A1B033/Pden_0765	18	19	17	18	30S ribosomal protein S3	Cytoplasmic	6,1,8,4,7
A1B034/Pden_0766	6	7	6	6	50S ribosomal protein L16	Cytoplasmic	6,1,8,4,7
A1B036/Pden_0768	8	8	10	5	30S ribosomal protein S17	Cytoplasmic	6,1,8,4,7
A1B037/Pden_0769	9	8	10	12	50S ribosomal protein L14	Cytoplasmic	6,1,8,4,7
A1B038/Pden_0770	9	7	10	8	50S ribosomal protein L24	Cytoplasmic	6,1,8,4,7
A1B039/Pden_0771	13	14	14	15	50S ribosomal protein L5	Cytoplasmic	6,1,8,4,7
A1B040/Pden_0772	7	4	6	7	30S ribosomal protein S14	Cytoplasmic	6,1,8,4,7
A1B041/Pden_0773	9	9	11	11	30S ribosomal protein S8	Cytoplasmic	6,1,8,4,7
A1B042/Pden_0774	13	16	13	15	50S ribosomal protein L6	Cytoplasmic	6,1,8,4,7
A1B043/Pden_0775	7	6	4	11	50S ribosomal protein L18	Cytoplasmic	6,1,8,4,7
A1B044/Pden_0776	14	13	15	16	30S ribosomal protein S5	Cytoplasmic	6,1,8,4,7
A1B045/Pden_0777	1	0	2	1	50S ribosomal protein L30	Unknown	6,1,8,4,7
A1B047/Pden_0779	12	9	10	14	50S ribosomal protein L15	Cytoplasmic	6,1,8,4,7
A1B048/Pden_0780	3	2	1	0	Protein translocase subunit (SecY)	Membrane	12,5,13,23
A1B049/Pden_0781	16	16	17	18	Adenylate kinase	Cytoplasmic	8,1,4,7,12,14
A1B050/Pden_0782	11	10	15	11	30S ribosomal protein S13	Cytoplasmic	6,1,8,4,7
A1B051/Pden_0783	10	11	11	11	30S ribosomal protein S11	Cytoplasmic	6,1,8,4,7
A1B052/Pden_0784	22	21	21	22	DNA-directed RNA polymerase subunit alpha	Cytoplasmic	6,1,8,4,7
A1B053/Pden_0785	14	12	13	14	50S ribosomal protein L17	Cytoplasmic	6,1,8,4,7
A1B054/Pden_0786	3	4	2	5	Transcriptional regulator, LuxR family	Unknown	9
A1B058/Pden_0790	2	3	3	5	Pseudouridine synthase	Cytoplasmic	6,8,4,7
A1B060/Pden_0792	2	3	2	2	ATP12, ATPase	Unknown	3
A1B061/Pden_0793	20	20	17	19	L-aspartate-binding protein / L-glutamate-binding protein / L-glutamine-binding protein / L-asparagine-binding protein	Unknown	0
A1B062/Pden_0794	0	1	2	1	L-asparagine/L-glutamine/L-glutamate/L-aspartate ABC transporter membrane protein	Membrane	0

A1B064/Pden_0796	7	8	9	8	L-aspartate/L-glutamine/L-glutamate/L-asparagine ABC transporter ATP-binding protein	Membrane	0
A1B066/Pden_0798	12	8	13	13	Branched chain amino acid: 2-keto-4-methylthiobutyrate aminotransferase	Cytoplasmic	0
A1B067/Pden_0799	12	12	10	11	Citrate -lyase	Cytoplasmic	0
A1B075/Pden_0807	11	12	9	10	ErfK/YbiS/YcfS/YnhG family protein	Unknown	0
A1B076/Pden_0808	2	3	2	1	Uncharacterized protein	Unknown	0
A1B078/Pden_0810	2	1	1	3	Extracellular solute-binding protein, family 5	Periplasmic	12,5,13
A1B080/Pden_0812	10	8	8	7	Phosphoserine phosphatase	Cytoplasmic	8,1,4,7,12,14
A1B081/Pden_0813	23	17	19	21	Phosphoserine aminotransferase apoenzyme	Cytoplasmic	8,1,4,7,12,14
A1B082/Pden_0814	33	32	32	30	D-3-phosphoglycerate dehydrogenase	Cytoplasmic	8,1,4,7,12,14
A1B091/Pden_0823	8	6	7	6	Uncharacterized protein	Cytoplasmic	0
A1B092/Pden_0824	7	4	6	5	Uncharacterized protein	Unknown	0
A1B093/Pden_0825	1	2	0	2	Transglutaminase, N-terminal domain protein	Cytoplasmic	0
A1B094/Pden_0826	1	0	1	2	20S proteasome, A and B subunits	Cytoplasmic	8,7
A1B095/Pden_0827	6	3	4	5	DNA mismatch repair protein (MutS)	Cytoplasmic	4,8,7,12,14,13
A1B096/Pden_0828	3	1	3	2	Hydratase/decarboxylase	Membrane	0
A1B097/Pden_0829	9	8	9	10	Extracellular solute-binding protein, family 1	Unknown	0
A1B0A0/Pden_0832	1	3	2	2	ABC transporter related protein	Membrane	0
A1B0A2/Pden_0834	2	4	3	3	Glycosyl transferase, WecB/TagA/CpsF family	Cytoplasmic	1
A1B0A4/Pden_0836	1	1	2	1	Beta-ketoacyl synthase	Membrane	1
A1B0A6/Pden_0838	4	3	11	6	Amino acid adenylation domain	Membrane	1
A1B0B2/Pden_0844	1	2	0	1	Glycosyl transferase, family 2	Cytoplasmic	0
A1B0B3/Pden_0845	2	2	2	3	ATPases involved in chromosome partitioning-like protein	Unknown	0
A1B0B4/Pden_0846	5	6	5	3	Lipopolysaccharide biosynthesis	Membrane	8,1,4,7,12,14
A1B0B6/Pden_0848	22	25	21	23	Malate dehydrogenase, Phosphate acetyltransferase	Cytoplasmic	4,7,12,14
A1B0B9/Pden_0851	1	2	2	1	Nicotinamidase	Cytoplasmic	0
A1B0C0/Pden_0852	6	4	7	4	Nicotinate phosphoribosyltransferase	Cytoplasmic	8,1,4,7,12,14
A1B0C1/Pden_0853	4	4	3	5	NH-dependent NAD synthetase	Cytoplasmic	8,1,4,7,12,14
A1B0C2/Pden_0854	3	3	2	3	MORN repeat-containing protein	Periplasmic	0
A1B0C3/Pden_0855	5	6	6	6	Xanthine phosphoribosyltransferase	Cytoplasmic	8,1,4,7,12,14
A1B0C5/Pden_0857	15	14	12	14	Enoyl-[acyl-carrier-protein] reductase [NADH]	Membrane	8,1,4,7,12,14
A1B0C6/Pden_0858	2	4	1	1	Pyridoxine/pyridoxamine 5'-phosphate oxidase	Cytoplasmic	1,6,4,7,12,14
A1B0C7/Pden_0859	2	3	2	4	Cold-shock DNA-binding protein family	Cytoplasmic	9
A1B0C8/Pden_0860	2	2	1	2	Uncharacterized protein	Unknown	0
A1B0C9/Pden_0862	15	18	16	16	Aspartokinase	Cytoplasmic	8,1,4,7,12,14
A1B0D0/Pden_0863	4	5	4	4	PTSINtr with GAF domain (PtsP)	Cytoplasmic	5,13
A1B0E4/Pden_0877	7	7	9	8	Membrane protein insertase (YidC)	Membrane	5,23
A1B0E5/Pden_0878	1	2	1	2	MOSC domain containing protein	Cytoplasmic	0
A1B0E6/Pden_0879	2	2	3	4	Probable GTP-binding protein (EngB)	Unknown	3,12
A1B0E7/Pden_0880	8	4	4	6	Acetylglutamate kinase	Cytoplasmic	8,1,4,7,12,14
A1B0E8/Pden_0881	5	3	3	5	Putative phosphohistidine phosphatase (SixA)	Cytoplasmic	0
A1B0E9/Pden_0882	3	3	1	1	Phosphofructokinase	Cytoplasmic	8,7
A1B0F1/Pden_0884	22	19	23	22	Glutamate--tRNA ligase 1	Cytoplasmic	8,6,4,7,12,14
A1B0F4/Pden_0887	31	30	31	32	Trigger factor	Cytoplasmic	25,12,5,23
A1B0F6/Pden_0889	16	18	20	17	50S ribosomal protein L9	Cytoplasmic	6,1,8,4,7
A1B0F7/Pden_0890	4	6	6	5	30S ribosomal protein S18	Cytoplasmic	6,1,8,4,7

A1B0F8/Pden_0891	10	11	10	12	30S ribosomal protein S6	Cytoplasmic	6,1,8,4,7
A1B0F9/Pden_0892	5	4	6	7	YceI family protein	Unknown	0
A1B0G0/Pden_0893	13	13	11	11	Cytochrome-c peroxidase	Unknown	0
A1B0G1/Pden_0894	8	5	4	6	Peptide chain release factor 1	Cytoplasmic	0
A1B0G3/Pden_0896	6	4	5	4	Uncharacterized protein	Unknown	0
A1B0G5/Pden_0898	4	1	2	3	4-hydroxythreonine-4-phosphate dehydrogenase, L-threonine dehydrogenase	Cytoplasmic	1,6,4,7,12,14
A1B0G6/Pden_0899	15	9	16	13	Chaperone (SurA)	Unknown	0
A1B0G7/Pden_0900	2	1	0	1	LPS-assembly protein (LptD)	Unknown	3,12,5,13,32
A1B0H0/Pden_0903	29	27	27	25	Probable cytosol aminopeptidase	Cytoplasmic	0
A1B0H4/Pden_0907	5	6	6	6	Sec-independent protein translocase protein (TatA)	Membrane	12,5,13,23
A1B0H5/Pden_0908	1	2	3	1	Twin-arginine translocation protein (TatB)	Unknown	12,5,13,23
A1B0H7/Pden_0910	5	7	5	7	Uncharacterized protein	Cytoplasmic	0
A1B0H8/Pden_0911	3	1	1	2	Penicillin amidase, Cysteine peptidase, MEROPS family C59	Unknown	0
A1B0I0/Pden_0913	2	0	3	0	Homogentisate 1,2-dioxygenase	Cytoplasmic	4,8,7,12,14,2
A1B0I5/Pden_0918	1	3	2	1	Alpha/beta hydrolase fold protein	Cytoplasmic	0
A1B0I7/Pden_0920	22	26	29	26	Serine hydroxymethyltransferase	Cytoplasmic	8,1,4,7,12,14
A1B0I9/Pden_0922	7	5	9	6	Peptide chain release factor 3	Cytoplasmic	9
A1B0J4/Pden_0927	2	3	2	2	Deoxyguanosinetriphosphate triphosphohydrolase-like protein	Cytoplasmic	8,6,4,7,12,14
A1B0J6/Pden_0929	3	2	3	2	Exodeoxyribonuclease III	Cytoplasmic	4,8,7,12,14,13
A1B0J9/Pden_0932	7	6	6	7	3-hydroxybutyrate dehydrogenase	Cytoplasmic	0
A1B0K1/Pden_0934	14	13	12	13	Flavin-dependent thymidylate synthase	Cytoplasmic	8,1,4,7,12,14
A1B0K2/Pden_0935	1	1	2	2	Glyoxalase/bleomycin resistance protein/dioxygenase	Cytoplasmic	0
A1B0K5/Pden_0938	10	13	10	10	Aminotransferase	Cytoplasmic	1
A1B0K8/Pden_0941	2	3	2	2	Malate/L-lactate dehydrogenase	Cytoplasmic	0
A1B0L0/Pden_0943	1	4	2	3	Bifunctional protein (PutA)	Cytoplasmic	4,8,7,12,14,2
A1B0L2/Pden_0945	6	4	5	5	Substrate-binding region of ABC-type glycine betaine transport system	Periplasmic	0
A1B0L7/Pden_0950	1	3	1	3	Fervidolysin, Serine peptidase, MEROPS family S08A	Cytoplasmic	0
A1B0L8/Pden_0951	11	7	9	8	Ribonucleoside-diphosphate reductase, adenosylcobalamin-dependent	Cytoplasmic	6,1,8,4,7
A1B0L9/Pden_0952	1	2	2	2	DSBA oxidoreductase	Cytoplasmic	0
A1B0M1/Pden_0954	5	5	2	8	Cold-shock DNA-binding protein family	Cytoplasmic	1,6,8,4,7,9
A1B0M2/Pden_0955	18	20	16	19	Threonine--tRNA ligase	Cytoplasmic	8,6,4,7,12,14
A1B0M3/Pden_0956	3	4	2	6	Uncharacterized protein	Cytoplasmic	0
A1B0M4/Pden_0957	12	16	12	13	Polyhydroxyalkanoate depolymerase, intracellular	Cytoplasmic	0
A1B0M5/Pden_0958	2	4	4	3	Poly-hydroxyalkanoic acid synthase, class I	Cytoplasmic	1,7
A1B0M6/Pden_0959	20	20	19	23	Uncharacterized protein	Unknown	0
A1B0M7/Pden_0960	3	6	2	4	Polyhydroxyalkonate synthesis repressor (PhaR)	Cytoplasmic	0
A1B0P2/Pden_0975	3	3	3	2	Putative phage repressor	Cytoplasmic	0
A1B0P5/Pden_0978	0	0	0	2	Uncharacterized protein	Unknown	0
A1B0W8/Pden_1051	1	2	0	0	Aldehyde dehydrogenase	Cytoplasmic	0
A1B0X5/Pden_1060	2	1	2	2	Glyceraldehyde-3-phosphate dehydrogenase, type I	Cytoplasmic	0
A1B0Y2/Pden_1067	1	2	2	1	OsmC family protein	Cytoplasmic	11
A1B0Y5/Pden_1070	2	1	1	1	Regulatory protein (IclR)	Cytoplasmic	0
A1B106/Pden_1092	2	1	1	0	Acetolactate synthase, large subunit	Cytoplasmic	0
A1B121/Pden_1107	0	2	1	2	Alcohol dehydrogenase GroES domain protein	Cytoplasmic	0
A1B125/Pden_1111	0	1	0	2	NCS1 nucleoside transporter family	Membrane	12,5,13

A1B126/Pden_1112	4	3	4	4	Dihydropyrimidinase	Cytoplasmic	0
A1B127/Pden_1113	1	1	0	2	Amidase, hydantoinase/carbamoylase family	Cytoplasmic	0
A1B133/Pden_1119	3	6	4	4	Reduced coenzyme F420:NADP oxidoreductase	Unknown	8,6,4,7,12,14
A1B134/Pden_1120	6	4	6	7	Methylenetetrahydromethanopterin reductase	Cytoplasmic	0
A1B157/Pden_1143	2	3	1	4	ABC transporter related protein	Membrane	0
A1B194/Pden_1183	0	2	1	2	Transcriptional regulator, GntR family	Cytoplasmic	6,1,8,4,7
A1B1A8/Pden_1197	1	2	2	0	NADH:flavin oxidoreductase/NADH oxidase	Cytoplasmic	0
A1B1B4/Pden_1203	9	7	8	8	Uncharacterized protein	Unknown	0
A1B1B5/Pden_1204	3	1	1	2	D-alanine--D-alanine ligase	Cytoplasmic	12,7,14,9,10,19
A1B1B8/Pden_1207	2	2	1	2	Urease accessory protein (UreE)	Cytoplasmic	3,8,4,7,12,14
A1B1B9/Pden_1208	8	6	5	10	Urease subunit alpha	Cytoplasmic	4,6,7,12,14,2
A1B1C2/Pden_1211	2	2	2	2	Urease subunit gamma	Cytoplasmic	4,6,7,12,14,2
A1B1C7/Pden_1216	1	2	0	1	Activator of Hsp90 ATPase 1 family protein	Unknown	11
A1B1D0/Pden_1219	0	2	3	2	Uncharacterized protein	Membrane	0
A1B1E4/Pden_1233	5	6	5	5	2-dehydro-3-deoxyphosphooctonate aldolase	Cytoplasmic	8,1,4,7,12,14
A1B1E5/Pden_1234	3	2	4	6	Capsule polysaccharide export protein-like protein	Unknown	0
A1B1E8/Pden_1237	2	2	1	2	Uncharacterized protein	Unknown	0
A1B1F1/Pden_1240	4	2	4	4	Peptidoglycan-binding domain 1 protein	Unknown	0
A1B1F2/Pden_1241	10	6	5	7	UDP-3-O-acylglucosamine N-acyltransferase	Cytoplasmic	8,1,4,7,12,14
A1B1F4/Pden_1243	2	0	2	3	3-oxoacyl-[acyl-carrier-protein] synthase II	Membrane	0
A1B1F6/Pden_1245	6	7	7	7	2-keto-3-deoxy-phosphogluconate aldolase	Cytoplasmic	0
A1B1F7/Pden_1246	4	3	3	3	Uncharacterized protein	Unknown	0
A1B1F8/Pden_1247	7	4	5	5	Glutathionylspermidine synthase	Cytoplasmic	0
A1B1F9/Pden_1248	20	16	20	20	Extracellular solute-binding protein, family 5	Periplasmic	12,5,13
A1B1G2/Pden_1251	0	2	0	1	ABC transporter related protein	Membrane	0
A1B1G5/Pden_1254	2	0	0	1	Aldehyde dehydrogenase	Cytoplasmic	0
A1B1G6/Pden_1255	4	4	4	5	Short-chain dehydrogenase/reductase SDR	Cytoplasmic	0
A1B1H0/Pden_1259	4	5	5	5	Periplasmic solute binding protein	Periplasmic	18,5,13
A1B1H5/Pden_1264	2	2	3	3	Peptidyl-tRNA hydrolase	Cytoplasmic	6,1,8,4,7
A1B1H6/Pden_1265	5	5	5	6	ATP-dependent Clp protease proteolytic subunit	Cytoplasmic	0
A1B1H7/Pden_1266	13	15	12	11	ATP-dependent Clp protease ATP-binding subunit (ClpX)	Cytoplasmic	25
A1B1H8/Pden_1267	4	4	3	4	NADH:ubiquinone oxidoreductase 17.2 kD subunit	Unknown	0
A1B1I2/Pden_1271	15	16	13	15	Acetyl-CoA carboxylase carboxyltransferase subunit alpha	Cytoplasmic	0
A1B1I3/Pden_1272	3	4	3	3	Biotin carboxyl carrier protein	Unknown	8,1,4,7,12,14
A1B1I4/Pden_1273	2	0	0	0	Lipid A biosynthesis acyltransferase	Unknown	0
A1B1I6/Pden_1275	36	36	43	38	Transketolase	Cytoplasmic	0
A1B1J1/Pden_1280	4	1	4	3	Uncharacterized protein	Unknown	0
A1B1J4/Pden_1283	12	8	9	9	3-dehydroquinate synthase	Cytoplasmic	8,1,4,7,12,14
A1B1K1/Pden_1290	6	4	4	4	Orn/DAP/Arg decarboxylase 2	Cytoplasmic	0
A1B1K3/Pden_1292	0	2	1	0	AMP-dependent synthetase and ligase	Cytoplasmic	0
A1B1K5/Pden_1294	2	4	2	2	Uncharacterized protein	Cytoplasmic	0
A1B1L0/Pden_1299	24	21	21	20	Proline--tRNA ligase	Cytoplasmic	8,6,4,7,12,14
A1B1L3/Pden_1302	0	2	2	3	Polyphosphate kinase	Membrane	1,4,7,12,14
A1B1L4/Pden_1303	10	7	5	6	Ppx/GppA phosphatase	Cytoplasmic	0
A1B1L5/Pden_1304	7	6	5	6	Dipeptidase AC, Metallo peptidase, MEROPS family M19	Cytoplasmic	0

A1B1L7/Pden_1306	6	7	8	6	Cobyrinic acid a,c-diamide synthase	Cytoplasmic	0
A1B1L9/Pden_1308	5	7	7	6	UvrABC system protein B	Cytoplasmic	7,4,12,14,13,29
A1B1M0/Pden_1309	6	6	6	7	ETC complex I subunit conserved region	Unknown	4,12,14
A1B1M5/Pden_1314	17	13	14	14	Periplasmic glucan biosynthesis protein (MdoG)	Periplasmic	1,8,7,14
A1B1M6/Pden_1315	2	2	3	2	Glycosyl transferase, family 2	Membrane	0
A1B1M8/Pden_1317	16	17	15	18	Formate--tetrahydrofolate ligase	Cytoplasmic	8,1,4,7,12,14
A1B1N1/Pden_1320	4	4	5	5	Uncharacterized protein	Cytoplasmic	0
A1B1N2/Pden_1321	24	23	21	25	Lysine--tRNA ligase	Cytoplasmic	8,6,4,7,12,14
A1B1N4/Pden_1323	1	3	2	4	Pyridoxamine 5'-phosphate oxidase-related, FMN-binding protein	Unknown	0
A1B1N5/Pden_1324	11	10	6	12	Oligopeptide/dipeptide ABC transporter, ATPase subunit	Membrane	5,13
A1B1N9/Pden_1328	50	51	51	48	Extracellular solute-binding protein, family 5	Periplasmic	12,5,13
A1B1R3/Pden_1355	5	2	3	4	Methionine synthase	Cytoplasmic	8,1,4,7,12,14
A1B1R9/Pden_1362	4	3	3	1	Periplasmic binding protein	Unknown	0
A1B1T3/Pden_1376	6	6	3	6	Lipoyl synthase	Cytoplasmic	8,4,7
A1B1T4/Pden_1377	1	1	2	1	Redoxin domain protein	Unknown	0
A1B1T7/Pden_1380	11	8	7	9	Uncharacterized protein	Unknown	0
A1B1T8/Pden_1381	3	6	7	6	Invasion associated locus B family protein	Periplasmic	0
A1B1T9/Pden_1382	12	13	12	12	Protease Do	Periplasmic	0
A1B1U1/Pden_1384	17	16	19	22	Ribonucleoside-diphosphate reductase class II	Cytoplasmic	6,1,8,4,7
A1B1U2/Pden_1385	0	2	0	1	5-formyltetrahydrofolate cyclo-ligase	Unknown	0
A1B1U3/Pden_1386	2	1	1	1	Magnesium transporter (MgtE)	Membrane	5,13
A1B1U4/Pden_1387	2	3	6	5	Inositol monophosphatase	Cytoplasmic	8,4,7,12,14
A1B1U6/Pden_1389	2	4	3	2	Alcohol dehydrogenase, zinc-binding domain protein	Cytoplasmic	0
A1B1U7/Pden_1390	2	0	1	2	Uncharacterized protein	Membrane	0
A1B1U9/Pden_1392	3	5	6	7	Glutaredoxin	Unknown	12,9,10
A1B1V1/Pden_1394	0	1	2	1	Uncharacterized protein	Cytoplasmic	0
A1B1V2/Pden_1395	18	17	17	17	Aminotransferase, class V	Cytoplasmic	0
A1B1V3/Pden_1396	3	2	2	4	Glutamine amidotransferase class-I	Cytoplasmic	8,6,4,7,12,14
A1B1V4/Pden_1397	1	2	1	0	L-glutamine synthetase	Cytoplasmic	8,1,4,7,12,14
A1B1V5/Pden_1398	2	2	3	1	Gamma-glutamylputrescine oxidase	Cytoplasmic	0
A1B1V7/Pden_1400	8	6	6	5	ppGpp synthetase I, SpoT/RelA	Cytoplasmic	8,6,4,7,12,14
A1B1V8/Pden_1401	5	4	6	7	DNA-directed RNA polymerase subunit omega	Cytoplasmic	6,1,8,4,7
A1B1W2/Pden_1405	5	8	6	5	Uncharacterized protein	Membrane	0
A1B1W5/Pden_1408	2	1	1	0	Prephenate dehydrogenase	Membrane	8,1,4,7,12,14
Q51687/Pden_1409	8	8	11	7	Histidinol-phosphate aminotransferase	Cytoplasmic	8,1,4,7,12,14
A1B1X2/Pden_1415	4	4	4	4	Protein-export membrane protein (SecF)	Membrane	12,5,13,23
A1B1X3/Pden_1416	12	13	11	10	Protein translocase subunit (SecD)	Membrane	12,5,13,23
A1B1X4/Pden_1417	4	5	6	6	Protein translocase subunit (YajC)	Membrane	0
A1B1X7/Pden_1420	1	1	1	2	tRNA-dihydrouridine synthase	Cytoplasmic	0
A1B1Z2/Pden_1435	1	3	1	0	Glutamine amidotransferase class-I	Cytoplasmic	8,6,4,7,12,14
A1B282/Pden_1526	2	3	2	4	Helicase domain protein	Cytoplasmic	0
A1B283/Pden_1527	1	1	2	2	DEAD/DEAH box helicase domain protein	Membrane	0
A1B284/Pden_1528	4	4	3	5	Uncharacterized protein	Cytoplasmic	0
A1B2A1/Pden_1545	1	5	2	3	Histone family protein nucleoid-structuring protein H-NS	Unknown	9
A1B2A7/Pden_1551	15	10	11	12	Uncharacterized protein	OuterMembrane	0

A1B2A9/Pden_1553	3	4	4	5	N utilization substance protein B homolog	Unknown	1,6,8,4,7,9
A1B2B0/Pden_1554	10	6	5	7	6,7-dimethyl-8-ribityllumazine synthase	Cytoplasmic	1,6,4,7,12,14
A1B2B1/Pden_1555	24	22	22	25	3,4-dihydroxy-2-butanone 4-phosphate synthase	Cytoplasmic	1,6,4,7,12,14
A1B2B3/Pden_1557	0	3	3	2	Riboflavin synthase, alpha subunit	Cytoplasmic	1,6,4,7,12,14
A1B2B5/Pden_1559	19	21	22	18	Polysaccharide export protein	Unknown	0
A1B2B7/Pden_1561	2	1	1	0	Diaminohydroxyphosphoribosylaminopyrimidine deaminase / 5-amino-6-uracil reductase	Cytoplasmic	1,6,4,7,12,14
A1B2B8/Pden_1562	4	4	3	4	Transcriptional repressor (NrdR)	Cytoplasmic	1,6,8,4,7,9
A1B2B9/Pden_1563	10	10	13	10	TRAP dicarboxylate transporter-DctP subunit	Periplasmic	5
A1B2C3/Pden_1567	12	10	10	13	Nicotinate-nucleotide--dimethylbenzimidazole phosphoribosyltransferase, α-phosphoribosyltransferase)	Cytoplasmic	8,1,4,7,12,14
A1B2C5/Pden_1569	50	51	50	48	Extracellular solute-binding protein, family 5	Periplasmic	12,5,13
A1B2E4/Pden_1588	5	7	7	6	UvrABC system protein B	Cytoplasmic	7,4,12,14,13,29
A1B2F0/Pden_1594	0	2	2	3	Polyphosphate kinase	Membrane	0
A1B2K2/Pden_1649	3	5	5	4	3-hydroxydecanoyl-[acyl-carrier-protein] dehydratase	Cytoplasmic	8,1,4,7,12,14
A1B2K3/Pden_1650	14	15	12	17	3-oxoacyl-[acyl-carrier-protein] synthase I	Cytoplasmic	0
A1B2K4/Pden_1651	8	8	10	9	Enoyl-[acyl-carrier-protein] reductase [NADH]	Membrane	8,1,4,7,12,14
A1B2K5/Pden_1652	9	8	6	8	Hydrolase or acyltransferase	Cytoplasmic	0
A1B2K6/Pden_1653	12	11	13	7	Lytic murein transglycosylase	Membrane	0
A1B2K8/Pden_1655	5	5	4	6	Elongation factor P	Cytoplasmic	0
A1B2K9/Pden_1656	11	10	10	9	N-acetyl-gamma-glutamyl-phosphate reductase	Cytoplasmic	8,1,4,7,12,14
A1B2L0/Pden_1657	5	4	2	3	3-methyl-2-oxobutanoate hydroxymethyltransferase	Cytoplasmic	1,6,4,7,12,14
A1B2M0/Pden_1667	4	1	4	5	ABC transporter related protein	Membrane	5,13
A1B2M1/Pden_1668	4	2	1	4	ABC transporter related protein	Membrane	5,13
A1B2M4/Pden_1671	31	31	29	28	Extracellular solute-binding protein, family 5	Periplasmic	12,5,13
A1B2M5/Pden_1672	1	2	2	1	5-deoxyglucuronate isomerase	Cytoplasmic	4,8,7,12,14,2
A1B2M7/Pden_1674	3	1	3	2	2-keto-myo-inositol dehydratase	Cytoplasmic	0
A1B2M8/Pden_1675	1	2	5	2	3D--trihydroxycyclohexane-1,2-dione hydrolase	Cytoplasmic	4,8,7,12,14,2
A1B2M9/Pden_1676	4	4	5	3	5-dehydro-2-deoxygluconokinase	Cytoplasmic	0
A1B2N0/Pden_1677	5	4	4	7	Xylose isomerase domain protein TIM barrel	Cytoplasmic	0
A1B2N1/Pden_1678	6	5	5	3	Inositol 2-dehydrogenase	Unknown	4,8,7,12,14,2
A1B2N2/Pden_1679	2	2	1	3	Xylose isomerase domain protein TIM barrel	Cytoplasmic	0
A1B2N3/Pden_1680	1	2	1	1	Oxidoreductase domain protein	Unknown	0
A1B2N4/Pden_1681	4	4	3	3	Monosaccharide ABC transporter substrate-binding protein, CUT2 family	Periplasmic	0
A1B2N6/Pden_1683	6	6	4	6	Putative ribose/galactose/methyl galactoside import ATP-binding protein	Membrane	0
A1B2N7/Pden_1684	4	4	7	4	Monosaccharide ABC transporter substrate-binding protein, CUT2 family	Periplasmic	0
A1B2S0/Pden_1717	3	1	0	2	Amino acid/amide ABC transporter substrate-binding protein, HAAT family	Unknown	0
A1B2S2/Pden_1719	6	4	4	4	AMP-dependent synthetase and ligase	Cytoplasmic	0
A1B2S3/Pden_1720	0	0	2	0	Malonyl-CoA decarboxylase	Cytoplasmic	8,1,4,7,12,14
A1B2T2/Pden_1729	6	2	3	3	Alanine dehydrogenase	Cytoplasmic	4,8,7,12,14,2
A1B2T7/Pden_1734	2	0	3	2	Uncharacterized protein	Periplasmic	0
A1B2T8/Pden_1735	3	4	4	4	Dyp-type peroxidase family	Periplasmic	12,5,13,10
A1B2T9/Pden_1736	6	10	7	7	Uncharacterized protein	Periplasmic	0
A1B2U3/Pden_1740	0	2	0	0	Uncharacterized protein	Cytoplasmic	0
A1B2U5/Pden_1742	0	0	0	2	D-aminoacyl-tRNA deacylase deacylase	Unknown	4,8,7,12,14,2
A1B2U6/Pden_1743	18	13	14	18	DEAD/DEAH box helicase domain protein	Cytoplasmic	0

A1B2U9/Pden_1746	9	14	13	13	Nucleoside diphosphate kinase	Extracellular	8,1,4,7,12,14
A1B2V0/Pden_1747	0	0	2	0	ABC transporter related protein	Cytoplasmic	0
A1B2V1/Pden_1748	5	8	7	8	MltA-interacting MipA family protein	Unknown	0
A1B2V5/Pden_1752	3	4	1	3	Coproporphyrinogen oxidase	Cytoplasmic	6,1,4,7
A1B2V6/Pden_1753	1	2	4	2	Uncharacterized protein	Unknown	0
A1B2V8/Pden_1755	1	2	1	0	16S rRNA mG 1207 methyltransferase	Cytoplasmic	0
A1B2W0/Pden_1757	4	8	4	2	Serine-type D-Ala-D-Ala carboxypeptidase	Membrane	0
A1B2W3/Pden_1760	23	26	23	21	Ketol-acid reductoisomerase	Cytoplasmic	8,1,4,7,12,14
A1B2W5/Pden_1762	2	2	3	2	DNA-directed DNA polymerase	Cytoplasmic	6,1,8,4,7
A1B2W6/Pden_1763	7	5	5	6	Nucleoside ABC transporter ATP-binding protein	Membrane	0
A1B2W9/Pden_1766	19	19	22	16	Nucleoside-binding protein	Unknown	0
A1B2X0/Pden_1767	2	3	4	4	Ferritin, Dps family protein	Cytoplasmic	11,12,10
A1B2X1/Pden_1768	2	2	3	4	Acyl carrier protein	Cytoplasmic	0
A1B2X2/Pden_1769	9	8	10	10	3-oxoacyl-[acyl-carrier-protein] reductase	Unknown	8,1,4,7,12,14
A1B2X3/Pden_1770	10	9	8	11	Malonyl CoA-acyl carrier protein transacylase	Cytoplasmic	0
A1B2X5/Pden_1772	2	2	2	2	Beta-lactamase domain protein	Unknown	0
A1B2Y3/Pden_1780	14	14	13	13	Efflux transporter, RND family, MFP subunit	Membrane	12,5,13
A1B2Z3/Pden_1790	1	3	2	4	Translation initiation factor IF-1	Cytoplasmic	0
A1B2Z5/Pden_1792	2	3	1	1	Ribonuclease	Cytoplasmic	0
A1B2Z9/Pden_1796	2	2	1	2	GCN5-related N-acetyltransferase	Cytoplasmic	0
A1B300/Pden_1797	6	4	4	3	Sulfate-transporting ATPase	Membrane	0
A1B301/Pden_1798	19	16	16	14	Sulfate ABC transporter, periplasmic sulfate-binding protein	Periplasmic	0
A1B307/Pden_1804	3	5	4	2	ABC transporter related protein	Membrane	5,13
A1B310/Pden_1807	5	9	7	7	Extracellular solute-binding protein, family 5	Unknown	12,5,13
A1B311/Pden_1808	7	7	7	8	Cytochrome *c*, class I	Membrane	0
A1B312/Pden_1809	5	6	4	8	Prephenate dehydratase	Cytoplasmic	8,1,4,7,12,14
A1B313/Pden_1810	1	0	2	1	Uncharacterized protein	Unknown	0
A1B316/Pden_1813	1	2	1	1	Pseudouridine-5'-phosphate glycosidase	Unknown	4,8,7,12,14,2
A1B318/Pden_1815	10	8	8	8	Peptide chain release factor 2	Cytoplasmic	0
A1B319/Pden_1816	3	8	8	9	Penicillin-binding protein, 1A family	Membrane	0
A1B321/Pden_1818	3	1	1	1	Aminotransferase	Cytoplasmic	1
A1B322/Pden_1819	7	9	10	9	DSBA oxidoreductase	Unknown	0
A1B323/Pden_1820	4	6	0	4	4-hydroxy-3-methylbut-2-en-1-yl diphosphate synthase, butenyl 4-diphosphate synthase	Cytoplasmic	8,1,4,7,12,14
P43089/Pden_1822	2	4	2	1	5-aminolevulinate synthase	Cytoplasmic	6,1,4,7
A1B326/Pden_1823	16	14	14	15	Peptidase M20	Cytoplasmic	0
A1B328/Pden_1825	10	10	10	7	Transcriptional regulator, CarD family	Cytoplasmic	0
A1B331/Pden_1828	19	16	20	16	Helicase domain protein	Cytoplasmic	0
A1B333/Pden_1830	4	5	7	5	Sterol-binding domain protein	Unknown	0
A1B335/Pden_1832	14	16	14	14	Oligoendopeptidase, pepF/M3 family	Cytoplasmic	0
A1B336/Pden_1833	1	1	2	1	Peptidase M19, renal dipeptidase	Unknown	0
A1B337/Pden_1834	2	3	2	2	Glutathione S-transferase, C-terminal domain	Cytoplasmic	0
A1B338/Pden_1835	2	1	1	0	Uncharacterized protein	Cytoplasmic	0
A1B339/Pden_1836	10	11	9	11	DNA topoisomerase 4 subunit B	Cytoplasmic	8,6,3,4,7,12
A1B345/Pden_1842	4	4	3	5	Heavy metal translocating P-type ATPase	Membrane	5,13
A1B346/Pden_1843	0	1	2	1	FixH family protein	Membrane	0
A1B347/Pden_1844	3	1	2	2	4Fe-4S ferredoxin, iron-sulfur binding domain protein	Membrane	0

A1B348/Pden_1845	11	12	10	10	*cbb*$_3$-type cytochrome *c* oxidase subunit (CcoP)	Cytoplasmic	7,4,12,14,5,13
A1B350/Pden_1847	9	10	10	8	Cytochrome c oxidase, *cbb*$_3$-type, subunit II	Cytoplasmic	4,12,14
A1B351/Pden_1848	1	1	2	1	Cytochrome c oxidase, *cbb*$_3$-type, subunit I	Membrane	4,12,14
A1B352/Pden_1849	20	18	19	16	UspA domain protein	Cytoplasmic	11
A1B353/Pden_1850	2	3	4	4	Putative transcriptional regulator, Crp/Fnr family	Membrane	6,1,8,4,7
Q51676/Pden_1851	12	11	11	12	Oxygen-independent coproporphyrinogen III oxidase	Cytoplasmic	6,1,4,7
A1B356/Pden_1853	15	14	13	12	DNA polymerase I	Cytoplasmic	6,1,8,4,7
A1B357/Pden_1854	2	3	2	3	Uncharacterized protein	Unknown	0
A1B358/Pden_1855	4	0	7	3	ABC transporter related protein	Membrane	0
A1B359/Pden_1856	1	2	2	4	Histidine triad protein	Cytoplasmic	0
A1B360/Pden_1857	1	2	1	1	Glycosyl transferase, family 14	Cytoplasmic	0
A1B362/Pden_1859	3	4	3	4	50S ribosomal protein L32	Cytoplasmic	6,1,8,4,7
A1B363/Pden_1860	5	6	6	6	Phosphate acyltransferase	Cytoplasmic	8,1,4,7,12,14
A1B364/Pden_1861	7	9	7	7	3-oxoacyl-[acyl-carrier-protein] synthase 3	Cytoplasmic	8,1,4,7,12,14
A1B366/Pden_1863	3	6	5	5	Integration host factor subunit alpha	Cytoplasmic	1,6,8,4,7,9
A1B368/Pden_1865	7	5	7	6	2-deoxycytidine 5-triphosphate deaminase	Cytoplasmic	8,6,4,7,12,14
A1B369/Pden_1866	0	1	1	2	Segregation and condensation protein B	Cytoplasmic	12
A1B371/Pden_1868	4	3	3	4	Beta-N-acetylhexosaminidase	Cytoplasmic	8,7
A1B372/Pden_1869	3	4	3	3	Sporulation domain protein	Unknown	0
A1B373/Pden_1870	18	19	23	23	Arginine--tRNA ligase	Cytoplasmic	8,6,4,7,12,14
A1B374/Pden_1873	17	13	18	13	S-adenosylmethionine synthase	Cytoplasmic	8,1,4,7,12,14
A1B375/Pden_1874	1	1	2	2	Heat shock protein DnaJ domain protein	Cytoplasmic	0
A1B377/Pden_1876	16	17	16	14	Fructose-1,6-bisphosphatase	Cytoplasmic	8,1,4,7,12,14
A1B378/Pden_1877	5	4	6	4	Exonuclease (RecJ)	Cytoplasmic	4,8,7,12,14,13
A1B380/Pden_1879	34	35	31	36	Carbamoyl-phosphate synthase large chain	Unknown	8,1,4,7,12,14
A1B382/Pden_1881	2	2	5	4	Cell division protein (ZapA)	Unknown	3,12
A1B383/Pden_1882	4	8	5	7	Uncharacterized protein	Cytoplasmic	0
A1B384/Pden_1883	3	3	4	5	Imidazoleglycerol-phosphate dehydratase	Cytoplasmic	8,1,4,7,12,14
A1B385/Pden_1884	0	3	3	1	Imidazole glycerol phosphate synthase subunit (HisH)	Cytoplasmic	8,1,4,7,12,14
A1B387/Pden_1886	6	8	8	7	1--5-[methylideneamino] imidazole-4-carboxamide isomerase	Cytoplasmic	8,1,4,7,12,14
A1B388/Pden_1887	1	2	1	1	Imidazole glycerol phosphate synthase subunit (HisF)	Cytoplasmic	8,1,4,7,12,14
A1B391/Pden_1890	8	7	8	8	Ribonuclease D	Cytoplasmic	6,8,4,7
A1B392/Pden_1891	2	2	2	2	Queuine tRNA-ribosyltransferase	Cytoplasmic	8,1,4,7,12,14
A1B395/Pden_1894	2	2	2	2	Uncharacterized protein	Unknown	0
A1B396/Pden_1895	1	3	1	4	Uncharacterized protein	Cytoplasmic	0
A1B3A0/Pden_1899	2	3	3	2	Substrate-binding region of ABC-type glycine betaine transport system	Unknown	5,13
A1B3A2/Pden_1901	1	2	0	0	ABC transporter related protein	Membrane	0
A1B3A6/Pden_1905	7	4	5	5	Probable transcriptional regulatory protein	Cytoplasmic	1,6,8,4,7,9
A1B3A9/Pden_1908	21	22	19	22	Fumarate hydratase class II	Cytoplasmic	8,4,7,12,14
A1B3B0/Pden_1909	1	1	3	1	Uncharacterized protein	Cytoplasmic	0
A1B3B3/Pden_1913	6	5	4	5	Tyrosine--tRNA ligase	Cytoplasmic	8,6,4,7,12,14
A1B3B6/Pden_1916	5	6	5	8	Peptidyl-prolyl cis-trans isomerase	Unknown	25
A1B3B7/Pden_1917	6	9	9	10	Peptidyl-prolyl cis-trans isomerase	Periplasmic	25
A1B3B8/Pden_1918	21	22	22	21	Phosphoglycerate kinase	Cytoplasmic	4,8,7,12,14,2
A1B3C0/Pden_1920	26	23	26	26	Fructose-bisphosphate aldolase	Cytoplasmic	0
A1B3C1/Pden_1921	6	6	6	7	Signal recognition particle receptor (FtsY)	Cytoplasmic	12,3,5,13,23,22

A1B3C3/Pden_1923	1	2	2	1	ATP phosphoribosyltransferase	Cytoplasmic	8,1,4,7,12,14
A1B3C4/Pden_1924	3	0	1	1	ATP phosphoribosyltransferase regulatory subunit	Cytoplasmic	0
A1B3C5/Pden_1925	19	18	15	19	Histidine--tRNA ligase	Cytoplasmic	8,6,4,7,12,14
A1B3C7/Pden_1927	4	7	6	6	Uncharacterized protein	Unknown	0
A1B3C9/Pden_1929	20	19	19	17	Aspartyl/glutamyl-tRNA amidotransferase subunit B	Cytoplasmic	6,1,8,4,7
A1B3D0/Pden_1931	11	10	10	11	Uncharacterized protein	Unknown	0
A1B3D2/Pden_1933	16	18	19	19	O-acetylhomoserine sulfhydrolase	Cytoplasmic	0
A1B3D4/Pden_1935	18	17	17	11	Methionine synthase	Cytoplasmic	6,4,7
A1B3D5/Pden_1936	1	1	0	2	Octanoyltransferase	Cytoplasmic	8,1,4,7,12,14
A1B3D6/Pden_1937	3	1	3	3	Cytochrome c, class I	Periplasmic	0
A1B3D7/Pden_1938	4	3	4	3	Cytochrome c oxidase subunit 1	Membrane	4,12,14
A1B3E0/Pden_1941	10	10	15	12	Glyoxylate reductase	Cytoplasmic	0
A1B3E3/Pden_1944	9	3	7	7	Peptidyl-dipeptidase (Dcp), Metallo peptidase, MEROPS family M03A	Cytoplasmic	0
A1B3E5/Pden_1946	1	0	1	2	Deoxyuridine 5'-triphosphate nucleotidohydrolase	Cytoplasmic	8,6,4,7,12,14
A1B3E7/Pden_1949	2	2	3	1	Putative L-sorbosone dehydrogenase	Membrane	8,7
A1B3E8/Pden_1950	2	2	0	0	Glucose-6-phosphate isomerase	Cytoplasmic	4,8,7,12,14,2
A1B3E9/Pden_1951	0	2	1	2	6-phosphogluconolactonase	Cytoplasmic	8,6,4,7,12,14
A1B3F0/Pden_1952	4	6	4	6	Glucose-6-phosphate 1-dehydrogenase	Cytoplasmic	8,6,4,7,12,14
A1B3F3/Pden_1955	4	3	4	4	6-phosphogluconate dehydratase	Cytoplasmic	4,8,7,12,14,2
A1B3F7/Pden_1959	11	10	10	12	Histidinol dehydrogenase	Cytoplasmic	8,1,4,7,12,14
A1B3F8/Pden_1960	1	1	2	2	Uncharacterized protein	Cytoplasmic	0
A1B3F9/Pden_1961	11	13	11	11	UDP-N-acetylglucosamine 1-carboxyvinyltransferase	Cytoplasmic	12,7,14,9,10,19
A1B3G7/Pden_1969	2	1	0	1	Uncharacterized protein	Cytoplasmic	0
A1B3H6/Pden_1978	2	2	1	2	Periplasmic binding protein/LacI transcriptional regulator	Cytoplasmic	6,1,8,4,7
A1B3H7/Pden_1979	10	8	10	9	Methionine aminopeptidase	Cytoplasmic	8,4,7
A1B3H8/Pden_1980	3	3	3	3	Molybdopterin binding domain	Cytoplasmic	0
A1B3H9/Pden_1981	1	1	2	0	GCN5-related N-acetyltransferase	Periplasmic	0
A1B3I2/Pden_1984	10	11	6	13	Dihydroxy-acid dehydratase	Cytoplasmic	8,1,4,7,12,14
A1B3I5/Pden_1987	13	6	8	8	Acetyl-coenzyme A carboxylase carboxyl transferase subunit beta	Cytoplasmic	8,1,4,7,12,14
A1B3I6/Pden_1988	3	2	1	2	FolC bifunctional protein	Cytoplasmic	0
A1B3I7/Pden_1989	6	9	5	4	AFG1-family ATPase	Cytoplasmic	0
A1B3I8/Pden_1990	5	3	7	7	Uncharacterized protein	Unknown	0
A1B3J3/Pden_1995	18	17	16	16	HemY domain protein	Unknown	0
A1B3J4/Pden_1996	20	24	24	23	Uncharacterized protein	Unknown	0
A1B3J5/Pden_1997	1	0	2	1	Putative uroporphyrinogen-III synthase	Unknown	6,1,4,7
A1B3J6/Pden_1998	0	0	2	2	tRNA N6-adenosine threonylcarbamoyltransferase A synthase) A37 threonylcarbamoyladenosine biosynthesis protein (TsaD)	Extracellular	6,8,4,7
A1B3J7/Pden_1999	4	3	3	1	Glycerol-3-phosphate dehydrogenase [NAD+], H-dependent glycerol-3-phosphate dehydrogenase	Unknown	4,8,7,12,14,2
A1B3J9/Pden_2001	1	4	3	6	Uncharacterized protein	Cytoplasmic	0
A1B3K0/Pden_2002	6	8	9	9	Lipid A ABC exporter family, fused ATPase and inner membrane subunits	Membrane	0
A1B3K2/Pden_2004	5	7	5	6	Peptidoglycan-binding (LysM)	Unknown	0
A1B3K3/Pden_2005	3	2	2	2	AMP-dependent synthetase and ligase	Cytoplasmic	0
A1B3K6/Pden_2008	24	26	22	22	ABC transporter related protein	Cytoplasmic	0
A1B3K8/Pden_2010	1	1	2	1	Ribosomal protein S12 methylthiotransferase (RimO), methylthiotransferase	Cytoplasmic	6,8,4,7

A1B3L2/Pden_2014	2	0	2	1	ABC transporter related protein	Membrane	0
A1B3L4/Pden_2016	4	3	5	2	Uncharacterized protein	Membrane	0
A1B3L8/Pden_2020	5	6	3	6	Diaminopimelate decarboxylase	Cytoplasmic	8,1,4,7,12,14
A1B3M0/Pden_2022	11	10	9	10	Argininosuccinate lyase	Cytoplasmic	8,1,4,7,12,14
A1B3M4/Pden_2026	28	27	28	28	Acetyl-CoA acetyltransferase	Cytoplasmic	0
A1B3M5/Pden_2027	13	15	17	17	3-oxoacyl-[acyl-carrier-protein] reductase	Cytoplasmic	1,7
A1B3M9/Pden_2031	7	7	11	7	Trans-hexaprenyltranstransferase	Cytoplasmic	8,1,4,7,12,14
A1B3N0/Pden_2032	2	2	2	2	Ammonium transporter	Membrane	0
A1B3N1/Pden_2033	10	7	8	8	Penicillin-binding protein, 1A family	Membrane	0
A1B3N2/Pden_2034	1	1	0	2	Toluene tolerance family protein	Unknown	0
A1B3N7/Pden_2039	8	5	5	6	Ornithine carbamoyltransferase	Cytoplasmic	8,1,4,7,12,14
A1B3N9/Pden_2041	12	13	9	11	Acetylornithine aminotransferase	Cytoplasmic	8,1,4,7,12,14
A1B3P1/Pden_2043	8	4	6	5	GcrA cell cycle regulator	Unknown	0
A1B3P3/Pden_2045	12	16	12	14	Methionine--tRNA ligase	Cytoplasmic	8,6,4,7,12,14
A1B3P4/Pden_2046	24	20	20	19	TonB-dependent receptor	OuterMembrane	5
A1B3P5/Pden_2047	3	2	3	5	Arginase	Unknown	8,6,4,7,12,14
A1B3Q9/Pden_2061	2	2	1	1	DNA-cytosine methyltransferase	Cytoplasmic	0
A1B3S3/Pden_2075	1	2	0	0	Ribonuclease (VapC)	Unknown	0
A1B3T6/Pden_2088	11	13	10	13	Endonuclease	Unknown	0
A1B3T8/Pden_2090	4	3	4	7	Uncharacterized protein	Cytoplasmic	0
A1B3U2/Pden_2094	3	2	3	2	Tetraacyldisaccharide 4'-kinase	Cytoplasmic	8,1,4,7,12,14
A1B3U3/Pden_2095	9	9	6	11	DSBA oxidoreductase	Unknown	0
A1B3V1/Pden_2103	6	4	5	6	Ureidoglycolate lyase	Cytoplasmic	4,8,7,12,14,2
A1B3V3/Pden_2105	3	3	5	4	Transcriptional regulator, GntR family	Cytoplasmic	6,1,8,4,7
A1B3V4/Pden_2106	2	3	3	3	ErfK/YbiS/YcfS/YnhG family protein	Unknown	0
A1B3W7/Pden_2119	5	6	5	2	Glucose-1-phosphate thymidylyltransferase	Cytoplasmic	8,1,4,7,12,14
A1B3W9/Pden_2121	5	5	7	5	dTDP-glucose 4,6-dehydratase	Cytoplasmic	6,8,4,7
A1B3X4/Pden_2126	9	9	6	9	Capsule polysaccharide export protein-like protein	Membrane	0
A1B3X6/Pden_2128	3	0	4	3	Uncharacterized protein	Unknown	0
A1B3Y0/Pden_2132	6	10	6	6	Anthranilate synthase, component I	Cytoplasmic	8,1,4,7,12,14
A1B3Y1/Pden_2133	14	15	14	14	Putative peptidyl-prolyl cis-trans isomerase	Membrane	0
A1B3Y4/Pden_2136	3	3	4	2	Anthranilate synthase, component II	Cytoplasmic	0
A1B3Y5/Pden_2137	2	3	5	4	Anthranilate phosphoribosyltransferase	Membrane	8,1,4,7,12,14
A1B3Y7/Pden_2139	5	7	5	6	Indole-3-glycerol phosphate synthase	Cytoplasmic	8,1,4,7,12,14
A1B3Y8/Pden_2140	1	2	3	1	Cyclic pyranopterin monophosphate synthase accessory protein	Cytoplasmic	6,1,8,4,7
A1B3Y9/Pden_2141	3	3	7	5	Molybdopterin molybdochelatase	Cytoplasmic	6,1,4,7
A1B3Z0/Pden_2142	6	5	6	6	LexA repressor	Cytoplasmic	12,7,14,9,13,29
A1B3Z1/Pden_2143	9	6	11	8	Serine-type D-Ala-D-Ala carboxypeptidase	Membrane	0
A1B3Z2/Pden_2144	0	1	2	1	Thymidylate kinase	Cytoplasmic	8,1,4,7,12,14
A1B3Z3/Pden_2145	0	2	2	2	DNA polymerase III, delta prime subunit	Cytoplasmic	0
A1B3Z4/Pden_2146	5	5	5	4	Hydrolase, TatD family	Cytoplasmic	0
A1B3Z9/Pden_2151	7	8	9	9	Polyamine ABC trasnporter, periplasmic polyamine-binding protein	Periplasmic	0
A1B400/Pden_2152	11	8	5	9	Extracellular solute-binding protein, family 1	Periplasmic	0
A1B401/Pden_2153	5	8	10	5	Polyamine-transporting ATPase	Membrane	0
A1B412/Pden_2164	2	2	2	2	GatB/Yqey domain protein	Cytoplasmic	0
A1B413/Pden_2165	7	8	9	9	Carbamoyl-phosphate synthase small chain	Cytoplasmic	8,1,4,7,12,14

A1B415/Pden_2167	2	3	1	1	Glutamate-ammonia-ligase adenylyltransferase	Cytoplasmic	0
A1B417/Pden_2169	19	13	20	19	Acetolactate synthase	Cytoplasmic	8,1,4,7,12,14
A1B418/Pden_2170	8	8	8	9	Acetolactate synthase, small subunit	Cytoplasmic	8,1,4,7,12,14
A1B420/Pden_2172	1	1	2	2	Peptidase M48, Ste24p	Unknown	0
A1B423/Pden_2175	2	2	4	4	Nitroreductase	Cytoplasmic	0
A1B426/Pden_2178	4	4	6	5	Methylmalonyl-CoA epimerase	Cytoplasmic	0
A1B428/Pden_2180	1	1	2	2	GCN5-related N-acetyltransferase	Unknown	0
A1B431/Pden_2183	26	28	18	30	Aspartate--tRNA ligase	Cytoplasmic	8,6,4,7,12,14
A1B433/Pden_2185	2	5	3	3	Glycoside hydrolase, family 25	Unknown	6,8,4,7,2
A1B435/Pden_2187	7	8	8	7	D-lactate dehydrogenase	Cytoplasmic	0
A1B437/Pden_2189	20	21	23	22	Cobaltochelatase (CobS)	Cytoplasmic	0
A1B439/Pden_2191	9	10	6	5	Cobaltochelatase (CobT)	Cytoplasmic	1,6,4,7,12,14
A1B440/Pden_2192	0	2	0	1	Glycosyl transferase, group 1	Cytoplasmic	0
A1B441/Pden_2193	8	10	6	8	Peptidase M24	Cytoplasmic	0
A1B443/Pden_2195	3	2	0	6	Hydroxypyruvate reductase	Cytoplasmic	0
A1B444/Pden_2196	7	5	6	7	D-isomer specific 2-hydroxyacid dehydrogenase, NAD-binding protein	Cytoplasmic	0
A1B445/Pden_2197	1	0	2	1	Export-related chaperone CsaA	Cytoplasmic	0
A1B446/Pden_2198	6	7	7	7	Pyrroline-5-carboxylate reductase	Cytoplasmic	8,1,4,7,12,14
A1B449/Pden_2201	10	10	11	8	N5-carboxyaminoimidazole ribonucleotide synthase imidazole ribonucleotide synthetase)	Membrane	8,1,4,7,12,14
A1B450/Pden_2202	2	3	2	4	N5-carboxyaminoimidazole ribonucleotide mutase imidazole ribonucleotide mutase)	Unknown	8,1,4,7,12,14
A1B452/Pden_2204	4	5	6	7	Uncharacterized protein	Unknown	0
A1B456/Pden_2208	11	10	14	14	Thioredoxin reductase	Cytoplasmic	4,12,14,21
A1B457/Pden_2209	0	3	4	3	Transcriptional regulator, AsnC family	Cytoplasmic	6,1,8,4,7
A1B459/Pden_2211	2	2	1	4	Laccase domain protein	Unknown	0
A1B464/Pden_2216	3	5	5	3	RNA polymerase sigma factor RpoH	Cytoplasmic	7,4,12,31,27,16
A1B465/Pden_2217	0	0	2	2	Uncharacterized protein	Cytoplasmic	0
A1B467/Pden_2219	8	6	6	7	Uracil phosphoribosyltransferase	Cytoplasmic	8,1,4,7,12,14
A1B468/Pden_2220	1	1	2	1	Adenosine deaminase	Cytoplasmic	0
A1B469/Pden_2221	1	3	0	1	Phosphopentomutase	Cytoplasmic	4,8,7,12,14,2
A1B470/Pden_2222	2	1	1	2	Thymidine phosphorylase	Unknown	8,6,4,7,12,14
A1B472/Pden_2224	20	26	24	23	Malate dehydrogenase)., Phosphate acetyltransferase	Cytoplasmic	4,7,12,14
A1B473/Pden_2225	3	3	3	1	Propionyl-CoA synthetase	Cytoplasmic	0
A1B474/Pden_2226	18	16	15	15	3-phosphoshikimate 1-carboxyvinyltransferase	Cytoplasmic	8,1,4,7,12,14
A1B475/Pden_2227	3	3	3	4	tRNA -)-methyltransferase -N)-methyltransferase) -methyltransferase)	Cytoplasmic	0
A1B476/Pden_2228	16	15	15	12	Beta-lactamase domain protein	Cytoplasmic	0
A1B478/Pden_2230	2	5	4	6	Biotin--acetyl-CoA-carboxylase ligase	Cytoplasmic	8,4,7
A1B479/Pden_2231	2	1	2	1	NADH-quinone oxidoreductase subunit N	Membrane	4,12,14
A1B481/Pden_2233	1	1	3	1	NADH dehydrogenase subunit L	Membrane	4,12,14
A1B486/Pden_2238	9	11	8	10	NADH-quinone oxidoreductase subunit I	Cytoplasmic	0
A1B488/Pden_2240	2	3	1	4	Uncharacterized protein	Unknown	0
A1B489/Pden_2241	33	29	27	34	NADH-quinone oxidoreductase	Cytoplasmic	4,12,14
A1B490/Pden_2242	0	2	2	2	Uncharacterized protein	Unknown	0
A1B491/Pden_2243	23	22	22	20	NADH dehydrogenase subunit F	Cytoplasmic	0
A1B493/Pden_2245	3	2	2	2	Uncharacterized protein	Membrane	0
A1B494/Pden_2246	10	9	11	9	NADH dehydrogenase subunit E	Unknown	0

A1B495/Pden_2247	18	13	15	14	NADH-quinone oxidoreductase subunit D	Cytoplasmic	5
A1B496/Pden_2248	12	14	13	14	NADH-quinone oxidoreductase subunit C	Cytoplasmic	5
A1B497/Pden_2249	3	3	2	5	NADH-quinone oxidoreductase subunit B	Membrane	5
A1B4A0/Pden_2252	7	5	10	7	UvrABC system protein A	Cytoplasmic	7,4,12,14,13,29
A1B4A3/Pden_2255	2	2	2	3	Uncharacterized protein	Periplasmic	0
A1B4A5/Pden_2257	22	21	20	23	Succinate semialdehyde dehydrogenase	Cytoplasmic	4,8,7,12,14,2
A1B4A6/Pden_2258	17	16	11	18	Uncharacterized protein	Unknown	0
A1B4B0/Pden_2262	21	25	26	23	Phenylalanine--tRNA ligase beta subunit	Cytoplasmic	8,6,4,7,12,14
A1B4B1/Pden_2263	5	5	4	6	Uncharacterized protein	Unknown	0
A1B4B2/Pden_2264	7	9	9	10	Glutathione S-transferase, N-terminal domain	Cytoplasmic	0
A1B4B5/Pden_2267	3	3	3	2	30S ribosomal protein S21	Unknown	6,1,8,4,7
A1B4B7/Pden_2269	3	1	2	2	Alcohol dehydrogenase, zinc-binding domain protein	Cytoplasmic	0
A1B4B8/Pden_2270	10	10	10	9	Phenylalanine--tRNA ligase alpha subunit	Cytoplasmic	8,6,4,7,12,14
A1B4B9/Pden_2271	3	2	3	3	Arsenate reductase	Cytoplasmic	0
A1B4C1/Pden_2273	5	5	6	5	50S ribosomal protein L20	Cytoplasmic	1,6,8,3,4,7
A1B4C2/Pden_2274	2	2	2	2	50S ribosomal protein L35	Unknown	6,1,8,4,7
A1B4C4/Pden_2276	19	21	15	18	Pyruvate kinase	Cytoplasmic	0
A1B4C5/Pden_2277	1	1	2	1	N-formylglutamate amidohydrolase	Cytoplasmic	6,7,2
A1B4D2/Pden_2285	7	7	5	7	Substrate-binding region of ABC-type glycine betaine transport system	Periplasmic	5,13
A1B4D3/Pden_2286	2	1	1	0	Sulfatase	Cytoplasmic	0
A1B4E4/Pden_2297	10	11	10	13	UDP-glucose pyrophosphorylase	Cytoplasmic	1
A1B4E6/Pden_2299	7	4	3	7	3-deoxy-manno-octulosonate cytidylyltransferase	Cytoplasmic	0
A1B4E7/Pden_2300	6	9	6	10	3',5'-bisphosphate nucleotidase	Cytoplasmic	8,4,7,12,14
A1B4E9/Pden_2302	32	33	30	37	Chaperone protein DnaK	Cytoplasmic	25
A1B4F0/Pden_2303	5	4	4	4	Chaperone protein DnaJ	Cytoplasmic	1,11,8,4,7,27
A1B4F2/Pden_2305	11	11	9	11	Ubiquinol-cytochrome c reductase iron-sulfur subunit	Membrane	0
A1B4F3/Pden_2306	4	3	3	4	Cytochrome b	Membrane	4,12,14
A1B4F4/Pden_2307	6	6	5	6	Cytochrome c1	Unknown	0
A1B4F5/Pden_2308	3	2	2	1	Ribokinase	Cytoplasmic	8,7,14
A1B4F8/Pden_2311	1	1	0	3	Short-chain dehydrogenase/reductase SDR	Cytoplasmic	0
A1B4G1/Pden_2314	2	3	3	3	Uncharacterized protein	Unknown	0
A1B4G2/Pden_2315	3	1	3	1	Mammalian cell entry related domain protein	Unknown	0
A1B4G6/Pden_2319	3	2	3	3	Methylenetetrahydrofolate reductase	Cytoplasmic	8,1,4,7,12,14
A1B4G8/Pden_2321	9	7	7	5	Orn/DAP/Arg decarboxylase 2	Cytoplasmic	6,1,4,7
A1B4H1/Pden_2324	6	9	5	5	FAD-dependent pyridine nucleotide-disulfide oxidoreductase	Cytoplasmic	12,9,10
A1B4H2/Pden_2325	5	6	4	3	Thiol peroxidase	Unknown	0
A1B4H8/Pden_2331	37	39	36	37	Valine--tRNA ligase	Cytoplasmic	8,6,4,7,12,14
A1B4H9/Pden_2332	0	1	1	2	Cobalamin biosynthesis CbiX protein	Cytoplasmic	1,6,4,7,12,14
A1B4I0/Pden_2333	4	5	5	5	Uncharacterized protein	Cytoplasmic	0
A1B4I5/Pden_2338	10	7	6	6	4-aminobutyrate aminotransferase apoenzyme	Cytoplasmic	8,6,4,7,12,14
A1B4I6/Pden_2339	11	8	12	11	Acetyl-coenzyme A carboxylase carboxyl transferase subunit alpha	Cytoplasmic	8,1,4,7,12,14
A1B4I9/Pden_2342	1	1	0	2	CDP-diacylglycerol--glycerol-3-phosphate 3-phosphatidyltransferase	Membrane	8,1,4,7,12,14
A1B4J2/Pden_2346	19	23	19	22	Glycine dehydrogenase)	Cytoplasmic	4,8,7,12,14,2
A1B4J4/Pden_2348	8	8	9	7	Aminomethyltransferase	Cytoplasmic	4,8,7,12,14,2
A1B4J5/Pden_2349	8	9	7	10	Short-chain dehydrogenase/reductase SDR	Cytoplasmic	0

A1B4L0/Pden_2364	17	19	19	18	Alpha-2-macroglobulin domain protein	Unknown	0
A1B4L2/Pden_2366	10	10	10	10	Aldehyde dehydrogenase)	Cytoplasmic	0
A1B4L3/Pden_2367	1	4	2	3	Alcohol dehydrogenase GroES domain protein	Cytoplasmic	0
A1B4L6/Pden_2370	2	3	1	2	Peptidase S16, lon domain protein	Cytoplasmic	8,7,2
A1B4L7/Pden_2371	3	5	6	7	Thioredoxin domain	Cytoplasmic	7,12,14,9,10
A1B4L8/Pden_2372	9	11	12	11	Exodeoxyribonuclease III Xth	Cytoplasmic	4,8,7,12,14,13
A1B4M0/Pden_2374	6	6	7	6	Two component transcriptional regulator, winged helix family	Cytoplasmic	1,6,8,4,7,9
A1B4M1/Pden_2375	0	2	2	0	GTP cyclohydrolase-2	Cytoplasmic	1,6,4,7,12,14
A1B4M3/Pden_2377	6	9	5	8	Short-chain dehydrogenase/reductase SDR	Cytoplasmic	0
A1B4N0/Pden_2384	1	2	3	2	Short-chain dehydrogenase/reductase SDR	Unknown	1,6,4,7,12,14
A1B4N1/Pden_2386	3	3	1	1	2,3-dihydroxybenzoate-AMP ligase	Cytoplasmic	1,6,4,7,12,14
A1B4N7/Pden_2393	11	11	7	11	6-phosphogluconate dehydrogenase, decarboxylating	Unknown	8,6,4,7,12,14
A1B4Q0/Pden_2406	5	4	3	4	D-alanine--D-alanine ligase	Cytoplasmic	12,7,14,9,10,19
A1B4Q1/Pden_2407	9	7	8	8	Mur ligase, middle domain protein	Membrane	12,7,14,9,10,19
A1B4Q4/Pden_2410	8	5	9	6	Uncharacterized protein	Cytoplasmic	0
A1B4Q5/Pden_2411	4	4	4	2	Outer membrane efflux protein	Cytoplasmic	0
A1B4Q6/Pden_2412	2	2	3	1	Type I secretion membrane fusion protein, HlyD family	Membrane	12,5,13,23
A1B4Q7/Pden_2413	5	4	4	5	ABC transporter related protein	Membrane	0
A1B4R8/Pden_2425	4	4	6	3	Uncharacterized protein	Membrane	0
A1B4R9/Pden_2426	14	15	15	13	Pyrrolo-quinoline quinone	OuterMembrane	0
A1B4S0/Pden_2427	10	10	11	9	GTPase Der	Membrane	20
A1B4S2/Pden_2429	16	16	15	14	Serine--tRNA ligase synthetase)	Cytoplasmic	8,1,4,7,12,14
A1B4S4/Pden_2431	1	2	2	1	LAO/AO transport system ATPase	Membrane	0
A1B4S6/Pden_2433	10	11	11	11	50S ribosomal protein L28	Cytoplasmic	6,1,8,4,7
A1B4T7/Pden_2444	2	1	0	1	Uncharacterized protein	Cytoplasmic	0
A1B4W5/Pden_2472	8	4	7	5	Carbohydrate ABC transporter substrate-binding protein, CUT1 family	Periplasmic	0
A1B4X1/Pden_2478	2	3	3	1	Putative transcriptional regulator, Crp/Fnr family	Cytoplasmic	1,6,8,4,7,9
A1B4X4/Pden_2481	12	12	6	9	von Willebrand factor, type A	Unknown	0
Q51664/Pden_2482	17	20	14	21	Protein NorQ	Cytoplasmic	0
A1B4X7/Pden_2484	5	6	5	4	Nitric oxide reductase, NorC subunit apoprotein	Membrane	0
O82951/Pden_2486	1	1	1	2	Genes for ORF1, ORF2, NorC and NorB	Unknown	0
Q51700/Pden_2487	39	36	43	39	Nitrite reductase	Periplasmic	0
Q51701/Pden_2488	4	7	6	7	Uroporphyrinogen-III C-methyltransferase	Cytoplasmic	1,6,4,7,12,14
A1B4Y3/Pden_2490	9	10	10	9	Cytochrome d1, heme region	Unknown	0
Q51703/Pden_2491	1	1	2	1	Protein NirD	Cytoplasmic	9
A1B4Y6/Pden_2493	1	2	2	2	Transcriptional regulator, AsnC family	Unknown	9
A1B4Y7/Pden_2494	2	3	3	1	Radical SAM domain protein	Cytoplasmic	0
A1B4Y8/Pden_2495	8	9	10	7	Cytochrome d1, heme region	Periplasmic	0
A1B4Z0/Pden_2497	57	51	58	56	Catalase-peroxidase	Cytoplasmic	11,4,12,14,2
A1B4Z2/Pden_2499	15	15	15	18	Probable transaldolase	Cytoplasmic	8,6,4,7,12,14
A1B4Z3/Pden_2500	1	2	1	1	Uncharacterized protein	Membrane	0
A1B4Z6/Pden_2503	2	1	1	2	Asparaginase	Cytoplasmic	0
A1B4Z7/Pden_2504	6	6	6	6	Uncharacterized protein	Unknown	0
A1B4Z8/Pden_2505	0	2	2	1	Dual-specificity RNA methyltransferase RlmN -C)-methyltransferase) -C)-methyltransferase)	Cytoplasmic	6,8,4,7,24
A1B4Z9/Pden_2506	13	11	12	14	2,3-bisphosphoglycerate-independent phosphoglycerate mutase	Cytoplasmic	4,8,7,12,14,2

A1B500/Pden_2507	1	4	2	2	Peptidase M23B	Unknown	0
A1B501/Pden_2508	16	16	16	17	Carboxyl-terminal protease	Membrane	0
A1B502/Pden_2509	2	1	2	2	RNA pyrophosphohydrolase nucleoside polyphosphate hydrolase)	Cytoplasmic	0
A1B503/Pden_2510	11	13	12	10	Lytic murein transglycosylase	Membrane	0
A1B505/Pden_2512	1	2	1	2	YbaK/prolyl-tRNA synthetase associated region	Unknown	0
A1B507/Pden_2514	11	9	11	8	2,3,4,5-tetrahydropyridine-2,6-dicarboxylate N-succinyltransferase	Cytoplasmic	8,1,4,7,12,14
A1B508/Pden_2515	5	3	4	5	Uncharacterized protein	Cytoplasmic	0
A1B511/Pden_2518	4	3	4	5	Iojap-like protein	Cytoplasmic	0
A1B513/Pden_2520	23	23	25	22	3-isopropylmalate dehydratase large subunit	Cytoplasmic	8,1,4,7,12,14
A1B515/Pden_2522	11	10	11	10	3-isopropylmalate dehydratase small subunit	Cytoplasmic	8,1,4,7,12,14
A1B522/Pden_2529	1	1	0	2	Cobyrinic acid a,c-diamide adenosyltransferase	Cytoplasmic	1,6,4,7,12,14
A1B524/Pden_2531	12	13	11	12	Cobalamin biosynthesis protein CobW	Cytoplasmic	1,6,4,7,12,14
A1B525/Pden_2532	2	2	1	0	Cobaltochelatase CobN subunit	Cytoplasmic	1
Q9R9K1/Pden_2533	1	1	0	2	Precorrin-3B synthase	Unknown	0
A1B527/Pden_2534	0	2	1	1	Precorrin-8X methylmutase	Unknown	1,6,4,7,12,14
A1B529/Pden_2536	1	1	0	2	Precorrin-3 methyltransferase	Cytoplasmic	1,6,4,7,12,14
A1B533/Pden_2540	0	2	0	1	Precorrin-4 C11-methyltransferase	Cytoplasmic	1,6,4,7,12,14
A1B534/Pden_2541	2	0	1	0	Hydrogenobyrinate a,c-diamide synthase	Cytoplasmic	8,1,4,7,12,14
A1B537/Pden_2544	1	1	2	1	Cytochrome c, class II	Periplasmic	4,12,14
A1B539/Pden_2546	9	9	11	9	DNA topoisomerase 4 subunit A	Cytoplasmic	8,6,3,4,7,12
A1B542/Pden_2549	5	4	5	4	ATP:cobalamin adenosyltransferase	Cytoplasmic	0
A1B543/Pden_2550	18	17	20	18	Electron transfer flavoprotein beta-subunit	Cytoplasmic	0
A1B544/Pden_2551	13	11	11	12	Electron transfer flavoprotein, alpha subunit	Unknown	0
A1B545/Pden_2552	11	8	8	12	3-hydroxyacyl-CoA dehydrogenase	Cytoplasmic	8,4,7,12,14
A1B547/Pden_2554	4	6	4	6	Dihydroorotase	Cytoplasmic	8,1,4,7,12,14
A1B548/Pden_2555	2	1	3	2	Aspartate carbamoyltransferase	Cytoplasmic	8,1,4,7,12,14
A1B549/Pden_2556	0	1	2	1	Uroporphyrinogen-III C-methyltransferase	Cytoplasmic	6,1,4,7
A1B553/Pden_2560	4	3	2	2	Enoyl-CoA hydratase/isomerase	Cytoplasmic	0
A1B557/Pden_2564	1	2	2	1	Amino acid adenylation domain	Cytoplasmic	1
A1B559/Pden_2566	12	12	15	14	Uncharacterized protein	Unknown	0
A1B560/Pden_2567	3	2	4	4	Periplasmic binding protein	Unknown	0
A1B565/Pden_2572	3	5	4	8	Ribulose-phosphate 3-epimerase	Cytoplasmic	8,6,4,7,12,14
A1B566/Pden_2573	2	4	2	3	33 kDa chaperonin	Cytoplasmic	25
A1B568/Pden_2575	1	2	1	0	Polynucleotide adenylyltransferase region	Cytoplasmic	6,8,4,7
A1B570/Pden_2577	1	0	2	0	23S rRNA mU-1939 methyltransferase	Cytoplasmic	6,8,4,7
A1B573/Pden_2580	5	6	5	5	Allergen V5/Tpx-1 family protein	Unknown	0
A1B576/Pden_2583	6	4	5	5	Ferrochelatase	Cytoplasmic	1,6,4,7,12,14
A1B577/Pden_2584	1	2	1	3	SAM-dependent methyltransferase	Cytoplasmic	0
A1B582/Pden_2589	4	4	3	5	Ubiquinone biosynthesis O-methyltransferase	Cytoplasmic	1,4,7,12,14
A1B584/Pden_2591	3	3	3	3	Ribosome maturation factor RimP	Cytoplasmic	20
A1B585/Pden_2592	23	22	24	23	Transcription termination/antitermination protein NusA	Cytoplasmic	1,6,8,4,7,9
A1B586/Pden_2593	1	1	1	3	Uncharacterized protein	Unknown	0
A1B587/Pden_2594	40	47	45	46	Translation initiation factor IF-2	Unknown	0
A1B590/Pden_2597	23	22	26	23	Arginine biosynthesis bifunctional protein (ArgJ) [Amino-acid acetyltransferase; Glutamate N-acetyltransferase]	Cytoplasmic	8,1,4,7,12,14

A1B591/Pden_2598	9	9	8	11	Peptidylprolyl isomerase	Membrane	0
A1B592/Pden_2599	34	41	39	42	Protein translocase subunit SecA	Cytoplasmic	12,5,13,23
A1B593/Pden_2600	0	3	1	1	Two component transcriptional regulator, winged helix family	Cytoplasmic	1,6,8,4,7,9
A1B595/Pden_2602	20	17	17	16	Branched chain amino acid aminotransferase apoenzyme	Cytoplasmic	8,6,4,7,12,14
A1B596/Pden_2603	0	2	1	1	PTS IIA-like nitrogen-regulatory protein PtsN	Cytoplasmic	0
A1B597/Pden_2604	15	15	17	12	SSU ribosomal protein S30P / sigma 54 modulation protein	Cytoplasmic	8
A1B598/Pden_2605	4	2	2	2	ABC transporter related protein	Cytoplasmic	12,5,13
A1B5A1/Pden_2608	1	2	4	0	KpsF/GutQ family protein	Unknown	8,7
A1B5A2/Pden_2609	3	4	5	5	3'-5' exonuclease	Cytoplasmic	0
A1B5A3/Pden_2610	25	26	26	25	TonB-dependent receptor	OuterMembrane	5
A1B5A6/Pden_2613	15	12	16	12	Formyltetrahydrofolate deformylase hydrolase)	Cytoplasmic	8,1,4,7,12,14
A1B5A7/Pden_2614	6	10	4	10	Bifunctional protein FolD 1 [Includes: Methylenetetrahydrofolate dehydrogenase ; Methenyltetrahydrofolate cyclohydrolase]	Cytoplasmic	8,1,4,7,12,14
A1B5B0/Pden_2617	4	2	4	2	Iron-containing alcohol dehydrogenase	Cytoplasmic	0
A1B5B2/Pden_2619	0	0	3	0	Transcriptional regulator, DeoR family	Cytoplasmic	6,1,8,4,7
A1B5B3/Pden_2620	3	2	4	2	Kynureninase	Cytoplasmic	4,8,7,12,14,2
A1B5B6/Pden_2623	4	4	4	4	Uncharacterized protein	Unknown	0
A1B5E7/Pden_2654	3	0	2	1	Efflux transporter, RND family, MFP subunit	Unknown	12,5,13
A1B5F2/Pden_2659	1	2	2	1	Murein endopeptidase, Metallo peptidase, MEROPS family M74	Periplasmic	0
A1B5F6/Pden_2663	7	5	6	4	Acetyl-CoA acetyltransferase	Cytoplasmic	0
A1B5G2/Pden_2669	1	1	2	3	Outer-membrane lipoprotein carrier protein	Unknown	5,23
A1B5G3/Pden_2670	2	2	2	2	2-octaprenyl-3-methyl-6-methoxy-1,4-benzoquinol hydroxylase / 2-octaprenyl-6-methoxyphenol hydroxylase	Membrane	1,4,7,12,14
A1B5G4/Pden_2671	3	3	4	3	Amidase	Cytoplasmic	0
A1B5G5/Pden_2672	4	4	4	3	Aminotransferase	Cytoplasmic	1
A1B5G6/Pden_2673	9	10	12	7	DNA translocase FtsK	Membrane	12
A1B5H1/Pden_2678	10	14	20	18	Uncharacterized protein	Unknown	0
A1B5H2/Pden_2679	4	2	5	5	Surface antigen	OuterMembrane	0
A1B5H3/Pden_2680	6	5	6	6	Glucokinase	Cytoplasmic	4,8,7,12,14,2
A1B5H5/Pden_2682	27	26	23	26	Adenosylhomocysteinase	Cytoplasmic	4,12,14
A1B5H7/Pden_2684	7	5	6	7	Uncharacterized protein	Unknown	0
A1B5H9/Pden_2686	11	11	12	14	Uncharacterized protein	Periplasmic	0
A1B5I0/Pden_2687	9	4	7	10	Uncharacterized protein	Cytoplasmic	0
A1B5I2/Pden_2689	1	2	3	2	Flavin reductase domain protein, FMN-binding protein	Unknown	0
A1B5I4/Pden_2691	4	5	6	5	Uncharacterized protein	Unknown	0
A1B5I5/Pden_2692	5	3	4	5	Mammalian cell entry related domain protein	Unknown	0
A1B5J5/Pden_2705	7	3	4	7	Carbonic anhydrase	Cytoplasmic	15
A1B5J7/Pden_2707	7	8	7	5	Leucyl aminopeptidase	Cytoplasmic	0
A1B5J8/Pden_2708	0	0	1	2	NLP/P60 protein	Cytoplasmic	0
A1B5J9/Pden_2709	2	3	3	3	Saccharopine dehydrogenase	Cytoplasmic	8,1,4,7,12,14
A1B5K1/Pden_2711	2	2	2	2	Uncharacterized protein	Cytoplasmic	0
A1B5K4/Pden_2714	4	5	5	6	Peptidase M16 domain protein	Unknown	0
A1B5K5/Pden_2715	7	6	6	8	Peptidase M16 domain protein	Unknown	0
A1B5K6/Pden_2716	5	4	6	5	Uncharacterized protein	Unknown	0

A1B5K8/Pden_2718	21	20	20	17	Bifunctional purine biosynthesis protein PurH [Includes: Phosphoribosylaminoimidazolecarboxamide formyltransferase ; IMP cyclohydrolase]	Cytoplasmic	8,1,4,7,12,14
A1B5K9/Pden_2719	3	0	1	1	Heparinase II/III family protein	Cytoplasmic	0
A1B5L0/Pden_2720	4	3	3	3	Fmu domain protein	Cytoplasmic	9
A1B5L3/Pden_2723	10	11	11	10	Selenide, water dikinase	Cytoplasmic	8,1,4,7,12,14
A1B5L6/Pden_2726	9	9	8	8	Protein-L-isoaspartate O-methyltransferase	Cytoplasmic	0
A1B5L7/Pden_2727	16	15	14	15	Type I secretion outer membrane protein, TolC family	OuterMembrane	0
A1B5L9/Pden_2729	12	7	10	9	Peptidase M23B	OuterMembrane	0
A1B5M0/Pden_2730	0	4	2	1	Protein-L-isoaspartate O-methyltransferase	Cytoplasmic	8,4,7
A1B5M1/Pden_2731	2	2	1	1	5'-nucleotidase SurE	Cytoplasmic	0
A1B5M5/Pden_2735	1	1	2	1	Glycerophosphoryl diester phosphodiesterase	Cytoplasmic	8,7,14
A1B5M6/Pden_2736	4	3	3	5	Endoribonuclease L-PSP	Unknown	0
A1B5M7/Pden_2737	1	0	0	2	Uncharacterized protein	Unknown	0
A1B5M8/Pden_2738	4	5	5	3	Chromosome partition protein Smc	Cytoplasmic	8,1,3,4,7,12
A1B5N3/Pden_2743	2	5	3	4	Elongation factor P	Cytoplasmic	0
A1B5P2/Pden_2752	2	1	2	3	Transcriptional regulator, GntR family	Cytoplasmic	6,1,8,4,7
A1B5P3/Pden_2753	4	3	3	4	5-carboxymethyl-2-hydroxymuconate delta-isomerase	Cytoplasmic	0
A1B5P4/Pden_2754	2	1	1	1	Short-chain dehydrogenase/reductase SDR	Cytoplasmic	0
A1B5P5/Pden_2755	17	18	16	16	Aldehyde dehydrogenase	Cytoplasmic	0
A1B5P6/Pden_2756	15	13	15	16	1-Cys peroxiredoxin	Cytoplasmic	0
A1B5P9/Pden_2759	49	49	49	48	Polyribonucleotide nucleotidyltransferase	Cytoplasmic	6,8,4,7,2
A1B5Q0/Pden_2760	5	6	8	9	30S ribosomal protein S15	Cytoplasmic	6,1,8,4,7
A1B5Q2/Pden_2762	2	2	1	3	Alanine racemase domain protein	Cytoplasmic	0
A1B5Q3/Pden_2763	24	21	21	20	Outer membrane porin	OuterMembrane	0
A1B5Q4/Pden_2764	6	5	6	5	Uncharacterized protein	Unknown	0
A1B5Q5/Pden_2765	23	23	24	20	Leucine--tRNA ligase	Cytoplasmic	8,6,4,7,12,14
A1B5Q7/Pden_2767	0	1	0	2	DNA polymerase III, delta subunit	Cytoplasmic	6,1,8,4,7
A1B5R3/Pden_2773	2	0	1	1	Glycosyl transferase, group 1	Cytoplasmic	0
A1B5R5/Pden_2775	23	21	21	19	Aspartate-semialdehyde dehydrogenase	Cytoplasmic	8,1,4,7,12,14
A1B5R6/Pden_2776	15	9	13	18	Uncharacterized protein	Unknown	0
A1B5S1/Pden_2781	7	8	7	6	Two component transcriptional regulator, Fis family	Cytoplasmic	9
A1B5S2/Pden_2782	8	9	10	10	Histone family protein nucleoid-structuring protein H-NS	Unknown	9
A1B5S4/Pden_2784	8	11	10	10	Protein-export protein SecB	Cytoplasmic	25,3,5,23
A1B5S5/Pden_2785	2	2	2	1	FxsA cytoplasmic membrane protein	Membrane	0
A1B5S6/Pden_2786	7	10	8	9	Import inner membrane translocase, subunit Tim44	Unknown	0
A1B5T0/Pden_2790	7	6	9	6	ATP-dependent protease ATPase subunit HslU	Cytoplasmic	26
A1B5T3/Pden_2793	5	8	7	6	Thioredoxin	Cytoplasmic	7,12,14,9,10
A1B5T4/Pden_2794	1	3	2	1	UvrD-like DNA helicase, C-terminal domain	Cytoplasmic	4,8,7,12,14,13
A1B5T7/Pden_2797	2	0	3	1	Uncharacterized protein	Cytoplasmic	6,8,4,7
A1B5T8/Pden_2798	6	8	7	8	4-hydroxy-tetrahydrodipicolinate reductase	Cytoplasmic	8,1,4,7,12,14
A1B5U1/Pden_2801	3	2	2	4	tRNA pseudouridine synthase B synthase)	Cytoplasmic	6,8,4,7
A1B5U3/Pden_2803	1	1	1	2	Beta-lactamase domain protein	Unknown	0
A1B5V3/Pden_2813	3	3	3	3	Shikimate dehydrogenase)	Cytoplasmic	8,1,4,7,12,14
A1B5W0/Pden_2820	0	1	2	1	RNA polymerase sigma factor	Cytoplasmic	6,1,8,4,7
A1B5W2/Pden_2822	5	5	6	4	Response regulator receiver protein	Cytoplasmic	1,6,8,4,7,9
A1B5W3/Pden_2823	1	1	0	2	2-nitropropane dioxygenase, NPD	Unknown	0

A1B5W6/Pden_2826	0	2	1	2	Protein FdhE homolog	Cytoplasmic	0
A1B5W9/Pden_2829	0	1	0	2	Formate dehydrogenase catalytic subunit	Periplasmic	4,12,14
A1B5X3/Pden_2833	1	4	1	5	Chromosomal replication initiator protein DnaA	Cytoplasmic	6,8,4,7,9
A1B5X4/Pden_2834	25	25	26	24	DNA polymerase III subunit beta	Cytoplasmic	6,1,8,4,7
A1B5X6/Pden_2836	12	11	8	9	Adenine deaminase	Cytoplasmic	4,8,7,12,14,2
A1B5X8/Pden_2838	2	3	3	3	Uncharacterized protein	Unknown	0
A1B5X9/Pden_2839	4	2	6	6	Uncharacterized protein	Unknown	0
A1B5Y0/Pden_2840	9	9	9	10	Acyl-CoA dehydrogenase	Cytoplasmic	0
A1B5Y2/Pden_2842	1	0	4	1	Succinyl-diaminopimelate desuccinylase	Cytoplasmic	8,1,4,7,12,14
A1B5Y5/Pden_2845	8	6	5	7	Glutamate--cysteine ligase	Cytoplasmic	6,1,4,7
A1B5Z0/Pden_2850	2	2	3	3	Nucleotide-binding protein Pden_2850	Cytoplasmic	0
A1B5Z2/Pden_2852	30	33	30	28	Phosphoenolpyruvate carboxykinase	Cytoplasmic	1,8,7,14
A1B5Z3/Pden_2853	3	3	1	2	Ribonuclease R	Cytoplasmic	0
A1B5Z6/Pden_2856	13	12	13	11	Formate dehydrogenase alpha subunit	Cytoplasmic	4,7,12,14
A1B5Z7/Pden_2857	4	1	2	2	Formate dehydrogenase beta subunit	Cytoplasmic	0
A1B600/Pden_2860	1	3	3	2	Phosphoribosylformylglycinamidine synthase subunit PurS	Unknown	8,1,4,7,12,14
A1B601/Pden_2861	14	11	13	10	Phosphoribosylaminoimidazole-succinocarboxamide synthase	Cytoplasmic	8,1,4,7,12,14
A1B602/Pden_2862	7	5	5	6	Uncharacterized protein	Cytoplasmic	0
A1B605/Pden_2865	0	1	2	1	Uncharacterized protein	Cytoplasmic	0
A1B608/Pden_2868	6	4	6	7	Acyl-CoA dehydrogenase domain protein	Cytoplasmic	0
A1B610/Pden_2870	6	7	4	3	Acetyl-CoA acetyltransferase	Cytoplasmic	0
A1B611/Pden_2871	5	6	7	9	3-hydroxyacyl-CoA dehydrogenase	Cytoplasmic	4,8,7,12,14,2
A1B612/Pden_2872	6	6	5	4	PfkB domain protein	Cytoplasmic	0
A1B613/Pden_2873	0	1	2	2	Endonuclease III lyase)	Cytoplasmic	4,8,7,12,14,13
A1B615/Pden_2875	9	9	10	10	OmpA/MotB domain protein	OuterMembrane	0
A1B616/Pden_2876	16	17	15	18	ATP synthase subunit b sector subunit b)	Unknown	7,4,12,14,5,13
A1B617/Pden_2877	13	11	14	14	ATP synthase subunit b sector subunit b)	Unknown	7,4,12,14,5,13
A1B619/Pden_2879	2	1	1	2	ATP synthase subunit a	Membrane	7,4,12,14,5,13
A1B622/Pden_2882	19	13	14	15	5'-Nucleotidase domain protein	Periplasmic	4,8,7,12,14,2
A1B624/Pden_2884	3	1	1	1	Dihydroorotate dehydrogenase	Membrane	8,1,4,7,12,14
A1B625/Pden_2885	0	0	0	2	Serine O-acetyltransferase	Cytoplasmic	8,1,4,7,12,14
A1B643/Pden_2903	4	5	3	8	Aminodeoxychorismate lyase	Unknown	0
A1B644/Pden_2904	21	20	20	17	3-oxoacyl-[acyl-carrier-protein] synthase 2	Cytoplasmic	8,1,4,7,12,14
A1B647/Pden_2907	3	1	2	2	Acetyl-CoA acetyltransferase	Cytoplasmic	0
A1B648/Pden_2908	2	0	3	0	Short-chain dehydrogenase/reductase SDR	Unknown	0
A1B652/Pden_2912	6	6	4	8	Replication protein, putative	Cytoplasmic	0
A1B653/Pden_2913	3	2	1	2	Cobyrinic acid a,c-diamide synthase	Cytoplasmic	0
A1B656/Pden_2916	4	3	4	5	Biotin synthase 2	Cytoplasmic	1,6,4,7,12,14
A1B658/Pden_2918	2	3	1	1	ATP-dependent dethiobiotin synthetase BioD	Cytoplasmic	1,6,4,7,12,14
A1B659/Pden_2919	1	2	1	0	Adenosylmethionine-8-amino-7-oxononanoate aminotransferase apoenzyme	Unknown	1,6,4,7,12,14
A1B662/Pden_2922	0	2	2	2	Transcriptional regulator, GntR family	Cytoplasmic	6,1,8,4,7
A1B673/Pden_2933	19	16	16	19	Dihydroxy-acid dehydratase	Cytoplasmic	8,1,4,7,12,14
A1B678/Pden_2938	3	2	2	2	Efflux transporter, RND family, MFP subunit	Membrane	12,5,13
A1B683/Pden_2943	1	2	4	1	RND efflux system, outer membrane lipoprotein, NodT family	OuterMembrane	0
A1B684/Pden_2944	23	25	19	24	Phosphomethylpyrimidine synthase	Cytoplasmic	1,6,4,7,12,14

A1B686/Pden_2946	6	6	6	5	PfkB domain protein	Unknown	0
A1B688/Pden_2948	2	7	2	5	Antifreeze protein, type I	Cytoplasmic	0
A1B690/Pden_2950	2	2	2	0	Uncharacterized protein	Unknown	0
A1B691/Pden_2951	16	17	17	18	Toxic anion resistance family protein	Cytoplasmic	0
A1B695/Pden_2955	18	16	16	16	Carboxynorspermidine dehydrogenase	Cytoplasmic	0
A1B696/Pden_2956	3	5	3	3	Carboxynorspermidine/carboxyspermidine decarboxylase	Cytoplasmic	6,1,4,7
A1B697/Pden_2957	1	2	4	1	Oxidoreductase domain protein	Unknown	0
A1B698/Pden_2958	2	3	5	3	Transcriptional regulator, BadM/Rrf2 family	Unknown	0
A1B699/Pden_2959	2	5	3	4	Uncharacterized protein	Unknown	0
A1B6A1/Pden_2961	34	33	28	35	Isocitrate dehydrogenase [NADP]	Cytoplasmic	8,4,7,12,14
A1B6A2/Pden_2962	6	6	7	6	Phosphoribosylformylglycinamidine synthase subunit PurQ	Cytoplasmic	8,1,4,7,12,14
A1B6A3/Pden_2963	1	1	2	1	Integral membrane sensor signal transduction histidine kinase	Membrane	0
A1B6A4/Pden_2964	17	18	18	21	Two component, sigma54 specific, transcriptional regulator, Fis family	Cytoplasmic	1,6,8,4,7,9
A1B6A5/Pden_2965	33	29	28	28	Ribonuclease E	Cytoplasmic	6,8,4,7,2
A1B6A7/Pden_2967	2	2	1	0	Enoyl-CoA hydratase/isomerase	Cytoplasmic	0
A1B6A9/Pden_2969	14	13	12	16	50S ribosomal protein L13	Cytoplasmic	6,1,8,4,7
A1B6B0/Pden_2970	9	9	12	13	30S ribosomal protein S9	Cytoplasmic	6,1,8,4,7
A1B6E4/Pden_3005	1	0	2	2	Uncharacterized protein	Unknown	0
A1B6E6/Pden_3007	0	1	2	1	TonB-dependent siderophore receptor	OuterMembrane	5,13
A1B6L9/Pden_3082	13	15	16	11	Outer membrane autotransporter barrel domain	OuterMembrane	0
A1B6M0/Pden_3083	1	1	3	3	Putative outer membrane protein	OuterMembrane	0
A1B6M1/Pden_3084	4	3	4	5	Lipoprotein, putative	Unknown	0
A1B6M2/Pden_3085	0	0	0	3	Uncharacterized protein	Unknown	0
A1B6M3/Pden_3086	3	2	2	2	Uncharacterized protein	Unknown	0
A1B6Q2/Pden_3115	1	0	1	2	Uncharacterized protein	Membrane	0
A1B6U3/Pden_3156	1	3	1	3	Histone family protein nucleoid-structuring protein H-NS	Unknown	9
A1B6Z3/Pden_3206	3	3	2	3	Transcriptional regulator, GntR family	Cytoplasmic	6,1,8,4,7
A1B703/Pden_3216	5	5	6	6	Tartronate semialdehyde reductase	Cytoplasmic	0
A1B711/Pden_3224	2	3	2	2	Acyl-CoA dehydrogenase domain protein	Cytoplasmic	0
A1B716/Pden_3229	2	2	1	2	Uncharacterized protein	Cytoplasmic	0
A1B718/Pden_3231	2	3	1	3	Putative transcriptional regulator, IclR family	Cytoplasmic	1,6,8,4,7,9
A1B781/Pden_3297	1	3	1	1	Heavy metal translocating P-type ATPase	Membrane	0
A1B7C8/Pden_3349	1	3	1	3	Nucleoid protein H-NS	Unknown	9
A1B7G9/Pden_3390	2	1	0	1	Uncharacterized protein	Unknown	0
A1B7I4/Pden_3407	0	1	2	1	Carbohydrate ABC transporter ATP-binding protein, CUT1 family	Membrane	5
A1B7L4/Pden_3437	2	1	1	1	Fumarylacetoacetate hydrolase	Cytoplasmic	0
A1B7M2/Pden_3445	3	2	2	2	Efflux transporter, RND family, MFP subunit	Membrane	12,5,13
A1B7P7/Pden_3470	6	8	7	6	Uncharacterized protein	Unknown	0
A1B7W6/Pden_3540	1	1	2	3	TRAP dicarboxylate transporter-DctP subunit	Unknown	5
A1B7X8/Pden_3552	9	10	12	14	ATP-dependent carboxylate-amine ligase domain protein, ATP-grasp	Cytoplasmic	0
A1B7X9/Pden_3553	8	6	7	6	NAD-dependent epimerase/dehydratase	Unknown	0
A1B7Y1/Pden_3555	2	1	3	3	Uncharacterized protein	Cytoplasmic	0
A1B7Y2/Pden_3556	12	14	11	10	N-acetylneuraminate synthase	Cytoplasmic	1,8,7,14
A1B7Y3/Pden_3557	16	13	18	13	DegT/DnrJ/EryC1/StrS aminotransferase	Cytoplasmic	0

A1B810/Pden_3586	2	0	2	1	Transglutaminase domain protein	Unknown	0
A1B811/Pden_3587	2	2	2	2	Uncharacterized protein	Membrane	0
A1B812/Pden_3588	4	4	2	4	Amidohydrolase	Cytoplasmic	0
A1B832/Pden_3609	11	15	18	18	Lon protease	Cytoplasmic	11,8,7,13
A1B835/Pden_3612	26	25	26	29	Isoleucine--tRNA ligase	Cytoplasmic	8,6,4,7,12,14
A1B841/Pden_3618	2	2	4	3	Glutathione S-transferase, C-terminal domain	Cytoplasmic	0
A1B842/Pden_3619	1	2	2	2	4-hydroxy-3-methylbut-2-enyl diphosphate reductase	Cytoplasmic	8,1,4,7,12,14
A1B843/Pden_3620	1	2	5	4	Adenine phosphoribosyltransferase	Cytoplasmic	8,1,4,7,12,14
A1B844/Pden_3621	1	2	3	1	Uncharacterized protein	Unknown	0
A1B848/Pden_3625	0	0	1	4	AAA ATPase	Cytoplasmic	0
A1B852/Pden_3629	11	11	11	12	Uroporphyrinogen decarboxylase	Cytoplasmic	6,1,4,7
A1B853/Pden_3630	10	11	11	10	Porphobilinogen deaminase	Cytoplasmic	6,1,8,4,7
A1B856/Pden_3633	4	1	2	1	Isovaleryl-CoA dehydrogenase	Cytoplasmic	0
A1B859/Pden_3636	7	8	9	6	OmpW family protein	OuterMembrane	0
A1B862/Pden_3639	1	0	2	3	3-methylcrotonoyl-CoA carboxylase, alpha subunit	Cytoplasmic	0
A1B868/Pden_3645	1	3	4	4	Uncharacterized protein	Unknown	0
A1B871/Pden_3648	1	2	1	2	Nucleoid-associated protein Pden_3648	Unknown	0
A1B872/Pden_3649	10	11	9	10	DNA polymerase III, subunits gamma and tau	Cytoplasmic	6,1,8,4,7
A1B873/Pden_3650	1	1	1	2	Uncharacterized protein	Cytoplasmic	0
A1B875/Pden_3652	0	1	0	3	ABC transporter related protein	Cytoplasmic	0
A1B876/Pden_3653	4	4	4	4	Cold-shock DNA-binding protein family	Cytoplasmic	9
A1B877/Pden_3654	72	66	64	58	60 kDa chaperonin	Cytoplasmic	25
A1B878/Pden_3655	2	2	1	1	Glycosyl transferase, group 1	Cytoplasmic	0
A1B880/Pden_3657	1	1	1	2	Uncharacterized protein	Unknown	0
A1B882/Pden_3659	13	12	12	13	Inorganic diphosphatase	Cytoplasmic	0
A1B883/Pden_3660	3	4	3	4	HAD-superfamily subfamily IIA hydrolase like protein	Cytoplasmic	0
A1B885/Pden_3662	2	2	0	1	Riboflavin biosynthesis protein	Cytoplasmic	8,1,4,7,12,14
A1B887/Pden_3664	4	5	6	5	L-threonine aldolase	Cytoplasmic	4,8,7,12,14,2
A1B890/Pden_3667	4	4	5	5	Bifunctional enzyme IspD/IspF [Includes: 2-C-methyl-D-erythritol 4-phosphate cytidylyltransferase ; 2-C-methyl-D-erythritol 2,4-cyclodiphosphate synthase]	Cytoplasmic	8,1,4,7,12,14
A1B891/Pden_3668	6	4	5	4	Ribose-5-phosphate isomerase A	Cytoplasmic	4,7,12,14
A1B892/Pden_3669	12	11	10	8	NADPH-glutathione reductase	Cytoplasmic	12,9,10
A1B893/Pden_3670	14	22	16	17	Protease FtsH subunit HflK	Cytoplasmic	0
A1B894/Pden_3671	21	23	24	23	Protease FtsH subunit HflC	Cytoplasmic	0
A1B896/Pden_3673	9	9	12	9	Aminopyrimidine aminohydrolase	Unknown	1,6,4,7,12,14
A1B898/Pden_3675	2	3	3	2	Uncharacterized protein	Cytoplasmic	0
A1B8A0/Pden_3677	0	1	0	2	Transcriptional regulator, GntR family	Cytoplasmic	6,1,8,4,7
A1B8A1/Pden_3678	13	10	15	14	Homoserine dehydrogenase	Cytoplasmic	8,6,4,7,12,14
A1B8A4/Pden_3681	11	8	7	9	Methylmalonyl-CoA mutase	Cytoplasmic	0
A1B8A7/Pden_3684	10	11	9	12	Biotin carboxyl carrier protein / biotin carboxylase	Cytoplasmic	0
A1B8B1/Pden_3688	7	6	6	6	Propionyl-CoA carboxylase carboxyltransferase subunit	Cytoplasmic	0
A1B8B2/Pden_3689	7	4	4	6	Alanine racemase	Cytoplasmic	8,1,4,7,12,14
A1B8B5/Pden_3692	11	10	11	12	PhoH family protein	Cytoplasmic	0
A1B8B7/Pden_3694	3	5	4	2	CBS domain containing protein	Membrane	0
A1B8B8/Pden_3695	3	1	2	5	GCN5-related N-acetyltransferase	Cytoplasmic	0
A1B8B9/Pden_3696	19	20	18	16	GMP synthase [glutamine-hydrolyzing]	Cytoplasmic	8,1,4,7,12,14

A1B8C0/Pden_3697	3	3	3	3	Thiamine monophosphate synthase	Cytoplasmic	1,6,4,7,12,14
A1B8C1/Pden_3698	1	1	1	2	tRNA -methyltransferase TrmJ /uridine-2'-O)-methyltransferase)	Cytoplasmic	6,8,4,7
A1B8C3/Pden_3700	20	13	14	18	Carboxypeptidase Taq, Metallo peptidase, MEROPS family M32	Cytoplasmic	0
A1B8C4/Pden_3701	2	1	2	3	tRNA-2-methylthio-N-dimethylallyladenosine synthase adenosine tRNA methylthiotransferase MiaB) A37 methylthiotransferase)	Cytoplasmic	6,8,4,7
A1B8C5/Pden_3702	3	3	4	4	Glutamate--putrescine ligase	Cytoplasmic	8,1,4,7,12,14
A1B8C8/Pden_3705	8	6	6	6	Amino acid ABC transporter substrate-binding protein, PAAT family	Periplasmic	0
A1B8C9/Pden_3706	2	1	3	3	Amino acid ABC transporter ATP-binding protein, PAAT family	Membrane	0
A1B8D0/Pden_3707	2	0	1	2	Uncharacterized protein	Unknown	0
A1B8D4/Pden_3711	21	16	18	22	CTP synthase	Cytoplasmic	8,1,4,7,12,14
A1B8D7/Pden_3714	2	6	5	7	Short-chain dehydrogenase/reductase SDR	Unknown	0
A1B8D9/Pden_3716	18	20	20	23	Citrate synthase	Cytoplasmic	8,4,7,12,14
A1B8E0/Pden_3717	16	16	18	16	Glutamate--tRNA ligase 2	Cytoplasmic	8,6,4,7,12,14
A1B8E4/Pden_3721	3	2	0	1	Uncharacterized protein	Cytoplasmic	0
A1B8E8/Pden_3725	28	28	29	25	Elongation factor Ts	Cytoplasmic	0
A1B8E9/Pden_3726	20	16	18	18	30S ribosomal protein S2	Cytoplasmic	6,1,8,4,7
A1B8G9/Pden_3746	0	1	0	2	Putative transcriptional regulator, XRE family	Unknown	0
A1B8K6/Pden_3784	5	6	3	4	Cytidylate kinase	Cytoplasmic	8,6,4,7,12,14
A1B8K7/Pden_3785	1	4	1	5	2-nitropropane dioxygenase, NPD	Unknown	0
A1B8K8/Pden_3786	48	43	48	43	30S ribosomal protein S1	Cytoplasmic	6,1,8,4,7
A1B8K9/Pden_3787	3	5	3	5	Integration host factor subunit beta	Cytoplasmic	1,6,8,4,7,9
A1B8L0/Pden_3788	0	2	2	3	Uncharacterized protein	Membrane	0
A1B8L1/Pden_3789	5	5	5	2	N-anthranilate isomerase	Unknown	8,1,4,7,12,14
A1B8L3/Pden_3791	12	11	12	10	Tryptophan synthase beta chain	Cytoplasmic	0
A1B8L4/Pden_3792	1	2	1	3	DSBA oxidoreductase	Unknown	0
A1B8L7/Pden_3795	6	6	5	3	Polysaccharide deacetylase	Cytoplasmic	6,1,8,4,7
A1B8M8/Pden_3806	5	11	10	11	Single-stranded DNA-binding protein	Cytoplasmic	4,8,7,12,14,13
A1B8N1/Pden_3809	8	6	7	7	Delta-aminolevulinic acid dehydratase	Cytoplasmic	6,1,4,7
A1B8N3/Pden_3811	3	4	5	4	Transcription-repair-coupling factor	Cytoplasmic	3,9
A1B8N4/Pden_3812	16	19	18	17	ATP-dependent Clp protease, ATP-binding subunit clpA	Cytoplasmic	0
A1B8N5/Pden_3813	3	7	4	3	Hydroxyacylglutathione hydrolase	Cytoplasmic	4,7,12,14,2
A1B8N7/Pden_3815	14	13	14	13	ATP synthase subunit delta sector subunit delta)	Cytoplasmic	7,4,12,14,5,13
A1B8N8/Pden_3816	51	47	46	48	ATP synthase subunit alpha	Cytoplasmic	7,4,12,14,5,13
A1B8N9/Pden_3817	22	18	20	20	ATP synthase gamma chain	Membrane	7,4,12,14,5,13
A1B8P0/Pden_3818	33	32	29	29	ATP synthase subunit beta	Cytoplasmic	7,4,12,14,5,13
A1B8P1/Pden_3819	6	7	7	6	ATP synthase epsilon chain	Cytoplasmic	7,4,12,14,5,13
A1B8P3/Pden_3821	1	1	2	1	Alpha/beta hydrolase fold protein	Cytoplasmic	0
A1B8P4/Pden_3822	4	4	4	4	DSBA oxidoreductase	Cytoplasmic	0
A1B8P5/Pden_3823	15	12	11	11	Ribose-phosphate pyrophosphokinase	Cytoplasmic	8,1,4,7,12,14
A1B8P8/Pden_3826	1	2	2	1	RNA methyltransferase, TrmH family, group 3	Cytoplasmic	6,8,4,7
A1B8R1/Pden_3839	1	2	2	2	Uncharacterized protein	Cytoplasmic	6,11,8,4,7
A1B8R2/Pden_3840	6	6	5	8	Uncharacterized protein	Cytoplasmic	0
A1B8R3/Pden_3841	2	3	3	2	Helicase domain protein	Cytoplasmic	0
A1B8S8/Pden_3856	1	1	1	2	Substrate-binding region of ABC-type glycine betaine transport system	Periplasmic	0
A1B8T2/Pden_3860	2	1	0	1	NUDIX hydrolase	Cytoplasmic	0

A1B8T3/Pden_3861	11	11	13	13	Pyridoxal-5'-phosphate-dependent enzyme, beta subunit	Cytoplasmic	8,1,4,7,12,14
A1B8T7/Pden_3865	15	23	20	20	DNA gyrase subunit A	Cytoplasmic	1,6,8,3,4,7
A1B8U3/Pden_3871	1	0	2	2	Uncharacterized protein	Cytoplasmic	0
A1B8U4/Pden_3872	22	22	21	20	Glutamate dehydrogenase	Unknown	8,6,4,7,12,14
A1B8U5/Pden_3873	6	6	5	7	Crotonyl-CoA reductase	Cytoplasmic	0
A1B8U7/Pden_3875	0	1	0	3	Methylmalonyl-CoA mutase	Cytoplasmic	0
A1B8U9/Pden_3877	2	2	2	2	Alkyl hydroperoxide reductase AhpD	Unknown	0
A1B8V0/Pden_3878	13	12	11	12	Signal recognition particle protein	Membrane	12,3,5,13,23,22
A1B8V2/Pden_3880	1	1	3	3	Chorismate mutase	Cytoplasmic	4,7,12,14
A1B8V3/Pden_3881	6	11	11	13	30S ribosomal protein S16	Cytoplasmic	6,1,8,4,7
A1B8V4/Pden_3882	1	2	3	1	Ribosome maturation factor RimM	Cytoplasmic	6,8,4,7,20
A1B8V5/Pden_3883	2	2	1	2	tRNA -)-methyltransferase	Cytoplasmic	0
A1B8V6/Pden_3884	16	11	15	16	50S ribosomal protein L19	Cytoplasmic	6,1,8,4,7
A1B8V7/Pden_3885	2	4	4	3	50S ribosomal protein L31	Cytoplasmic	6,1,8,4,7
A1B8V9/Pden_3887	3	3	2	3	Chromosome partitioning protein	Cytoplasmic	0
A1B8W0/Pden_3888	0	2	2	1	Transcriptional regulator, Fis family	Unknown	12,5,13
A1B8W2/Pden_3890	24	24	22	22	Acetyltransferase component of pyruvate dehydrogenase complex	Cytoplasmic	4,7,12,14
A1B8W3/Pden_3891	21	23	22	23	Transketolase, central region	Cytoplasmic	0
A1B8W4/Pden_3892	15	19	17	19	Pyruvate dehydrogenase E1 component subunit alpha	Cytoplasmic	1,4,7,12,14
A1B8W6/Pden_3894	3	2	3	1	Acetylornithine deacetylase or succinyl-diaminopimelate desuccinylase	Cytoplasmic	0
A1B8W9/Pden_3897	1	2	2	3	Deoxyribose-phosphate aldolase	Cytoplasmic	4,8,7,12,14,2
A1B8X0/Pden_3898	11	11	6	9	Aldehyde dehydrogenase)	Cytoplasmic	0
A1B8X3/Pden_3901	8	4	6	8	ATPase associated with various cellular activities, AAA_3	Cytoplasmic	0
A1B8X4/Pden_3902	2	1	1	1	Uncharacterized protein	Unknown	0
A1B8X5/Pden_3903	1	1	1	4	Conserved hypothetical membrane protein	Membrane	0
A1B8X8/Pden_3906	3	2	3	2	3-hydroxyacyl-[acyl-carrier-protein] dehydratase FabZ -hydroxymyristoyl-[acyl-carrier-protein] dehydratase) -hydroxymyristoyl-ACP dehydrase)	Cytoplasmic	8,1,4,7,12,14
A1B8X9/Pden_3907	8	7	6	7	Acyl-[acyl-carrier-protein]--UDP-N-acetylglucosamine O-acyltransferase	Cytoplasmic	8,1,4,7,12,14
A1B8Y0/Pden_3908	1	1	2	3	Uncharacterized protein	Unknown	0
A1B8Y2/Pden_3910	3	3	2	4	Endoribonuclease L-PSP	Cytoplasmic	0
A1B8Z4/Pden_3922	2	2	1	1	Transcriptional regulator, IclR family	Cytoplasmic	1,6,8,4,7,9
A1B8Z8/Pden_3926	13	11	13	11	Phosphoribosylformylglycinamidine cyclo-ligase	Cytoplasmic	8,1,4,7,12,14
A1B8Z9/Pden_3927	3	1	3	2	Phosphoribosylglycinamide formyltransferase	Cytoplasmic	8,1,4,7,12,14
A1B902/Pden_3930	4	6	4	4	Amino acid/amide ABC transporter ATP-binding protein 2, HAAT family	Unknown	0
A1B903/Pden_3931	4	3	2	4	Amino acid/amide ABC transporter ATP-binding protein 1, HAAT family	Membrane	0
A1B904/Pden_3932	22	18	18	18	Amino acid/amide ABC transporter substrate-binding protein, HAAT family	Periplasmic	0
A1B906/Pden_3934	25	24	25	21	Phospho-2-dehydro-3-deoxyheptonate aldolase	Unknown	8,1,4,7,12,14
A1B908/Pden_3936	1	2	3	4	Guanylate kinase	Cytoplasmic	0
A1B909/Pden_3937	0	2	2	1	Transferase hexapeptide protein	Cytoplasmic	0
A1B910/Pden_3938	1	2	2	4	PAS/PAC sensor signal transduction histidine kinase	Membrane	9
A1B911/Pden_3939	13	8	10	8	Amidophosphoribosyltransferase	Cytoplasmic	8,1,4,7,12,14
A1B912/Pden_3940	2	2	3	3	Colicin V production protein	Membrane	1,4,12,14
A1B913/Pden_3941	4	3	3	4	DNA repair protein RadA	Membrane	4,8,7,12,14,13
A1B915/Pden_3943	2	3	1	1	Pyrimidine 5'-nucleotidase	Cytoplasmic	0

A1B916/Pden_3944	5	9	6	5	Transcriptional regulator, GntR family	Cytoplasmic	6,1,8,4,7
A1B925/Pden_3953	6	10	9	8	Tryptophan--tRNA ligase	Cytoplasmic	8,6,4,7,12,14
A1B928/Pden_3956	2	2	3	1	Bifunctional uridylyltransferase/uridylyl-removing enzyme [Includes: [Protein-PII]-UMP uridylyl-removing enzyme ; [Protein-PII] uridylyltransferase]	Cytoplasmic	6,9
A1B929/Pden_3957	8	8	8	7	Amino acid/amide ABC transporter substrate-binding protein, HAAT family	Unknown	0
A1B930/Pden_3958	4	3	6	5	Ribosomal RNA small subunit methyltransferase I - methyltransferase RsmI)	Cytoplasmic	6,8,4,7,24
A1B932/Pden_3960	8	8	11	9	Glutathione synthetase	Cytoplasmic	0
A1B934/Pden_3962	14	12	10	12	Phosphopantothenoylcysteine decarboxylase / Phosphopantothenate-cysteine ligase	Cytoplasmic	4,8,7,12,14,2
A1B935/Pden_3963	7	8	8	4	Cysteine desulfurase	Unknown	0
A1B936/Pden_3964	15	13	19	19	Iron-regulated ABC transporter membrane component SufB	Cytoplasmic	1,3
A1B938/Pden_3966	13	15	13	17	FeS assembly ATPase SufC	Cytoplasmic	5
A1B939/Pden_3967	19	14	14	15	SufBD protein	Cytoplasmic	1,3
A1B942/Pden_3970	2	3	4	4	Cysteine desulfurase	Cytoplasmic	8,6,4,7,12,14
A1B943/Pden_3971	19	20	20	20	Phosphoribosylformylglycinamidine synthase subunit PurL	Cytoplasmic	8,1,4,7,12,14
A1B944/Pden_3972	8	7	10	7	Transcriptional regulator, LysR family	Cytoplasmic	6,1,8,4,7
A1B945/Pden_3973	8	8	9	9	Glutamate racemase	Cytoplasmic	12,7,14,9,10,19
A1B946/Pden_3974	4	3	4	5	Cytochrome c-type biogenesis protein CcmE	Unknown	8,3,4,7
A1B950/Pden_3978	1	0	2	1	Cytochrome C biogenesis protein	Membrane	0
A1B952/Pden_3980	3	3	3	4	NUDIX hydrolase	Unknown	0
A1B953/Pden_3981	1	1	3	2	Uncharacterized protein	Membrane	0
A1B956/Pden_3984	15	14	18	16	Aminotransferase	Cytoplasmic	0
A1B962/Pden_3990	1	3	2	3	Ribosomal RNA large subunit methyltransferase E - methyltransferase)	Cytoplasmic	0
A1B965/Pden_3993	7	6	8	9	Uridylate kinase	Cytoplasmic	8,1,4,7,12,14
A1B966/Pden_3994	4	5	5	6	Ribosome-recycling factor	Cytoplasmic	3,12
A1B967/Pden_3995	2	4	2	2	Isoprenyl transferase	Cytoplasmic	0
A1B969/Pden_3997	2	2	0	0	1-deoxy-D-xylulose 5-phosphate reductoisomerase	Cytoplasmic	8,1,4,7,12,14
A1B970/Pden_3998	1	1	1	2	Zinc metalloprotease	Membrane	0
A1B971/Pden_3999	37	32	42	36	Outer membrane protein assembly factor BamA	OuterMembrane	3,12,5
A1B973/Pden_4001	2	0	1	0	Purine nucleosidase	Cytoplasmic	0
A1B974/Pden_4002	2	1	2	0	Penicillin amidase	Unknown	1,4
A1B975/Pden_4003	1	2	1	2	3-hydroxyisobutyrate dehydrogenase	Unknown	0
A1B987/Pden_4015	5	4	4	5	Linocin_M18 bacteriocin protein	Unknown	11,29,28
A1B989/Pden_4017	17	12	11	13	Amino acid/amide ABC transporter substrate-binding protein, HAAT family	Unknown	0
A1B995/Pden_4023	4	4	5	3	Phosphoglycolate phosphatase	Cytoplasmic	1,4,7,12,14
A1B996/Pden_4024	4	4	4	3	Uncharacterized protein	Unknown	0
A1B997/Pden_4025	18	15	18	15	GTP-binding protein TypA	Membrane	0
A1B998/Pden_4026	1	3	2	0	Uncharacterized protein	Cytoplasmic	0
A1B999/Pden_4027	3	3	2	3	Ferredoxin	Cytoplasmic	0
A1B9A0/Pden_4028	1	3	1	1	Exodeoxyribonuclease 7 large subunit	Cytoplasmic	6,8,4,7,2
A1B9A1/Pden_4029	15	13	11	12	Phosphoribosylamine--glycine ligase	Cytoplasmic	8,1,4,7,12,14
A1B9A2/Pden_4030	17	17	18	17	2-isopropylmalate synthase	Cytoplasmic	8,1,4,7,12,14
A1B9A4/Pden_4032	13	14	17	15	Rod shape-determining protein MreB	Cytoplasmic	3,12,31,17
A1B9A9/Pden_4037	3	3	2	3	D-isomer specific 2-hydroxyacid dehydrogenase, NAD-binding protein	Cytoplasmic	14
A1B9B0/Pden_4038	5	4	4	3	Mannose-6-phosphate isomerase, type 2 / mannose-1-phosphate guanylyltransferase	Cytoplasmic	1,8,7,14

A1B9B3/Pden_4041	1	1	1	2	Arsenate reductase related protein	Unknown	0
A1B9B4/Pden_4042	2	3	2	2	Uncharacterized protein	Cytoplasmic	0
A1B9B5/Pden_4043	1	1	0	2	ErfK/YbiS/YcfS/YnhG family protein	Unknown	0
A1B9B9/Pden_4047	6	6	5	6	NAD transhydrogenase subunit beta	Membrane	0
A1B9C0/Pden_4048	24	23	23	22	NAD transhydrogenase subunit alpha	Membrane	5,13
A1B9C3/Pden_4051	14	10	10	11	Malate synthase G	Cytoplasmic	8,4,7,12,14
A1B9C4/Pden_4052	4	5	4	3	Mucin-associated surface protein	Unknown	0
A1B9C5/Pden_4053	17	15	14	13	Alanyl aminopeptidase, Metallo peptidase, MEROPS family M01	Unknown	0
A1B9C7/Pden_4055	2	1	2	1	Glutamate 5-kinase	Cytoplasmic	8,1,4,7,12,14
A1B9C8/Pden_4056	5	5	7	4	GTPase Obg	Cytoplasmic	20
A1B9D2/Pden_4060	26	25	24	21	Enolase	Cytoplasmic	4,8,7,12,14,2
A1B9D4/Pden_4062	8	6	5	7	2-isopropylmalate synthase	Cytoplasmic	8,1,4,7,12,14
A1B9D5/Pden_4063	4	5	4	3	Cysteine--tRNA ligase	Cytoplasmic	8,6,4,7,12,14
A1B9D7/Pden_4065	3	2	2	3	Uncharacterized protein	Unknown	0
A1B9D8/Pden_4066	3	4	5	5	ABC transporter related protein	Membrane	0
A1B9E3/Pden_4071	4	4	4	5	NADPH-dependent FMN reductase	Cytoplasmic	0
A1B9E4/Pden_4072	22	22	22	21	RNA polymerase sigma factor RpoD	Cytoplasmic	6,1,8,4,7
A1B9E5/Pden_4073	1	2	2	2	DNA primase	Cytoplasmic	0
A1B9E7/Pden_4075	5	4	5	6	L-lactate dehydrogenase	Cytoplasmic	0
A1B9E9/Pden_4077	13	12	15	13	50S ribosomal protein L25	Cytoplasmic	6,1,8,4,7
A1B9F0/Pden_4078	1	2	2	2	Methylenetetrahydrofolate--tRNA--methyltransferase TrmFO -methyltransferase) -methyltransferase)	Cytoplasmic	0
A1B9F5/Pden_4083	3	1	6	6	DNA ligase])	Cytoplasmic	4,8,7,12,14,13
A1B9H0/Pden_4098	1	4	3	5	tRNA-specific 2-thiouridylase MnmA	Cytoplasmic	6,8,4,7
A1B9H1/Pden_4099	1	0	2	2	Cysteine desulfuration protein SufE	Cytoplasmic	0
A1B9H2/Pden_4100	1	1	1	2	Uncharacterized protein	Unknown	0
A1B9H3/Pden_4101	1	2	2	5	GCN5-related N-acetyltransferase	Unknown	0
A1B9H4/Pden_4102	3	4	5	5	50S ribosomal protein L27	Cytoplasmic	6,1,8,4,7
A1B9H5/Pden_4103	9	8	6	8	50S ribosomal protein L21	Unknown	6,1,8,4,7
A1B9I1/Pden_4109	0	2	1	1	Putative sulfonate/nitrate transport system substrate-binding protein	Cytoplasmic	0
A1B9J3/Pden_4123	5	6	5	3	GTPase HflX	Cytoplasmic	0
A1B9J4/Pden_4124	0	4	2	3	RNA-binding protein Hfq	Cytoplasmic	9
A1B9J6/Pden_4126	1	1	2	2	TrkA-N domain protein	Membrane	0
A1B9J7/Pden_4127	12	10	13	8	Two component, sigma54 specific, transcriptional regulator, Fis family	Cytoplasmic	1,6,8,4,7,9
A1B9J8/Pden_4128	4	2	1	1	PAS/PAC sensor signal transduction histidine kinase	Membrane	9
A1B9J9/Pden_4129	17	20	20	19	Two component, sigma54 specific, transcriptional regulator, Fis family	Cytoplasmic	1,6,8,4,7,9
A1B9K0/Pden_4130	2	4	3	4	Signal transduction histidine kinase, nitrogen specific, NtrB	Membrane	0
A1B9K3/Pden_4133	2	3	4	3	FHA domain containing protein	Unknown	0
A1B9K4/Pden_4134	14	15	15	15	Uncharacterized protein	OuterMembrane	0
A1B9K5/Pden_4135	10	10	8	11	Uncharacterized protein	Unknown	0
A1B9K9/Pden_4139	1	0	2	0	Putative ferric uptake regulator, Fur family	Cytoplasmic	0
A1B9L0/Pden_4140	3	3	3	5	Periplasmic solute binding protein	Periplasmic	5,13
A1B9L2/Pden_4142	1	2	0	2	Pseudouridine synthase	Cytoplasmic	6,8,4,7
A1B9L4/Pden_4144	35	34	35	33	Pyruvate carboxylase	Cytoplasmic	8,1,4,7,12,14
A1B9N9/Pden_4169	3	1	2	1	Putative sulfonate/nitrate transport system substrate-binding protein	Unknown	0

A1B9P1/Pden_4171	0	2	1	1	Uncharacterized protein	Unknown	0
A1B9P3/Pden_4173	3	4	3	6	TonB-dependent receptor	OuterMembrane	5
A1B9Q2/Pden_4182	2	1	1	1	NADPH-dependent 7-cyano-7-deazaguanine reductase reductase)	Cytoplasmic	8,1,4,7,12,14
A1B9R0/Pden_4190	6	6	4	7	Carbohydrate ABC transporter substrate-binding protein, CUT1 family	Periplasmic	0
A1B9S2/Pden_4202	2	2	2	2	Hemin-degrading family protein	Cytoplasmic	5,13
A1B9S9/Pden_4209	4	3	1	2	Phosphate butyryltransferase	Cytoplasmic	0
A1B9T1/Pden_4211	2	3	2	1	SSS sodium solute transporter superfamily	Membrane	12,5,13
A1B9T2/Pden_4212	1	1	2	1	Uncharacterized protein	Membrane	0
A1B9T3/Pden_4213	14	16	15	14	Acetyl-coenzyme A synthetase	Cytoplasmic	1,4,7,12,14
A1B9T4/Pden_4214	1	2	0	0	FAD:protein FMN transferase	Unknown	8,4,7
A1B9T5/Pden_4215	1	0	1	2	NosL family protein	Unknown	0
A1B9T7/Pden_4217	7	9	7	8	ABC transporter related protein	Cytoplasmic	0
A1B9T8/Pden_4218	6	8	6	7	Periplasmic copper-binding protein	Periplasmic	0
A1B9T9/Pden_4219	44	44	39	44	Nitrous-oxide reductase OR)	Periplasmic	0
A1B9U0/Pden_4220	8	8	9	10	FMN-binding domain protein	Membrane	9
Q71RW5/Pden_4222	8	8	8	12	Pseudoazurin	Periplasmic	0
A1B9U4/Pden_4224	8	8	10	8	Uncharacterized protein	Cytoplasmic	0
A1B9U5/Pden_4225	2	2	2	2	Flavin prenyltransferase UbiX	Membrane	1,4
A1B9U6/Pden_4226	8	11	11	8	3-octaprenyl-4hydroxybenzoate decarboxylase	Cytoplasmic	0
A1B9U7/Pden_4227	3	3	2	1	Uncharacterized protein	Membrane	0
A1B9U8/Pden_4228	14	12	12	10	Peptidase U32	Cytoplasmic	0
A1B9U9/Pden_4229	11	9	9	7	Peptidase U32	Unknown	0
A1B9V1/Pden_4231	1	2	2	1	Uncharacterized protein	Unknown	0
A1B9V2/Pden_4232	5	7	9	9	Peptidylprolyl isomerase	Cytoplasmic	0
A1B9V3/Pden_4233	2	1	2	2	Respiratory nitrate reductase gamma subunit	Membrane	0
A1B9V4/Pden_4234	4	4	4	5	Respiratory nitrate reductase chaperone NarJ	Cytoplasmic	3
A1B9V5/Pden_4235	43	40	43	44	Respiratory nitrate reductase beta subunit	Membrane	6,4,7,12,14
A1B9V6/Pden_4236	83	84	80	84	Respiratory nitrate reductase alpha subunit apoprotein	Membrane	6,4,7,12,14
A1B9V7/Pden_4237	4	4	3	2	Nitrite transporter	Membrane	0
A1B9W4/Pden_4244	6	5	5	3	Asparaginase	Unknown	8,6,4,7,12,14
A1B9X6/Pden_4256	2	3	1	1	Transcriptional regulator, LysR family	Cytoplasmic	6,1,8,4,7
A1B9X7/Pden_4257	1	1	1	3	Molybdopterin dehydrogenase, FAD-binding protein	Cytoplasmic	0
A1B9X8/Pden_4258	1	2	2	3	Xanthine dehydrogenase, molybdenum binding subunit apoprotein / Xanthine oxidase	Cytoplasmic	0
A1B9Z3/Pden_4273	6	5	6	6	Aminotransferase	Cytoplasmic	0
A1BA00/Pden_4280	3	4	1	3	Transcriptional regulator, IclR family	Cytoplasmic	1,6,8,4,7,9
A1BA12/Pden_4292	5	6	5	5	50S ribosomal protein L33	Cytoplasmic	6,1,8,4,7
A1BA15/Pden_4295	3	3	3	3	DNA helicase	Cytoplasmic	0
A1BA17/Pden_4297	3	0	3	1	GTP cyclohydrolase FolE2	Cytoplasmic	6,1,4,7
A1BA18/Pden_4298	17	15	13	15	O-succinylhomoserine sulfhydrylase	Cytoplasmic	8,1,4,7,12,14
A1BA21/Pden_4301	30	37	29	37	Aconitate hydratase	Cytoplasmic	0
A1BA23/Pden_4303	27	28	26	30	30S ribosomal protein S4	Cytoplasmic	6,1,8,4,7
A1BA25/Pden_4305	10	10	9	10	Triosephosphate isomerase	Cytoplasmic	4,8,7,12,14,2
A1BA26/Pden_4306	3	3	2	2	Methionine import ATP-binding protein MetN	Membrane	5,13
A1BA28/Pden_4308	6	4	6	6	Lipoprotein, YaeC family	Membrane	0
A1BA29/Pden_4309	2	2	3	0	Acetyl-CoA hydrolase	Cytoplasmic	4,7

A1BA32/Pden_4312	4	2	3	3	FAD linked oxidase domain protein	Cytoplasmic	0
A1BA34/Pden_4314	6	6	4	5	Peptidase M16 domain protein	Cytoplasmic	0
A1BA35/Pden_4315	17	15	16	13	L-threonine synthase	Cytoplasmic	0
A1BA38/Pden_4318	1	0	0	2	Cytochrome c oxidase assembly protein CtaG	Unknown	3
A1BA41/Pden_4321	5	5	4	4	Cytochrome c oxidase subunit 2	Membrane	4,12,14
A1BA42/Pden_4322	9	8	12	8	Uncharacterized protein	Cytoplasmic	0
A1BA47/Pden_4327	1	2	2	1	Phosphate import ATP-binding protein PstB	Membrane	0
A1BA50/Pden_4330	3	3	4	3	Phosphate ABC transporter substrate-binding protein, PhoT family	Membrane	0
A1BA52/Pden_4332	7	6	7	11	Chorismate synthase	Cytoplasmic	8,1,4,7,12,14
A1BA61/Pden_4341	6	5	3	4	Sulfate-transporting ATPase	Membrane	0
A1BA64/Pden_4344	15	14	13	14	Sulfate ABC transporter, periplasmic sulfate-binding protein	Periplasmic	0
A1BA70/Pden_4350	6	5	6	2	Periplasmic glucan biosynthesis protein, MdoG	Periplasmic	1,8,7,14
A1BA86/Pden_4366	13	15	12	14	DEAD/DEAH box helicase domain protein	Cytoplasmic	0
A1BA93/Pden_4373	2	1	2	2	TonB-dependent siderophore receptor	OuterMembrane	5,13
A1BA97/Pden_4377	10	9	11	9	Ribonucleoside-diphosphate reductase	Cytoplasmic	6,1,8,4,7
A1BA98/Pden_4378	6	4	5	5	Ribonucleoside-diphosphate reductase subunit beta	Cytoplasmic	8,1,4,7,12,14
A1BAA0/Pden_4380	1	2	2	2	Putative transcriptional regulator, GntR family	Cytoplasmic	1
A1BAA2/Pden_4382	6	8	7	7	TonB-dependent receptor	OuterMembrane	5
A1BAA7/Pden_4387	4	6	9	5	Carbohydrate ABC transporter substrate-binding protein, CUT1 family	Periplasmic	0
A1BAB5/Pden_4395	13	13	9	11	Sulfate adenylyltransferase subunit 2	Cytoplasmic	1,4,12,14
A1BAB6/Pden_4396	22	24	24	22	Adenylyl-sulfate kinase	Cytoplasmic	4
A1BAC1/Pden_4401	1	0	3	3	Uncharacterized protein UPF0065	Unknown	0
A1BAC4/Pden_4404	5	5	4	7	Uncharacterized protein UPF0065	Unknown	0
A1BAD2/Pden_4412	2	2	2	2	Acyl-CoA dehydrogenase domain protein	Cytoplasmic	0
A1BAD6/Pden_4416	9	10	6	6	Succinate semialdehyde dehydrogenase	Cytoplasmic	0
A1BAD7/Pden_4417	8	7	8	6	Elongation factor 4	Membrane	9
A1BAE1/Pden_4421	5	4	4	5	Phosphonate metabolism protein PhnM	Cytoplasmic	4,7,2
A1BAE3/Pden_4423	16	13	16	17	Phosphoglucomutase/phosphomannomutase alpha/beta/alpha domain I	Cytoplasmic	8,7
A1BAE6/Pden_4426	0	0	2	1	Glycogen synthase	Cytoplasmic	8,1,4,7,12,14
A1BAE8/Pden_4428	2	1	2	1	1,4-alpha-glucan branching enzyme GlgB -glucan branching enzyme)	Cytoplasmic	8,1,4,7,12,14
A1BAE9/Pden_4429	9	7	8	10	Alpha-1,4 glucan phosphorylase	Cytoplasmic	8,7
A1BAF0/Pden_4430	3	2	2	2	Nicotinate-nucleotide pyrophosphorylase	Cytoplasmic	8,1,4,7,12,14
A1BAF1/Pden_4431	3	2	3	1	L-aspartate oxidase	Cytoplasmic	0
A1BAF2/Pden_4432	4	4	4	2	Quinolinate synthase A	Cytoplasmic	8,1,4,7,12,14
A1BAF3/Pden_4433	2	2	1	2	Transcriptional regulator, IclR family	Cytoplasmic	1,6,8,4,7,9
A1BAG3/Pden_4443	3	5	4	4	Electron transport protein SCO1/SenC	Unknown	0
A1BAG4/Pden_4444	10	14	10	11	Uncharacterized protein	Unknown	0
A1BAG7/Pden_4447	16	13	15	17	Extracellular solute-binding protein, family 1	Periplasmic	0
A1BAH2/Pden_4452	15	3	2	3	Assimilatory nitrite reductase H) large subunit	Cytoplasmic	6,4,7,12,14
A1BAH8/Pden_4458	10	7	10	6	RNA binding S1 domain protein	Cytoplasmic	6,8,4,7
A1BAI1/Pden_4461	5	6	5	6	Nitrogen regulatory protein P-II	Membrane	1,6,8,4,7,9
A1BAI2/Pden_4462	33	33	28	28	Glutamine synthetase	Cytoplasmic	8,1,4,7,12,14
A1BAI3/Pden_4463	15	18	16	17	Adenylosuccinate lyase	Cytoplasmic	8,1,4,7,12,14
A1BAI5/Pden_4465	38	35	33	35	Glyceraldehyde-3-phosphate dehydrogenase	Cytoplasmic	8,7,14
A1BAI7/Pden_4467	6	5	8	5	Dihydroorotase	Cytoplasmic	8,1,4,7,12,14

A1BAI8/Pden_4468	8	9	10	8	Orotate phosphoribosyltransferase	Cytoplasmic	8,1,4,7,12,14
A1BAI9/Pden_4469	3	5	6	4	Replicative DNA helicase	Cytoplasmic	6,1,8,4,7
A1BAJ4/Pden_4474	8	8	10	8	Uncharacterized protein	Cytoplasmic	0
A1BAJ6/Pden_4476	3	5	7	5	Phosphopantetheine adenylyltransferase	Cytoplasmic	8,1,4,7,12,14
A1BAJ7/Pden_4477	8	8	8	8	Putative signal-transduction protein with CBS domains	Cytoplasmic	0
A1BAJ9/Pden_4479	16	20	19	16	Methylmalonate-semialdehyde dehydrogenase	Cytoplasmic	0
A1BAK1/Pden_4481	2	1	1	1	Enoyl-CoA hydratase/isomerase	Cytoplasmic	0
A1BAK2/Pden_4482	3	2	2	2	3-hydroxyisobutyrate dehydrogenase	Cytoplasmic	4,8,7,12,14,2
A1BAK4/Pden_4484	4	4	4	3	DNA repair protein RecN	Cytoplasmic	4,8,7,12,14,13
A1BAK5/Pden_4485	8	9	7	7	Outer membrane protein assembly factor BamD	Unknown	3,12,5
A1BAK7/Pden_4487	23	22	21	23	Cell division protein FtsZ	Cytoplasmic	3,12,30
A1BAL0/Pden_4490	10	6	5	8	D-alanine--D-alanine ligase	Cytoplasmic	12,7,14,9,10,19
A1BAL1/Pden_4491	3	3	2	3	UDP-N-acetylenolpyruvoylglucosamine reductase	Cytoplasmic	12,7,14,9,10,19
A1BAL4/Pden_4494	7	7	7	7	UDP-N-acetylmuramate--L-alanine ligase	Cytoplasmic	12,7,14,9,10,19
A1BAL5/Pden_4495	7	6	5	5	UDP-N-acetylglucosamine--N-acetylmuramyl-pyrophosphoryl-undecaprenol N-acetylglucosamine transferase	Membrane	12,7,14,9,10,19
A1BAL9/Pden_4499	2	3	6	3	Homoserine O-succinyltransferase	Cytoplasmic	8,1,4,7,12,14
A1BAM2/Pden_4502	8	6	6	5	ABC polyamine transporter, periplasmic substrate-binding protein	Periplasmic	5,13
A1BAM3/Pden_4503	9	12	9	11	Gamma-glutamyl phosphate reductase	Cytoplasmic	8,1,4,7,12,14
A1BAM6/Pden_4506	1	2	2	2	Ornithine-acyl[acyl carrier protein] N-acyltransferase	Cytoplasmic	0
A1BAM9/Pden_4509	3	3	3	6	Uncharacterized protein	Cytoplasmic	0
A1BAN0/Pden_4510	6	4	7	3	Probable protein kinase UbiB	Membrane	1,4,7,12,14
A1BAN1/Pden_4511	0	1	1	2	Ubiquinone/menaquinone biosynthesis C-methyltransferase UbiE	Cytoplasmic	1,4,7,12,14
A1BAN2/Pden_4512	1	1	3	2	Formamidopyrimidine-DNA glycosylase lyase MutM)	Cytoplasmic	4,8,7,12,14,13
A1BAN3/Pden_4513	6	5	5	4	Short chain enoyl-CoA hydratase	Cytoplasmic	0
A1BAN4/Pden_4514	2	3	4	3	30S ribosomal protein S20	Cytoplasmic	6,1,8,4,7
A1BAN5/Pden_4515	2	1	2	2	3-demethylubiquinone-9 3-methyltransferase	Unknown	0
A1BAP1/Pden_4521	11	7	6	6	Uncharacterized protein	Unknown	0
A1BAR5/Pden_4546	3	2	3	3	TRAP dicarboxylate transporter-DctP subunit	Unknown	5
A1BAR7/Pden_4548	3	0	0	1	Aldehyde dehydrogenase	Cytoplasmic	0
A1BAR8/Pden_4549	3	3	4	5	Iron-containing alcohol dehydrogenase	Cytoplasmic	0
A1BAR9/Pden_4550	14	16	15	14	Acetyl-coenzyme A synthetase	Cytoplasmic	1,4,7,12,14
A1BAT6/Pden_4567	44	43	41	38	Aconitase	Cytoplasmic	8,4,7,12,14
A1BAV5/Pden_4586	9	10	10	10	10 kDa chaperonin	Cytoplasmic	25
A1BAW1/Pden_4592	0	1	2	3	Alkyl hydroperoxide reductase AhpD	Unknown	0
A1BAW3/Pden_4594	1	1	0	2	Thiamine pyrophosphate enzyme domain protein TPP-binding protein	Cytoplasmic	0
A1BAY8/Pden_4619	2	1	1	0	TRAP dicarboxylate transporter-DctP subunit	Periplasmic	5
A1BB09/Pden_4642	3	3	3	2	Uncharacterized protein	Unknown	0
A1BB12/Pden_4645	1	2	1	2	Transcriptional regulator, TetR family	Cytoplasmic	1,6,8,4,7,9
A1BB21/Pden_4654	1	1	1	2	HAD superfamily phosphatase, TIGR01672	Unknown	0
A1BB25/Pden_4658	7	7	5	4	Extracellular solute-binding protein, family 1	Unknown	0
A1BB39/Pden_4672	1	0	2	0	Transcriptional regulator, GntR family	Cytoplasmic	6,1,8,4,7
A1BB40/Pden_4673	20	18	21	19	Band 7 protein	Membrane	0
A1BB46/Pden_4679	1	0	2	2	6,7-dimethyl-8-ribityllumazine synthase	Cytoplasmic	1,6,4,7,12,14
A1BB50/Pden_4683	9	9	8	9	3-oxoacid CoA-transferase, A subunit	Cytoplasmic	0

A1BB51/Pden_4684	6	5	5	4	Butyryl-CoA:acetate CoA transferase	Cytoplasmic	0
A1BB52/Pden_4685	0	2	3	1	Secretion protein HlyD family protein	Membrane	0
A1BB57/Pden_4690	1	1	1	2	Phosphatidyl-N-methylethanolamine N-methyltransferase	Cytoplasmic	0
A1BB59/Pden_4692	2	1	3	2	Phosphatidylserine decarboxylase proenzyme [Cleaved into: Phosphatidylserine decarboxylase beta chain; Phosphatidylserine decarboxylase alpha chain]	Cytoplasmic	8,1,4,7,12,14
A1BB73/Pden_4706	2	0	2	0	Beta-lactamase domain protein	Cytoplasmic	0
A1BB75/Pden_4708	3	1	3	1	Glyoxalase/bleomycin resistance protein/dioxygenase	Cytoplasmic	0
A1BB77/Pden_4710	2	2	3	3	Glucose-methanol-choline oxidoreductase	Cytoplasmic	0
A1BB78/Pden_4711	0	4	6	4	Cell division topological specificity factor	Cytoplasmic	12,9
A1BB79/Pden_4712	15	16	15	15	Site-determining protein	Cytoplasmic	12
A1BB80/Pden_4713	5	7	7	6	Probable septum site-determining protein MinC	Cytoplasmic	3,12,9,31,17
A1BB82/Pden_4715	12	11	12	12	TRAP dicarboxylate transporter, DctP subunit	Periplasmic	5
A1BB88/Pden_4721	0	2	3	1	Periplasmic nitrate reductase	Periplasmic	8,1,4,7,12,14
A1BB94/Pden_4727	2	1	1	0	Glucose sorbosone dehydrogenase	Unknown	8,7
A1BBA6/Pden_4739	1	0	1	2	Alkylphosphonate utilization operon protein PhnA	Unknown	0
A1BBB0/Pden_4743	2	1	0	1	Peptidoglycan-binding domain 1 protein	Unknown	0
A1BBB1/Pden_4744	3	2	1	2	Uncharacterized protein	Unknown	0
A1BBB2/Pden_4745	11	14	10	14	Sel1 domain protein repeat-containing protein	Unknown	0
A1BBC2/Pden_4755	1	1	2	0	Putative carbohydrate binding protein	Membrane	8,7
A1BBC6/Pden_4759	1	2	3	1	Dihydrolipoamide acetyltransferase component of pyruvate dehydrogenase complex	Cytoplasmic	0
A1BBC7/Pden_4760	1	0	2	1	Dihydrolipoyl dehydrogenase	Cytoplasmic	12,7,14,9,2,10
A1BBD2/Pden_4765	2	4	4	5	C4-dicarboxylate transport protein	Membrane	5,13
A1BBF5/Pden_4788	3	4	4	2	Uncharacterized protein	Unknown	0
A1BBG9/Pden_4802	0	3	2	3	Transcriptional regulator, TetR family	Cytoplasmic	1,6,8,4,7,9
A1BBH9/Pden_4812	2	1	3	1	Transcriptional regulator, ArsR family	Cytoplasmic	0
A1BBI4/Pden_4817	7	5	6	7	TRAP dicarboxylate transporter, DctP subunit	Periplasmic	5
A1BBI6/Pden_4819	0	3	1	0	Thiolase	Unknown	0
A1BBJ1/Pden_4824	3	2	3	2	Phosphoglucomutase/phosphomannomutase alpha/beta/alpha domain I	Cytoplasmic	8,7
A1BBL2/Pden_4846	2	1	1	3	Periplasmic binding protein/LacI transcriptional regulator	Cytoplasmic	6,1,8,4,7
A1BBN1/Pden_4865	3	0	2	4	Aldehyde dehydrogenase	Cytoplasmic	0
A1BBN3/Pden_4867	2	2	2	1	Dihydrodipicolinate synthase	Cytoplasmic	0
A1BBN4/Pden_4868	2	2	0	1	Transcriptional regulator, GntR family	Cytoplasmic	6,1,8,4,7
A1BBQ4/Pden_4888	1	3	3	2	Substrate-binding region of ABC-type glycine betaine transport system	Membrane	0
A1BBS1/Pden_4905	6	10	4	10	Bifunctional protein FolD 2 [Includes: Methylenetetrahydrofolate dehydrogenase ; Methenyltetrahydrofolate cyclohydrolase]	Cytoplasmic	8,1,4,7,12,14
A1BBT9/Pden_4923	0	1	2	0	Transcriptional regulator, IclR family	Cytoplasmic	1,6,8,4,7,9
A1BC00/Pden_4984	1	2	2	1	Transketolase, central region	Cytoplasmic	0
A1BC11/Pden_4995	0	2	1	2	Transcriptional regulator, GntR family	Cytoplasmic	6,1,8,4,7
A1BC33/Pden_5017	2	2	3	2	Uncharacterized protein	Unknown	0
A1BC39/Pden_5023	5	6	3	4	NAD-dependent epimerase/dehydratase	Cytoplasmic	8,7
A1BC41/Pden_5025	1	1	4	5	Glycerol-3-phosphate cytidylyltransferase	Cytoplasmic	1,4,7,12,14
A1BC43/Pden_5027	7	9	11	11	CDP-glycerol:poly glycerophosphotransferase	Membrane	0
A1BC44/Pden_5028	5	4	4	4	Uncharacterized protein	Cytoplasmic	0
A1BC45/Pden_5029	1	1	2	1	Uncharacterized protein	Cytoplasmic	0
A1BC46/Pden_5030	3	1	2	1	Uncharacterized protein	Cytoplasmic	0

A1BC51/Pden_5035	2	0	0	2	Cobyrinic acid a,c-diamide synthase	Cytoplasmic	0
A1BC52/Pden_5036	5	5	6	4	ParB-like partition protein	Cytoplasmic	0
A1BC54/Pden_5038	9	9	6	9	Capsule polysaccharide export protein-like protein	Membrane	0
A1BC82/Pden_5066	3	2	3	1	TRAP dicarboxylate transporter, DctP subunit	Periplasmic	5
A1BC85/Pden_5069	6	5	3	5	Cystathionine gamma-synthase	Cytoplasmic	0
A1BCB1/Pden_5095	9	9	14	11	Amino acid/amide ABC transporter substrate-binding protein, HAAT family	Periplasmic	0
A1BCB9/Pden_5103	2	1	1	1	Poly-beta-hydroxybutyrate polymerase domain protein	Cytoplasmic	1,7
A1BCE1/Pden_5125	4	4	7	4	Putative monooxygenase protein	Cytoplasmic	0
A1BCE3/Pden_5127	2	2	3	1	Transcriptional regulator, Fis family	Unknown	0
A1BCF0/Pden_5134	4	5	4	3	Uncharacterized protein	Unknown	0

[1]Protein annotated from UniProt (UP000000361). [2]Genes annotated from GeneBank (T00440). [3]Subcellular location according with PSOTb v3.0.2. [4]GOi (Gene Ontology respect to biological process at the third level), numbers refer to 0: unknown, 1: biosynthetic process, 2: catabolic process, 3: cellular component organization, 4: cellular metabolic process, 5: establishment of localization, 6: nitrogen compound metabolic process, 7: organic substance metabolic process, 8: primary metabolic process, 9: regulation of biological process, 10: regulation of biological quality, 11: response to stress, 12: single-organism cellular process, 13: single-organism localization, 14: single-organism metabolic process, 15: carbon utilization, 16: anatomical structure formation involved in morphogenesis, 17: anatomical structure morphogenesis, 18: cell adhesion, 19: cell wall organization or biogenesis, 20: cellular component biogenesis, 21: cellular detoxification, 22: cellular localization, 23: macromolecule localization, 24: methylation, 25: protein folding, 26: protein unfolding, 27: response to abiotic stimulus, 28: response to biotic stimulus, 29: response to external stimulus, 30: single organism reproductive process, 31: single-organism developmental process and 32: response to chemicals.
