# Supplementary material for: Exploring the Denitrification Proteome of Paracoccus denitrificans PD1222
Source: Front Microbiol. 2018 May 29;9:1137. doi: 10.3389/fmicb.2018.01137 (PMC5987163; doi:10.3389/fmicb.2018.01137)
Supplement: Supplementary file 3 [file Table_3.PDF]

Table S3. *P. denitrificans* PD1222 proteins identified from cells grown aerobically with nitrate.

Protein[1]/Gene[2]	R1	R2	R3	R4	Protein names	Subcellular location[3]	GO[4]
A1AXX5/Pden_0002	20	19	18	21	Transcription termination factor Rho	Cytoplasmic	1,6,8,4,7,9
A1AXX7/Pden_0004	1	0	0	2	tRNA uridine 5-carboxymethylaminomethyl modification enzyme MnmG	Cytoplasmic	6,8,4,7
A1AXX9/Pden_0006	2	1	1	3	Cobyrinic acid a,c-diamide synthase	Membrane	0
A1AXY0/Pden_0007	8	8	3	7	ParB-like partition protein	Cytoplasmic	0
A1AXY1/Pden_0008	0	1	2	0	Protein GrpE	Cytoplasmic	25
A1AXY3/Pden_0010	5	5	7	5	Ribonuclease PH	Cytoplasmic	6,8,4,7
A1AXY4/Pden_0011	3	1	2	3	Non-canonical purine NTP pyrophosphatase	Cytoplasmic	4,8,7,12,14,2
A1AXY9/Pden_0016	8	6	6	6	S-glutathione dehydrogenase	Cytoplasmic	7,14
A1AXZ0/Pden_0017	9	8	6	9	ATP-dependent Clp protease proteolytic subunit	Cytoplasmic	0
A1AXZ2/Pden_0019	7	6	9	9	S-formylglutathione hydrolase	Unknown	4,7,12,14,2
A1AY07/Pden_0034	4	4	4	4	ABC transporter related protein	Membrane	0
A1AY08/Pden_0035	14	8	6	6	Aminopyrimidine aminohydrolase	Unknown	6,4,7,12,14
A1AY09/Pden_0036	11	8	8	12	NlpA lipoprotein	Unknown	1,6,4,7,12,14
A1AY10/Pden_0037	7	6	5	5	UBA/THIF-type NAD/FAD binding protein	Membrane	0
A1AY11/Pden_0038	6	6	6	5	Thiamine-phosphate diphosphorylase	Cytoplasmic	1,6,4,7,12,14
A1AY12/Pden_0039	11	11	12	12	Thiazole synthase	Cytoplasmic	6,1,4,7
A1AY14/Pden_0041	22	19	20	19	Glycine oxidase	Cytoplasmic	0
A1AY15/Pden_0042	11	8	6	7	Hydroxymethylpyrimidine kinase / phosphomethylpyrimidine kinase	Unknown	1,6,4,7,12,14
A1AY16/Pden_0043	2	1	1	1	HAD-superfamily hydrolase, subfamily IA, variant 3	Unknown	0
A1AY25/Pden_0052	2	3	0	3	Amino acid/amide ABC transporter substrate-binding protein, HAAT family	Unknown	0
A1AY28/Pden_0055	1	1	2	0	50S ribosomal protein L36	Unknown	6,1,8,4,7
A1AY30/Pden_0057	8	8	8	6	SPFH domain, Band 7 family protein	Cytoplasmic	0
A1AY32/Pden_0059	3	2	1	2	Glutathione S-transferase, N-terminal domain	Membrane	0
A1AY33/Pden_0060	5	5	4	3	Orotidine 5'-phosphate decarboxylase	Cytoplasmic	8,1,4,7,12,14
A1AY35/Pden_0062	3	8	5	6	Chaperone protein ClpB	Cytoplasmic	11,8,7,27
A1AY45/Pden_0072	2	2	1	1	CHAD domain containing protein	Cytoplasmic	0
A1AY46/Pden_0073	6	3	7	6	Cobyrinic acid a,c-diamide synthase	Membrane	0
A1AY60/Pden_0087	6	5	4	5	Acylneuraminate cytidylyltransferase	Cytoplasmic	0
A1AY61/Pden_0088	3	3	3	5	Flagellin modification protein FlmD	Cytoplasmic	0
A1AY62/Pden_0089	16	16	17	15	Uncharacterized protein	Cytoplasmic	0
A1AY63/Pden_0090	23	23	22	23	Uncharacterized protein	Cytoplasmic	1
A1AY66/Pden_0093	29	28	27	25	Polysaccharide biosynthesis protein CapD	Cytoplasmic	0
A1AY85/Pden_0112	3	3	2	3	Uncharacterized protein	Cytoplasmic	0
A1AYG9/Pden_0197	2	2	1	1	3-hydroxyacyl-CoA dehydrogenase	Unknown	4,8,7,12,14,2
A1AYH6/Pden_0206	1	0	0	2	Short-chain dehydrogenase/reductase SDR	Cytoplasmic	0
A1AYI0/Pden_0210	1	0	1	2	Uncharacterized protein	Unknown	0
A1AYM0/Pden_0250	4	1	2	1	Transcriptional regulator, RpiR family	Cytoplasmic	8,7
A1AYM5/Pden_0255	5	6	7	4	Uncharacterized protein	Cytoplasmic	0
A1AYP7/Pden_0277	0	0	0	2	Gamma-glutamylcyclotransferase	Unknown	0
A1AYQ1/Pden_0281	2	3	4	1	Hydroxyectoine-binding protein / ectoine-binding protein	Periplasmic	0
A1AYS0/Pden_0300	0	0	0	2	Periplasmic binding protein	Unknown	0
A1AYS8/Pden_0308	4	4	3	2	Uncharacterized protein UPF0065	Periplasmic	0

A1AYT5/Pden_0315	1	2	0	1	Oxidoreductase domain protein	Unknown	0
A1AYU0/Pden_0320	2	1	2	2	UPF0271 protein Pden_0320	Unknown	8,7
A1AYU3/Pden_0323	5	4	6	5	Biotin carboxyl carrier protein / biotin carboxylase	Cytoplasmic	0
A1AYU5/Pden_0325	4	3	1	4	Uncharacterized protein	Unknown	0
A1AYV0/Pden_0330	4	2	3	3	NADPH-dependent FMN reductase	Cytoplasmic	0
A1AYV3/Pden_0333	6	4	7	6	Tryptophan synthase alpha chain	Cytoplasmic	0
A1AYV4/Pden_0334	10	13	12	12	Ribosome-binding ATPase YchF	Unknown	0
A1AZ13/Pden_0393	1	3	0	0	Uncharacterized protein	Unknown	0
A1AZ15/Pden_0395	2	2	3	2	Diaminopimelate epimerase	Cytoplasmic	8,1,4,7,12,14
A1AZ17/Pden_0397	3	0	2	0	Histone deacetylase superfamily	Cytoplasmic	0
A1AZ19/Pden_0399	4	4	3	4	Farnesyl-diphosphate synthase	Cytoplasmic	8,1,4,7,12,14
A1AZ20/Pden_0400	6	3	3	6	1-deoxy-D-xylulose-5-phosphate synthase	Cytoplasmic	8,1,4,7,12,14
A1AZ21/Pden_0401	3	0	2	1	SH3, type 3 domain protein	Membrane	0
A1AZ24/Pden_0404	0	0	2	1	AMP nucleosidase	Cytoplasmic	8,1,4,7,12,14
A1AZ25/Pden_0405	7	6	6	6	Histone family protein DNA-binding protein	Cytoplasmic	0
A1AZ36/Pden_0416	14	17	14	13	DNA gyrase subunit B	Cytoplasmic	1,6,8,3,4,7
A1AZ38/Pden_0418	5	6	3	2	GTPase Era	Membrane	20
A1AZ39/Pden_0419	3	3	2	4	Ribonuclease 3	Cytoplasmic	6,8,4,7,2
A1AZ40/Pden_0420	5	4	3	3	Signal peptidase I	Membrane	0
A1AZ42/Pden_0422	6	3	5	4	Pyridoxine 5'-phosphate synthase	Cytoplasmic	1,6,4,7,12,14
A1AZ44/Pden_0424	7	5	3	1	Tetratricopeptide TPR_2 repeat protein	Unknown	0
A1AZ45/Pden_0425	15	14	9	10	Electron-transferring-flavoprotein dehydrogenase	Unknown	0
A1AZ46/Pden_0426	8	8	10	6	Transcription elongation factor GreA	Cytoplasmic	1,6,8,4,7,9
A1AZ51/Pden_0431	9	6	8	10	Uncharacterized protein	Unknown	0
A1AZ54/Pden_0434	10	10	9	9	Acyl-CoA dehydrogenase domain protein	Cytoplasmic	0
A1AZ55/Pden_0435	1	0	2	2	Threonylcarbamoyl-AMP synthase	Unknown	6,8,4,7
A1AZ60/Pden_0440	19	15	12	12	3-isopropylmalate dehydrogenase	Cytoplasmic	8,1,4,7,12,14
A1AZ63/Pden_0443	1	2	1	0	Uncharacterized protein	Cytoplasmic	0
A1AZ64/Pden_0444	3	3	3	3	Uncharacterized protein	Cytoplasmic	0
A1AZA6/Pden_0488	56	54	49	41	Glutamate synthase large subunit	Unknown	8,1,4,7,12,14
A1AZA8/Pden_0490	21	22	19	19	Glutamate synthase small subunit	Cytoplasmic	8,1,4,7,12,14
A1AZB0/Pden_0492	11	8	4	7	NAD-dependent epimerase/dehydratase	Cytoplasmic	0
A1AZB5/Pden_0497	10	5	4	6	L-threonine dehydratase	Cytoplasmic	8,1,4,7,12,14
A1AZB7/Pden_0499	27	28	23	26	Argininosuccinate synthase	Cytoplasmic	8,1,4,7,12,14
A1AZC5/Pden_0507	4	5	1	2	Chaperone DnaJ domain protein	Cytoplasmic	25
A1AZC8/Pden_0510	2	2	1	2	Putative pre-16S rRNA nuclease	Cytoplasmic	6,8,4,7
A1AZC9/Pden_0511	2	2	2	1	Putative cytochrome c-type biogenesis protein, CycH	Unknown	0
A1AZD0/Pden_0512	2	2	0	0	Sarcosine oxidase, beta subunit family	Cytoplasmic	8,6,4,7,12,14
A1AZD3/Pden_0515	9	6	6	4	Sarcosine oxidase, alpha subunit family	Cytoplasmic	8,6,4,7,12,14
A1AZD4/Pden_0516	2	1	1	0	Sarcosine oxidase, gamma subunit	Unknown	0
A1AZD5/Pden_0517	1	3	0	1	Uncharacterized protein	Unknown	0
A1AZD6/Pden_0518	13	10	9	9	Superoxide dismutase	Periplasmic	0
A1AZE0/Pden_0522	7	6	4	6	ErfK/YbiS/YcfS/YnhG family protein	Unknown	0
A1AZE6/Pden_0528	6	6	7	5	Nucleoside-binding protein	Unknown	0
A1AZE9/Pden_0531	3	4	4	4	Nitrogen-fixing NifU domain protein	Unknown	1,3
A1AZF3/Pden_0535	2	2	0	2	Uncharacterized protein	Membrane	0

A1AZF5/Pden_0537	1	3	2	2	Peptidoglycan-binding domain 1 protein	Cytoplasmic	0
A1AZF6/Pden_0538	5	3	1	1	Glycine--tRNA ligase alpha subunit	Cytoplasmic	8,6,4,7,12,14
A1AZF7/Pden_0539	5	4	5	2	Signal transduction histidine kinase regulating citrate/malate metabolism	Unknown	0
A1AZF8/Pden_0540	17	16	13	11	Glycine--tRNA ligase beta subunit	Cytoplasmic	8,6,4,7,12,14
A1AZF9/Pden_0541	3	4	2	2	Pyruvate phosphate dikinase	Cytoplasmic	4,7,12,14
A1AZG1/Pden_0543	2	1	0	0	Dihydroneopterin aldolase	Cytoplasmic	8,6,4,7,12,14
A1AZG3/Pden_0545	7	7	8	6	Phosphoglucosamine mutase	Cytoplasmic	8,7
A1AZG5/Pden_0547	3	4	1	2	RNA polymerase-binding transcription factor DksA	Cytoplasmic	9
A1AZG6/Pden_0548	5	3	3	1	ATPase associated with various cellular activities, AAA_5	Cytoplasmic	0
A1AZG9/Pden_0551	29	25	20	17	Dihydrolipoyl dehydrogenase	Cytoplasmic	12,7,14,9,2,10
A1AZH2/Pden_0554	24	24	23	21	Dihydrolipoyllysine-residue succinyltransferase component of 2-oxoglutarate dehydrogenase complex	Cytoplasmic	4,8,7,12,14,2
A1AZH3/Pden_0555	41	42	38	35	2-oxoglutarate dehydrogenase E1 component	Cytoplasmic	8,4,7,12,14
A1AZH6/Pden_0558	23	22	19	21	Succinate--CoA ligase [ADP-forming] subunit alpha	Cytoplasmic	0
A1AZH7/Pden_0559	37	41	39	37	Succinate--CoA ligase [ADP-forming] subunit beta	Cytoplasmic	8,4,7,12,14
A1AZH9/Pden_0561	20	19	22	20	Malate dehydrogenase	Cytoplasmic	8,4,7,12,14
A1AZI1/Pden_0563	7	6	5	3	Citryl-CoA lyase	Cytoplasmic	0
A1AZI4/Pden_0566	9	7	6	5	L-erythro-3-methylmalyl-CoA dehydratase	Cytoplasmic	0
A1AZI7/Pden_0569	40	38	39	32	Succinate dehydrogenase flavoprotein subunit	Membrane	8,4,7,12,14
A1AZJ0/Pden_0572	13	8	15	12	Succinate dehydrogenase iron-sulfur subunit	Membrane	8,4,7,12,14
A1AZJ1/Pden_0573	0	1	2	1	Uncharacterized protein	Cytoplasmic	0
A1AZJ2/Pden_0574	4	2	1	3	UPF0173 metal-dependent hydrolase Pden_0574	Cytoplasmic	0
A1AZJ4/Pden_0576	9	8	10	9	Glutamyl-tRNA amidotransferase subunit A	Cytoplasmic	6,1,8,4,7
A1AZJ5/Pden_0577	3	2	3	2	Uncharacterized protein	Unknown	0
A1AZJ9/Pden_0581	7	5	7	5	Iron-sulfur cluster carrier protein	Cytoplasmic	0
A1AZK0/Pden_0582	1	0	2	1	Transcriptional regulator MraZ	Cytoplasmic	6,1,8,4,7
A1AZK1/Pden_0583	7	5	4	3	Ribosomal RNA small subunit methyltransferase H	Cytoplasmic	6,8,4,7,24
A1AZK4/Pden_0586	11	7	8	8	UDP-N-acetylmuramoyl-L-alanyl-D-glutamate--2,6-diaminopimelate ligase	Cytoplasmic	12,7,14,9,10,19
A1AZK5/Pden_0587	5	6	5	6	UDP-N-acetylmuramoyl-tripeptide--D-alanyl-D-alanine ligase	Cytoplasmic	12,7,14,9,10,19
A1AZK7/Pden_0589	13	8	4	9	UDP-N-acetylmuramoylalanine--D-glutamate ligase	Unknown	12,7,14,9,10,19
A1AZK8/Pden_0590	1	2	1	1	Membrane protein involved in aromatic hydrocarbon degradation	OuterMembrane	0
A1AZL0/Pden_0592	29	30	28	23	Inosine-5'-monophosphate dehydrogenase	Cytoplasmic	8,1,4,7,12,14
A1AZL2/Pden_0594	2	0	2	1	Fmu domain protein	Cytoplasmic	0
A1AZL5/Pden_0597	7	9	7	7	Protein RecA	Cytoplasmic	7,4,12,14,13,29
A1AZL6/Pden_0598	30	30	27	26	Alanine--tRNA ligase	Cytoplasmic	8,6,4,7,12,14
A1AZL7/Pden_0599	6	3	3	4	Uncharacterized protein	Unknown	0
A1AZM1/Pden_0603	17	16	12	15	Adenylosuccinate synthetase	Cytoplasmic	8,1,4,7,12,14
A1AZM3/Pden_0605	1	1	1	2	Thiamine diphosphokinase	Membrane	1,6,4,7,12,14
A1AZM6/Pden_0608	21	23	26	17	Extracellular solute-binding protein, family 5	Periplasmic	0
A1AZM7/Pden_0609	3	2	3	3	ABC transporter related protein	Membrane	5,13
A1AZM8/Pden_0610	11	10	5	9	Amidohydrolase	Cytoplasmic	0
A1AZM9/Pden_0611	27	23	20	20	Dihydrolipoyl dehydrogenase	Cytoplasmic	12,7,14,9,2,10
A1AZN0/Pden_0612	2	1	1	2	Uncharacterized protein	Unknown	0
A1AZN4/Pden_0616	16	14	12	12	Bacterioferritin	Cytoplasmic	12,5,13,10
A1AZN5/Pden_0617	2	1	0	2	HAD-superfamily hydrolase, subfamily IA, variant 3	Cytoplasmic	0

A1AZN6/Pden_0618	12	13	14	13	Bifunctional protein GlmU [Includes: UDP-N-acetylglucosamine pyrophosphorylase ; Glucosamine-1-phosphate N-acetyltransferase]	Cytoplasmic	9,14,31,17,10,19
A1AZN7/Pden_0619	25	27	21	18	Glutamine--fructose-6-phosphate aminotransferase [isomerizing]	Cytoplasmic	8,1,4,7,12,14
A1AZN9/Pden_0621	5	2	5	2	Peptide methionine sulfoxide reductase MsrA -S-oxide reductase) reductase)	Unknown	11,8,4,7
A1AZP0/Pden_0622	5	3	5	3	Ribosomal protein L11 methyltransferase	Cytoplasmic	0
A1AZP2/Pden_0624	1	1	2	1	Ribosomal RNA small subunit methyltransferase E	Cytoplasmic	6,8,4,7
A1AZP4/Pden_0626	2	0	3	2	OmpA/MotB domain protein	Membrane	0
A1AZP8/Pden_0630	20	22	25	20	Aromatic amino acid aminotransferase apoenzyme	Cytoplasmic	8,1,4,7,12,14
A1AZP9/Pden_0631	13	11	9	11	3-mercaptopyruvate sulfurtransferase	Cytoplasmic	0
A1AZQ1/Pden_0633	4	4	2	3	Thiamine pyrophosphate enzyme TPP binding domain protein	Cytoplasmic	0
A1AZR0/Pden_0642	1	0	1	2	Methionyl-tRNA formyltransferase	Cytoplasmic	0
A1AZR1/Pden_0643	2	0	1	3	Dienelactone hydrolase	Unknown	0
A1AZR6/Pden_0648	7	3	4	7	Aminotransferase, class I and II	Cytoplasmic	1
A1AZS0/Pden_0652	4	6	4	1	Aminotransferase	Cytoplasmic	1
A1AZS1/Pden_0653	2	1	2	1	Transcriptional regulator, AsnC family	Cytoplasmic	6,1,8,4,7
A1AZS3/Pden_0655	9	7	5	4	Nitrite/sulfite reductase, hemoprotein beta-component, ferrodoxin domain protein	Cytoplasmic	0
A1AZS5/Pden_0657	2	1	0	1	Uncharacterized protein	Unknown	0
A1AZS6/Pden_0658	9	12	9	12	Ferredoxin--NADP reductase	Cytoplasmic	0
A1AZS7/Pden_0659	17	14	14	10	DNA topoisomerase 1	Cytoplasmic	6,8,3,4,7,
A1AZT2/Pden_0664	9	8	8	5	TRAP transporter solute receptor, TAXI family	Unknown	0
A1AZU8/Pden_0680	1	1	1	2	Transcriptional regulator, GntR family	Cytoplasmic	6,1,8,4,7
A1AZU9/Pden_0681	2	0	1	0	Uncharacterized protein	Unknown	0
A1AZV1/Pden_0683	5	4	3	6	Cell division and transport-associated protein TolQ	Membrane	12
A1AZV3/Pden_0685	2	3	5	4	Cell division and transport-associated protein TolA	OuterMembrane	12
A1AZV4/Pden_0686	13	19	12	15	Protein TolB	Periplasmic	5,13,23
A1AZV5/Pden_0687	7	8	7	5	OmpA domain protein	OuterMembrane	0
A1AZV8/Pden_0690	19	17	16	17	ATP-dependent zinc metalloprotease FtsH	Membrane	8,7,2
A1AZX2/Pden_0704	13	14	12	10	4-hydroxy-tetrahydrodipicolinate synthase	Cytoplasmic	8,1,4,7,12,14
A1AZX5/Pden_0707	6	3	3	5	Inositol monophosphatase	Cytoplasmic	4,8,7,12,14,2
A1AZX7/Pden_0709	4	3	2	2	Formiminoglutamate deiminase	Cytoplasmic	0
A1AZX9/Pden_0711	5	5	2	4	Histidine ammonia-lyase	Cytoplasmic	4,8,7,12,14,2
A1AZY3/Pden_0715	6	6	4	6	Urocanate hydratase	Cytoplasmic	4,8,7,12,14,2
A1AZY4/Pden_0716	10	11	8	9	Amino acid ABC transporter substrate-binding protein, PAAT family	Periplasmic	0
A1AZY8/Pden_0720	6	6	5	3	NADH:flavin oxidoreductase/NADH oxidase	Cytoplasmic	0
A1AZZ1/Pden_0723	1	2	1	3	Uncharacterized protein	Membrane	0
A1AZZ3/Pden_0725	3	2	1	1	TonB family protein	Unknown	5
A1AZZ5/Pden_0727	5	4	2	3	Outer membrane transport energization protein ExbB	Membrane	0
A1AZZ7/Pden_0729	1	2	1	1	Aminotransferase, class V	Cytoplasmic	0
A1AZZ9/Pden_0731	2	2	3	2	Glycine cleavage T protein	Cytoplasmic	0
A1B001/Pden_0733	0	2	1	1	Uncharacterized protein	Unknown	0
A1B002/Pden_0734	52	57	53	48	Elongation factor Tu	Cytoplasmic	0
A1B004/Pden_0736	1	1	2	2	Glyoxalase/bleomycin resistance protein/dioxygenase	Unknown	0
A1B010/Pden_0742	11	13	11	10	Transcription termination/antitermination protein NusG	Cytoplasmic	1,6,8,4,7,9
A1B011/Pden_0743	6	5	6	4	50S ribosomal protein L11	Cytoplasmic	6,1,8,4,7
A1B012/Pden_0744	16	15	16	15	50S ribosomal protein L1	Cytoplasmic	1,6,8,4,7,9

A1B013/Pden_0745	6	6	5	3	50S ribosomal protein L10	Cytoplasmic	1,6,8,4,7,20
A1B014/Pden_0746	8	8	5	5	50S ribosomal protein L7/L12	Unknown	6,1,8,4,7
A1B015/Pden_0747	70	68	67	64	DNA-directed RNA polymerase subunit beta	Cytoplasmic	6,1,8,4,7
A1B017/Pden_0749	69	59	57	55	DNA-directed RNA polymerase subunit beta'	Cytoplasmic	6,1,8,4,7
A1B021/Pden_0753	6	5	7	7	30S ribosomal protein S12	Cytoplasmic	6,1,8,4,7
A1B022/Pden_0754	14	12	13	14	30S ribosomal protein S7	Cytoplasmic	6,1,8,4,7
A1B023/Pden_0755	60	54	49	49	Elongation factor G	Cytoplasmic	0
A1B026/Pden_0758	4	6	7	6	30S ribosomal protein S10	Cytoplasmic	6,1,8,4,7
A1B027/Pden_0759	17	12	17	11	50S ribosomal protein L3	Cytoplasmic	6,1,8,4,7
A1B028/Pden_0760	18	18	18	15	50S ribosomal protein L4	Cytoplasmic	6,1,8,4,7
A1B029/Pden_0761	7	7	5	6	50S ribosomal protein L23	Cytoplasmic	6,1,8,4,7
A1B030/Pden_0762	16	14	18	13	50S ribosomal protein L2	Cytoplasmic	6,1,8,4,7
A1B031/Pden_0763	6	7	7	6	30S ribosomal protein S19	Cytoplasmic	6,1,8,4,7
A1B032/Pden_0764	9	8	7	9	50S ribosomal protein L22	Cytoplasmic	6,1,8,4,7
A1B033/Pden_0765	16	15	12	11	30S ribosomal protein S3	Cytoplasmic	6,1,8,4,7
A1B034/Pden_0766	6	7	5	7	50S ribosomal protein L16	Cytoplasmic	6,1,8,4,7
A1B036/Pden_0768	6	8	5	6	30S ribosomal protein S17	Cytoplasmic	6,1,8,4,7
A1B037/Pden_0769	10	8	7	8	50S ribosomal protein L14	Cytoplasmic	6,1,8,4,7
A1B038/Pden_0770	8	7	8	7	50S ribosomal protein L24	Cytoplasmic	6,1,8,4,7
A1B039/Pden_0771	13	11	11	9	50S ribosomal protein L5	Cytoplasmic	6,1,8,4,7
A1B040/Pden_0772	7	7	7	7	30S ribosomal protein S14	Cytoplasmic	6,1,8,4,7
A1B041/Pden_0773	11	9	8	9	30S ribosomal protein S8	Cytoplasmic	6,1,8,4,7
A1B042/Pden_0774	14	10	10	10	50S ribosomal protein L6	Cytoplasmic	6,1,8,4,7
A1B043/Pden_0775	8	4	5	4	50S ribosomal protein L18	Cytoplasmic	6,1,8,4,7
A1B044/Pden_0776	12	12	12	11	30S ribosomal protein S5	Cytoplasmic	6,1,8,4,7
A1B045/Pden_0777	1	2	1	1	50S ribosomal protein L30	Unknown	6,1,8,4,7
A1B047/Pden_0779	13	13	10	9	50S ribosomal protein L15	Cytoplasmic	6,1,8,4,7
A1B049/Pden_0781	11	12	10	10	Adenylate kinase	Cytoplasmic	8,1,4,7,12,14
A1B050/Pden_0782	11	10	9	12	30S ribosomal protein S13	Cytoplasmic	6,1,8,4,7
A1B051/Pden_0783	11	10	11	10	30S ribosomal protein S11	Cytoplasmic	6,1,8,4,7
A1B052/Pden_0784	16	16	11	11	DNA-directed RNA polymerase subunit alpha	Cytoplasmic	6,1,8,4,7
A1B053/Pden_0785	10	12	7	10	50S ribosomal protein L17	Cytoplasmic	6,1,8,4,7
A1B054/Pden_0786	3	1	2	2	Transcriptional regulator, LuxR family	Unknown	9
A1B058/Pden_0790	1	2	1	1	Pseudouridine synthase	Cytoplasmic	6,8,4,7
A1B060/Pden_0792	4	3	2	1	ATP12 ATPase	Unknown	3
A1B061/Pden_0793	25	21	19	21	L-aspartate-binding protein / L-glutamate-binding protein / L-glutamine-binding protein / L-asparagine-binding protein	Unknown	0
A1B063/Pden_0795	1	1	2	1	L-glutamate ABC transporter membrane protein / L-asparagine ABC transporter membrane protein / L-glutamine ABC transporter membrane protein / L-aspartate ABC transporter membrane protein	Membrane	0
A1B064/Pden_0796	12	11	9	9	L-aspartate ABC transporter ATP-binding protein / L-glutamine ABC transporter ATP-binding protein / L-glutamate ABC transporter ATP-binding protein / L-asparagine ABC transporter ATP-binding protein	Membrane	0
A1B066/Pden_0798	14	11	11	12	Branched chain amino acid: 2-keto-4-methylthiobutyrate aminotransferase	Cytoplasmic	0
A1B067/Pden_0799	13	9	8	5	Citrate -lyase	Cytoplasmic	0
A1B075/Pden_0807	9	9	10	9	ErfK/YbiS/YcfS/YnhG family protein	Unknown	0
A1B078/Pden_0810	0	1	3	2	Extracellular solute-binding protein, family 5	Periplasmic	12,5,13

A1B080/Pden_0812	5	5	6	5	Phosphoserine phosphatase	Cytoplasmic	8,1,4,7,12,14
A1B081/Pden_0813	14	15	10	15	Phosphoserine aminotransferase apoenzyme	Cytoplasmic	8,1,4,7,12,14
A1B082/Pden_0814	39	31	30	28	D-3-phosphoglycerate dehydrogenase	Cytoplasmic	8,1,4,7,12,14
A1B085/Pden_0817	2	0	0	1	CreA family protein	Unknown	0
A1B091/Pden_0823	8	11	8	11	Uncharacterized protein	Cytoplasmic	0
A1B092/Pden_0824	7	7	7	7	Uncharacterized protein	Unknown	0
A1B093/Pden_0825	7	8	4	6	Transglutaminase, N-terminal domain protein	Cytoplasmic	0
A1B094/Pden_0826	2	1	0	2	20S proteasome, A and B subunits	Cytoplasmic	8,7
A1B095/Pden_0827	5	5	2	5	DNA mismatch repair protein MutS	Cytoplasmic	4,8,7,12,14,13
A1B096/Pden_0828	2	2	3	3	Hydratase/decarboxylase	Membrane	0
A1B097/Pden_0829	4	5	6	6	Extracellular solute-binding protein, family 1	Unknown	0
A1B0A0/Pden_0832	2	4	1	3	ABC transporter related protein	Membrane	0
A1B0A2/Pden_0834	2	3	2	3	Glycosyl transferase, WecB/TagA/CpsF family	Cytoplasmic	1
A1B0B2/Pden_0844	2	0	0	0	Glycosyl transferase, family 2	Cytoplasmic	0
A1B0B3/Pden_0845	5	6	6	4	ATPases involved in chromosome partitioning-like protein	Unknown	0
A1B0B4/Pden_0846	4	6	6	6	Lipopolysaccharide biosynthesis	Membrane	8,1,4,7,12,14
A1B0B6/Pden_0848	19	15	14	16	Malate dehydrogenase	Cytoplasmic	4,7,12,14
A1B0B7/Pden_0849	2	1	0	1	Putative outer membrane protein	OuterMembrane	0
A1B0C0/Pden_0852	7	6	2	5	Nicotinate phosphoribosyltransferase	Cytoplasmic	8,1,4,7,12,14
A1B0C1/Pden_0853	5	5	2	5	NH-dependent NAD synthetase	Cytoplasmic	8,1,4,7,12,14
A1B0C2/Pden_0854	1	2	1	1	MORN repeat-containing protein	Periplasmic	0
A1B0C3/Pden_0855	3	6	6	5	Xanthine phosphoribosyltransferase	Cytoplasmic	8,1,4,7,12,14
A1B0C5/Pden_0857	10	9	11	7	Enoyl-[acyl-carrier-protein] reductase [NADH]	Membrane	8,1,4,7,12,14
A1B0C6/Pden_0858	1	2	1	3	Pyridoxine/pyridoxamine 5'-phosphate oxidase	Cytoplasmic	1,6,4,7,12,14
A1B0C7/Pden_0859	3	2	2	2	Cold-shock DNA-binding protein family	Cytoplasmic	9
A1B0C9/Pden_0862	14	14	11	12	Aspartokinase	Cytoplasmic	8,1,4,7,12,14
A1B0D0/Pden_0863	10	17	13	10	PTSINtr with GAF domain, PtsP	Cytoplasmic	5,13
A1B0E4/Pden_0877	5	6	4	6	Membrane protein insertase YidC	Membrane	5,23
A1B0E5/Pden_0878	3	2	1	3	MOSC domain containing protein	Cytoplasmic	0
A1B0E6/Pden_0879	2	2	0	1	Probable GTP-binding protein EngB	Unknown	3,12
A1B0E7/Pden_0880	9	9	6	6	Acetylglutamate kinase	Cytoplasmic	8,1,4,7,12,14
A1B0E8/Pden_0881	7	3	7	8	Putative phosphohistidine phosphatase, SixA	Cytoplasmic	0
A1B0E9/Pden_0882	1	3	3	3	Phosphofructokinase	Cytoplasmic	8,7
A1B0F1/Pden_0884	20	18	18	17	Glutamate--tRNA ligase 1	Cytoplasmic	8,6,4,7,12,14
A1B0F4/Pden_0887	22	22	21	18	Trigger factor	Cytoplasmic	25,12,5,23
A1B0F6/Pden_0889	15	15	15	16	50S ribosomal protein L9	Cytoplasmic	6,1,8,4,7
A1B0F7/Pden_0890	4	4	4	4	30S ribosomal protein S18	Cytoplasmic	6,1,8,4,7
A1B0F8/Pden_0891	13	12	13	13	30S ribosomal protein S6	Cytoplasmic	6,1,8,4,7
A1B0F9/Pden_0892	6	4	5	4	YceI family protein	Unknown	0
A1B0G0/Pden_0893	1	2	2	1	Cytochrome-c peroxidase	Unknown	0
A1B0G1/Pden_0894	4	5	2	4	Peptide chain release factor 1	Cytoplasmic	0
A1B0G3/Pden_0896	5	5	4	5	Uncharacterized protein	Unknown	0
A1B0G5/Pden_0898	2	2	2	2	4-hydroxythreonine-4-phosphate dehydrogenase -L-threonine dehydrogenase)	Cytoplasmic	1,6,4,7,12,14
A1B0G6/Pden_0899	12	13	9	12	Chaperone SurA	Unknown	0
A1B0G7/Pden_0900	2	1	0	2	LPS-assembly protein LptD	Unknown	3,12,5,13,27
A1B0H0/Pden_0903	26	26	24	21	Probable cytosol aminopeptidase	Cytoplasmic	0

A1B0H4/Pden_0907	3	4	3	2	Sec-independent protein translocase protein TatA	Membrane	12,5,13,23
A1B0H5/Pden_0908	2	2	2	2	Twin-arginine translocation protein, TatB subunit	Unknown	12,5,13,23
A1B0H7/Pden_0910	8	3	4	4	Uncharacterized protein	Cytoplasmic	0
A1B0H8/Pden_0911	6	3	4	3	Penicillin amidase, Cysteine peptidase, MEROPS family C59	Unknown	0
A1B0I0/Pden_0913	0	0	0	2	Homogentisate 1,2-dioxygenase	Cytoplasmic	4,8,7,12,14,2
A1B0I7/Pden_0920	25	24	24	25	Serine hydroxymethyltransferase	Cytoplasmic	8,1,4,7,12,14
A1B0I9/Pden_0922	6	6	3	6	Peptide chain release factor 3	Cytoplasmic	9
A1B0J4/Pden_0927	7	6	5	7	Deoxyguanosinetriphosphate triphosphohydrolase-like protein	Cytoplasmic	8,6,4,7,12,14
A1B0J6/Pden_0929	2	1	2	3	Exodeoxyribonuclease III	Cytoplasmic	4,8,7,12,14,13
A1B0J9/Pden_0932	7	7	8	5	3-hydroxybutyrate dehydrogenase	Cytoplasmic	0
A1B0K1/Pden_0934	8	6	9	5	Flavin-dependent thymidylate synthase	Cytoplasmic	8,1,4,7,12,14
A1B0K2/Pden_0935	1	3	2	1	Glyoxalase/bleomycin resistance protein/dioxygenase	Cytoplasmic	0
A1B0K4/Pden_0937	1	2	1	1	Uncharacterized protein	Unknown	0
A1B0K5/Pden_0938	11	7	6	8	Aminotransferase	Cytoplasmic	1
A1B0K8/Pden_0941	3	1	1	1	Malate/L-lactate dehydrogenase	Cytoplasmic	0
A1B0L0/Pden_0943	1	2	0	2	Bifunctional protein PutA	Cytoplasmic	4,8,7,12,14,2
A1B0L2/Pden_0945	11	8	9	5	Substrate-binding region of ABC-type glycine betaine transport system	Periplasmic	0
A1B0L7/Pden_0950	4	5	3	4	Fervidolysin, Serine peptidase, MEROPS family S08A	Cytoplasmic	0
A1B0L8/Pden_0951	9	11	6	8	Ribonucleoside-diphosphate reductase, adenosylcobalamin-dependent	Cytoplasmic	6,1,8,4,7
A1B0L9/Pden_0952	1	2	1	1	DSBA oxidoreductase	Cytoplasmic	0
A1B0M1/Pden_0954	4	6	5	6	Cold-shock DNA-binding protein family	Cytoplasmic	1,6,8,4,7,9
A1B0M2/Pden_0955	12	11	6	11	Threonine--tRNA ligase	Cytoplasmic	8,6,4,7,12,14
A1B0M3/Pden_0956	8	4	3	4	Uncharacterized protein	Cytoplasmic	0
A1B0M4/Pden_0957	12	10	10	11	Polyhydroxyalkanoate depolymerase, intracellular	Cytoplasmic	0
A1B0M5/Pden_0958	2	7	3	3	Poly-hydroxyalkanoic acid synthase, class I	Cytoplasmic	1,7
A1B0M6/Pden_0959	26	20	19	15	Uncharacterized protein	Unknown	0
A1B0M7/Pden_0960	6	2	2	2	Polyhydroxyalkonate synthesis repressor, PhaR	Cytoplasmic	0
A1B0P2/Pden_0975	1	2	2	2	Putative phage repressor	Cytoplasmic	0
A1B0P5/Pden_0978	2	3	1	2	Uncharacterized protein	Unknown	0
A1B0V7/Pden_1040	0	2	0	1	Short-chain dehydrogenase/reductase SDR	Cytoplasmic	0
A1B0W8/Pden_1051	2	2	0	0	Aldehyde dehydrogenase	Cytoplasmic	0
A1B0Y5/Pden_1070	1	2	0	2	Regulatory protein, IclR	Cytoplasmic	0
A1B121/Pden_1107	1	0	1	2	Alcohol dehydrogenase GroES domain protein	Cytoplasmic	0
A1B126/Pden_1112	5	2	2	4	Dihydropyrimidinase	Cytoplasmic	0
A1B127/Pden_1113	3	3	1	1	Amidase, hydantoinase/carbamoylase family	Cytoplasmic	0
A1B133/Pden_1119	7	4	4	5	Reduced coenzyme F420:NADP oxidoreductase	Unknown	8,6,4,7,12,14
A1B134/Pden_1120	3	4	2	1	Methylenetetrahydromethanopterin reductase	Cytoplasmic	0
A1B194/Pden_1183	1	2	1	0	Transcriptional regulator, GntR family	Cytoplasmic	6,1,8,4,7
A1B1A8/Pden_1197	2	1	1	0	NADH:flavin oxidoreductase/NADH oxidase	Cytoplasmic	0
A1B1B5/Pden_1204	2	3	3	2	D-alanine--D-alanine ligase	Cytoplasmic	12,7,14,9,10,19
A1B1B6/Pden_1205	1	4	4	2	Urease accessory protein UreG	Cytoplasmic	6
A1B1B8/Pden_1207	8	7	5	5	Urease accessory protein UreE	Cytoplasmic	3,8,4,7,12,14
A1B1B9/Pden_1208	22	22	23	24	Urease subunit alpha	Cytoplasmic	4,6,7,12,14,2
A1B1C2/Pden_1211	3	2	2	2	Urease subunit gamma	Cytoplasmic	4,6,7,12,14,2
A1B1C3/Pden_1212	5	5	5	2	Urease accessory protein UreD	Cytoplasmic	6
A1B1C7/Pden_1216	1	1	3	4	Activator of Hsp90 ATPase 1 family protein	Unknown	11

A1B1E4/Pden_1233	5	4	4	4	2-dehydro-3-deoxyphosphooctonate aldolase	Cytoplasmic	8,1,4,7,12,14
A1B1E5/Pden_1234	1	2	1	1	Capsule polysaccharide export protein-like protein	Unknown	0
A1B1E8/Pden_1237	2	0	1	2	Uncharacterized protein	Unknown	0
A1B1F2/Pden_1241	8	10	7	8	UDP-3-O-acylglucosamine N-acyltransferase	Cytoplasmic	8,1,4,7,12,14
A1B1F4/Pden_1243	2	0	1	0	3-oxoacyl-[acyl-carrier-protein] synthase II	Membrane	0
A1B1F6/Pden_1245	7	7	5	5	2-keto-3-deoxy-phosphogluconate aldolase	Cytoplasmic	0
A1B1F7/Pden_1246	3	2	2	2	Uncharacterized protein	Unknown	0
A1B1F8/Pden_1247	4	3	5	3	Glutathionylspermidine synthase	Cytoplasmic	0
A1B1F9/Pden_1248	20	29	23	20	Extracellular solute-binding protein, family 5	Periplasmic	12,5,13
A1B1G2/Pden_1251	3	1	2	4	ABC transporter related protein	Membrane	0
A1B1G3/Pden_1252	5	4	5	5	ABC transporter related protein	Membrane	0
A1B1G5/Pden_1254	2	1	1	1	Aldehyde dehydrogenase	Cytoplasmic	0
A1B1G6/Pden_1255	7	4	5	4	Short-chain dehydrogenase/reductase SDR	Cytoplasmic	0
A1B1H0/Pden_1259	3	3	4	2	Periplasmic solute binding protein	Periplasmic	18,5,13
A1B1H5/Pden_1264	3	4	3	3	Peptidyl-tRNA hydrolase	Cytoplasmic	6,1,8,4,7
A1B1H6/Pden_1265	10	7	7	10	ATP-dependent Clp protease proteolytic subunit	Cytoplasmic	0
A1B1H7/Pden_1266	12	8	11	6	ATP-dependent Clp protease ATP-binding subunit ClpX	Cytoplasmic	25
A1B1H8/Pden_1267	1	3	3	1	NADH:ubiquinone oxidoreductase 17.2 kD subunit	Unknown	0
A1B1H9/Pden_1268	1	2	1	1	Mammalian cell entry related domain protein	Unknown	5,13
A1B1I2/Pden_1271	18	15	15	16	Acetyl-CoA carboxylase carboxyltransferase subunit alpha	Cytoplasmic	0
A1B1I3/Pden_1272	2	2	1	1	Biotin carboxyl carrier protein	Unknown	8,1,4,7,12,14
A1B1I6/Pden_1275	36	35	35	31	Transketolase	Cytoplasmic	0
A1B1J1/Pden_1280	4	3	3	3	Uncharacterized protein	Unknown	0
A1B1J4/Pden_1283	7	9	5	4	3-dehydroquinate synthase	Cytoplasmic	8,1,4,7,12,14
A1B1J5/Pden_1284	1	2	2	1	Multisubunit potassium/proton antiporter, PhaG subunit	Membrane	0
A1B1J8/Pden_1287	1	2	1	1	Multisubunit potassium/proton antiporter, PhaD subunit	Membrane	0
A1B1K1/Pden_1290	3	5	2	3	Orn/DAP/Arg decarboxylase 2	Cytoplasmic	0
A1B1K5/Pden_1294	4	1	3	2	Uncharacterized protein	Cytoplasmic	0
A1B1L0/Pden_1299	22	20	19	20	Proline--tRNA ligase	Cytoplasmic	8,6,4,7,12,14
A1B1L3/Pden_1302	4	3	1	3	Polyphosphate kinase	Membrane	1,4,7,12,14
A1B1L4/Pden_1303	11	6	5	5	Ppx/GppA phosphatase	Cytoplasmic	0
A1B1L5/Pden_1304	3	3	5	3	Dipeptidase AC, Metallo peptidase, MEROPS family M19	Cytoplasmic	0
A1B1L6/Pden_1305	3	2	1	2	Antibiotic biosynthesis monooxygenase	Unknown	0
A1B1L7/Pden_1306	6	6	8	7	Cobyrinic acid a,c-diamide synthase	Cytoplasmic	0
A1B1L9/Pden_1308	6	6	4	5	UvrABC system protein B	Cytoplasmic	7,4,12,14,13,29
A1B1M0/Pden_1309	2	3	4	4	ETC complex I subunit conserved region	Unknown	4,12,14
A1B1M5/Pden_1314	18	20	16	17	Periplasmic glucan biosynthesis protein, MdoG	Periplasmic	1,8,7,14
A1B1M6/Pden_1315	2	2	2	3	Glycosyl transferase, family 2	Membrane	0
A1B1M8/Pden_1317	12	12	10	13	Formate--tetrahydrofolate ligase	Cytoplasmic	8,1,4,7,12,14
A1B1N1/Pden_1320	5	4	3	3	Uncharacterized protein	Cytoplasmic	0
A1B1N2/Pden_1321	23	22	17	15	Lysine--tRNA ligase	Cytoplasmic	8,6,4,7,12,14
A1B1N4/Pden_1323	4	4	2	2	Pyridoxamine 5'-phosphate oxidase-related, FMN-binding protein	Unknown	0
A1B1N5/Pden_1324	8	9	6	7	Oligopeptide/dipeptide ABC transporter, ATPase subunit	Membrane	5,13
A1B1N6/Pden_1325	11	13	10	12	Oligopeptide/dipeptide ABC transporter, ATPase subunit	Membrane	5,13
A1B1N9/Pden_1328	55	59	50	50	Extracellular solute-binding protein, family 5	Periplasmic	12,5,13
A1B1R3/Pden_1355	2	2	2	3	Methionine synthase	Cytoplasmic	8,1,4,7,12,14

A1B1T3/Pden_1376	3	2	2	3	Lipoyl synthase	Cytoplasmic	8,4,7
A1B1T4/Pden_1377	4	4	4	2	Redoxin domain protein	Unknown	0
A1B1T7/Pden_1380	5	5	4	5	Uncharacterized protein	Unknown	0
A1B1T8/Pden_1381	4	4	4	3	Invasion associated locus B family protein	Periplasmic	0
A1B1T9/Pden_1382	7	7	7	8	Protease Do	Periplasmic	0
A1B1U1/Pden_1384	11	9	15	8	Ribonucleoside-diphosphate reductase class II	Cytoplasmic	6,1,8,4,7
A1B1U2/Pden_1385	0	0	2	0	5-formyltetrahydrofolate cyclo-ligase	Unknown	0
A1B1U3/Pden_1386	1	0	1	2	Magnesium transporter MgtE	Membrane	5,13
A1B1U4/Pden_1387	2	4	5	4	Inositol monophosphatase	Cytoplasmic	8,4,7,12,14
A1B1U6/Pden_1389	2	0	3	2	Alcohol dehydrogenase, zinc-binding domain protein	Cytoplasmic	0
A1B1U7/Pden_1390	1	0	2	1	Uncharacterized protein	Membrane	0
A1B1U9/Pden_1392	4	4	3	2	Glutaredoxin	Unknown	12,9,10
A1B1V2/Pden_1395	13	16	15	13	Aminotransferase, class V	Cytoplasmic	0
A1B1V3/Pden_1396	4	2	1	4	Glutamine amidotransferase class-I	Cytoplasmic	8,6,4,7,12,14
A1B1V4/Pden_1397	1	0	2	0	L-glutamine synthetase	Cytoplasmic	8,1,4,7,12,14
A1B1V7/Pden_1400	2	2	2	2	ppGpp synthetase I, SpoT/RelA	Cytoplasmic	8,6,4,7,12,14
A1B1V8/Pden_1401	4	0	2	2	DNA-directed RNA polymerase subunit omega	Cytoplasmic	6,1,8,4,7
A1B1W0/Pden_1403	1	1	1	2	Uncharacterized protein	Cytoplasmic	0
A1B1W2/Pden_1405	6	4	5	4	Uncharacterized protein	Membrane	0
A1B1W5/Pden_1408	2	1	0	1	Prephenate dehydrogenase	Membrane	8,1,4,7,12,14
Q51687/Pden_1409	0	2	1	2	Histidinol-phosphate aminotransferase	Cytoplasmic	8,1,4,7,12,14
A1B1X2/Pden_1415	1	3	1	2	Protein-export membrane protein SecF	Membrane	12,5,13,23
A1B1X3/Pden_1416	11	5	11	8	Protein translocase subunit SecD	Membrane	12,5,13,23
A1B1X4/Pden_1417	8	6	8	5	Protein translocase subunit yajC	Membrane	0
A1B1Z6/Pden_1439	0	0	1	2	Uncharacterized protein	Cytoplasmic	0
A1B281/Pden_1525	1	2	1	3	Uncharacterized protein	Membrane	0
A1B284/Pden_1528	2	3	1	3	Uncharacterized protein	Cytoplasmic	0
A1B2A7/Pden_1551	11	15	13	11	Uncharacterized protein	OuterMembrane	0
A1B2A9/Pden_1553	6	7	5	5	N utilization substance protein B homolog	Unknown	1,6,8,4,7,9
A1B2B0/Pden_1554	6	7	4	6	6,7-dimethyl-8-ribityllumazine synthase	Cytoplasmic	1,6,4,7,12,14
A1B2B1/Pden_1555	20	22	22	20	3,4-dihydroxy-2-butanone 4-phosphate synthase	Cytoplasmic	1,6,4,7,12,14
A1B2B5/Pden_1559	21	22	21	20	Polysaccharide export protein	Unknown	0
A1B2B7/Pden_1561	2	3	2	1	Diaminohydroxyphosphoribosylaminopyrimidine deaminase / 5-amino-6-uracil reductase	Cytoplasmic	1,6,4,7,12,14
A1B2B8/Pden_1562	8	3	7	3	Transcriptional repressor NrdR	Cytoplasmic	1,6,8,4,7,9
A1B2B9/Pden_1563	7	8	5	4	TRAP dicarboxylate transporter-DctP subunit	Periplasmic	5
A1B2C3/Pden_1567	8	5	7	7	Nicotinate-nucleotide--dimethylbenzimidazole phosphoribosyltransferase -alpha-phosphoribosyltransferase)	Cytoplasmic	8,1,4,7,12,14
A1B2C5/Pden_1569	56	60	50	50	Extracellular solute-binding protein, family 5	Periplasmic	12,5,13
A1B2E4/Pden_1588	6	6	4	5	UvrABC system protein B	Cytoplasmic	7,4,12,14,13,29
A1B2F4/Pden_1598	1	2	2	1	Uncharacterized protein	Unknown	0
A1B2K2/Pden_1649	2	3	4	4	3-hydroxydecanoyl-[acyl-carrier-protein] dehydratase	Cytoplasmic	8,1,4,7,12,14
A1B2K3/Pden_1650	15	13	16	14	3-oxoacyl-[acyl-carrier-protein] synthase I	Cytoplasmic	0
A1B2K4/Pden_1651	9	7	8	7	Enoyl-[acyl-carrier-protein] reductase [NADH]	Membrane	8,1,4,7,12,14
A1B2K5/Pden_1652	7	8	4	4	Hydrolase or acyltransferase	Cytoplasmic	0
A1B2K6/Pden_1653	9	10	8	7	Lytic murein transglycosylase	Membrane	0
A1B2K8/Pden_1655	3	3	4	3	Elongation factor P	Cytoplasmic	0
A1B2K9/Pden_1656	12	11	8	6	N-acetyl-gamma-glutamyl-phosphate reductase	Cytoplasmic	8,1,4,7,12,14

A1B2L0/Pden_1657	2	3	2	1	3-methyl-2-oxobutanoate hydroxymethyltransferase	Cytoplasmic	1,6,4,7,12,14
A1B2M0/Pden_1667	4	2	4	3	ABC transporter related protein	Membrane	5,13
A1B2M1/Pden_1668	4	2	0	2	ABC transporter related protein	Membrane	5,13
A1B2M4/Pden_1671	25	23	23	20	Extracellular solute-binding protein, family 5	Periplasmic	12,5,13
A1B2M5/Pden_1672	2	2	1	2	5-deoxyglucuronate isomerase	Cytoplasmic	4,8,7,12,14,2
A1B2N0/Pden_1677	1	2	1	1	Xylose isomerase domain protein TIM barrel	Cytoplasmic	0
A1B2N1/Pden_1678	4	1	7	1	Inositol 2-dehydrogenase	Unknown	4,8,7,12,14,2
A1B2S0/Pden_1717	2	1	2	2	Amino acid/amide ABC transporter substrate-binding protein, HAAT family	Unknown	0
A1B2S2/Pden_1719	4	1	4	4	AMP-dependent synthetase and ligase	Cytoplasmic	0
A1B2T2/Pden_1729	2	1	3	1	Alanine dehydrogenase	Cytoplasmic	4,8,7,12,14,2
A1B2T5/Pden_1732	3	1	1	1	Putative glutathione S-transferase	Cytoplasmic	0
A1B2T8/Pden_1735	3	3	2	3	Dyp-type peroxidase family	Periplasmic	12,5,13,10
A1B2T9/Pden_1736	2	3	3	2	Uncharacterized protein	Periplasmic	0
A1B2U5/Pden_1742	0	0	2	1	D-aminoacyl-tRNA deacylase deacylase)	Unknown	4,8,7,12,14,2
A1B2U6/Pden_1743	3	2	2	3	DEAD/DEAH box helicase domain protein	Cytoplasmic	0
A1B2U9/Pden_1746	10	8	11	12	Nucleoside diphosphate kinase	Extracellular	8,1,4,7,12,14
A1B2V1/Pden_1748	6	6	8	5	MltA-interacting MipA family protein	Unknown	0
A1B2V5/Pden_1752	4	5	2	2	Coproporphyrinogen oxidase	Cytoplasmic	6,1,4,7
A1B2V6/Pden_1753	4	3	4	4	Uncharacterized protein	Unknown	0
A1B2V8/Pden_1755	0	0	1	2	16S rRNA mG 1207 methyltransferase	Cytoplasmic	0
A1B2W0/Pden_1757	2	2	2	1	Serine-type D-Ala-D-Ala carboxypeptidase	Membrane	0
A1B2W3/Pden_1760	25	22	20	20	Ketol-acid reductoisomerase)	Cytoplasmic	8,1,4,7,12,14
A1B2W5/Pden_1762	3	2	1	1	DNA-directed DNA polymerase	Cytoplasmic	6,1,8,4,7
A1B2W6/Pden_1763	10	9	7	7	Nucleoside ABC transporter ATP-binding protein	Membrane	0
A1B2W9/Pden_1766	21	17	17	15	Nucleoside-binding protein	Unknown	0
A1B2X0/Pden_1767	2	0	1	1	Ferritin, Dps family protein	Cytoplasmic	11,12,10,
A1B2X2/Pden_1769	8	8	12	8	3-oxoacyl-[acyl-carrier-protein] reductase	Unknown	8,1,4,7,12,14
A1B2X3/Pden_1770	6	5	5	3	Malonyl CoA-acyl carrier protein transacylase	Cytoplasmic	0
A1B2X5/Pden_1772	2	2	4	3	Beta-lactamase domain protein	Unknown	0
A1B2Y3/Pden_1780	10	12	6	10	Efflux transporter, RND family, MFP subunit	Membrane	12,5,13
A1B2Y4/Pden_1781	0	2	0	1	Transporter, hydrophobe/amphiphile efflux-1 family	Membrane	0
A1B2Z3/Pden_1790	4	3	2	1	Translation initiation factor IF-1	Cytoplasmic	0
A1B2Z5/Pden_1792	4	2	1	0	Ribonuclease	Cytoplasmic	0
A1B2Z9/Pden_1796	1	1	1	2	GCN5-related N-acetyltransferase	Cytoplasmic	0
A1B301/Pden_1798	8	11	9	6	Sulfate ABC transporter, periplasmic sulfate-binding protein	Periplasmic	0
A1B307/Pden_1804	5	4	4	3	ABC transporter related protein	Membrane	5,13
A1B310/Pden_1807	11	10	6	9	Extracellular solute-binding protein, family 5	Unknown	12,5,13
A1B311/Pden_1808	7	6	8	7	Cytochrome c, class I	Membrane	0
A1B312/Pden_1809	4	3	3	3	Prephenate dehydratase	Cytoplasmic	8,1,4,7,12,14
A1B313/Pden_1810	3	1	2	0	Uncharacterized protein	Unknown	0
A1B318/Pden_1815	6	7	9	7	Peptide chain release factor 2	Cytoplasmic	0
A1B319/Pden_1816	5	5	3	2	Penicillin-binding protein, 1A family	Membrane	0
A1B321/Pden_1818	1	1	2	1	Aminotransferase	Cytoplasmic	1
A1B322/Pden_1819	7	6	5	6	DSBA oxidoreductase	Unknown	0
A1B323/Pden_1820	5	3	5	2	4-hydroxy-3-methylbut-2-en-1-yl diphosphate synthase - butenyl 4-diphosphate synthase)	Cytoplasmic	8,1,4,7,12,14
A1B324/Pden_1821	2	1	1	2	Uncharacterized protein	Cytoplasmic	0

P43089/Pden_1822	2	0	1	1	5-aminolevulinate synthase	Cytoplasmic	6,1,4,7
A1B326/Pden_1823	18	16	13	14	Peptidase M20	Cytoplasmic	0
A1B328/Pden_1825	6	7	8	8	Transcriptional regulator, CarD family	Cytoplasmic	0
A1B331/Pden_1828	16	14	11	14	Helicase domain protein	Cytoplasmic	0
A1B333/Pden_1830	4	1	4	1	Sterol-binding domain protein	Unknown	0
A1B335/Pden_1832	10	7	4	9	Oligoendopeptidase, pepF/M3 family	Cytoplasmic	0
A1B337/Pden_1834	2	2	3	2	Glutathione S-transferase, C-terminal domain	Cytoplasmic	0
A1B339/Pden_1836	7	9	5	7	DNA topoisomerase 4 subunit B	Cytoplasmic	8,6,3,4,7,12
A1B348/Pden_1845	8	7	9	4	Cbb3-type cytochrome c oxidase subunit CcoP)	Cytoplasmic	7,4,12,14,5,13
A1B350/Pden_1847	3	4	4	3	Cytochrome c oxidase, cbb3-type, subunit II	Cytoplasmic	4,12,14
A1B352/Pden_1849	12	12	15	15	UspA domain protein	Cytoplasmic	11
A1B353/Pden_1850	2	3	3	1	Putative transcriptional regulator, Crp/Fnr family	Membrane	6,1,8,4,7
A1B356/Pden_1853	18	16	11	10	DNA polymerase I	Cytoplasmic	6,1,8,4,7
A1B357/Pden_1854	2	2	2	2	Uncharacterized protein	Unknown	0
A1B358/Pden_1855	3	3	3	3	ABC transporter related protein	Membrane	0
A1B359/Pden_1856	5	3	1	4	Histidine triad protein	Cytoplasmic	0
A1B362/Pden_1859	2	3	2	1	50S ribosomal protein L32	Cytoplasmic	6,1,8,4,7
A1B363/Pden_1860	5	5	4	5	Phosphate acyltransferase	Cytoplasmic	8,1,4,7,12,14
A1B364/Pden_1861	10	9	6	8	3-oxoacyl-[acyl-carrier-protein] synthase 3	Cytoplasmic	8,1,4,7,12,14
A1B366/Pden_1863	6	7	7	8	Integration host factor subunit alpha	Cytoplasmic	1,6,8,4,7,9
A1B367/Pden_1864	1	1	2	1	Putative transcriptional regulator, MerR family	Unknown	9
A1B368/Pden_1865	3	6	4	2	2-deoxycytidine 5-triphosphate deaminase	Cytoplasmic	8,6,4,7,12,14
A1B369/Pden_1866	3	1	1	2	Segregation and condensation protein B	Cytoplasmic	12
A1B371/Pden_1868	2	5	4	3	Beta-N-acetylhexosaminidase	Cytoplasmic	8,7
A1B372/Pden_1869	1	3	2	1	Sporulation domain protein	Unknown	0
A1B373/Pden_1870	19	12	15	19	Arginine--tRNA ligase	Cytoplasmic	8,6,4,7,12,14
A1B374/Pden_1873	19	14	14	13	S-adenosylmethionine synthase	Cytoplasmic	8,1,4,7,12,14
A1B375/Pden_1874	3	2	4	1	Heat shock protein DnaJ domain protein	Cytoplasmic	0
A1B377/Pden_1876	14	12	12	9	Fructose-1,6-bisphosphatase	Cytoplasmic	8,1,4,7,12,14
A1B378/Pden_1877	1	3	2	2	Exonuclease RecJ	Cytoplasmic	4,8,7,12,14,13
A1B380/Pden_1879	33	36	33	32	Carbamoyl-phosphate synthase large chain	Unknown	8,1,4,7,12,14
A1B382/Pden_1881	2	1	0	1	Cell division protein ZapA	Unknown	3,12
A1B383/Pden_1882	1	2	2	1	Uncharacterized protein	Cytoplasmic	0
A1B384/Pden_1883	5	3	2	1	Imidazoleglycerol-phosphate dehydratase	Cytoplasmic	8,1,4,7,12,14
A1B385/Pden_1884	2	3	2	1	Imidazole glycerol phosphate synthase subunit HisH	Cytoplasmic	8,1,4,7,12,14
A1B387/Pden_1886	6	8	7	7	1--5-[methylideneamino] imidazole-4-carboxamide isomerase	Cytoplasmic	8,1,4,7,12,14
A1B391/Pden_1890	5	4	4	4	Ribonuclease D	Cytoplasmic	6,8,4,7
A1B392/Pden_1891	3	4	2	1	Queuine tRNA-ribosyltransferase	Cytoplasmic	8,1,4,7,12,14
A1B395/Pden_1894	2	3	4	2	Uncharacterized protein	Unknown	0
A1B396/Pden_1895	1	3	3	1	Uncharacterized protein	Cytoplasmic	0
A1B398/Pden_1897	2	2	1	1	Betaine aldehyde dehydrogenase	Unknown	0
A1B3A0/Pden_1899	5	3	2	5	Substrate-binding region of ABC-type glycine betaine transport system	Unknown	5,13
A1B3A2/Pden_1901	2	2	1	1	ABC transporter related protein	Membrane	0
A1B3A6/Pden_1905	3	4	3	3	Probable transcriptional regulatory protein Pden_1905	Cytoplasmic	1,6,8,4,7,9
A1B3A9/Pden_1908	17	9	12	13	Fumarate hydratase class II	Cytoplasmic	8,4,7,12,14
A1B3B0/Pden_1909	3	4	4	3	Uncharacterized protein	Cytoplasmic	0

A1B3B2/Pden_1912	0	0	2	0	Uncharacterized protein	Unknown	6,7
A1B3B3/Pden_1913	6	3	0	1	Tyrosine--tRNA ligase	Cytoplasmic	8,6,4,7,12,14
A1B3B6/Pden_1916	6	6	4	4	Peptidyl-prolyl cis-trans isomerase	Unknown	25
A1B3B7/Pden_1917	8	8	7	6	Peptidyl-prolyl cis-trans isomerase	Periplasmic	25
A1B3B8/Pden_1918	18	18	14	15	Phosphoglycerate kinase	Cytoplasmic	4,8,7,12,14,2
A1B3C0/Pden_1920	27	23	23	17	Fructose-bisphosphate aldolase	Cytoplasmic	0
A1B3C1/Pden_1921	7	2	6	4	Signal recognition particle receptor FtsY	Cytoplasmic	12,3,5,13,23,22
A1B3C3/Pden_1923	3	1	1	1	ATP phosphoribosyltransferase	Cytoplasmic	8,1,4,7,12,14
A1B3C5/Pden_1925	15	14	13	12	Histidine--tRNA ligase	Cytoplasmic	8,6,4,7,12,14
A1B3C7/Pden_1927	8	6	7	7	Uncharacterized protein	Unknown	0
A1B3C9/Pden_1929	19	18	15	16	Aspartyl/glutamyl-tRNA amidotransferase subunit B	Cytoplasmic	6,1,8,4,7
A1B3D0/Pden_1931	9	12	10	8	Uncharacterized protein	Unknown	0
A1B3D2/Pden_1933	14	13	9	13	O-acetylhomoserine sulfhydrolase	Cytoplasmic	0
A1B3D4/Pden_1935	14	11	11	14	Methionine synthase	Cytoplasmic	6,4,7
A1B3D6/Pden_1937	1	2	1	2	Cytochrome c, class I	Periplasmic	0
A1B3D7/Pden_1938	2	2	2	1	Cytochrome c oxidase subunit 1	Membrane	4,12,14
A1B3D9/Pden_1940	2	1	1	0	FAD dependent oxidoreductase	Cytoplasmic	0
A1B3E0/Pden_1941	8	7	10	7	Glyoxylate reductase	Cytoplasmic	0
A1B3E1/Pden_1942	1	1	1	2	Uncharacterized protein	Unknown	0
A1B3E3/Pden_1944	6	6	5	6	Peptidyl-dipeptidase Dcp, Metallo peptidase, MEROPS family M03A	Cytoplasmic	0
A1B3E5/Pden_1946	0	2	1	1	Deoxyuridine 5'-triphosphate nucleotidohydrolase	Cytoplasmic	8,6,4,7,12,14
A1B3E7/Pden_1949	3	4	1	2	Putative L-sorbosone dehydrogenase	Membrane	8,7
A1B3E8/Pden_1950	1	1	1	2	Glucose-6-phosphate isomerase	Cytoplasmic	4,8,7,12,14,2
A1B3E9/Pden_1951	0	0	2	0	6-phosphogluconolactonase	Cytoplasmic	8,6,4,7,12,14
A1B3F0/Pden_1952	4	1	4	2	Glucose-6-phosphate 1-dehydrogenase	Cytoplasmic	8,6,4,7,12,14
A1B3F3/Pden_1955	2	7	5	4	6-phosphogluconate dehydratase	Cytoplasmic	4,8,7,12,14,2
A1B3F7/Pden_1959	9	8	11	9	Histidinol dehydrogenase	Cytoplasmic	8,1,4,7,12,14
A1B3F8/Pden_1960	2	3	3	3	Uncharacterized protein	Cytoplasmic	0
A1B3F9/Pden_1961	15	13	14	10	UDP-N-acetylglucosamine 1-carboxyvinyltransferase	Cytoplasmic	12,7,14,9,10,19
A1B3G7/Pden_1969	1	0	0	2	Uncharacterized protein	Cytoplasmic	0
A1B3H6/Pden_1978	3	3	3	2	Periplasmic binding protein/LacI transcriptional regulator	Cytoplasmic	6,1,8,4,7
A1B3H7/Pden_1979	7	7	6	4	Methionine aminopeptidase	Cytoplasmic	8,4,7
A1B3H8/Pden_1980	5	5	2	4	Molybdopterin binding domain	Cytoplasmic	0
A1B3H9/Pden_1981	1	1	2	3	GCN5-related N-acetyltransferase	Periplasmic	0
A1B3I2/Pden_1984	11	10	9	6	Dihydroxy-acid dehydratase	Cytoplasmic	8,1,4,7,12,14
A1B3I3/Pden_1985	1	2	2	1	Putative pterin-4-alpha-carbinolamine dehydratase	Cytoplasmic	6,1,4,7
A1B3I5/Pden_1987	6	8	5	3	Acetyl-coenzyme A carboxylase carboxyl transferase subunit beta	Cytoplasmic	8,1,4,7,12,14
A1B3I6/Pden_1988	2	2	1	0	FolC bifunctional protein	Cytoplasmic	0
A1B3I7/Pden_1989	6	5	4	6	AFG1-family ATPase	Cytoplasmic	0
A1B3I8/Pden_1990	4	1	1	3	Uncharacterized protein	Unknown	0
A1B3J3/Pden_1995	14	12	13	10	HemY domain protein	Unknown	0
A1B3J4/Pden_1996	12	17	17	13	Uncharacterized protein	Unknown	0
A1B3J9/Pden_2001	0	1	2	0	Uncharacterized protein	Cytoplasmic	0
A1B3K0/Pden_2002	1	2	3	3	Lipid A ABC exporter family, fused ATPase and inner membrane subunits	Membrane	0
A1B3K2/Pden_2004	3	3	2	3	Peptidoglycan-binding LysM	Unknown	0

A1B3K3/Pden_2005	2	2	0	1	AMP-dependent synthetase and ligase	Cytoplasmic	0
A1B3K6/Pden_2008	22	20	21	19	ABC transporter related protein	Cytoplasmic	0
A1B3K8/Pden_2010	0	0	2	0	Ribosomal protein S12 methylthiotransferase RimO)-methylthiotransferase)	Cytoplasmic	6,8,4,7
A1B3L4/Pden_2016	4	2	1	2	Uncharacterized protein	Membrane	0
A1B3L8/Pden_2020	4	6	4	3	Diaminopimelate decarboxylase	Cytoplasmic	8,1,4,7,12,14
A1B3M0/Pden_2022	8	9	8	7	Argininosuccinate lyase	Cytoplasmic	8,1,4,7,12,14
A1B3M4/Pden_2026	29	28	30	30	Acetyl-CoA acetyltransferase	Cytoplasmic	0
A1B3M5/Pden_2027	17	13	13	12	3-oxoacyl-[acyl-carrier-protein] reductase	Cytoplasmic	1,7
A1B3M9/Pden_2031	7	6	7	6	Trans-hexaprenyltranstransferase	Cytoplasmic	8,1,4,7,12,14
A1B3N0/Pden_2032	2	2	2	2	Ammonium transporter	Membrane	0
A1B3N1/Pden_2033	3	8	4	4	Penicillin-binding protein, 1A family	Membrane	0
A1B3N2/Pden_2034	3	1	1	1	Toluene tolerance family protein	Unknown	0
A1B3N7/Pden_2039	6	7	6	4	Ornithine carbamoyltransferase	Cytoplasmic	8,1,4,7,12,14
A1B3N9/Pden_2041	12	13	8	10	Acetylornithine aminotransferase	Cytoplasmic	8,1,4,7,12,14
A1B3P1/Pden_2043	4	6	4	7	GcrA cell cycle regulator	Unknown	0
A1B3P3/Pden_2045	10	11	7	7	Methionine--tRNA ligase	Cytoplasmic	8,6,4,7,12,14
A1B3P4/Pden_2046	17	20	15	15	TonB-dependent receptor	OuterMembrane	5
A1B3P5/Pden_2047	6	4	4	4	Arginase	Unknown	8,6,4,7,12,14
A1B3T6/Pden_2088	14	13	9	11	Endonuclease	Unknown	0
A1B3T8/Pden_2090	3	3	1	5	Uncharacterized protein	Cytoplasmic	0
A1B3U2/Pden_2094	1	2	0	1	Tetraacyldisaccharide 4'-kinase	Cytoplasmic	8,1,4,7,12,14
A1B3U3/Pden_2095	8	8	9	7	DSBA oxidoreductase	Unknown	0
A1B3V1/Pden_2103	4	5	3	5	Ureidoglycolate lyase	Cytoplasmic	4,8,7,12,14,2
A1B3V3/Pden_2105	4	4	4	4	Transcriptional regulator, GntR family	Cytoplasmic	6,1,8,4,7
A1B3V4/Pden_2106	2	2	3	1	ErfK/YbiS/YcfS/YnhG family protein	Unknown	0
A1B3W7/Pden_2119	2	2	1	1	Glucose-1-phosphate thymidylyltransferase	Cytoplasmic	8,1,4,7,12,14
A1B3X0/Pden_2122	0	2	1	1	dTDP-4-dehydrorhamnose 3,5-epimerase	Cytoplasmic	0
A1B3X4/Pden_2126	8	4	9	8	Capsule polysaccharide export protein-like protein	Membrane	0
A1B3X6/Pden_2128	2	2	3	3	Uncharacterized protein	Unknown	0
A1B3Y0/Pden_2132	6	5	6	4	Anthranilate synthase, component I	Cytoplasmic	8,1,4,7,12,14
A1B3Y1/Pden_2133	13	13	9	11	Putative peptidyl-prolyl cis-trans isomerase	Membrane	0
A1B3Y2/Pden_2134	1	0	1	2	Aminotransferase	Cytoplasmic	1
A1B3Y4/Pden_2136	6	4	3	5	Anthranilate synthase, component II	Cytoplasmic	0
A1B3Y5/Pden_2137	5	4	3	3	Anthranilate phosphoribosyltransferase	Membrane	8,1,4,7,12,14
A1B3Y7/Pden_2139	7	6	7	4	Indole-3-glycerol phosphate synthase	Cytoplasmic	8,1,4,7,12,14
A1B3Y8/Pden_2140	1	0	2	1	Cyclic pyranopterin monophosphate synthase accessory protein	Cytoplasmic	6,1,8,4,7
A1B3Y9/Pden_2141	4	6	2	3	Molybdopterin molybdochelatase	Cytoplasmic	6,1,4,7
A1B3Z0/Pden_2142	7	5	3	7	LexA repressor	Cytoplasmic	12,7,14,9,13,29
A1B3Z1/Pden_2143	10	6	4	7	Serine-type D-Ala-D-Ala carboxypeptidase	Membrane	0
A1B3Z2/Pden_2144	3	2	2	1	Thymidylate kinase	Cytoplasmic	8,1,4,7,12,14
A1B3Z4/Pden_2146	5	2	5	2	Hydrolase, TatD family	Cytoplasmic	0
A1B3Z7/Pden_2149	2	2	2	1	Binding-protein-dependent transport systems inner membrane component	Membrane	5
A1B3Z8/Pden_2150	4	3	4	4	Binding-protein-dependent transport systems inner membrane component	Membrane	5
A1B3Z9/Pden_2151	14	11	12	14	Polyamine ABC trasnporter, periplasmic polyamine-binding protein	Periplasmic	0
A1B400/Pden_2152	9	6	7	6	Extracellular solute-binding protein, family 1	Periplasmic	0

A1B401/Pden_2153	16	10	8	9	Polyamine-transporting ATPase	Membrane	0
A1B403/Pden_2155	2	0	0	2	Short-chain dehydrogenase/reductase SDR	Cytoplasmic	0
A1B412/Pden_2164	2	0	1	1	GatB/Yqey domain protein	Cytoplasmic	0
A1B413/Pden_2165	6	7	4	5	Carbamoyl-phosphate synthase small chain	Cytoplasmic	8,1,4,7,12,14
A1B417/Pden_2169	13	10	10	7	Acetolactate synthase	Cytoplasmic	8,1,4,7,12,14
A1B418/Pden_2170	8	6	6	7	Acetolactate synthase, small subunit	Cytoplasmic	8,1,4,7,12,14
A1B423/Pden_2175	5	4	5	4	Nitroreductase	Cytoplasmic	0
A1B426/Pden_2178	1	2	2	0	Methylmalonyl-CoA epimerase	Cytoplasmic	0
A1B428/Pden_2180	0	2	2	1	GCN5-related N-acetyltransferase	Unknown	0
A1B431/Pden_2183	21	20	23	24	Aspartate--tRNA ligase	Cytoplasmic	8,6,4,7,12,14
A1B433/Pden_2185	4	6	2	3	Glycoside hydrolase, family 25	Unknown	6,8,4,7,2
A1B435/Pden_2187	6	8	5	7	D-lactate dehydrogenase	Cytoplasmic	0
A1B437/Pden_2189	16	13	18	14	Cobaltochelatase CobS subunit	Cytoplasmic	0
A1B439/Pden_2191	6	6	3	7	Cobaltochelatase CobT subunit	Cytoplasmic	1,6,4,7,12,14
A1B440/Pden_2192	1	1	1	2	Glycosyl transferase, group 1	Cytoplasmic	0
A1B441/Pden_2193	13	13	9	8	Peptidase M24	Cytoplasmic	0
A1B443/Pden_2195	1	3	1	2	Hydroxypyruvate reductase	Cytoplasmic	0
A1B444/Pden_2196	5	5	4	5	D-isomer specific 2-hydroxyacid dehydrogenase, NAD-binding protein	Cytoplasmic	0
A1B445/Pden_2197	2	1	1	0	Export-related chaperone CsaA	Cytoplasmic	0
A1B446/Pden_2198	7	7	5	5	Pyrroline-5-carboxylate reductase	Cytoplasmic	8,1,4,7,12,14
A1B449/Pden_2201	7	7	4	6	N5-carboxyaminoimidazole ribonucleotide synthase imidazole ribonucleotide synthetase)	Membrane	8,1,4,7,12,14
A1B450/Pden_2202	4	5	1	2	N5-carboxyaminoimidazole ribonucleotide mutase imidazole ribonucleotide mutase)	Unknown	8,1,4,7,12,14
A1B452/Pden_2204	8	8	10	7	Uncharacterized protein	Unknown	0
A1B453/Pden_2205	1	1	0	2	Heat shock protein Hsp20	Cytoplasmic	0
A1B456/Pden_2208	13	13	8	9	Thioredoxin reductase	Cytoplasmic	4,12,14,21
A1B457/Pden_2209	4	3	5	4	Transcriptional regulator, AsnC family	Cytoplasmic	6,1,8,4,7
A1B459/Pden_2211	4	3	4	2	Laccase domain protein	Unknown	0
A1B463/Pden_2215	1	2	1	2	Ribosomal large subunit pseudouridine synthase D	Cytoplasmic	6,8,4,7
A1B464/Pden_2216	4	5	3	1	RNA polymerase sigma factor RpoH	Cytoplasmic	7,4,12,31,27,16
A1B465/Pden_2217	1	2	0	1	Uncharacterized protein	Cytoplasmic	0
A1B467/Pden_2219	7	5	6	7	Uracil phosphoribosyltransferase	Cytoplasmic	8,1,4,7,12,14
A1B469/Pden_2221	3	1	1	2	Phosphopentomutase	Cytoplasmic	4,8,7,12,14,2
A1B470/Pden_2222	3	2	2	2	Thymidine phosphorylase	Unknown	8,6,4,7,12,14
A1B471/Pden_2223	1	2	0	1	Cytidine deaminase	Cytoplasmic	0
A1B472/Pden_2224	23	21	20	20	Malate dehydrogenase)., Phosphate acetyltransferase	Cytoplasmic	4,7,12,14
A1B474/Pden_2226	16	15	16	14	3-phosphoshikimate 1-carboxyvinyltransferase	Cytoplasmic	8,1,4,7,12,14
A1B475/Pden_2227	2	2	3	2	tRNA -)-methyltransferase -N)-methyltransferase) -methyltransferase)	Cytoplasmic	0
A1B476/Pden_2228	11	12	10	11	Beta-lactamase domain protein	Cytoplasmic	0
A1B478/Pden_2230	2	2	3	2	Biotin--acetyl-CoA-carboxylase ligase	Cytoplasmic	8,4,7
A1B486/Pden_2238	5	3	6	5	NADH-quinone oxidoreductase subunit I	Cytoplasmic	0
A1B488/Pden_2240	1	1	2	2	Uncharacterized protein	Unknown	0
A1B489/Pden_2241	29	32	31	28	NADH-quinone oxidoreductase	Cytoplasmic	4,12,14
A1B491/Pden_2243	15	14	11	12	NADH dehydrogenase subunit F	Cytoplasmic	0
A1B493/Pden_2245	2	2	3	1	Uncharacterized protein	Membrane	0
A1B494/Pden_2246	8	6	9	10	NADH dehydrogenase subunit E	Unknown	0

A1B495/Pden_2247	12	11	10	11	NADH-quinone oxidoreductase subunit D	Cytoplasmic	5
A1B496/Pden_2248	12	7	10	9	NADH-quinone oxidoreductase subunit C	Cytoplasmic	5
A1B497/Pden_2249	3	5	2	4	NADH-quinone oxidoreductase subunit B	Membrane	5
A1B4A0/Pden_2252	5	6	4	8	UvrABC system protein A	Cytoplasmic	7,4,12,14,13,29
A1B4A3/Pden_2255	1	1	1	2	Uncharacterized protein	Periplasmic	0
A1B4A5/Pden_2257	23	21	21	14	Succinate semialdehyde dehydrogenase	Cytoplasmic	4,8,7,12,14,2
A1B4A6/Pden_2258	10	10	12	12	Uncharacterized protein	Unknown	0
A1B4B0/Pden_2262	18	21	20	19	Phenylalanine--tRNA ligase beta subunit	Cytoplasmic	8,6,4,7,12,14
A1B4B1/Pden_2263	5	1	3	3	Uncharacterized protein	Unknown	0
A1B4B2/Pden_2264	11	11	7	10	Glutathione S-transferase, N-terminal domain	Cytoplasmic	0
A1B4B3/Pden_2265	1	1	1	2	Large-conductance mechanosensitive channel	Membrane	0
A1B4B5/Pden_2267	3	4	4	3	30S ribosomal protein S21	Unknown	6,1,8,4,7
A1B4B6/Pden_2268	1	1	2	2	RpsU-divergently transcribed protein	Cytoplasmic	1,4,7,12,14
A1B4B7/Pden_2269	2	3	2	3	Alcohol dehydrogenase, zinc-binding domain protein	Cytoplasmic	0
A1B4B8/Pden_2270	8	7	9	6	Phenylalanine--tRNA ligase alpha subunit	Cytoplasmic	8,6,4,7,12,14
A1B4B9/Pden_2271	3	2	2	0	Arsenate reductase	Cytoplasmic	0
A1B4C1/Pden_2273	4	2	4	3	50S ribosomal protein L20	Cytoplasmic	1,6,8,3,4,7
A1B4C4/Pden_2276	25	23	20	19	Pyruvate kinase	Cytoplasmic	0
A1B4C5/Pden_2277	1	0	2	0	N-formylglutamate amidohydrolase	Cytoplasmic	6,7,2
A1B4D2/Pden_2285	3	5	3	6	Substrate-binding region of ABC-type glycine betaine transport system	Periplasmic	5,13
A1B4D3/Pden_2286	4	2	5	5	Sulfatase	Cytoplasmic	0
A1B4D6/Pden_2289	2	2	2	1	Glutathione S-transferase, N-terminal domain	Cytoplasmic	0
A1B4E3/Pden_2296	0	0	2	2	UDP-galactose 4-epimerase	Cytoplasmic	8,7,14
A1B4E4/Pden_2297	13	9	10	9	UDP-glucose pyrophosphorylase	Cytoplasmic	1
A1B4E6/Pden_2299	5	5	4	5	3-deoxy-manno-octulosonate cytidylyltransferase	Cytoplasmic	0
A1B4E7/Pden_2300	8	6	7	5	3',5'-bisphosphate nucleotidase	Cytoplasmic	8,4,7,12,14
A1B4E9/Pden_2302	20	24	20	17	Chaperone protein DnaK	Cytoplasmic	25
A1B4F0/Pden_2303	5	3	3	4	Chaperone protein DnaJ	Cytoplasmic	1,11,8,4,7,27
A1B4F2/Pden_2305	9	11	9	11	Ubiquinol-cytochrome c reductase iron-sulfur subunit	Membrane	0
A1B4F3/Pden_2306	3	3	2	1	Cytochrome b	Membrane	4,12,14
A1B4F4/Pden_2307	4	6	5	5	Cytochrome c1	Unknown	0
A1B4F5/Pden_2308	1	1	1	2	Ribokinase	Cytoplasmic	8,7,14
A1B4F8/Pden_2311	1	2	1	2	Short-chain dehydrogenase/reductase SDR	Cytoplasmic	0
A1B4G1/Pden_2314	3	2	2	3	Uncharacterized protein	Unknown	0
A1B4G2/Pden_2315	1	1	3	2	Mammalian cell entry related domain protein	Unknown	0
A1B4G6/Pden_2319	3	1	2	2	Methylenetetrahydrofolate reductase	Cytoplasmic	8,1,4,7,12,14
A1B4G8/Pden_2321	5	5	5	7	Orn/DAP/Arg decarboxylase 2	Cytoplasmic	6,1,4,7
A1B4H1/Pden_2324	6	5	3	4	FAD-dependent pyridine nucleotide-disulfide oxidoreductase	Cytoplasmic	12,9,10
A1B4H2/Pden_2325	3	3	4	2	Thiol peroxidase	Unknown	0
A1B4H8/Pden_2331	33	32	30	29	Valine--tRNA ligase	Cytoplasmic	8,6,4,7,12,14
A1B4H9/Pden_2332	0	2	1	1	Cobalamin biosynthesis CbiX protein	Cytoplasmic	1,6,4,7,12,14
A1B4I0/Pden_2333	7	3	3	6	Uncharacterized protein	Cytoplasmic	0
A1B4I5/Pden_2338	7	3	4	7	4-aminobutyrate aminotransferase apoenzyme	Cytoplasmic	8,6,4,7,12,14
A1B4I6/Pden_2339	10	7	7	7	Acetyl-coenzyme A carboxylase carboxyl transferase subunit alpha	Cytoplasmic	8,1,4,7,12,14
A1B4J2/Pden_2346	16	14	21	14	Glycine dehydrogenase)	Cytoplasmic	4,8,7,12,14,2
A1B4J4/Pden_2348	5	7	5	6	Aminomethyltransferase	Cytoplasmic	4,8,7,12,14,2

A1B4J5/Pden_2349	9	9	8	8	Short-chain dehydrogenase/reductase SDR	Cytoplasmic	0
A1B4L0/Pden_2364	17	25	18	16	Alpha-2-macroglobulin domain protein	Unknown	0
A1B4L2/Pden_2366	2	2	3	2	Aldehyde dehydrogenase)	Cytoplasmic	0
A1B4L3/Pden_2367	2	2	2	2	Alcohol dehydrogenase GroES domain protein	Cytoplasmic	0
A1B4L6/Pden_2370	5	4	4	5	Peptidase S16, lon domain protein	Cytoplasmic	8,7,2
A1B4L7/Pden_2371	4	7	4	3	Thioredoxin domain	Cytoplasmic	7,12,14,9,10,
A1B4L8/Pden_2372	9	5	7	8	Exodeoxyribonuclease III Xth	Cytoplasmic	4,8,7,12,14,13
A1B4M0/Pden_2374	6	6	5	4	Two component transcriptional regulator, winged helix family	Cytoplasmic	1,6,8,4,7,9
A1B4M1/Pden_2375	2	2	1	2	GTP cyclohydrolase-2	Cytoplasmic	1,6,4,7,12,14
A1B4M3/Pden_2377	5	3	3	3	Short-chain dehydrogenase/reductase SDR	Cytoplasmic	0
A1B4N1/Pden_2386	4	1	2	1	2,3-dihydroxybenzoate-AMP ligase	Cytoplasmic	1,6,4,7,12,14
A1B4N7/Pden_2393	10	7	7	9	6-phosphogluconate dehydrogenase, decarboxylating	Unknown	8,6,4,7,12,14
A1B4Q0/Pden_2406	4	5	6	5	D-alanine--D-alanine ligase	Cytoplasmic	12,7,14,9,10,19
A1B4Q1/Pden_2407	3	4	3	3	Mur ligase, middle domain protein	Membrane	12,7,14,9,10,19
A1B4Q4/Pden_2410	2	1	0	1	Uncharacterized protein	Cytoplasmic	0
A1B4Q5/Pden_2411	6	3	1	3	Outer membrane efflux protein	Cytoplasmic	0
A1B4R8/Pden_2425	4	2	2	1	Uncharacterized protein	Membrane	0
A1B4R9/Pden_2426	17	14	14	15	Pyrrolo-quinoline quinone	OuterMembrane	0
A1B4S0/Pden_2427	12	10	6	8	GTPase Der	Membrane	20
A1B4S2/Pden_2429	18	22	20	15	Serine--tRNA ligase synthetase)	Cytoplasmic	8,1,4,7,12,14
A1B4S6/Pden_2433	5	5	6	5	50S ribosomal protein L28	Cytoplasmic	6,1,8,4,7
A1B4W5/Pden_2472	4	4	4	4	Carbohydrate ABC transporter substrate-binding protein, CUT1 family	Periplasmic	0
A1B4X1/Pden_2478	3	4	4	3	Putative transcriptional regulator, Crp/Fnr family	Cytoplasmic	1,6,8,4,7,9
Q51664/Pden_2482	0	0	2	0	Protein NorQ	Cytoplasmic	0
A1B4X7/Pden_2484	2	0	0	0	Nitric oxide reductase, NorC subunit apoprotein	Membrane	0
A1B4Z0/Pden_2497	54	54	44	47	Catalase-peroxidase	Cytoplasmic	11,4,12,14,2
A1B4Z2/Pden_2499	16	13	13	12	Probable transaldolase	Cytoplasmic	8,6,4,7,12,14
A1B4Z3/Pden_2500	2	0	0	1	Uncharacterized protein	Membrane	0
A1B4Z6/Pden_2503	3	2	2	1	Asparaginase	Cytoplasmic	0
A1B4Z7/Pden_2504	5	4	6	6	Uncharacterized protein	Unknown	0
A1B4Z9/Pden_2506	10	9	8	8	2,3-bisphosphoglycerate-independent phosphoglycerate mutase	Cytoplasmic	4,8,7,12,14,2
A1B501/Pden_2508	16	15	11	14	Carboxyl-terminal protease	Membrane	0
A1B502/Pden_2509	1	1	3	2	RNA pyrophosphohydrolase nucleoside polyphosphate hydrolase)	Cytoplasmic	0
A1B503/Pden_2510	19	16	17	16	Lytic murein transglycosylase	Membrane	0
A1B505/Pden_2512	1	2	1	0	YbaK/prolyl-tRNA synthetase associated region	Unknown	0
A1B507/Pden_2514	11	7	9	9	2,3,4,5-tetrahydropyridine-2,6-dicarboxylate N-succinyltransferase	Cytoplasmic	8,1,4,7,12,14
A1B508/Pden_2515	3	3	0	4	Uncharacterized protein	Cytoplasmic	0
A1B511/Pden_2518	2	1	2	1	Iojap-like protein	Cytoplasmic	0
A1B513/Pden_2520	20	19	18	15	3-isopropylmalate dehydratase large subunit	Cytoplasmic	8,1,4,7,12,14
A1B515/Pden_2522	6	10	7	6	3-isopropylmalate dehydratase small subunit	Cytoplasmic	8,1,4,7,12,14
A1B522/Pden_2529	2	0	0	0	Cobyrinic acid a,c-diamide adenosyltransferase	Cytoplasmic	1,6,4,7,12,14
A1B524/Pden_2531	6	6	8	6	Cobalamin biosynthesis protein CobW	Cytoplasmic	1,6,4,7,12,14
A1B527/Pden_2534	3	1	2	0	Precorrin-8X methylmutase	Unknown	1,6,4,7,12,14
A1B529/Pden_2536	1	2	1	1	Precorrin-3 methyltransferase	Cytoplasmic	1,6,4,7,12,14
A1B533/Pden_2540	0	2	2	0	Precorrin-4 C11-methyltransferase	Cytoplasmic	1,6,4,7,12,14

A1B534/Pden_2541	3	2	0	0	Hydrogenobyrinate a,c-diamide synthase	Cytoplasmic	8,1,4,7,12,14
A1B539/Pden_2546	11	9	8	11	DNA topoisomerase 4 subunit A	Cytoplasmic	8,6,3,4,7,12
A1B542/Pden_2549	4	7	6	6	ATP:cobalamin adenosyltransferase	Cytoplasmic	0
A1B543/Pden_2550	16	17	17	16	Electron transfer flavoprotein beta-subunit	Cytoplasmic	0
A1B544/Pden_2551	11	9	12	10	Electron transfer flavoprotein, alpha subunit	Unknown	0
A1B545/Pden_2552	9	7	2	4	3-hydroxyacyl-CoA dehydrogenase	Cytoplasmic	8,4,7,12,14
A1B547/Pden_2554	6	8	4	5	Dihydroorotase	Cytoplasmic	8,1,4,7,12,14
A1B548/Pden_2555	2	2	2	2	Aspartate carbamoyltransferase	Cytoplasmic	8,1,4,7,12,14
A1B549/Pden_2556	3	2	2	1	Uroporphyrinogen-III C-methyltransferase	Cytoplasmic	6,1,4,7
A1B553/Pden_2560	5	3	5	6	Enoyl-CoA hydratase/isomerase	Cytoplasmic	0
A1B559/Pden_2566	10	11	5	9	Uncharacterized protein	Unknown	0
A1B560/Pden_2567	3	4	3	3	Periplasmic binding protein	Unknown	0
A1B565/Pden_2572	6	4	4	4	Ribulose-phosphate 3-epimerase	Cytoplasmic	8,6,4,7,12,14
A1B566/Pden_2573	1	2	1	1	33 kDa chaperonin	Cytoplasmic	25
A1B573/Pden_2580	7	7	7	5	Allergen V5/Tpx-1 family protein	Unknown	0
A1B574/Pden_2581	2	1	1	1	ErfK/YbiS/YcfS/YnhG family protein	Unknown	0
A1B576/Pden_2583	4	2	2	2	Ferrochelatase	Cytoplasmic	1,6,4,7,12,14
A1B577/Pden_2584	0	0	3	0	SAM-dependent methyltransferase	Cytoplasmic	0
A1B582/Pden_2589	4	2	2	4	Ubiquinone biosynthesis O-methyltransferase	Cytoplasmic	1,4,7,12,14
A1B584/Pden_2591	1	1	2	1	Ribosome maturation factor RimP	Cytoplasmic	20
A1B585/Pden_2592	25	24	19	17	Transcription termination/antitermination protein NusA	Cytoplasmic	1,6,8,4,7,9
A1B586/Pden_2593	2	1	3	1	Uncharacterized protein	Unknown	0
A1B587/Pden_2594	30	30	27	29	Translation initiation factor IF-2	Unknown	0
A1B590/Pden_2597	25	19	22	21	Arginine biosynthesis bifunctional protein ArgJ [Cleaved into: Arginine biosynthesis bifunctional protein ArgJ alpha chain; Arginine biosynthesis bifunctional protein ArgJ beta chain] [Includes: Amino-acid acetyltransferase ; Glutamate N-acetyltransferase]	Cytoplasmic	8,1,4,7,12,14
A1B591/Pden_2598	7	5	4	2	Peptidylprolyl isomerase	Membrane	0
A1B592/Pden_2599	36	29	27	29	Protein translocase subunit SecA	Cytoplasmic	12,5,13,23
A1B594/Pden_2601	0	0	2	0	Transcriptional regulator, MarR family	Cytoplasmic	6,1,8,4,7
A1B595/Pden_2602	15	14	11	12	Branched chain amino acid aminotransferase apoenzyme	Cytoplasmic	8,6,4,7,12,14
A1B596/Pden_2603	2	1	1	1	PTS IIA-like nitrogen-regulatory protein PtsN	Cytoplasmic	0
A1B597/Pden_2604	12	11	9	10	SSU ribosomal protein S30P / sigma 54 modulation protein	Cytoplasmic	8
A1B598/Pden_2605	3	3	1	1	ABC transporter related protein	Cytoplasmic	12,5,13
A1B5A0/Pden_2607	1	0	1	2	Uncharacterized protein	Unknown	0
A1B5A1/Pden_2608	4	4	4	3	KpsF/GutQ family protein	Unknown	8,7
A1B5A2/Pden_2609	5	2	4	3	3'-5' exonuclease	Cytoplasmic	0
A1B5A3/Pden_2610	26	26	22	24	TonB-dependent receptor	OuterMembrane	5
A1B5A6/Pden_2613	9	10	8	7	Formyltetrahydrofolate deformylase hydrolase)	Cytoplasmic	8,1,4,7,12,14
A1B5A7/Pden_2614	6	3	4	5	Bifunctional protein FolD 1 [Includes: Methylenetetrahydrofolate dehydrogenase ; Methenyltetrahydrofolate cyclohydrolase]	Cytoplasmic	8,1,4,7,12,14
A1B5B0/Pden_2617	2	1	1	1	Iron-containing alcohol dehydrogenase	Cytoplasmic	0
A1B5B3/Pden_2620	0	2	2	3	Kynureninase	Cytoplasmic	4,8,7,12,14,2
A1B5B6/Pden_2623	5	3	3	3	Uncharacterized protein	Unknown	0
A1B5E6/Pden_2653	0	1	3	3	Efflux transporter, RND family, MFP subunit	Unknown	12,5,13
A1B5E7/Pden_2654	2	1	0	0	Efflux transporter, RND family, MFP subunit	Unknown	12,5,13
A1B5F6/Pden_2663	2	2	3	1	Acetyl-CoA acetyltransferase	Cytoplasmic	0

A1B5G2/Pden_2669	5	2	2	1	Outer-membrane lipoprotein carrier protein	Unknown	5,23
A1B5G4/Pden_2671	5	7	3	5	Amidase	Cytoplasmic	0
A1B5G5/Pden_2672	5	4	5	4	Aminotransferase	Cytoplasmic	1
A1B5G6/Pden_2673	5	4	8	7	DNA translocase FtsK	Membrane	12
A1B5H1/Pden_2678	19	25	23	24	Uncharacterized protein	Unknown	0
A1B5H2/Pden_2679	5	6	4	6	Surface antigen	OuterMembrane	0
A1B5H3/Pden_2680	3	3	3	3	Glucokinase	Cytoplasmic	4,8,7,12,14,2
A1B5H5/Pden_2682	17	17	17	13	Adenosylhomocysteinase	Cytoplasmic	4,12,14
A1B5H7/Pden_2684	4	5	5	4	Uncharacterized protein	Unknown	0
A1B5H8/Pden_2685	0	0	1	2	Short chain enoyl-CoA hydratase / Enoyl-CoA hydratase	Membrane	0
A1B5H9/Pden_2686	7	9	5	7	Uncharacterized protein	Periplasmic	0
A1B5I0/Pden_2687	4	6	6	6	Uncharacterized protein	Cytoplasmic	0
A1B5I2/Pden_2689	1	1	1	2	Flavin reductase domain protein, FMN-binding protein	Unknown	0
A1B5I4/Pden_2691	5	3	6	4	Uncharacterized protein	Unknown	0
A1B5I5/Pden_2692	4	5	2	5	Mammalian cell entry related domain protein	Unknown	0
A1B5J5/Pden_2705	6	5	7	5	Carbonic anhydrase	Cytoplasmic	15
A1B5J7/Pden_2707	7	9	7	8	Leucyl aminopeptidase	Cytoplasmic	0
A1B5J9/Pden_2709	3	2	2	3	Saccharopine dehydrogenase	Cytoplasmic	8,1,4,7,12,14
A1B5K4/Pden_2714	2	5	1	3	Peptidase M16 domain protein	Unknown	0
A1B5K5/Pden_2715	5	4	6	5	Peptidase M16 domain protein	Unknown	0
A1B5K6/Pden_2716	4	2	4	2	Uncharacterized protein	Unknown	0
A1B5K8/Pden_2718	18	18	17	14	Bifunctional purine biosynthesis protein PurH [Includes: Phosphoribosylaminoimidazolecarboxamide formyltransferase ; IMP cyclohydrolase]	Cytoplasmic	8,1,4,7,12,14
A1B5K9/Pden_2719	1	2	0	1	Heparinase II/III family protein	Cytoplasmic	0
A1B5L0/Pden_2720	5	2	1	1	Fmu domain protein	Cytoplasmic	9
A1B5L1/Pden_2721	2	1	1	1	Efflux transporter, RND family, MFP subunit	Membrane	12,5,13
A1B5L3/Pden_2723	10	11	9	8	Selenide, water dikinase	Cytoplasmic	8,1,4,7,12,14
A1B5L5/Pden_2725	1	2	1	1	Uncharacterized protein	Cytoplasmic	0
A1B5L6/Pden_2726	8	5	6	5	Protein-L-isoaspartate O-methyltransferase	Cytoplasmic	0
A1B5L7/Pden_2727	12	11	13	11	Type I secretion outer membrane protein, TolC family	OuterMembrane	0
A1B5L9/Pden_2729	7	8	7	10	Peptidase M23B	OuterMembrane	0
A1B5M0/Pden_2730	2	2	1	2	Protein-L-isoaspartate O-methyltransferase	Cytoplasmic	8,4,7
A1B5M1/Pden_2731	2	0	1	0	5'-nucleotidase SurE	Cytoplasmic	0
A1B5M6/Pden_2736	5	4	4	2	Endoribonuclease L-PSP	Unknown	0
A1B5M7/Pden_2737	3	2	0	1	Uncharacterized protein	Unknown	0
A1B5M8/Pden_2738	2	6	6	5	Chromosome partition protein Smc	Cytoplasmic	8,1,3,4,7,12
A1B5N3/Pden_2743	2	4	1	1	Elongation factor P	Cytoplasmic	0
A1B5P3/Pden_2753	4	4	3	5	5-carboxymethyl-2-hydroxymuconate delta-isomerase	Cytoplasmic	0
A1B5P5/Pden_2755	17	19	18	18	Aldehyde dehydrogenase	Cytoplasmic	0
A1B5P6/Pden_2756	10	11	10	9	1-Cys peroxiredoxin	Cytoplasmic	0
A1B5P9/Pden_2759	44	38	36	42	Polyribonucleotide nucleotidyltransferase	Cytoplasmic	6,8,4,7,2
A1B5Q0/Pden_2760	5	8	7	6	30S ribosomal protein S15	Cytoplasmic	6,1,8,4,7
A1B5Q2/Pden_2762	2	1	0	2	Alanine racemase domain protein	Cytoplasmic	0
A1B5Q3/Pden_2763	25	22	25	23	Outer membrane porin	OuterMembrane	0
A1B5Q4/Pden_2764	6	4	6	5	Uncharacterized protein	Unknown	0
A1B5Q5/Pden_2765	18	20	17	17	Leucine--tRNA ligase	Cytoplasmic	8,6,4,7,12,14

A1B5Q6/Pden_2766	2	1	2	1	Secreted periplasmic protein	Unknown	3,12
A1B5Q7/Pden_2767	2	1	0	0	DNA polymerase III, delta subunit	Cytoplasmic	6,1,8,4,7
A1B5R3/Pden_2773	2	1	1	1	Glycosyl transferase, group 1	Cytoplasmic	0
A1B5R5/Pden_2775	16	16	18	14	Aspartate-semialdehyde dehydrogenase	Cytoplasmic	8,1,4,7,12,14
A1B5R6/Pden_2776	12	11	16	12	Uncharacterized protein	Unknown	0
A1B5R7/Pden_2777	2	1	1	2	5'-methylthioadenosine/S-adenosylhomocysteine nucleosidase	Cytoplasmic	8,6,4,7,12,14
A1B5S1/Pden_2781	8	8	9	7	Two component transcriptional regulator, Fis family	Cytoplasmic	9
A1B5S2/Pden_2782	7	7	5	5	Histone family protein nucleoid-structuring protein H-NS	Unknown	9
A1B5S4/Pden_2784	9	12	8	7	Protein-export protein SecB	Cytoplasmic	25,3,5,23
A1B5S5/Pden_2785	1	1	2	1	FxsA cytoplasmic membrane protein	Membrane	0
A1B5S6/Pden_2786	10	10	12	9	Import inner membrane translocase, subunit Tim44	Unknown	0
A1B5T0/Pden_2790	10	5	5	7	ATP-dependent protease ATPase subunit HslU	Cytoplasmic	26
A1B5T1/Pden_2791	2	1	1	1	ATP-dependent protease subunit HslV	Cytoplasmic	8,7
A1B5T3/Pden_2793	5	5	4	2	Thioredoxin	Cytoplasmic	7,12,14,9,10,
A1B5T7/Pden_2797	1	1	0	2	Uncharacterized protein	Cytoplasmic	6,8,4,7
A1B5T8/Pden_2798	7	7	7	7	4-hydroxy-tetrahydrodipicolinate reductase	Cytoplasmic	8,1,4,7,12,14
A1B5U0/Pden_2800	2	1	2	1	Uncharacterized protein	Unknown	0
A1B5U1/Pden_2801	3	2	1	1	tRNA pseudouridine synthase B synthase)	Cytoplasmic	6,8,4,7
A1B5U3/Pden_2803	0	1	2	1	Beta-lactamase domain protein	Unknown	0
A1B5U7/Pden_2807	0	0	0	2	Uncharacterized protein	Cytoplasmic	0
A1B5V2/Pden_2812	1	1	3	1	Transcriptional regulator, BadM/Rrf2 family	Cytoplasmic	0
A1B5V3/Pden_2813	4	4	3	2	Shikimate dehydrogenase)	Cytoplasmic	8,1,4,7,12,14
A1B5W2/Pden_2822	5	4	4	5	Response regulator receiver protein	Cytoplasmic	1,6,8,4,7,9
A1B5W3/Pden_2823	1	2	2	1	2-nitropropane dioxygenase, NPD	Unknown	0
A1B5W6/Pden_2826	2	0	2	0	Protein FdhE homolog	Cytoplasmic	0
A1B5W9/Pden_2829	0	2	0	0	Formate dehydrogenase catalytic subunit	Periplasmic	4,12,14
A1B5X4/Pden_2834	21	17	17	17	DNA polymerase III subunit beta	Cytoplasmic	6,1,8,4,7
A1B5X6/Pden_2836	10	10	8	8	Adenine deaminase	Cytoplasmic	4,8,7,12,14,2
A1B5X8/Pden_2838	3	3	2	2	Uncharacterized protein	Unknown	0
A1B5X9/Pden_2839	5	5	4	3	Uncharacterized protein	Unknown	0
A1B5Y0/Pden_2840	5	3	1	3	Acyl-CoA dehydrogenase	Cytoplasmic	0
A1B5Y2/Pden_2842	2	2	2	2	Succinyl-diaminopimelate desuccinylase	Cytoplasmic	8,1,4,7,12,14
A1B5Y5/Pden_2845	7	9	5	8	Glutamate--cysteine ligase	Cytoplasmic	6,1,4,7
A1B5Y9/Pden_2849	0	0	3	2	PTS system fructose subfamily IIA component	Unknown	5,13
A1B5Z0/Pden_2850	1	0	2	1	Nucleotide-binding protein Pden_2850	Cytoplasmic	0
A1B5Z2/Pden_2852	20	20	17	20	Phosphoenolpyruvate carboxykinase	Cytoplasmic	1,8,7,14
A1B5Z3/Pden_2853	3	4	2	3	Ribonuclease R	Cytoplasmic	0
A1B600/Pden_2860	3	2	1	2	Phosphoribosylformylglycinamidine synthase subunit PurS	Unknown	8,1,4,7,12,14
A1B601/Pden_2861	7	7	10	7	Phosphoribosylaminoimidazole-succinocarboxamide synthase	Cytoplasmic	8,1,4,7,12,14
A1B602/Pden_2862	7	6	4	3	Uncharacterized protein	Cytoplasmic	0
A1B606/Pden_2866	2	1	2	2	Transcriptional regulator, MerR family	Cytoplasmic	9
A1B608/Pden_2868	7	4	4	4	Acyl-CoA dehydrogenase domain protein	Cytoplasmic	0
A1B610/Pden_2870	2	4	2	2	Acetyl-CoA acetyltransferase	Cytoplasmic	0
A1B611/Pden_2871	3	4	2	1	3-hydroxyacyl-CoA dehydrogenase	Cytoplasmic	4,8,7,12,14,2
A1B612/Pden_2872	6	5	5	5	PfkB domain protein	Cytoplasmic	0
A1B615/Pden_2875	9	10	9	10	OmpA/MotB domain protein	OuterMembrane	0

A1B616/Pden_2876	17	15	18	17	ATP synthase subunit b sector subunit b)	Unknown	7,4,12,14,5,13
A1B617/Pden_2877	7	9	9	4	ATP synthase subunit b sector subunit b)	Unknown	7,4,12,14,5,13
A1B622/Pden_2882	10	12	9	10	5'-Nucleotidase domain protein	Periplasmic	4,8,7,12,14,2
A1B643/Pden_2903	4	5	6	6	Aminodeoxychorismate lyase	Unknown	0
A1B644/Pden_2904	17	14	17	14	3-oxoacyl-[acyl-carrier-protein] synthase 2	Cytoplasmic	8,1,4,7,12,14
A1B646/Pden_2906	1	2	1	0	Acyl-CoA dehydrogenase domain protein	Cytoplasmic	0
A1B647/Pden_2907	3	2	2	3	Acetyl-CoA acetyltransferase	Cytoplasmic	0
A1B648/Pden_2908	0	3	1	2	Short-chain dehydrogenase/reductase SDR	Unknown	0
A1B652/Pden_2912	3	2	6	4	Replication protein, putative	Cytoplasmic	0
A1B653/Pden_2913	3	2	0	2	Cobyrinic acid a,c-diamide synthase	Cytoplasmic	0
A1B656/Pden_2916	2	2	1	2	Biotin synthase 2	Cytoplasmic	1,6,4,7,12,14
A1B658/Pden_2918	2	1	2	1	ATP-dependent dethiobiotin synthetase BioD	Cytoplasmic	1,6,4,7,12,14
A1B673/Pden_2933	15	16	15	16	Dihydroxy-acid dehydratase	Cytoplasmic	8,1,4,7,12,14
A1B678/Pden_2938	1	2	1	0	Efflux transporter, RND family, MFP subunit	Membrane	12,5,13
A1B683/Pden_2943	2	2	0	0	RND efflux system, outer membrane lipoprotein, NodT family	OuterMembrane	0
A1B684/Pden_2944	30	28	26	27	Phosphomethylpyrimidine synthase	Cytoplasmic	1,6,4,7,12,14
A1B686/Pden_2946	3	4	2	2	PfkB domain protein	Unknown	0
A1B688/Pden_2948	4	3	4	1	Antifreeze protein, type I	Cytoplasmic	0
A1B690/Pden_2950	1	2	1	2	Uncharacterized protein	Unknown	0
A1B691/Pden_2951	16	16	13	14	Toxic anion resistance family protein	Cytoplasmic	0
A1B695/Pden_2955	16	8	7	11	Carboxynorspermidine dehydrogenase	Cytoplasmic	0
A1B696/Pden_2956	4	2	2	3	Carboxynorspermidine/carboxyspermidine decarboxylase	Cytoplasmic	6,1,4,7
A1B697/Pden_2957	3	2	2	2	Oxidoreductase domain protein	Unknown	0
A1B698/Pden_2958	2	1	3	1	Transcriptional regulator, BadM/Rrf2 family	Unknown	0
A1B699/Pden_2959	3	2	3	2	Uncharacterized protein	Unknown	0
A1B6A1/Pden_2961	32	29	19	27	Isocitrate dehydrogenase [NADP]	Cytoplasmic	8,4,7,12,14
A1B6A2/Pden_2962	9	5	6	6	Phosphoribosylformylglycinamidine synthase subunit PurQ	Cytoplasmic	8,1,4,7,12,14
A1B6A4/Pden_2964	13	13	13	9	Two component, sigma54 specific, transcriptional regulator, Fis family	Cytoplasmic	1,6,8,4,7,9
A1B6A5/Pden_2965	24	21	21	23	Ribonuclease E	Cytoplasmic	6,8,4,7,2
A1B6A7/Pden_2967	2	1	1	1	Enoyl-CoA hydratase/isomerase	Cytoplasmic	0
A1B6A9/Pden_2969	11	12	9	11	50S ribosomal protein L13	Cytoplasmic	6,1,8,4,7
A1B6B0/Pden_2970	10	8	7	10	30S ribosomal protein S9	Cytoplasmic	6,1,8,4,7
A1B6E6/Pden_3007	1	2	1	2	TonB-dependent siderophore receptor	OuterMembrane	5,13
A1B6I4/Pden_3047	3	1	1	2	Transcriptional regulator, MarR family	Cytoplasmic	6,1,8,4,7
A1B6L9/Pden_3082	4	4	3	3	Outer membrane autotransporter barrel domain	OuterMembrane	0
A1B6M0/Pden_3083	2	1	1	0	Putative outer membrane protein	OuterMembrane	0
A1B6M1/Pden_3084	3	2	1	1	Lipoprotein, putative	Unknown	0
A1B6M9/Pden_3092	2	1	0	1	Uncharacterized protein	Cytoplasmic	0
A1B6U3/Pden_3156	1	2	1	1	Histone family protein nucleoid-structuring protein H-NS	Unknown	9
A1B6Z3/Pden_3206	3	2	3	2	Transcriptional regulator, GntR family	Cytoplasmic	6,1,8,4,7
A1B703/Pden_3216	5	3	4	4	Tartronate semialdehyde reductase	Cytoplasmic	0
A1B711/Pden_3224	2	1	2	1	Acyl-CoA dehydrogenase domain protein	Cytoplasmic	0
A1B716/Pden_3229	3	2	1	3	Uncharacterized protein	Cytoplasmic	0
A1B718/Pden_3231	3	2	3	2	Putative transcriptional regulator, IclR family	Cytoplasmic	1,6,8,4,7,9
A1B787/Pden_3305	2	1	0	1	Filamentation induced by cAMP protein Fic	Cytoplasmic	0
A1B7C8/Pden_3349	1	2	1	1	Nucleoid protein H-NS	Unknown	9

A1B7G9/Pden_3390	1	2	2	0	Uncharacterized protein	Unknown	0
A1B7K4/Pden_3427	4	1	1	1	TRAP dicarboxylate transporter-DctP subunit	Periplasmic	5
A1B7L4/Pden_3437	3	1	2	4	Fumarylacetoacetate hydrolase	Cytoplasmic	0
A1B7M2/Pden_3445	1	2	1	0	Efflux transporter, RND family, MFP subunit	Membrane	12,5,13
A1B7P7/Pden_3470	6	7	8	5	Uncharacterized protein	Unknown	0
A1B7X8/Pden_3552	16	12	14	14	ATP-dependent carboxylate-amine ligase domain protein, ATP-grasp	Cytoplasmic	0
A1B7X9/Pden_3553	11	9	7	5	NAD-dependent epimerase/dehydratase	Unknown	0
A1B7Y0/Pden_3554	1	3	1	1	Uncharacterized protein	Cytoplasmic	0
A1B7Y1/Pden_3555	4	7	5	5	Uncharacterized protein	Cytoplasmic	0
A1B7Y2/Pden_3556	16	13	13	14	N-acetylneuraminate synthase	Cytoplasmic	1,8,7,14
A1B7Y3/Pden_3557	15	12	11	10	DegT/DnrJ/EryC1/StrS aminotransferase	Cytoplasmic	0
A1B810/Pden_3586	4	3	3	3	Transglutaminase domain protein	Unknown	0
A1B811/Pden_3587	2	1	1	1	Uncharacterized protein	Membrane	0
A1B812/Pden_3588	3	4	1	3	Amidohydrolase	Cytoplasmic	0
A1B832/Pden_3609	15	11	13	10	Lon protease	Cytoplasmic	11,8,7,13
A1B835/Pden_3612	17	17	15	19	Isoleucine--tRNA ligase	Cytoplasmic	8,6,4,7,12,14
A1B841/Pden_3618	6	0	1	3	Glutathione S-transferase, C-terminal domain	Cytoplasmic	0
A1B843/Pden_3620	3	3	2	4	Adenine phosphoribosyltransferase	Cytoplasmic	8,1,4,7,12,14
A1B844/Pden_3621	3	1	0	0	Uncharacterized protein	Unknown	0
A1B852/Pden_3629	7	4	3	5	Uroporphyrinogen decarboxylase	Cytoplasmic	6,1,4,7
A1B853/Pden_3630	8	5	5	2	Porphobilinogen deaminase	Cytoplasmic	6,1,8,4,7
A1B856/Pden_3633	4	1	1	1	Isovaleryl-CoA dehydrogenase	Cytoplasmic	0
A1B859/Pden_3636	3	2	1	2	OmpW family protein	OuterMembrane	0
A1B862/Pden_3639	3	0	2	1	3-methylcrotonoyl-CoA carboxylase, alpha subunit	Cytoplasmic	0
A1B872/Pden_3649	12	13	8	11	DNA polymerase III, subunits gamma and tau	Cytoplasmic	6,1,8,4,7
A1B875/Pden_3652	3	3	3	2	ABC transporter related protein	Cytoplasmic	0
A1B876/Pden_3653	2	2	2	1	Cold-shock DNA-binding protein family	Cytoplasmic	9
A1B877/Pden_3654	58	62	55	57	60 kDa chaperonin	Cytoplasmic	25
A1B878/Pden_3655	1	3	1	1	Glycosyl transferase, group 1	Cytoplasmic	0
A1B880/Pden_3657	2	1	1	2	Uncharacterized protein	Unknown	0
A1B882/Pden_3659	11	10	8	7	Inorganic diphosphatase	Cytoplasmic	0
A1B883/Pden_3660	3	5	6	4	HAD-superfamily subfamily IIA hydrolase like protein	Cytoplasmic	0
A1B885/Pden_3662	1	2	1	0	Riboflavin biosynthesis protein	Cytoplasmic	8,1,4,7,12,14
A1B887/Pden_3664	6	4	4	5	L-threonine aldolase	Cytoplasmic	4,8,7,12,14,2
A1B890/Pden_3667	3	5	3	2	Bifunctional enzyme IspD/IspF [Includes: 2-C-methyl-D-erythritol 4-phosphate cytidylyltransferase ; 2-C-methyl-D-erythritol 2,4-cyclodiphosphate synthase]	Cytoplasmic	8,1,4,7,12,14
A1B891/Pden_3668	6	5	6	5	Ribose-5-phosphate isomerase A	Cytoplasmic	4,7,12,14
A1B892/Pden_3669	7	6	8	6	NADPH-glutathione reductase	Cytoplasmic	12,9,10
A1B893/Pden_3670	19	13	13	13	Protease FtsH subunit HflK	Cytoplasmic	0
A1B894/Pden_3671	17	19	18	19	Protease FtsH subunit HflC	Cytoplasmic	0
A1B896/Pden_3673	14	11	12	13	Aminopyrimidine aminohydrolase	Unknown	1,6,4,7,12,14
A1B8A1/Pden_3678	7	6	7	9	Homoserine dehydrogenase	Cytoplasmic	8,6,4,7,12,14
A1B8A2/Pden_3679	2	1	1	1	Uncharacterized protein	Cytoplasmic	0
A1B8A4/Pden_3681	6	11	7	8	Methylmalonyl-CoA mutase	Cytoplasmic	0
A1B8A7/Pden_3684	12	12	7	6	Biotin carboxyl carrier protein / biotin carboxylase	Cytoplasmic	0
A1B8A8/Pden_3685	2	2	2	2	Uncharacterized protein	Unknown	0

A1B8A9/Pden_3686	2	1	1	2	Uncharacterized protein	Unknown	0
A1B8B1/Pden_3688	4	4	3	2	Propionyl-CoA carboxylase carboxyltransferase subunit	Cytoplasmic	0
A1B8B2/Pden_3689	7	4	1	3	Alanine racemase	Cytoplasmic	8,1,4,7,12,14
A1B8B5/Pden_3692	11	8	7	7	PhoH family protein	Cytoplasmic	0
A1B8B7/Pden_3694	1	2	2	2	CBS domain containing protein	Membrane	0
A1B8B8/Pden_3695	5	4	4	3	GCN5-related N-acetyltransferase	Cytoplasmic	0
A1B8B9/Pden_3696	15	13	13	10	GMP synthase [glutamine-hydrolyzing]	Cytoplasmic	8,1,4,7,12,14
A1B8C0/Pden_3697	2	2	1	0	Thiamine monophosphate synthase	Cytoplasmic	1,6,4,7,12,14
A1B8C1/Pden_3698	1	2	1	0	tRNA -methyltransferase TrmJ /uridine-2'-O)-methyltransferase)	Cytoplasmic	6,8,4,7
A1B8C3/Pden_3700	19	14	18	14	Carboxypeptidase Taq, Metallo peptidase, MEROPS family M32	Cytoplasmic	0
A1B8C5/Pden_3702	5	4	4	5	Glutamate--putrescine ligase	Cytoplasmic	8,1,4,7,12,14
A1B8C8/Pden_3705	5	7	4	5	Amino acid ABC transporter substrate-binding protein, PAAT family	Periplasmic	0
A1B8C9/Pden_3706	4	2	3	1	Amino acid ABC transporter ATP-binding protein, PAAT family	Membrane	0
A1B8D3/Pden_3710	1	1	0	2	Uncharacterized protein	Unknown	0
A1B8D4/Pden_3711	19	21	14	14	CTP synthase	Cytoplasmic	8,1,4,7,12,14
A1B8D7/Pden_3714	6	7	4	4	Short-chain dehydrogenase/reductase SDR	Unknown	0
A1B8D9/Pden_3716	21	19	16	15	Citrate synthase	Cytoplasmic	8,4,7,12,14
A1B8E0/Pden_3717	16	16	13	14	Glutamate--tRNA ligase 2	Cytoplasmic	8,6,4,7,12,14
A1B8E8/Pden_3725	27	26	22	23	Elongation factor Ts	Cytoplasmic	0
A1B8E9/Pden_3726	16	20	14	17	30S ribosomal protein S2	Cytoplasmic	6,1,8,4,7
A1B8K7/Pden_3785	3	2	0	1	2-nitropropane dioxygenase, NPD	Unknown	0
A1B8K8/Pden_3786	38	40	32	26	30S ribosomal protein S1	Cytoplasmic	6,1,8,4,7
A1B8K9/Pden_3787	3	5	3	2	Integration host factor subunit beta	Cytoplasmic	1,6,8,4,7,9
A1B8L0/Pden_3788	2	1	0	2	Uncharacterized protein	Membrane	0
A1B8L1/Pden_3789	2	1	1	0	N-anthranilate isomerase	Unknown	8,1,4,7,12,14
A1B8L3/Pden_3791	12	7	8	7	Tryptophan synthase beta chain	Cytoplasmic	0
A1B8L6/Pden_3794	2	1	1	2	Uncharacterized protein	Cytoplasmic	0
A1B8L7/Pden_3795	5	5	4	4	Polysaccharide deacetylase	Cytoplasmic	6,1,8,4,7
A1B8M8/Pden_3806	9	9	9	7	Single-stranded DNA-binding protein	Cytoplasmic	4,8,7,12,14,13
A1B8N0/Pden_3808	2	2	1	2	Uncharacterized protein	Unknown	0
A1B8N1/Pden_3809	3	4	6	5	Delta-aminolevulinic acid dehydratase	Cytoplasmic	6,1,4,7
A1B8N3/Pden_3811	6	1	4	4	Transcription-repair-coupling factor	Cytoplasmic	3,9
A1B8N4/Pden_3812	10	11	10	9	ATP-dependent Clp protease, ATP-binding subunit clpA	Cytoplasmic	0
A1B8N5/Pden_3813	7	8	9	8	Hydroxyacylglutathione hydrolase	Cytoplasmic	4,7,12,14,2
A1B8N7/Pden_3815	13	10	4	10	ATP synthase subunit delta sector subunit delta)	Cytoplasmic	7,4,12,14,5,13
A1B8N8/Pden_3816	43	39	37	37	ATP synthase subunit alpha	Cytoplasmic	7,4,12,14,5,13
A1B8N9/Pden_3817	16	18	16	16	ATP synthase gamma chain	Membrane	7,4,12,14,5,13
A1B8P0/Pden_3818	31	32	31	32	ATP synthase subunit beta	Cytoplasmic	7,4,12,14,5,13
A1B8P1/Pden_3819	5	6	4	4	ATP synthase epsilon chain	Cytoplasmic	7,4,12,14,5,13
A1B8P3/Pden_3821	2	0	0	0	Alpha/beta hydrolase fold protein	Cytoplasmic	0
A1B8P4/Pden_3822	5	3	4	3	DSBA oxidoreductase	Cytoplasmic	0
A1B8P5/Pden_3823	11	9	12	13	Ribose-phosphate pyrophosphokinase	Cytoplasmic	8,1,4,7,12,14
A1B8P8/Pden_3826	4	4	2	1	RNA methyltransferase, TrmH family, group 3	Cytoplasmic	6,8,4,7
A1B8R1/Pden_3839	5	3	2	2	Uncharacterized protein	Cytoplasmic	6,11,8,4,7
A1B8R2/Pden_3840	3	7	4	2	Uncharacterized protein	Cytoplasmic	0
A1B8R3/Pden_3841	1	0	2	0	Helicase domain protein	Cytoplasmic	0

A1B8S2/Pden_3850	3	0	1	0	Uncharacterized protein	Unknown	0
A1B8S8/Pden_3856	0	1	1	2	Substrate-binding region of ABC-type glycine betaine transport system	Periplasmic	0
A1B8T3/Pden_3861	14	15	12	12	Pyridoxal-5'-phosphate-dependent enzyme, beta subunit	Cytoplasmic	8,1,4,7,12,14
A1B8T7/Pden_3865	15	20	18	14	DNA gyrase subunit A	Cytoplasmic	1,6,8,3,4,7
A1B8U4/Pden_3872	19	19	12	15	Glutamate dehydrogenase	Unknown	8,6,4,7,12,14
A1B8U5/Pden_3873	5	4	4	4	Crotonyl-CoA reductase	Cytoplasmic	0
A1B8U7/Pden_3875	4	3	2	1	Methylmalonyl-CoA mutase	Cytoplasmic	0
A1B8U9/Pden_3877	2	1	1	2	Alkyl hydroperoxide reductase AhpD	Unknown	0
A1B8V0/Pden_3878	14	8	10	10	Signal recognition particle protein	Membrane	12,3,5,13,23,22
A1B8V1/Pden_3879	2	1	2	1	GCN5-related N-acetyltransferase	Unknown	0
A1B8V2/Pden_3880	1	2	0	0	Chorismate mutase	Cytoplasmic	4,7,12,14
A1B8V3/Pden_3881	10	10	8	8	30S ribosomal protein S16	Cytoplasmic	6,1,8,4,7
A1B8V4/Pden_3882	3	2	3	1	Ribosome maturation factor RimM	Cytoplasmic	6,8,4,7,20
A1B8V5/Pden_3883	2	1	1	1	tRNA -)-methyltransferase	Cytoplasmic	0
A1B8V6/Pden_3884	15	10	13	13	50S ribosomal protein L19	Cytoplasmic	6,1,8,4,7
A1B8V7/Pden_3885	3	3	3	1	50S ribosomal protein L31	Cytoplasmic	6,1,8,4,7
A1B8V9/Pden_3887	2	0	0	1	Chromosome partitioning protein	Cytoplasmic	0
A1B8W2/Pden_3890	24	23	23	21	Acetyltransferase component of pyruvate dehydrogenase complex	Cytoplasmic	4,7,12,14
A1B8W3/Pden_3891	21	16	19	20	Transketolase, central region	Cytoplasmic	0
A1B8W4/Pden_3892	15	13	14	9	Pyruvate dehydrogenase E1 component subunit alpha	Cytoplasmic	1,4,7,12,14
A1B8W6/Pden_3894	1	1	1	2	Acetylornithine deacetylase or succinyl-diaminopimelate desuccinylase	Cytoplasmic	0
A1B8W9/Pden_3897	1	1	0	2	Deoxyribose-phosphate aldolase	Cytoplasmic	4,8,7,12,14,2
A1B8X0/Pden_3898	6	9	8	9	Aldehyde dehydrogenase)	Cytoplasmic	0
A1B8X3/Pden_3901	6	6	5	5	ATPase associated with various cellular activities, AAA_3	Cytoplasmic	0
A1B8X5/Pden_3903	1	1	1	2	Conserved hypothetical membrane protein	Membrane	0
A1B8X7/Pden_3905	3	1	1	4	Uncharacterized protein	Membrane	0
A1B8X8/Pden_3906	4	4	4	5	3-hydroxyacyl-[acyl-carrier-protein] dehydratase FabZ - hydroxymyristoyl-[acyl-carrier-protein] dehydratase) - hydroxymyristoyl-ACP dehydrase)	Cytoplasmic	8,1,4,7,12,14
A1B8X9/Pden_3907	7	8	5	7	Acyl-[acyl-carrier-protein]--UDP-N-acetylglucosamine O-acyltransferase	Cytoplasmic	8,1,4,7,12,14
A1B8Y0/Pden_3908	2	2	2	0	Uncharacterized protein	Unknown	0
A1B8Y2/Pden_3910	6	3	4	3	Endoribonuclease L-PSP	Cytoplasmic	0
A1B8Z8/Pden_3926	11	10	12	13	Phosphoribosylformylglycinamidine cyclo-ligase	Cytoplasmic	8,1,4,7,12,14
A1B900/Pden_3928	1	2	2	1	Amino acid/amide ABC transporter membrane protein 2, HAAT family	Membrane	0
A1B902/Pden_3930	7	7	7	9	Amino acid/amide ABC transporter ATP-binding protein 2, HAAT family	Unknown	0
A1B903/Pden_3931	9	8	6	9	Amino acid/amide ABC transporter ATP-binding protein 1, HAAT family	Membrane	0
A1B904/Pden_3932	22	21	14	18	Amino acid/amide ABC transporter substrate-binding protein, HAAT family	Periplasmic	0
A1B906/Pden_3934	19	14	18	12	Phospho-2-dehydro-3-deoxyheptonate aldolase	Unknown	8,1,4,7,12,14
A1B908/Pden_3936	3	2	1	1	Guanylate kinase	Cytoplasmic	0
A1B909/Pden_3937	3	2	2	3	Transferase hexapeptide protein	Cytoplasmic	0
A1B910/Pden_3938	3	1	1	2	PAS/PAC sensor signal transduction histidine kinase	Membrane	9
A1B911/Pden_3939	11	10	9	7	Amidophosphoribosyltransferase	Cytoplasmic	8,1,4,7,12,14
A1B912/Pden_3940	1	3	2	2	Colicin V production protein	Membrane	1,4,12,14
A1B915/Pden_3943	1	0	2	1	Pyrimidine 5'-nucleotidase	Cytoplasmic	0
A1B916/Pden_3944	6	5	2	2	Transcriptional regulator, GntR family	Cytoplasmic	6,1,8,4,7

A1B925/Pden_3953	9	10	8	6	Tryptophan--tRNA ligase	Cytoplasmic	8,6,4,7,12,14
A1B928/Pden_3956	4	4	1	2	Bifunctional uridylyltransferase/uridylyl-removing enzyme [Includes: [Protein-PII]-UMP uridylyl-removing enzyme ; [Protein-PII] uridylyltransferase]	Cytoplasmic	6,9
A1B929/Pden_3957	6	7	10	8	Amino acid/amide ABC transporter substrate-binding protein, HAAT family	Unknown	0
A1B930/Pden_3958	2	1	3	3	Ribosomal RNA small subunit methyltransferase I - methyltransferase RsmI)	Cytoplasmic	6,8,4,7,24
A1B932/Pden_3960	9	6	3	5	Glutathione synthetase	Cytoplasmic	0
A1B934/Pden_3962	14	13	14	15	Phosphopantothenoylcysteine decarboxylase / Phosphopantothenate-cysteine ligase	Cytoplasmic	4,8,7,12,14,2
A1B935/Pden_3963	4	3	3	3	Cysteine desulfurase	Unknown	0
A1B936/Pden_3964	12	15	8	11	Iron-regulated ABC transporter membrane component SufB	Cytoplasmic	1,3
A1B937/Pden_3965	2	0	0	0	UPF0145 protein Pden_3965	Unknown	0
A1B938/Pden_3966	12	9	11	9	FeS assembly ATPase SufC	Cytoplasmic	5
A1B939/Pden_3967	16	8	11	11	SufBD protein	Cytoplasmic	1,3
A1B942/Pden_3970	4	4	2	4	Cysteine desulfurase	Cytoplasmic	8,6,4,7,12,14
A1B943/Pden_3971	18	14	16	18	Phosphoribosylformylglycinamidine synthase subunit PurL	Cytoplasmic	8,1,4,7,12,14
A1B944/Pden_3972	6	4	6	4	Transcriptional regulator, LysR family	Cytoplasmic	6,1,8,4,7
A1B945/Pden_3973	6	5	5	6	Glutamate racemase	Cytoplasmic	12,7,14,9,10,19
A1B946/Pden_3974	2	6	2	3	Cytochrome c-type biogenesis protein CcmE	Unknown	8,3,4,7
A1B951/Pden_3979	1	2	1	1	Enoyl-CoA hydratase	Cytoplasmic	0
A1B952/Pden_3980	1	2	1	2	NUDIX hydrolase	Unknown	0
A1B953/Pden_3981	0	0	2	1	Uncharacterized protein	Membrane	0
A1B956/Pden_3984	14	14	15	12	Aminotransferase	Cytoplasmic	0
A1B962/Pden_3990	5	3	2	3	Ribosomal RNA large subunit methyltransferase E - methyltransferase)	Cytoplasmic	0
A1B965/Pden_3993	8	6	6	6	Uridylate kinase	Cytoplasmic	8,1,4,7,12,14
A1B966/Pden_3994	3	4	3	5	Ribosome-recycling factor	Cytoplasmic	3,12
A1B967/Pden_3995	0	0	1	2	Isoprenyl transferase	Cytoplasmic	0
A1B969/Pden_3997	0	0	2	0	1-deoxy-D-xylulose 5-phosphate reductoisomerase	Cytoplasmic	8,1,4,7,12,14
A1B970/Pden_3998	2	1	0	2	Zinc metalloprotease	Membrane	0
A1B971/Pden_3999	46	41	38	38	Outer membrane protein assembly factor BamA	OuterMembrane	3,12,5
A1B972/Pden_4000	2	1	1	1	Outer membrane chaperone Skp	Unknown	0
A1B973/Pden_4001	3	1	0	1	Purine nucleosidase	Cytoplasmic	0
A1B974/Pden_4002	2	1	1	0	Penicillin amidase	Unknown	1,4
A1B975/Pden_4003	2	1	1	1	3-hydroxyisobutyrate dehydrogenase	Unknown	0
A1B987/Pden_4015	8	6	7	6	Linocin_M18 bacteriocin protein	Unknown	11,29,28
A1B989/Pden_4017	18	17	17	18	Amino acid/amide ABC transporter substrate-binding protein, HAAT family	Unknown	0
A1B990/Pden_4018	9	13	13	12	Amino acid/amide ABC transporter membrane protein 1, HAAT family	Membrane	0
A1B992/Pden_4020	8	9	8	8	ABC transporter related protein	Membrane	0
A1B993/Pden_4021	11	9	6	5	Amino acid/amide ABC transporter ATP-binding protein 2, HAAT family	Membrane	0
A1B994/Pden_4022	2	1	2	2	Redoxin domain protein	Unknown	0
A1B995/Pden_4023	2	2	3	3	Phosphoglycolate phosphatase	Cytoplasmic	1,4,7,12,14
A1B997/Pden_4025	18	15	13	15	GTP-binding protein TypA	Membrane	0
A1B9A1/Pden_4029	13	10	9	9	Phosphoribosylamine--glycine ligase	Cytoplasmic	8,1,4,7,12,14
A1B9A2/Pden_4030	15	13	9	9	2-isopropylmalate synthase	Cytoplasmic	8,1,4,7,12,14
A1B9A4/Pden_4032	12	12	12	14	Rod shape-determining protein MreB	Cytoplasmic	3,12,31,17
A1B9B0/Pden_4038	4	3	2	4	Mannose-6-phosphate isomerase, type 2 / mannose-1-phosphate guanylyltransferase	Cytoplasmic	1,8,7,14

A1B9B1/Pden_4039	0	1	2	1	ABC-type nitrate/sulfonate/bicarbonate transport systems, periplasmic components TauA	Unknown	1,6,4,7,12,14
A1B9B4/Pden_4042	4	2	3	3	Uncharacterized protein	Cytoplasmic	0
A1B9B5/Pden_4043	0	2	0	0	ErfK/YbiS/YcfS/YnhG family protein	Unknown	0
A1B9B9/Pden_4047	5	6	4	5	NAD transhydrogenase subunit beta	Membrane	0
A1B9C0/Pden_4048	18	18	17	18	NAD transhydrogenase subunit alpha	Membrane	5,13
A1B9C3/Pden_4051	4	8	5	6	Malate synthase G	Cytoplasmic	8,4,7,12,14
A1B9C5/Pden_4053	13	15	9	11	Alanyl aminopeptidase, Metallo peptidase, MEROPS family M01	Unknown	0
A1B9C7/Pden_4055	2	1	1	2	Glutamate 5-kinase	Cytoplasmic	8,1,4,7,12,14
A1B9C8/Pden_4056	5	4	6	3	GTPase Obg	Cytoplasmic	20
A1B9D2/Pden_4060	25	23	28	23	Enolase	Cytoplasmic	4,8,7,12,14,2
A1B9D4/Pden_4062	7	6	6	8	2-isopropylmalate synthase	Cytoplasmic	8,1,4,7,12,14
A1B9D5/Pden_4063	4	1	3	4	Cysteine--tRNA ligase	Cytoplasmic	8,6,4,7,12,14
A1B9D8/Pden_4066	4	4	4	3	ABC transporter related protein	Membrane	0
A1B9E1/Pden_4069	2	0	0	0	Uncharacterized protein	Unknown	0
A1B9E3/Pden_4071	4	4	4	2	NADPH-dependent FMN reductase	Cytoplasmic	0
A1B9E4/Pden_4072	18	17	15	12	RNA polymerase sigma factor RpoD	Cytoplasmic	6,1,8,4,7
A1B9E5/Pden_4073	1	1	2	1	DNA primase	Cytoplasmic	0
A1B9E7/Pden_4075	4	3	4	2	L-lactate dehydrogenase	Cytoplasmic	0
A1B9E9/Pden_4077	12	12	10	12	50S ribosomal protein L25	Cytoplasmic	6,1,8,4,7
A1B9F0/Pden_4078	2	2	2	0	Methylenetetrahydrofolate--tRNA--methyltransferase TrmFO - methyltransferase) -methyltransferase)	Cytoplasmic	0
A1B9F2/Pden_4080	2	1	0	0	Phosphoribosyl-AMP cyclohydrolase	Cytoplasmic	8,1,4,7,12,14
A1B9F5/Pden_4083	4	5	2	3	DNA ligase])	Cytoplasmic	4,8,7,12,14,13
A1B9H0/Pden_4098	3	0	1	0	tRNA-specific 2-thiouridylase MnmA	Cytoplasmic	6,8,4,7
A1B9H1/Pden_4099	3	1	1	0	Cysteine desulfuration protein SufE	Cytoplasmic	0
A1B9H2/Pden_4100	1	2	2	2	Uncharacterized protein	Unknown	0
A1B9H4/Pden_4102	3	4	4	4	50S ribosomal protein L27	Cytoplasmic	6,1,8,4,7
A1B9H5/Pden_4103	6	6	5	5	50S ribosomal protein L21	Unknown	6,1,8,4,7
A1B9J3/Pden_4123	7	6	2	4	GTPase HflX	Cytoplasmic	0
A1B9J4/Pden_4124	1	0	2	0	RNA-binding protein Hfq	Cytoplasmic	9
A1B9J6/Pden_4126	2	2	2	0	TrkA-N domain protein	Membrane	0
A1B9J7/Pden_4127	4	5	5	3	Two component, sigma54 specific, transcriptional regulator, Fis family	Cytoplasmic	1,6,8,4,7,9
A1B9J8/Pden_4128	1	2	2	2	PAS/PAC sensor signal transduction histidine kinase	Membrane	9
A1B9J9/Pden_4129	28	25	25	26	Two component, sigma54 specific, transcriptional regulator, Fis family	Cytoplasmic	1,6,8,4,7,9
A1B9K0/Pden_4130	8	11	6	8	Signal transduction histidine kinase, nitrogen specific, NtrB	Membrane	0
A1B9K3/Pden_4133	2	3	3	6	FHA domain containing protein	Unknown	0
A1B9K4/Pden_4134	16	13	13	18	Uncharacterized protein	OuterMembrane	0
A1B9K5/Pden_4135	11	11	10	14	Uncharacterized protein	Unknown	0
A1B9K9/Pden_4139	1	2	1	1	Putative ferric uptake regulator, Fur family	Cytoplasmic	0
A1B9L0/Pden_4140	5	6	3	5	Periplasmic solute binding protein	Periplasmic	5,13
A1B9L4/Pden_4144	31	25	29	29	Pyruvate carboxylase	Cytoplasmic	8,1,4,7,12,14
A1B9Q5/Pden_4185	0	1	0	2	7-cyano-7-deazaguanine synthase synthase)	Unknown	8,1,4,7,12,14
A1B9S2/Pden_4202	3	3	5	4	Hemin-degrading family protein	Cytoplasmic	5,13
A1B9S9/Pden_4209	2	2	4	4	Phosphate butyryltransferase	Cytoplasmic	0
A1B9T1/Pden_4211	1	2	1	3	SSS sodium solute transporter superfamily	Membrane	12,5,13
A1B9T3/Pden_4213	10	10	11	6	Acetyl-coenzyme A synthetase	Cytoplasmic	1,4,7,12,14

A1B9T9/Pden_4219	9	12	11	11	Nitrous-oxide reductase OR)	Periplasmic	0
A1B9V6/Pden_4236	3	1	4	4	Respiratory nitrate reductase alpha subunit apoprotein	Membrane	6,4,7,12,14
A1B9W4/Pden_4244	1	0	3	1	Asparaginase	Unknown	8,6,4,7,12,14
A1B9X6/Pden_4256	1	3	2	4	Transcriptional regulator, LysR family	Cytoplasmic	6,1,8,4,7
A1B9X7/Pden_4257	1	3	1	3	Molybdopterin dehydrogenase, FAD-binding protein	Cytoplasmic	0
A1B9X8/Pden_4258	5	7	6	4	Xanthine dehydrogenase, molybdenum binding subunit apoprotein / Xanthine oxidase	Cytoplasmic	0
A1B9Y0/Pden_4260	2	0	1	1	Guanine deaminase	Cytoplasmic	4,8,7,12,14,2
A1B9Z2/Pden_4272	0	0	2	1	Amino acid/amide ABC transporter substrate-binding protein, HAAT family	Unknown	0
A1B9Z3/Pden_4273	4	4	3	2	Aminotransferase	Cytoplasmic	0
A1BA00/Pden_4280	2	1	2	1	Transcriptional regulator, IclR family	Cytoplasmic	1,6,8,4,7,9
A1BA12/Pden_4292	5	4	4	4	50S ribosomal protein L33	Cytoplasmic	6,1,8,4,7
A1BA15/Pden_4295	2	2	2	0	DNA helicase	Cytoplasmic	0
A1BA18/Pden_4298	16	15	14	12	O-succinylhomoserine sulfhydrylase	Cytoplasmic	8,1,4,7,12,14
A1BA21/Pden_4301	29	27	30	24	Aconitate hydratase	Cytoplasmic	0
A1BA23/Pden_4303	28	26	25	25	30S ribosomal protein S4	Cytoplasmic	6,1,8,4,7
A1BA25/Pden_4305	4	3	4	5	Triosephosphate isomerase	Cytoplasmic	4,8,7,12,14,2
A1BA26/Pden_4306	1	0	1	2	Methionine import ATP-binding protein MetN	Membrane	5,13
A1BA28/Pden_4308	2	2	4	2	Lipoprotein, YaeC family	Membrane	0
A1BA29/Pden_4309	2	0	0	1	Acetyl-CoA hydrolase	Cytoplasmic	4,7
A1BA32/Pden_4312	3	2	2	3	FAD linked oxidase domain protein	Cytoplasmic	0
A1BA34/Pden_4314	5	3	3	3	Peptidase M16 domain protein	Cytoplasmic	0
A1BA35/Pden_4315	17	14	13	10	L-threonine synthase	Cytoplasmic	0
A1BA41/Pden_4321	4	3	4	5	Cytochrome c oxidase subunit 2	Membrane	4,12,14
A1BA42/Pden_4322	8	7	6	9	Uncharacterized protein	Cytoplasmic	0
A1BA52/Pden_4332	5	3	8	5	Chorismate synthase	Cytoplasmic	8,1,4,7,12,14
A1BA61/Pden_4341	3	3	5	3	Sulfate-transporting ATPase	Membrane	0
A1BA64/Pden_4344	11	11	6	7	Sulfate ABC transporter, periplasmic sulfate-binding protein	Periplasmic	0
A1BA70/Pden_4350	6	6	7	6	Periplasmic glucan biosynthesis protein, MdoG	Periplasmic	1,8,7,14
A1BA86/Pden_4366	8	8	10	5	DEAD/DEAH box helicase domain protein	Cytoplasmic	0
A1BA93/Pden_4373	8	8	8	4	TonB-dependent siderophore receptor	OuterMembrane	5,13
A1BA97/Pden_4377	3	0	1	0	Ribonucleoside-diphosphate reductase	Cytoplasmic	6,1,8,4,7
A1BA98/Pden_4378	2	1	1	1	Ribonucleoside-diphosphate reductase subunit beta	Cytoplasmic	8,1,4,7,12,14
A1BAA0/Pden_4380	1	2	1	2	Putative transcriptional regulator, GntR family	Cytoplasmic	1
A1BAA2/Pden_4382	7	5	7	8	TonB-dependent receptor	OuterMembrane	5
A1BAA7/Pden_4387	0	2	1	1	Carbohydrate ABC transporter substrate-binding protein, CUT1 family	Periplasmic	0
A1BAB5/Pden_4395	10	7	8	9	Sulfate adenylyltransferase subunit 2	Cytoplasmic	1,4,12,14
A1BAB6/Pden_4396	13	14	7	11	Adenylyl-sulfate kinase	Cytoplasmic	4
A1BAC0/Pden_4400	0	0	1	2	Transcriptional regulator, GntR family	Cytoplasmic	6,1,8,4,7
A1BAC4/Pden_4404	5	4	4	1	Uncharacterized protein UPF0065	Unknown	0
A1BAC8/Pden_4408	1	0	2	3	5-oxoprolinase	Cytoplasmic	0
A1BAC9/Pden_4409	0	0	3	2	5-oxoprolinase	Unknown	0
A1BAD2/Pden_4412	3	1	0	2	Acyl-CoA dehydrogenase domain protein	Cytoplasmic	0
A1BAD6/Pden_4416	10	8	7	6	Succinate semialdehyde dehydrogenase	Cytoplasmic	0
A1BAD7/Pden_4417	8	7	6	5	Elongation factor 4	Membrane	9
A1BAE1/Pden_4421	3	3	3	2	Phosphonate metabolism protein PhnM	Cytoplasmic	4,7,2

A1BAE3/Pden_4423	12	11	10	10	Phosphoglucomutase/phosphomannomutase alpha/beta/alpha domain I	Cytoplasmic	8,7
A1BAE7/Pden_4427	1	2	0	0	Glucose-1-phosphate adenylyltransferase	Cytoplasmic	8,1,4,7,12,14
A1BAE8/Pden_4428	2	1	1	0	1,4-alpha-glucan branching enzyme GlgB -glucan branching enzyme)	Cytoplasmic	8,1,4,7,12,14
A1BAE9/Pden_4429	5	7	3	7	Alpha-1,4 glucan phosphorylase	Cytoplasmic	8,7
A1BAF0/Pden_4430	3	2	3	3	Nicotinate-nucleotide pyrophosphorylase	Cytoplasmic	8,1,4,7,12,14
A1BAF1/Pden_4431	0	2	1	1	L-aspartate oxidase	Cytoplasmic	0
A1BAF2/Pden_4432	2	1	0	0	Quinolinate synthase A	Cytoplasmic	8,1,4,7,12,14
A1BAF3/Pden_4433	2	1	2	2	Transcriptional regulator, IclR family	Cytoplasmic	1,6,8,4,7,9
A1BAG7/Pden_4447	14	13	11	11	Extracellular solute-binding protein, family 1	Periplasmic	0
A1BAG9/Pden_4449	29	31	30	29	Assimilatory nitrate reductase alpha subunit apoprotein	Cytoplasmic	0
A1BAH0/Pden_4450	6	5	6	6	Formate/nitrite transporter	Membrane	0
A1BAH1/Pden_4451	10	10	10	9	Assimilatory nitrite reductase H) small subunit	Unknown	6,4,7,12,14
A1BAH2/Pden_4452	59	53	50	54	Assimilatory nitrite reductase H) large subunit	Cytoplasmic	6,4,7,12,14
A1BAH3/Pden_4453	3	4	3	4	Major facilitator superfamily MFS_1	Membrane	12,5,13
A1BAH4/Pden_4454	1	1	3	2	Putative nitrate transport protein	Membrane	0
A1BAH5/Pden_4455	2	3	1	2	Response regulator receiver and ANTAR domain protein	Cytoplasmic	9
A1BAH8/Pden_4458	2	2	3	2	RNA binding S1 domain protein	Cytoplasmic	6,8,4,7
A1BAI1/Pden_4461	9	8	7	4	Nitrogen regulatory protein P-II	Membrane	1,6,8,4,7,9
A1BAI2/Pden_4462	44	40	32	31	Glutamine synthetase	Cytoplasmic	8,1,4,7,12,14
A1BAI3/Pden_4463	13	10	9	11	Adenylosuccinate lyase	Cytoplasmic	8,1,4,7,12,14
A1BAI5/Pden_4465	24	23	24	23	Glyceraldehyde-3-phosphate dehydrogenase	Cytoplasmic	8,7,14
A1BAI7/Pden_4467	5	5	4	3	Dihydroorotase	Cytoplasmic	8,1,4,7,12,14
A1BAI8/Pden_4468	9	6	6	6	Orotate phosphoribosyltransferase	Cytoplasmic	8,1,4,7,12,14
A1BAI9/Pden_4469	2	0	2	1	Replicative DNA helicase	Cytoplasmic	6,1,8,4,7
A1BAJ4/Pden_4474	6	5	5	2	Uncharacterized protein	Cytoplasmic	0
A1BAJ6/Pden_4476	7	7	4	3	Phosphopantetheine adenylyltransferase	Cytoplasmic	8,1,4,7,12,14
A1BAJ7/Pden_4477	7	7	6	7	Putative signal-transduction protein with CBS domains	Cytoplasmic	0
A1BAJ9/Pden_4479	11	13	5	13	Methylmalonate-semialdehyde dehydrogenase	Cytoplasmic	0
A1BAK2/Pden_4482	1	1	1	2	3-hydroxyisobutyrate dehydrogenase	Cytoplasmic	4,8,7,12,14,2
A1BAK4/Pden_4484	6	6	5	6	DNA repair protein RecN	Cytoplasmic	4,8,7,12,14,13
A1BAK5/Pden_4485	5	5	4	7	Outer membrane protein assembly factor BamD	Unknown	3,12,5
A1BAK7/Pden_4487	21	20	19	20	Cell division protein FtsZ	Cytoplasmic	3,12,30
A1BAK8/Pden_4488	0	0	0	2	Cell division protein FtsA	Cytoplasmic	12,9,10,30
A1BAL0/Pden_4490	11	9	8	11	D-alanine--D-alanine ligase	Cytoplasmic	12,7,14,9,10,19
A1BAL4/Pden_4494	6	6	7	5	UDP-N-acetylmuramate--L-alanine ligase	Cytoplasmic	12,7,14,9,10,19
A1BAL5/Pden_4495	8	7	3	6	UDP-N-acetylglucosamine--N-acetylmuramyl- pyrophosphoryl-undecaprenol N-acetylglucosamine transferase	Membrane	12,7,14,9,10,19
A1BAL9/Pden_4499	2	1	2	2	Homoserine O-succinyltransferase	Cytoplasmic	8,1,4,7,12,14
A1BAM2/Pden_4502	5	6	8	6	ABC polyamine transporter, periplasmic substrate-binding protein	Periplasmic	5,13
A1BAM3/Pden_4503	11	11	10	9	Gamma-glutamyl phosphate reductase	Cytoplasmic	8,1,4,7,12,14
A1BAM9/Pden_4509	3	5	1	3	Uncharacterized protein	Cytoplasmic	0
A1BAN0/Pden_4510	1	2	2	1	Probable protein kinase UbiB	Membrane	1,4,7,12,14
A1BAN3/Pden_4513	5	5	1	3	Short chain enoyl-CoA hydratase	Cytoplasmic	0
A1BAN4/Pden_4514	3	3	3	3	30S ribosomal protein S20	Cytoplasmic	6,1,8,4,7
A1BAN5/Pden_4515	3	3	2	4	3-demethylubiquinone-9 3-methyltransferase	Unknown	0
A1BAP1/Pden_4521	8	6	6	5	Uncharacterized protein	Unknown	0

A1BAR5/Pden_4546	4	5	4	5	TRAP dicarboxylate transporter-DctP subunit	Unknown	5
A1BAR6/Pden_4547	6	4	0	3	L-glutamine synthetase	Cytoplasmic	8,1,4,7,12,14
A1BAR7/Pden_4548	4	3	2	2	Aldehyde dehydrogenase	Cytoplasmic	0
A1BAR8/Pden_4549	4	4	4	4	Iron-containing alcohol dehydrogenase	Cytoplasmic	0
A1BAR9/Pden_4550	10	10	11	6	Acetyl-coenzyme A synthetase	Cytoplasmic	1,4,7,12,14
A1BAT6/Pden_4567	26	22	21	19	Aconitase	Cytoplasmic	8,4,7,12,14
A1BAU3/Pden_4574	2	0	0	0	Aldo/keto reductase	Cytoplasmic	0
A1BAV5/Pden_4586	8	9	10	9	10 kDa chaperonin	Cytoplasmic	25
A1BAW1/Pden_4592	2	1	1	1	Alkyl hydroperoxide reductase AhpD	Unknown	0
A1BAY7/Pden_4618	1	2	1	1	Asp/Glu racemase	Unknown	6
A1BAY8/Pden_4619	5	8	7	4	TRAP dicarboxylate transporter-DctP subunit	Periplasmic	5
A1BB09/Pden_4642	4	0	2	2	Uncharacterized protein	Unknown	0
A1BB12/Pden_4645	2	1	1	2	Transcriptional regulator, TetR family	Cytoplasmic	1,6,8,4,7,9
A1BB25/Pden_4658	2	5	4	2	Extracellular solute-binding protein, family 1	Unknown	0
A1BB40/Pden_4673	18	19	17	17	Band 7 protein	Membrane	0
A1BB44/Pden_4677	1	1	2	1	PKHD-type hydroxylase Pden_4677	Cytoplasmic	0
A1BB50/Pden_4683	7	6	6	5	3-oxoacid CoA-transferase, A subunit	Cytoplasmic	0
A1BB51/Pden_4684	6	2	3	4	Butyryl-CoA:acetate CoA transferase	Cytoplasmic	0
A1BB52/Pden_4685	2	2	1	1	Secretion protein HlyD family protein	Membrane	0
A1BB73/Pden_4706	2	2	2	2	Beta-lactamase domain protein	Cytoplasmic	0
A1BB75/Pden_4708	4	0	5	1	Glyoxalase/bleomycin resistance protein/dioxygenase	Cytoplasmic	0
A1BB77/Pden_4710	0	1	3	1	Glucose-methanol-choline oxidoreductase	Cytoplasmic	0
A1BB78/Pden_4711	3	3	3	2	Cell division topological specificity factor	Cytoplasmic	12,9
A1BB79/Pden_4712	17	13	12	13	Site-determining protein	Cytoplasmic	12
A1BB80/Pden_4713	5	5	6	3	Probable septum site-determining protein MinC	Cytoplasmic	3,12,9,31,17
A1BB82/Pden_4715	9	10	11	7	TRAP dicarboxylate transporter, DctP subunit	Periplasmic	5
A1BB88/Pden_4721	9	8	7	9	Periplasmic nitrate reductase	Periplasmic	8,1,4,7,12,14
A1BB94/Pden_4727	0	4	2	2	Glucose sorbosone dehydrogenase	Unknown	8,7
A1BBB0/Pden_4743	1	2	2	2	Peptidoglycan-binding domain 1 protein	Unknown	0
A1BBB2/Pden_4745	11	12	13	16	Sel1 domain protein repeat-containing protein	Unknown	0
A1BBC6/Pden_4759	2	1	2	1	Dihydrolipoamide acetyltransferase component of pyruvate dehydrogenase complex	Cytoplasmic	0
A1BBC7/Pden_4760	1	2	0	3	Dihydrolipoyl dehydrogenase	Cytoplasmic	12,7,14,9,2,10
A1BBD2/Pden_4765	2	3	2	2	C4-dicarboxylate transport protein	Membrane	5,13
A1BBF8/Pden_4791	2	3	3	0	Phospho-2-dehydro-3-deoxyheptonate aldolase	Unknown	8,1,4,7,12,14
A1BBF9/Pden_4792	1	1	3	1	3-hydroxyacyl-CoA dehydrogenase	Cytoplasmic	4,8,7,12,14,2
A1BBG9/Pden_4802	2	2	1	2	Transcriptional regulator, TetR family	Cytoplasmic	1,6,8,4,7,9
A1BBH9/Pden_4812	2	1	1	0	Transcriptional regulator, ArsR family	Cytoplasmic	0
A1BBI4/Pden_4817	2	1	0	1	TRAP dicarboxylate transporter, DctP subunit	Periplasmic	5
A1BBJ1/Pden_4824	3	2	1	2	Phosphoglucomutase/phosphomannomutase alpha/beta/alpha domain I	Cytoplasmic	8,7
A1BBL2/Pden_4846	1	1	2	1	Periplasmic binding protein/LacI transcriptional regulator	Cytoplasmic	6,1,8,4,7
A1BBN0/Pden_4864	4	2	2	1	Amino acid ABC transporter substrate-binding protein, PAAT family	Periplasmic	0
A1BBN1/Pden_4865	2	3	2	2	Aldehyde dehydrogenase	Cytoplasmic	0
A1BBN3/Pden_4867	2	2	1	2	Dihydrodipicolinate synthase	Cytoplasmic	0
A1BBN4/Pden_4868	2	2	1	2	Transcriptional regulator, GntR family	Cytoplasmic	6,1,8,4,7
A1BBQ2/Pden_4886	2	4	1	3	Glycine betaine/L-proline ABC transporter, ATPase subunit	Membrane	5,13

A1BBQ4/Pden_4888	1	3	2	3	Substrate-binding region of ABC-type glycine betaine transport system	Membrane	0
A1BBS1/Pden_4905	6	3	4	5	Bifunctional protein FolD 2 [Includes: Methylenetetrahydrofolate dehydrogenase ; Methenyltetrahydrofolate cyclohydrolase]	Cytoplasmic	8,1,4,7,12,14
A1BBT1/Pden_4915	0	1	2	1	Transcriptional regulator, DeoR family	Cytoplasmic	6,1,8,4,7
A1BC00/Pden_4984	3	3	2	2	Transketolase, central region	Cytoplasmic	0
A1BC11/Pden_4995	1	2	1	2	Transcriptional regulator, GntR family	Cytoplasmic	6,1,8,4,7
A1BC29/Pden_5013	2	1	1	0	Alkanesulfonate monooxygenase	Cytoplasmic	0
A1BC33/Pden_5017	3	2	0	2	Uncharacterized protein	Unknown	0
A1BC39/Pden_5023	5	4	3	4	NAD-dependent epimerase/dehydratase	Cytoplasmic	8,7
A1BC41/Pden_5025	4	2	1	2	Glycerol-3-phosphate cytidylyltransferase	Cytoplasmic	1,4,7,12,14
A1BC43/Pden_5027	7	10	7	9	CDP-glycerol:poly glycerophosphotransferase	Membrane	0
A1BC44/Pden_5028	6	5	4	2	Uncharacterized protein	Cytoplasmic	0
A1BC51/Pden_5035	3	3	0	0	Cobyrinic acid a,c-diamide synthase	Cytoplasmic	0
A1BC52/Pden_5036	3	6	4	2	ParB-like partition protein	Cytoplasmic	0
A1BC54/Pden_5038	8	4	9	8	Capsule polysaccharide export protein-like protein	Membrane	0
A1BC60/Pden_5044	2	1	1	0	Aldo/keto reductase	Cytoplasmic	0
A1BCB1/Pden_5095	13	9	12	12	Amino acid/amide ABC transporter substrate-binding protein, HAAT family	Periplasmic	0
A1BCD2/Pden_5116	0	2	2	2	Agmatinase	Cytoplasmic	0
A1BCE1/Pden_5125	2	2	0	1	Putative monooxygenase protein	Cytoplasmic	0
A1BCE3/Pden_5127	2	2	4	2	Transcriptional regulator, Fis family	Unknown	0
A1BCF0/Pden_5134	3	2	1	4	Uncharacterized protein	Unknown	0

[1]Protein annotated from UniProt (UP000000361). [2]Genes annotated from GeneBank (T00440). [3]Subcellular location according with PSOTb v3.0.2. [4]GOi (Gene Ontology respect to biological process at the third level), numbers refer to 0: unknown, 1: biosynthetic process, 2: catabolic process, 3: cellular component organization, 4: cellular metabolic process, 5: establishment of localization, 6: nitrogen compound metabolic process, 7: organic substance metabolic process, 8: primary metabolic process, 9: regulation of biological process, 10: regulation of biological quality, 11: response to stress, 12: single-organism cellular process, 13: single-organism localization, 14: single-organism metabolic process, 16: anatomical structure formation involved in morphogenesis, 17: anatomical structure morphogenesis, 18: cell adhesion, 19: cell wall organization or biogenesis, 20: cellular component biogenesis, 21: cellular detoxification, 22: cellular localization, 23: macromolecule localization, 24: methylation, 25: protein folding, 26: protein unfolding, 27: response to abiotic stimulus, 28: response to biotic stimulus, 29: response to external stimulus, 30: single organism reproductive process, 31: single-organism developmental process and 32: response to chemicals.
