# Supplementary figures and images for: Exploring the Denitrification Proteome of Paracoccus denitrificans PD1222
Source: Front Microbiol. 2018 May 29;9:1137. doi: 10.3389/fmicb.2018.01137 (PMC5987163; doi:10.3389/fmicb.2018.01137)

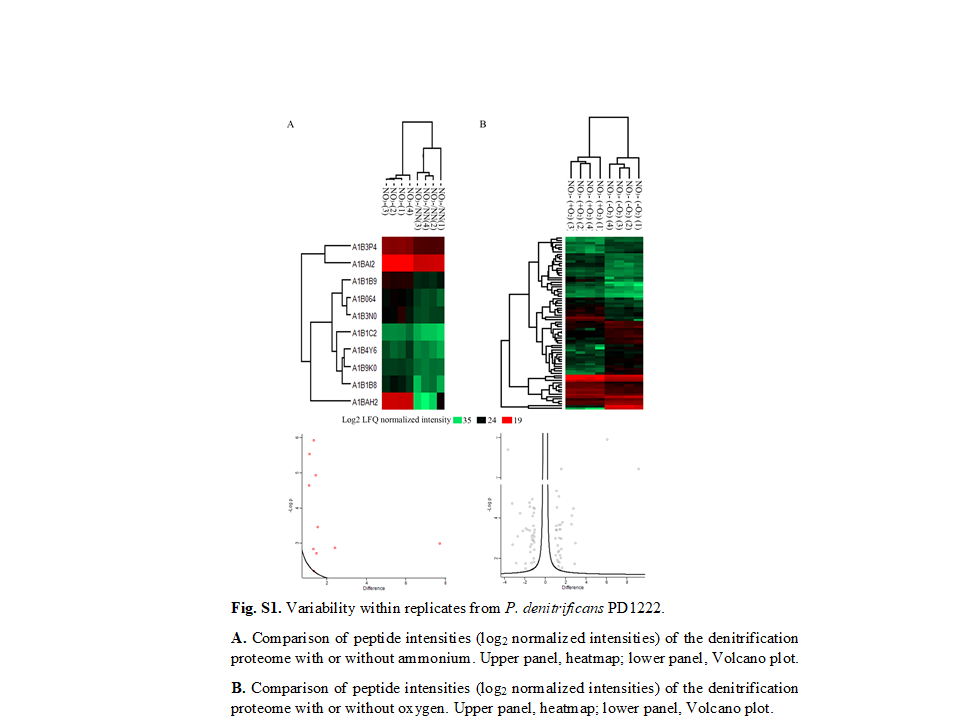

Supplement: Supplementary file 4 [file Image_1.TIF]
