# Supplementary material for: Exploring the Denitrification Proteome of Paracoccus denitrificans PD1222
Source: Front Microbiol. 2018 May 29;9:1137. doi: 10.3389/fmicb.2018.01137 (PMC5987163; doi:10.3389/fmicb.2018.01137)
Supplement: Supplementary file 5 [file Image_2.PDF]

ADDITIONAL INFORMATION FIGURE 2

A. Electron transport chain *norEDQ* (Pden_2480-Pden_2482), nitric oxide reductase *norBC* (Pden_2483, Pden_2484), FAD:component FMN transferase *norX* (Pden_2485), regulator *nirI* (Pden_2486), nitrite reductase *nirS* (Pden_2487), uroporphyrinogen-III C-methyltransferase *nirE* (Pden_2488), cytochrome c_{55x} *nirC* (Pden_2489), cytochrome d_1 heme *nirF* (Pden_2490), *nirD* (Pden_2491), transcriptional regulators *asnC* family (Pden_2492, Pden_2493), *S*-adenosylmethionine containing component (Pden_2494) and cytochrome d_1 heme gene (Pden_2495). **B**. Acetyl-coenzyme A synthetase *ascA* (Pden_4213), FMN transferase *nosX* (Pden_4214), *nosL* (Pden_4215), maturation *nosY* (Pden_4216), ABC transporter gene (Pden_4217), periplasmic copper binding component *nosD* (Pden_4218), nitrous oxide reductase *nosZ* (Pden_4219), FMN-binding regulator *nosR* (Pden_4220), *nosC* (Pden_4221), pseudoazurin (Pden_4222), uncharacterized (Pden_4223), *nnr* family (Pden_4224) and flavin prenyltransferase *ubiX* (Pden_4225). **C.** Periplasmic nitrate reductase *napEDABC* (Pden_4719-Pden_4723). **D.** Respiratory nitrate reductase *narIJHG* (Pden_4233-Pden_4236), nitrite transporter *narK* (Pden_4237) and transcriptional regulator Crp/Fnr-type *narR* (Pden_4238). **E.** Glutamate synthase *gltBD* (Pden_0488, Pden_0490) and uncharacterized (Pden_0489). **F.** Assimilatory nitrate reductase *nasC* (Pden_4449), formate/nitrite transporter *nasH* (Pden_4450), assimilatory nitrite reductase *nasGB* (Pden-4451, Pden_4452), major facilitator superfamily nitrate transporter *nasA* (Pden-4453), nitrate sensor *nasS* (Pden_4454), response regulator *nasT* (Pden_4455), MOSC domain (Pden_4456), pyridoxal kinase (Pden_4457), RNA binding S1 domain (Pden_4458), uncharacterized (Pden_4459), ADP-dependent dehydratase (Pden_4460), nitrogen regulatory PII *glnB* (Pden_4461) and glutamine synthetase *glnA* (Pden_4462). **G**. Two-component transcriptional regulator Fis family *ntrX* (Pden_4127), sensor signal transduction histidine kinase *ntrY* (Pden_4128), two-component transcriptional regulator *ntrC* (Pden_4129) and signal transduction histidine kinase *ntrB* (Pden_2130). **H.** Urease accessory *ureGFEJD* (Pden_1205-Pden_1207; Pden_1210; Pden_1212) and urease *ureABC* (Pden_1208, Pden_1209, Pden_1211). **I**. Succinate dehydrogenase components (Pden_0567-Pden_0569, Pden_0572) and uncharacterized (Pden_0570, Pden_0571). **J.** Heavy metal (copper) translocating P-type ATPase (Pden_1842), cytochrome cbb_3-type maturation *fixH* (Pden_1843), ferredoxin iron-sulfur binding (Pden_1844), cbb_3-type cytochrome *c*

oxidase subunits (Pden_1845-Pden_1848), USPA domain component (Pden_1849), transcriptional regulator Crp/Fpr-like *fnrP* (Pden_1850) and oxygen-independent coproporphyrinogen III oxidase (Pden_1851). **K.** NADH-quinone oxidoreductase components (Pden_2231-Pden_2235, Pden_2238, Pden_2239, Pden_2241, Pden_2243, Pden_2246-Pden_2248), carboxymuconolactone decarboxylase (Pden_2236) and uncharacterized (Pden_2237, Pden_2240, Pden_2242, Pden_2244, Pden_2245).
